# Meta-Analysis of the Effects of Foods and Derived Products Containing Ellagitannins and Anthocyanins on Cardiometabolic Biomarkers: Analysis of Factors Influencing Variability of the Individual Responses

**DOI:** 10.3390/ijms19030694

**Published:** 2018-02-28

**Authors:** María-Teresa García-Conesa, Karen Chambers, Emilie Combet, Paula Pinto, Mar Garcia-Aloy, Cristina Andrés-Lacueva, Sonia de Pascual-Teresa, Pedro Mena, Aleksandra Konic Ristic, Wendy J. Hollands, Paul A. Kroon, Ana Rodríguez-Mateos, Geoffrey Istas, Christos A. Kontogiorgis, Dilip K. Rai, Eileen R. Gibney, Christine Morand, Juan Carlos Espín, Antonio González-Sarrías

**Affiliations:** 1 Research Group on Quality, Safety and Bioactivity of Plant Foods, CEBAS-CSIC, P.O. Box 164, Campus de Espinardo, 30100 Murcia, Spain; jcespin@cebas.csic.es; 2 Quadram Institute Bioscience, Norwich Research Park, Norwich NR4 7UA, UK; Karen.Chambers@quadram.ac.uk (K.C.); wendy.hollands@quadram.ac.uk (W.J.H.); paul.kroon@quadram.ac.uk (P.A.K.); 3 Human Nutrition, School of Medicine, Dentistry and Nursing, College of Medical, Veterinary and Life Sciences, University of Glasgow, Glasgow G31 2ER, UK; emilie.combetAspray@glasgow.ac.uk; 4 Biotechnology and Nutrition, Department of Food Technology, ESA, Polytechnic Institute of Santarem, 2001-904 Santarém, Portugal; paula.pinto@esa.ipsantarem.pt; 5 Molecular Nutrition Health Laboratory, iBET/ITQB, 2780-157 Oeiras, Portugal; 6 Biomarkers and Nutrimetabolomic Laboratory, Department of Nutrition, Food Sciences and Gastronomy, XaRTA, INSA, Faculty of Pharmacy and Food Sciencies, University of Barcelona, 08028 Barcelona, Spain; margarcia@ub.edu (M.G.-A.); candres@ub.edu (C.A.-L.); 7 CIBER de Fragilidad y Envejecimiento Saludable (CIBERFES), Instituto de Salud Carlos III, 08028 Barcelona, Spain; 8 Department of Metabolism and Nutrition, Institute of Food Science, Technology and Nutrition (ICTAN-CSIC), Jose Antonio Novais 10, 28040 Madrid, Spain; s.depascualteresa@csic.es; 9 Human Nutrition Unit, Department of Food Drug, University of Parma, 43125 Parma, Italy; pedromiguel.menaparreno@unipr.it; 10 Institute for Medical Research, University of Belgrade, 11000 Belgrade, Serbia; a.konicristic@leeds.ac.uk; 11 School of Food Science and Nutrition, University of Leeds, Leeds LS2 9JT, UK; 12 Department of Nutritional Sciences, School of Life Course Sciences, Faculty of Life Science and Medicine, King’s College London, London SE1 9NH, UK; ana.rodriguez-mateos@kcl.ac.uk (A.R.-M.); g.istas@kcl.ac.uk (G.I.); 13 Laboratory of Hygiene and Environmental Protection, Department of Medicine, Democritus University of Thrace, 68100 Alexandroupolis, Greece; ckontogi@med.duth.gr; 14 Teagasc Food Research Centre Ashtown, D15 KN3K Dublin, Ireland; dilip.rai@teagasc.ie; 15 UCD Institute of Food and Health, University College Dublin, Dublin 4, Ireland; eileen.gibney@ucd.ie; 16 INRA, Human Nutrition Unit, UCA, CRNH Auvergne, F-63000 Clermont-Ferrand, France; christine.morand@inra.fr

**Keywords:** ellagitannins, anthocyanins, interindividual variability, meta-analysis, cardiometabolic disorders, pomegranate, nuts, berries, red wine, red grapes

## Abstract

Understanding interindividual variability in response to dietary polyphenols remains essential to elucidate their effects on cardiometabolic disease development. A meta-analysis of 128 randomized clinical trials was conducted to investigate the effects of berries and red grapes/wine as sources of anthocyanins and of nuts and pomegranate as sources of ellagitannins on a range of cardiometabolic risk biomarkers. The potential influence of various demographic and lifestyle factors on the variability in the response to these products were explored. Both anthocyanin- and ellagitannin-containing products reduced total-cholesterol with nuts and berries yielding more significant effects than pomegranate and grapes. Blood pressure was significantly reduced by the two main sources of anthocyanins, berries and red grapes/wine, whereas waist circumference, LDL-cholesterol, triglycerides, and glucose were most significantly lowered by the ellagitannin-products, particularly nuts. Additionally, we found an indication of a small increase in HDL-cholesterol most significant with nuts and, in flow-mediated dilation by nuts and berries. Most of these effects were detected in obese/overweight people but we found limited or non-evidence in normoweight individuals or of the influence of sex or smoking status. The effects of other factors, i.e., habitual diet, health status or country where the study was conducted, were inconsistent and require further investigation.

## 1. Introduction

Cardiometabolic dysfunction is diagnosed in about 8% of the global adult population and is characterized by dyslipidemia, hypertension, obesity, glucose intolerance and insulin resistance [1]. It is well recognized that a diet rich in plant-based foods helps prevent or reduce these cardiometabolic disorders and that increasing the intake of fruits, vegetables, cereals and nuts constitutes part of the strategy to combat these disorders [2,3]. Plant foods provide a variety of micro- and macronutrients, i.e., minerals, vitamins, fibers, proteins as well as a range of bioactive compounds that are beneficial for our health [4]. Over the past few decades, a major area of research has specifically focused on the study of the plant-derived bioactive compounds, such as polyphenols, and their cardiometabolic protective properties in humans which is summarized by Lecour et al. [5].

A variety of fruits and nuts are good sources of different polyphenols including anthocyanins (ANCs) and ellagitannins (ETs). In particular, berries, red grapes and red wine are important sources of ANCs [6,7]. The main ANCs present in our diet are cyanidin, delphinidin, malvidin, pelargonidin, peonidin, and petunidin [8,9]. Cyanidin-3-glucoside is the most frequent ANC found in raspberries, blackberries, elderberries, purple corn or black carrots. Moreover, malvidin-3-glucoside is the major ANC in red grapes and wines whilst pelargonidin-3-glucoside in strawberries. Blueberries, very often used in intervention studies, contain a mixture of delphinidin, malvidin and cyanidin derivatives [8,10]. The highest ANCs content is found in chokeberry (400 to 1500 mg/100 g fresh weight (F.W.)), blackcurrant (100 to 500 mg/100 g F.W.), blackberries (50 to 350 mg/100 g F.W.), blueberries (60 to 300 mg/100 g F.W.) and purple corn (≥1500 mg/100 g F.W.) [8,11]. On the other hand, pomegranate and nuts contain important quantities of ETs (150–500 mg/100 mL pomegranate juice; ~1600 mg/100 g walnuts) although they can also be found in berries at lower concentrations (50–350 mg/100 g F.W. raspberry; 25–80 mg/100 g F.W. strawberry) [12,13,14]. The ETs most commonly ingested by humans are punicalagin, peduncalagin and sanguiin [15].

Previous reviews and meta-analyses of randomized-controlled trials (RCTs) have explored the evidence of the effects of the intake of berry [16,17,18,19,20,21], nut [22,23], pomegranate [24,25], and grape [26,27] foods and (or) derived products on different cardiometabolic risk factors (i.e., serum lipids, blood pressure, glucose). The results of these analyses have indicated inconsistencies in the overall effects and have pointed at potential different responses between different subpopulations. Some of the reasons for the lack of consistent results might be the insufficient number of the RCTs included in these meta-analyses as well as their heterogeneity and inadequate description of the study population. There are important differences between studies for a number of key factors (body mass index (BMI), sex, smoking habits, diet, health status) that likely influence the response of the participants to the intake of the compounds tested. These differences can mask significant effects in specific populations [28,29].

Another critical issue is the test products used in those RCTs. In general, these studies have been conducted with different types of foods or derived processed products (extracts, drinks, freeze-dried products) with different origin, quality and composed by mixtures of diverse components often not fully characterized. It is thus difficult to attribute the beneficial effects to a particular compound(s). In this regard, some studies have been conducted with polyphenol-rich berry products in comparison with nutrient-matched controls to try to associate the polyphenols intake with the response of the individuals [30]. Also, the association between the intake of ET-containing pomegranate products and the effects on cardiometabolic risk factors has been investigated with some evidence of a potential link between responses and specific ET-derived metabolites, but the effects of other constituents of these products have not been fully discarded [31,32]. At present, specific associations between pure ANCs or ETS and cardiometabolic effects remain unproved. 

As the number of RCTs published increases, it is thus important that: (i) we continue evaluating the accumulated overall evidence that support the benefits of the plant bioactive compounds on cardiometabolic health and (ii) we try to elucidate the contribution of the factors that determine interindividual variability in response to the intake of these plant-derived bioactive compounds. Eventually, we shall be able to understand and establish the true effectiveness of these compounds against cardiometabolic disease [33]. Along these lines, the main goals of the present study were: (i) to systematically review and appraise through meta-analysis, all available human RCTs that have investigated the association between the intake of various foods (berries, red grapes and wine, pomegranate and nuts) as well as of their derived products (extracts, powders, drinks), as sources of ANCs and ETs, on 13 biomarkers of cardiometabolic risk; and (ii) to provide an in-depth evaluation of the potential influence of a range of key factors on the interindividual variability in response to the intake of these products.

## 2. Results

### 2.1. Description of the Selected Studies

**Figure 1:**
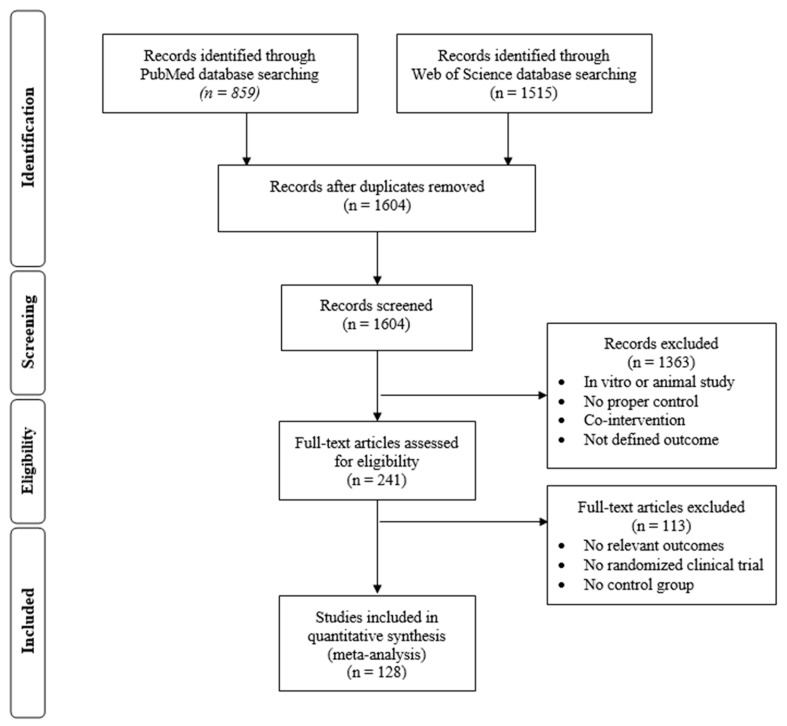
Flow diagram showing the study selection process.

A total of 2374 articles were initially selected through the search on the electronic databases (Medline (PubMed) and Web of Science). After removal of duplicates, screening and application of exclusion criteria, 241 trials were selected for data extraction. After detailed analysis of the full text, 113 articles were rejected, due to lack of relevant outcomes, aspects of the study design, etc. Finally, 128 human RCTs published between March 1995 and March 2016 (included) [34,35,36,37,38,39,40,41,42,43,44,45,46,47,48,49,50,51,52,53,54,55,56,57,58,59,60,61,62,63,64,65,66,67,68,69,70,71,72,73,74,75,76,77,78,79,80,81,82,83,84,85,86,87,88,89,90,91,92,93,94,95,96,97,98,99,100,101,102,103,104,105,106,107,108,109,110,111,112,113,114,115,116,117,118,119,120,121,122,123,124,125,126,127,128,129,130,131,132,133,134,135,136,137,138,139,140,141,142,143,144,145,146,147,148,149,150,151,152,153,154,155,156,157,158,159,160] were incorporated in this systematic review and meta-analysis. A flow diagram with the details of the study selection is shown in Figure 1.

### 2.2. Characteristics and Quality of the Included Studies

The 128 RCTs included a total of 5538 participants from countries distributed over five continents as follows: 1500 participants from Asia, 1731 from North-America, 119 participants from South America (Chile), 1830 participants from Europe, 64 participants from South Africa, and 294 participants from Oceania (Australia and New Zealand). Of all the studies, 30 RCTs (1542 total participants) were conducted with foods and derived products containing ETs as the main polyphenols, i.e., pomegranate and nuts (walnuts, almond, pistachios, peanuts, pine nuts, hazelnuts) and 99 RCTs (4086 total participants) were conducted with foods and food products considered rich sources of ANCs (berries, red grapes, red wine). The test products were provided as a liquid (drinks, beverages, juices) or a solid (powder or extracts in capsules, tablets, foods). Intervention doses ranged between 30 g and 230 g for nuts, between 100 mL and 500 mL for pomegranate juice and between 435 mg and 700 mg for pomegranate extracts. RCTs conducted with berries had doses ranging from 80 mg to 38 g for extracts and from 230 mL to 750 mL for beverages or juices. Regarding red wines or grapes doses ranged from 100 mg to 2 g for extracts and from 250 to 400 mL for drinks. The participants in these RCTs represented a mixed population of men and women ranging from young adults to elderly participants, and with a higher prevalence (~60%) of individuals with a BMI ≥ 25.0 kg/m^2^ (overweight and (or) obese volunteers). Only 28 and 9 RCTs were conducted separately with men and women, respectively. Most studies failed to report the smoking habits of participants; when reported, participants were typically non-smokers (~35%) or a mixed sample population (~50%). With regards to the health status, the sample population constituted of healthy individuals (1764 participants), overweight and (or) obese individuals but not medicated (classified as ‘at risk’; 870 participants), as well as individuals with an incipient or with a reported chronic risk factor or metabolic disease (2804 participants). Among these, some participants were taking medication, others were not medicated or medication use was not reported. Most interventions (~80%) ranged from 1 week to 3 months during which the participants followed either a controlled diet (~40%) or their habitual diets (~60%). RCTs conducted for more than 3 months or acute studies represented each ~10% of the total number of interventions. Most of the studies (~62%) were classified as studies with a moderate to low risk of bias (score ≥ 5.0 and <8.0 or ≥8.0 and ≤10.0, respectively), while ~38% of the studies obtained a low score (<5.0) and were considered as a high risk of bias. A list of all the studies included in this meta-analysis, their characteristics and corresponding risk bias score is included in Supplementary Table S1.

### 2.3. Overall Impact of Supplementation with Foods and Derived Products Containing ETs and/or ANCs on Biomarkers of Cardiovascular Risk

Initial analysis examined the effects of the supplementation with ET- and/or ANC-containing foods and/or derived products on the list of selected biomarkers of cardiometabolic risk at a total population level. A substantial number of RCTs (18 to 109 depending on the variable investigated) including a total number of participants (*n* = 563 to 3991) with highly variable heterogeneity (*I*^2^ ≈ 25.0 to 93.0%) were included in the analyses. Forest plots and Funnel plots representing all the individual studies and the overall impact of the supplementation with these products on each biomarker are shown in Supplementary Figures S1–S39. Visual inspection of the Funnel plots showed symmetrical shapes and absence of publication bias in most of the variables investigated, however, we detected some asymmetry in the case of LDL-C, HDL-C, TAGs, and FMD (SDM values) and of DBP (DM values). All these results were further confirmed by Egger’s regression. Supplementary Tables S2 and S3 display the overall results as standardized difference in means (SDM) and difference in means (DM), respectively, using the random model. A summary listing the significant effects expressed as SDM and DM values as well as their corresponding 95% confidence intervals and GRADE quality of evidence is presented in Table 1.

Among the 13 cardiometabolic outcomes investigated, we observed a significant evidence of the reduction in WC, T-C, SBP and DBP following supplementation with the ET- and/or ANC-containing products. Additionally, we detected an increase in HDL-C further supported by a significant although small positive relationship with the duration (days) of the supplementation (SDM random-effects meta-regression; slope: 0.002; *p*-value: 0.004) (Supplementary Figure S40). FMD was also consistently increased by the treatment with these compounds although it was only statistically significant when the effects were calculated as DM. The quality of the evidence was evaluated as high for blood pressure, moderate for WC, T-C, HDL-C and low for FMD due to many studies reporting serious risk of bias across studies. We additionally detected a small reduction, although not significant, in glucose (SDM: −0.101, *p*-value = 0.095) and TAGs (DM: −0.006, *p*-value = 0.086) whereas BMI, LDL-C, insulin, HbA1c, and HOMA-IR were seemingly not affected by the intervention with these types of products.

We next compared the effects on all the biomarkers between those foods and/or products that are richer sources of ETs, nuts and pomegranate (subgroup referred as to ETs) and those foods and products that contain higher levels of ANCs, mostly berries, red grapes, red wine (subgroup referred as to ANCs). Supplementary Table S4 includes the random effects (SDM and DM) for each separate group and the comparison between them (ETs vs. ANCs sources) for all the investigated biomarkers. A summary with the most significant effects is presented in Table 2.

Overall, there was a higher number of clinical trials looking at the cardiometabolic regulatory effects of ANC-rich berries, grapes and wine (*n* = 99) than studies carried out with pomegranate or nuts (*n* = 30). Regardless of the number of studies per subgroup, stratification into these two types of products did not alter the significant effect on T-C levels. However, the analysis of the separate subgroups highlighted some dissimilarity between the supplementation with the two types of products, with the ET-containing pomegranate and nuts being more effective (i.e., greater effect size and more significant results) than the ANCs subgroup at reducing WC (subgroups comparison (SDM): Q statistic = 6.70, *p*-value = 0.01), LDL-C, TAGs (subgroups comparison (SDM): Q statistic = 4.06, *p*-value = 0.044) and glucose. On the other hand, the ANC-rich products were significantly effective at lowering both SBP and DBP, whereas this was not the case in the ETs subgroup. We cannot ignore, however, that this may be due to the smaller number of studies carried out with the ETs-containing products. Also, probably due to the smaller number of studies per subgroup, the initially observed significant increase in HDL-C (all products together) was not significant in each separate subgroup. In addition, we found a significant increase in FMD (SDM) in the ETs subgroup, though these results should be interpreted cautiously since the number of trials was very small (*n* = 3). Given some of the differences found between the ETs and ANCs subgroups, we next carried out the rest of the stratification analyses in each separate subgroup.

### 2.4. Comparative Analysis of the Potential Factors Influencing Interindividual Variability in the Responses to the Consumption of Foods and Food Products Containing ETs and ANCs

#### 2.4.1. Stratification by the Individuals’ Baseline BMI, Sex, Smoking Habits and Background Diet

Stratification by baseline BMI values: <25.0 Kg/m^2^ (normal individuals) vs. ≥25.0 Kg/m^2^ (overweight and obese individuals) (Supplementary Table S5) evidenced a general absence or a small number of studies carried out in normal individuals in the ETs subgroup (≤3) and in the ANCs subgroup (between 5 and 13) for most of the biomarkers investigated. Neither ETs nor ANCs had any effect on the biomarkers investigated in the normoweight subpopulation. The most noteworthy effects of the ETs- and ANCs-containing products in overweight and obese individuals are summarized in Table 3.

The reduction of T-C by these two types of products remained significant in the individuals with a baseline BMI ≥ 25.0 Kg/m^2^ although the extent of the reduction appeared to be smaller in the ETs subgroup. The reduction of WC, LDL-C, TAGs and glucose by the ETs-containing pomegranate and nuts in the total population (Table 2) was still seen in the overweight/obese subgroup. For FMD, we found that the 3 studies included in the analysis of the ETs subgroup were all carried out in overweight/obese individuals and thus the results are the same as in Table 2. Regarding blood pressure, the ANC-containing products were also effective at lowering SBP and DBP in the overweight/obese people. Of note, the ET-products which did not significantly affect blood pressure in the overall population group (Table 2) became effective and significant at lowering the SBP in the overweight/obese people. 

Regarding sex stratification, there were also few studies specifically carried out with either only men (*n* = 1–24) or women (*n* = 1–9) depending on the product and biomarker and thus, the evidence for the effect of sex in the response to ETs or ANCs was limited and most results were not significant (Supplementary Table S6). We found, however, that the reduction of DBP in men by ANCs was still significant (SDM: −0.19, *p*-value = 0.017, *n* = 24; DM: −1.70 mm Hg, *p*-value = 0.012, *n* = 22) whereas in women the results did not reach significance (SDM: −0.19, *p*-value = 0.092, *n* = 8; DM: −1.81 mm Hg, *p*-value = 0.087, *n* = 8), possibly due to the small number of studies in the women subgroup.

We did not find any study carried out specifically with smokers in the ETs subgroup and only very few studies (*n* = 1–3) in the ANCs subgroup (Supplementary Table S7). Most studies were conducted with non-smokers or in a mix population. The ET-containing products reduced WC (DM = −1.72, *p*-value = 0.047, *n* = 3), DBP (SDM: −0.27, *p*-value = 0.035, *n* = 9), and glucose (DM = −0.11, *p*-value = 0.000, *n* = 5) in the non-smokers subgroup. In the ANCs subgroup, the reduction of both SBP and DBP remained significant for non-smoker individuals. FMD was significantly reduced by the ANC-containing products in smokers (SDM = −1.61, *p*-value = 0.000; DM = −3.53%, *p*-value = 0.000). Statistical comparison between smokers vs. non-smokers confirmed the difference in the effect between the two subgroups ((SDM): Q statistic = 5.93, *p*-value = 0.015; (DM): Q statistic = 4.84, *p*-value = 0.028). These results should be interpreted with caution due to the small number of studies included.

Studies were also stratified according to the type of background diet (controlled vs. usual) followed during the supplementation period with the ET- or the ANC-containing products (Supplementary Table S8). In most cases, the results of the analyses were not significant with independence of the type of diet followed during the intervention. The reduction of T-C by the products containing ETs were significant both in trials carried out with the usual diet, as well as those carried out with controlled diet whereas the levels of TAGs and the WC were most significantly reduced only in the subgroup that followed a controlled diet (subgroups comparison (SDM): Q statistic = −4.66, *p*-value = 0.031 for TAGs; (SDM): Q statistic = 19.52, *p*-value < 0.001 and (DM): Q statistic = 20.32, *p*-value < 0.001 for WC). Additionally, we found some indication of the increase of FMD in studies carried out with the usual diet (SDM: +0.62, *p*-value = 0.014, *n* = 3) and of the decrease of LDL-C (DM: −0.11 mmol/L, *p*-value = 0.000, *n* = 9) and glucose (DM: −0.15, *p*-value = 0.038, *n* = 8) under a controlled diet.

Regarding supplementation with ANCs, the reduction of SBP and DBP by these compounds was significant both in interventions maintaining the usual diet and in those carried out with controlled diets. We additionally detected a significant decrease in the LDL-C levels but only in the subgroup that followed the usual diet (SDM: −0.31, *p*-value = 0.027; DM: −0.22 mmol/L, *p*-value = 0.016, *n* = 16–17).

#### 2.4.2. Stratification by the Health Status of the Participants

As for the previous factors examined, stratification of the studies by reported health status of the participants reduced considerably the number of studies in many of the resulting subgroups, as well as the significance of the results (Supplementary Table S9a,b). 

In the subgroup containing studies with healthy participants, the evidence of the effects of the ET-containing pomegranate and nuts was limited to a significant reduction of T-C (SDM: −0.21, *p*-value = 0.021; DM: −0.15 mmol/L, *p*-value = 0.028, *n* = 10) and of glucose (SDM: −0.62, *p*-value = 0.023; DM: −0.23 mmol/L, *p*-value = 0.016, *n* = 5) (Supplementary Table S9a). In those studies carried out with volunteers ‘at risk’, the ET-containing products reduced LDL-C (SDM: −0.55, *p*-value = 0.030; DM: −0.11 mmol/L, *p*-value = 0.000, *n* = 4) and SBP (SDM: −0.40, *p*-value = 0.004; DM: −1.15 mm Hg, *p*-value = 0.003, *n* = 4). There was also a small reduction in T-C (DM: −0.08 mmol/L, *p*-value = 0.000, *n* = 4) and in TAGs (DM: −0.11 mmol/L, *p*-value = 0.000, *n* = 4) and an increase in HDL-C (SDM: +0.35, *p*-value = 0.030, *n* = 6). In the reported disease subgroup, we only detected a small reduction in WC (SDM: −0.57, *p*-value = 0.065; DM: −0.71 cm, *p*-value = 0.029) and an increase in FMD (SDM: +0.66, *p*-value = 0.037). Subgroups comparison also highlighted that the ET-containing products were more effective at reducing glucose in healthy volunteers than in participants with some disease ((SDM): Q statistic = 5.99, *p*-value = 0.014 and (DM): Q statistic = 3.91, *p*-value = 0.048). All these results were based on a very small number of studies and should be taken with caution.

Regarding the ANC-containing products, the most clear and consistent evidence was that supplementation with these products significantly reduced blood pressure independent of the health status of the participants and thus, they similarly lowered SBP and DBP in healthy non-medicated individuals, in participants ‘at risk’ (also non medicated), as well as in those patients with some diagnosed cardiovascular disease and under medication (Supplementary Table S9b). Also, the ANCs significanlty reduced WC only in the subgroup of ‘at risk’ participants (SDM: −0.24, *p*-value = 0.017; DM: −1.72 cm, *p*-value = 0.064, *n* = 7) whereas T-C was downregulated in the healthy subgroup (DM: −0.15 mmol/L, *p*-value = 0.003, *n* = 32) and in the disease subgroup (SDM: −0.24, *p*-value = 0.042, *n* = 36).

We did not find significant evidence in any particular subgroup (healthy, ‘at risk’, disease) in which the supplementation with ANC-containing products caused a change in BMI, LDL-C, HDL-C, TAGs, glucose, insulin, and HOMA-IR. Instead, we detected a significant increase in FMD in the healthy subgroup (DM: +0.92%, *p*-value = 0.049, *n* = 10).

#### 2.4.3. Stratification by the Country Where the Study Was Carried Out

In the absence of proper characterization of the ethnicity of the participants (most studies did not report this information), we explored the potential influence of the country of recruitment or country where the study was carried out. Once more and despite the reduction of the number of studies per subgroup and the limited significance of most results, we were able to find some significant data with sufficient number of studies for some of the outcomes examined (Supplementary Table S10a–c). 

There were very few studies (*n* = 1–4) carried out with ET-containing pomegranate or nuts in East Asian countries. In this subgroup, we only found a significant reduction of T-C (DM: −0.19 mmol/L, *p*-value = 0.050, *n* = 4). In the subgroup constituted by all-the-other-countries except the East Asian ones, the reduction of T-C was also significant, as well as that of WC, LDL-C, TAGs and glucose (Supplementary Table S10a). The subgroup of studies carried out in North America (Supplementary Table S10b) also gave some significant evidence of the reduction of WC, T-C, LDL-C, TAGs, and of insulin levels even though the number of studies included was not very big (*n* = 4–11). Subgroup comparions between studies conducted in North America and those carried out in Europe detected a significant difference in the effects of these products on insuline which was increased in the European studies ((DM): Q statistic = 5.79, *p*-value = 0.016). In addition, a small but significant increase in FMD was seen in the North American countries but only with two studies included. Overall, the studies conducted in European countries with ET-containing products show limited evidence of the reduction of T-C, LDL-C, glucose and insulin. Of note, the DBP was reduced by this type of product in European countries (DM: −5.21 mm Hg, *p*-value = 0.048, *n* = 5). Further stratification into Mediterraean or non-Mediterranean European countries (Supplementary Table S10c) reduced the number of studies per subgroup to 1 or 2 for most of the biomarkers investigated and the results were mostly not significant. Nevertheless, in the Non-Mediterranean countries there was a tendency towards the reduction of T-C, LDL-C, DBP, and glucose by the intake of ET-products. Insulin was significantly augmented in this subgroup (DM: +3.04 mIU/mL, *p*-value = 0.027) in the limited studies reported (*n* = 2).

In the all-other-countries-but-not-East-Asian subgroup, the ANC-containing products had no apparent effect on LDL-C, HDL-C or FMD whereas in the studies conducted only in East Asian countries, the ANCs significantly reduced LDL-C (SDM: −0.45, *p*-value = 0.003, DM: −0.30 mmol/L, *p*-value = 0.000, *n* = 6), and increased HDL-C (SDM: +0.57, *p*-value = 0.000, DM: +0.15 mmol/L, *p*-value = 0.000, *n* = 6) and FMD (DM: +2.19%, *p*-value = 0.000, *n* = 2) (Supplementary Table S10a). Subgroup comparisons between studies conducted in East Asian countries and those carried out in the rest of the world confirmed a significant effectiveness in the upregulatory effects of the ANC-containing products in the Asian countries for HDL-C ((SDM): Q statistic = 5.21, *p*-value = 0.022; (DM): Q statistic = 5.51, *p*-value = 0.019) and for FMD ((SDM): Q statistic = 4.09, *p*-value = 0.043; (DM): Q statistic = 4.28, *p*-value = 0.038). For T-C and DBP, the evidence of a reduction by the ANC-containing products was stronger in the all-other-countries subgroup than in the East Asian ones. These results give some preliminary evidence of a potential influence of the East Asian countries associated characteristics on the effect of the ANC-containing products. On the other hand, the effects on SBP remained significant in both subgroups reinforcing a general effectivity of these type of compounds at regulating blood pressure. In support of this statement, when we classified the studies into those carried out in North America and those conducted in Europe (Supplementary Table S10b), the ANCs still significantly reduced SBP and DBP in both subgroups. We additionally detected a significant reduction of T-C only in the European countries (SDM: −0.18, *p*-value = 0.017, *n* = 33; DM: −0.17 mmol/L, *p*-value = 0.000, *n* = 31) and some differences between the two subgroups at regulating the levels of insulin, with an apparent increase in North America studies and a reduction in European studies. Finally, stratification of the European countries into the Mediterranean and the Non-Mediterranean ones also shows some differences between the two areas, i.e., a significant reduction of LDL-C by the ANCs only in the Mediterranean countries (SDM: −0.35, *p*-value = 0.002, *n* = 14; DM: −0.19 mmol/L, *p*-value = 0.003, *n* = 14) or a significant increase in FMD only in the Non-Mediterranean area (SDM: +0.54, *p*-value = 0.006, *n* = 13; DM: +1.21%, *p*-value = 0.002, *n* = 13) (Supplementary Table S10c). SBP and DBP were downregulated in both areas of Europe.

#### 2.4.4. Stratification by Specific Sources of ETs and ANCs.

Examination for specific differences between the main sources of compounds was investigated: pomegranate and nuts for ETs, and berries and red grapes/wine for ANCs. The complete results of this analysis can be seen in Supplementary Table S11. A summary with the most significant results and differences are listed in Table 4.

Comparison of studies by sources of ETs into pomegranate and nuts (Table 4a) demonstrated that nuts reduced significantly WC, T-C, LDL-C and TAGs. They also showed a tendency to reduce glucose levels. Further, nuts had a small but significant increase in HDL-C and a marginally significant increase in FMD. None of these effects were seen in the group of studies carried out with pomegranate. In addition, a very significant difference in the regulation of DBP was detected between these two types of products with studies conducted with pomegranate reporting that DBP was significantly reduced whereas nuts reported a small but significant increase in DBP (subgroups comparison (SDM): Q statistic = 12.95, *p*-value < 0.001; (DM): Q statistic = 17.32, *p*-value < 0.001).

Both sources of ANCs, berries and red wine/grapes, caused a significant reduction in blood pressure (Table 4b) but some differences were also detected between the two types of products. The berries reduced T-C and increased FMD whereas grapes and wine did not. In addition, the glycated hemoglobin was significantly reduced by the berries as opposed to the grapes/wine which increased the values of this biomarker (subgroups comparison (SDM): Q statistic = 8.59, *p*-value = 0.003; (DM): Q statistic = 9.41, *p*-value = 0.002). 

## 3. Discussion

As far as the authors are aware, this is the largest (128 reported dietary intervention studies involving 5438 participants from countries covering five continents) systematic review and subsequent meta-analysis investigating and comparing the effects of the consumption of plant food products and derived extracts containing substantial quantities of ANCs and (or) ETs, i.e., berries, red grapes and wine, pomegranate and nuts, on a number of well-established risk biomarkers associated with cardiometabolic disease. We have analyzed all these trials in an effort to determine the evidence accumulated so far in relation with their potential cardiometabolic benefits in humans, as well as the factors that cause variability in the results and may influence the response of the individuals to the consumption of these products. In the first part of our analysis, it was pertinent to investigate all these foods and food products together because some of them can contain high concentrations of both types of polyphenols, notably various berries [161,162]. This approach provides a most significant association between the intake of ANC- and (or) ET-containing products and beneficial changes in WC, T-C, SBP and DBP (reductions compared to control), or HDL-C (increase compared to control). Further, there were modest borderline significant reductions in fasting plasma glucose and TAGs and an increase in FMD. On the other hand, our analyses of data from studies separately investigating the effects of ET-rich foods (pomegranate and nuts) or ANC-rich foods and extracts (berries, red wine, red/black grapes) confirms effective and similar reduction of T-C levels by both types of products but points out differences between the beneficial effects of pomegranate and nuts (more efficacious at reducing WC, LDL-C, TAGs or glucose) and the benefits berries/grapes/wine (significant effective regulators of blood pressure). It is important to acknowledge that the beneficial effects detected by our analysis cannot be exclusively attributed to the ANCs or ETs present in them and that we cannot discard these effects may be also attributed to other components. Nevertheless, these results support the benefits of consuming food products containing ANCs and (or) ETs that, at least partially, may be due to these polyphenols.

With regards to the magnitude of the effects, it has been previously stated that a reduction of 1 mmol/L for non-HDL-C and an increase of 0.3–0.4 mmol/L for HDL-C are associated each with a one third reduction in ischemic heart disease risk [163] or a 22% reduction in coronary heart disease risk [164]. It has also been reported that a reduction of 12 mm Hg for SBP and of 5 mm Hg for DBP are accompanied by significant reductions of major cardiovascular events [165]. The results of our meta-analysis (expressed as DM values) show that, on average, the intake of ANC- and ET-containing products is associated with approximately 10-fold lower effects, i.e., a reduction of 0.10 mmol/L in T-C, an increase of 0.03 mmol/L in HDL-C and a decrease of 1.5–2.0 mm Hg for blood pressure, both SBP and DBP. These changes constitute between 1% and 3% change of the desirable threshold values for these biomarkers [164,166,167] and might be comparatively considered small or very small changes. This is also shown by the SDM values, mostly in the range of 0.1 to 0.2, generally considered as small changes [168]. Nevertheless, it is recognized that conventional risk factors interact to increase the risk for cardiometabolic diseases [169] and that combined treatment may be advantageous for the lowering of cardiometabolic events [170]. The fact that multiple vascular biomarkers that reflect multiple components of the cardiometabolic system are significantly altered in response to the consumption of ANC- and ET-rich foods and food products may contribute to the reduction of major cardiovascular events. In support of this, a previous meta-analysis of three prospective cohort studies concluded that ANCs intake was inversely associated with the risk of cardiovascular disease comparing the highest and lowest categories of intake [171]. Together, all these data suggest that foods and food products containing ANCs and ETs may act via the regulation of multiple biomarkers, including lipids, blood pressure and glucose homeostasis/insulin resistance.

The second aim of this systematic review and meta-analysis was to investigate the potential associations between some participant variables (baseline BMI, age, gender, smoking, geographical location where the study was carried out, health status and nature of the diet followed during the intervention) and the effects of the intervention with the food products containing ETs and (or) ANCs. We show, for first time, that the significant effects of ET-rich products on T-C, LDL, TAGS and DBP and of ANC-rich food products on T-C, DBP and SBP were consistently observed in participants with BMIs ≥ 25 kg/m^2^ (overweight/obese). Similar results were found in a previous meta-analysis, looking at the effects of flavanol-containing products in T-C [29]. Together, these results suggest that supplementation with polyphenol-rich products may have a beneficial impact on some cardiometabolic risk factors in overweight and/or obese people. In addition, significant effects of ET-rich products on SBP were observed in overweight/obese subjects but no significant effects on SBP were observed in the global study population, again supporting the need for population stratification, within such intervention studies, in order to discern effective regulation of biomarkers by the intake of bioactive compounds or products. It should be noted, however, that we found limited or non-evidence in normoweight participants since the number of studies reported was very small (*n* = 3). Thus, further investigation of the effects of ANC- and ET-products, and of other bioactive compounds, in individuals with BMI < 25 kg/m^2^ are needed. With regard to the influence of participant sex, smoking habits, health status and habitual diet, there were, in general, a very small number of trials that provided this information in a useable form, limiting our ability to investigate how these host factors might affect the responses to intervention with foods and food products containing ANCs and/or ETs. Nevertheless, in the subsequent paragraphs we discuss some of the most relevant effects found in this meta-analysis for some of these factors.

Different responses were observed depending on the biomarkers analyzed and the health status of the participants. Accordingly, the beneficial effects of ANC-rich foods and extracts on blood pressure appeared to be independent of the participant health status, whereas we found some differences between healthy, ‘at risk’ and diseased participants in the effects of these foods on WC and T-C. On the other hand, supplementation with the ET-containing products modified WC, T-C and DBP only in healthy people, but affected FMD, LDL and TAGs in the ‘at risk’ and disease subpopulations. Previous reviews and meta-analyses had already indicated differences in the response to the intake of these and other bioactive compounds depending on the health status of the sample population. For example, systematic reviews of the effects of interventions with ANCs on lipid biomarkers have reported that only ANCs cause significant reductions in LDL-C in participants with hypercholesterolemia (4 out of 4 studies), but not in participants who had normal cholesterol (zero out of 8 studies) [172]. It was also reported that ANCs supplementation significantly decreased T-C, TAGs, LDL-C and increased HDL-C in dyslipidemic patients (6 studies including 586 subjects [173]. Similarly, it has been reported that high-dose quercetin supplementation caused a significant reduction in blood pressure in stage 1 hypertensive participants but was not affected in pre-hypertensive participants [174]. Also, the consumption of green tea [175,176], black tea [177], and flavanol-containing tea, cocoa and apple products [29] had beneficial effects on blood lipids both in healthy subjects and in patients with hyperlipidaemia or in individuals with cardiovascular risk and/or diagnosed diseases. On the contrary, meta-analyses conducted with flavonols [28], cocoa products [178], and soy products [179] reported beneficial effects in LDL-C, HDL-C and TAGs in participants with cardiovascular risk and/or diagnosed diseases, but no effect on healthy participants. Overall, these results show some evidence of the influence of the health status on the cardiometabolic response to the intake of bioactive compounds but this interaction is complex and far from understood. It is essential to continue the research to clarify the impact of the health/disease baseline conditions of the participants on the response to bioactive compounds intake.

Previous studies have indicated that the country or area of the world where the clinical study was conducted appears to have some influence on the results of these types of interventions with plant bioactive polyphenols. For example, specific differences between East Asian and Non-Asian countries or between Mediterranean and Non-Mediterranean countries for some lipid biomarkers in response to the intake of flavonol-containing products [28] or flavanol-containing products [29] have been reported. In the current meta-analysis, we have also identified a few differences in the effects of the ET- and ANC-containing foods for some of the biomarkers examined between specific countries or geographical locations. In the case of products with ANCs, a significant reduction of LDL-C was seen only in East-Asian countries, but not in the rest of other countries; whereas the products containing ETs were efficient at reducing LDL-C in the subgroup of all the countries, but not in the East-Asian ones. The rationale for these potential differences and as to why a particular country or world area population may benefit best from the intake of these or other bioactive compounds is not yet known. In addition to the fact that the number of studies per subgroup of countries remains low and the results are very unstable, important country-associated features, such as the ethnicity of the participants, which may influence greatly the results have not been clearly indicated in most of the revised publications included in this meta-analysis and in previous ones [28,29]. Future studies should better describe these country-related characteristics of the participants.

Although the source of bioactive compounds cannot be categorized as a host determinant of interindividual variability, it has been already shown that it can significantly affect the response to the intake of these compounds [33]. In our meta-analysis we also stratifiyed the results taking into account the main categories of foods investigated, i.e., pomegranate, nuts, berries and red grape/wine. Our results highlighted differences in the effects between pomegranate and nuts. The pomegranate products significantly reduced DBP, but had no apparent effect on SBP, whereas nuts were found to cause a small but significant increase in DBP and had no significant effect on the SBP either. Also, nuts were associated with significant reductions in WC, T-C, LDL-C and TAGs, as well as a borderline increase in FMD, but these effects were not observed for the pomegranate. In comparison with our results, we found both agreement and disagreement with previous meta-analyses. For example, Sahebkar and colleagues reported significant reductions in SBP and DBP caused by the consumption of pomegranate products [24], but no significant effects on plasma lipids/lipoproteins [25]. Mohammadifard et al. [22] saw significant reductions in SBP only in participants without diabetes and significant reductions in DBP (but not SBP) in response to all nut supplementation and suggested that this was largely due to pistachios. Also, the effects of tree nuts were shown to reduce T-C, LDL-Cand TAGs [23]. Regarding the ANC-containing products, a previous meta-analysis looking at the supplementation with blueberries and their effect on blood pressure concluded that the results were not convincing and that more RCTs were needed [19]. In the current meta-analysis, ANC-containing products have come out as quite consistent regulators of SBP and DBP. Separation between studies conducted with berries and those carried out with red grapes/wine confirmed that both sources of ANCs significantly lowered SBP and DBP and supported that this type of bioactive compounds might have an impact on blood pressure regulation. We additionally found that the berries but not the grapes/wine significantly reduced T-C and increased FMD. In agreement with this last result, a recent meta-analysis on 24 RCTs showed that both acute and chronic supplementation with ANC-rich foods or extracts significantly improved FMD and improved wave velocity after acute consumption [21].

Various factors might be implicated in the differences found between the different types of food investigated. Most of the studies gathered in our meta-analysis, as well as in previous ones, have been conducted with various whole foods or derived extracts which are complex mixtures of compounds and thus, we cannot discard that the differences observed may be caused by differences in the types and doses of ETs or ANCs provided by these products and (or) by differences in the presence of other bioactive components with a beneficial effect. For example, there is a substantial literature describing the beneficial effects of nuts on biomarkers of cardiovascular health and on cardiovascular events see reviews by Schwingschackl et al., 2017 [180] and by Mayhew et al., 2016 [181], but it is not yet known whether these benefits may be due to the fatty acids/lipids, the polyphenols (which are mainly in the skins) or a combination of the two. Additional evidence from further well-designed trials are required before we can unequivocally attribute the benefits to the specific ETs or ANCs present in these products.

In addition to the factors examined in this meta-analysis, other important factors that may play a critical role in the inter-individual variability and can contribute to explain the lack of consistent evidence in humans of the beneficial cardiometabolic effects of the ANC- and ET-containing food products (as well as of other polyphenol-containing foods and extracts) are: bioavailability of all these compounds and their derived metabolites [182,183], individuals enterotypes and functional stratification of the gut microbiome profile (i.e., metabotypes) [31,184,185], and the (epi)genetic characteristics of the host individual.

Regarding microbiota enterotypes, it is important to note that ETs are hydrolyzed into ellagic acid (EA) and further broken down into urolithin metabolites by the gut bacteria. Urolithins are much better absorbed and reach significant concentrations in plasma that can persist for hours in the human body after the intake of ETs-containing products suggesting that these urolithins may be the actual bioactive molecules [186]. Recently, it was reported that three urolithin metabotypes, based on the qualitative and quantitative proportions of urolithins produced, were consistently observed across multiple intervention studies and appeared to be independent of the ET food source and age or health status of participants [184]. These observations create a new paradigm where the urolithin metabotype of the participants should be determined and included as a covariate in future studies investigating the effects of consuming ET-rich products such as pomegranate and nuts. Along these lines, a recent RCT has shown that intervention with a purified pomegranate extract containing mainly ETs significantly improved lipid/lipoprotein profiles only in participants who produced a particular type of urolithins (around 30% of the total sample population), whereas no significant effects were detected when all participants were included in the analysis [31]. Therefore, since the urolithin metabotype of participants has not been reported or used to stratify participants for the vast majority of studies investigating the effects of ET-rich foods that were included in the current meta-analysis, it is not surprising that the meta-analysis failed to detect significant effects of ETs on lipoprotein profiles. Similar cases have been reported for the conversion of the soy isoflavone daidzein into equol, where volunteers can be categorized into equol producers and non-producers and this stratification might explain the discrepancy of the soy/isoflavones effects on human health, mainly cardiovascular. Thus, obesity has been correlated with the non equol-producer phenotype [187]. In addition, a positive correlation has been observed in the cardiovascular risk profiles and the equol-producer phenotype in pre-hypertensive postmenopausal women [188].

On the other hand, the relevance of the genotype-dependent response to dietary constituents is recognized as an additional key variability factor [189]. In this regard, an increasing number of genetic variants has been identified and related to obesity and diet-interaction and, the studied population has been segregated into groups of responders and non-responders in association with the specific genetic variations [190]. A number of studies have now also looked at the role of specific host genetic variants in response to the consumption of polyphenols or polyphenol-containing products, including genetic polymorphisms involved in: (i) the metabolism and transport of polyphenols such as Catechol-*O*-methyl transferase [191] or phase II enzymes UGT1A1 [192] and (ii) the cardiometabolic responses such as the lipid and blood pressure variation associated with the apolipoprotein e genotype in response to quercetin in overweight people [193] or the interaction between the *IL-6*-174 G/C polymorphism and the reduction of body fat following the intake of a polyphenol-rich apple juice [194]. More genes and polymorphisms involved in the response to polyphenols need to be identified and more RCTs need to be performed reporting and associating the presence of those relevant genetic polymorphisms, as well the microbiota composition and microbiota-derived metabolic phenotype with the differences in the response of the individuals to the consumption plant food bioactive compounds. These studies will contribute to better understanding of the effectiveness of these compounds in different subpopulations.

Some of the limitations of our meta-analysis are those inherent to this type of analysis. Given the heterogeneity of the RCTs included, the size of the sub-groups and the effect size detected for the variables investigated, some of the results presented here should be regarded with caution, even if some *p*-values resulted significant. A critical issue to consider would be the level of statistical significance accepted for the meta-analysis. We have accepted *p*-value < 0.05 as significant and indicated also some results that were marginally significant (0.1> *p*-value > 0.05) since we thought that they could be indicative of an effect (that, of course, would need future confirmation). Some researchers may believe that more restrictive *p*-values <0.01 or <0.001 should be applied [195] whereas, more recently, estimation based on effect size and confidence intervals is recommended [196]. In any case, the interpretation of the results of the meta-analysis may vary, especially for those most unstable results. Equally important is to address and understand the clinical relevance of the effects. We have addressed this in our discussion and suggested that, in general, the size of the effects of the dietary interventions with foods and (or) food products containing ANCs or ETs (or other polyphenols) may be considered small. Future RCTs should be sufficiently powered to validate these small changes. We additionally detected some publication bias for some of the variables investigated in this review. It is important that future publications of this kind of interventions also report the less favourable or negative results.

In conclusion, and despite these limitations, this is one of the largest meta-analysis performed in the area of the beneficial effects of the consumption of plant polyphenols in humans, and provides a good summary of the available information on the cardiometabolic effects of the intake of foods and food products containing ANCs and ETs. Overall, these foods and products appear to promote small but beneficial regulatory changes on a combination of risk factors and may contribute to prevent cardiometabolic diseases. Nevertheless, from a nutritional practice point of view, it is not yet possible to establish specific intake recommendations for these foods and (or) for the ANCs and ETs present in them since there are still some important challenges to solve. One of those is that there are still very few trials conducted with specific doses of purified ANCs or ETs compared with nutritionally matched placebos to demonstrate unequivocally the effects of these compounds and the doses needed. More RCTs designed for this purpose should be done in the future. Another important issue is that responses to polyphenol dietary interventions can be significantly dependent on different host factors and that within a study population there are subgroups of participants that respond strongly to a polyphenol intervention while other participants respond weakly or not at all. Our meta-analysis has explored some of the factors that might affect the response to the intake of ANC- or ET-containing products (baseline BMI, health status or food source) but our results were not sufficient to draw definitive conclusions in the subgroup analyses. Research to establish the determinants that cause inter-individual variability of the responses to the consumption of these and other bioactive compounds is a high current priority [33]. Ideally, better study design providing detailed descriptions, particularly around the choice and numbers of participants, should be addressed so that significant and clinically relevant effects on a primary outcome can be established for each subpopulation investigated with the ultimate goal of developing personalized nutrition strategies for human health and disease prevention. Such studies are still rare, and it is instead typical that stratifying of participants is done as an afterthought and was not considered in setting the participants’ numbers at the stage calculating study power. An alternative approach would be to use data reported from completed and future trials to determine what factors affect responses to polyphenols. The ideal scenario would be the reporting of individual level data, notwithstanding the complex ethical and regulatory issues that this would raise. If all outcome responses were available for each participant along with pertinent participant characteristics such as age, gender, BMI, ethnicity, health status, habitual diet, smoking habit, baseline values for a series of risk biomarkers, relevant host genetic makeup and metabolic phenotype, this would allow studies to determine relationships between participant characteristics and their propensity to respond to polyphenols to advance rapidly. One could envisage an online repository for such information being of tremendous value and supporting high quality research that would relatively rapidly allow individual characteristics that determine responses to be identified. However, the ethical and regulatory issues are not trivial, and are not even consistent between territories, and such a data repository is not currently available.

## 4. Materials and Methods

A meta-analysis was performed to explore the potential regulatory effects of foods and (or) products containing ellagitannins (ETs) and/or anthocyanins (ACNs). We used the preferred reporting items and statement guidelines for systematic review and meta-analysis protocols (PRISMA, Preferred Reporting Items for Systematic Reviews and Meta-Analyses) [197], the Cochrane Handbook for Systematic Reviews of Interventions [198], and the Centre for Reviews and Dissemination’s guidance for undertaking reviews in health care [199]. The protocol for this review was registered on the International Prospective Register of Systematic Reviews (PROSPERO) [200] with the registration number CRD42016037539.

### 4.1. Search Strategy and Study Selection

A comprehensive search on Medline [201] and on the Web of Knowledge [202] databases was conducted between April and June 2016. The search strategy included a combination of the following search terms: #1 AND #2 AND #3 AND #4 AND #5 (#1 polyphenol* OR ellagitannin* OR urolithin* OR hydrolizable tannin OR “ellagic acid” OR punicalagin OR peduncalagin OR sanguiin OR anthocyanin* OR anthocyanidin* OR pelargonidin OR delphinidin OR cyanidin OR petunidin OR peonidin OR malvidin; #2 berry OR berries OR “black currant” OR nuts OR walnut* OR “black carrot” OR “purple corn” OR pomegranate OR aronia OR wine OR grape*; #3 trial OR experiment OR study OR studies OR intervention; #4 human* OR subject* OR men OR male OR women OR female OR patient* OR volunteer* OR participant*; #5 FMD OR “flow-mediated dilation” OR “flow-mediated vasodilation” OR “flow-mediated vasodilatation” OR “endothelial function” OR “endothelial dysfunction” OR “blood pressure” OR hypertens* OR “arterial pressure” OR “pulse pressure” OR cholesterol OR LDL OR HDL OR BMI OR “body mass index” OR waist* OR HOMA-IR OR HOMA2 OR “homeostatic model assessment” OR insulin* OR QUICKI OR “impaired sensitivity” OR “Syndrome X” OR “Metabolic Syndrome X” OR glucose OR “blood glucose” OR glycemia OR “glycemic control” OR HbA1c OR “glycosylated haemoglobin” OR “glycated haemoglobin” or “haemoglobin A1c” OR “hemoglobin A, glycosylated” OR “euglycemic clamp” OR dyslipidemia* OR hyperlipidemia* OR hypertriglyceridemia* OR triglyceride* OR triacylglycer*).

The search terms were queried using the “topic” field in the WOS database; whereas for PubMed search we used the corresponding Mesh Terms, when available, and the presence of the keywords in the title or abstract of the papers using the tag [TIAB].

Two authors independently assessed all papers and a third author double-checked data selection to reach a consensus with the final selected studies. Studies included in the meta-analysis were limited to human RCTs testing the effect of ET- or ANC-containing foods or products, which had a control group receiving a placebo (group of participants who were exposed to a similar test product but without the ETs or ANCs) and measured one or more of the defined outcomes. Additional exclusion criteria were: case series, case reports, cohort studies, case-control studies, co-intervention, and cross-sectional studies, studies with multifactorial interventions (dietary or physical activity co-intervention), studies written in a non-European language and duplications.

### 4.2. Data Extraction

Data extraction was performed in duplicate by two authors, independently, and cross-checked by a third author using a standardized data extraction form. Extracted data included: (i) publication details (year of publication, name of first author, name and e-mail of corresponding author, clinical trial registration number (when available), country where the study was carried out); (ii) participants’ characteristics (gender, age, ethnicity, health status, menopausal status, smoking habits, baseline BMI, use of medication); (iii) study setting (total number of participants included in the study and in the analysis, design (cross-over or parallel), washout duration, treatment duration, number of arms and description, number of participants located in each arm and completing the study, composition of test and placebo, dose and mode of administration); and (iv) information on reported outcomes (type of sample, changes in the outcome, values before and after intervention, *p*-value when available, dropouts). Before analysis, outcomes on blood lipid levels and glucose levels were converted to mmol/L if reported in a different unit.

### 4.3. Assessment of the Risk of Bias

A systematic assessment of the risk of bias for each of the included studies was based on the Cochrane Collaboration measurement with some modifications [198]. The specific items used for the assessments of each study are those used in a previous meta-analysis [28]: (1) selection bias—random sequence generation, allocation concealment (in each item, yes = 1; no = 0, unclear = 0); (2) performance bias—blinding (yes = 1 for each participants, researchers and statisticians, no = 0, unclear = 0), measurement of compliance (1 for biomarker measure, 0.5 if compliance information was collected by counting non used capsules or recipients, or by self-reporting, 0 if no measurement of compliance was done or the information is insufficient); (3) attrition bias – flow of participants (1 if flow of participants is explained in detail, including number of withdrawals and reasons, 0 if there is no information or insufficient information); (4) other bias—baseline comparability between test and control groups (yes = 1, no = 0, unclear = 0), data report (1 if pre and post data or change is reported in table with central measure and spread for placebo and treatment groups, and number per group, 0 if anything is missing), industry funding (0 if any commercial source provided some or all monetary funding for the trial, if a company carried out a study “in house”, if any of the authors was employed by a relevant industry or if it was unclear that there was any kind of industry funding, 1 if there was no funding from industry or if the only involvement of a company was to provide any ingredient for the intervention). Studies were rated as low risk of bias when total score was ≥8 and ≤10, moderate risk of bias when total score was ≥5 and <8 and high risk of bias when total score was below 5.

### 4.4. Data Analysis

Data for each outcome were analyzed using the Comprehensive Meta-Analysis Software, version 3.0 (Biostat, Englewood, NJ, USA) [203]. Standardized difference in means (SDM), standard error (SE) and the 95% confidence intervals (CI) were calculated and pooled using random effects models to determine test/placebo differences across studies. We additionally determined absolute difference in means (DM) to estimate effect size. The heterogeneity of studies was assessed using the Cochran’s Q statistic, the between-studies variance (*T*^2^) and *I*^2^ (the proportion of total variation contributed by between-study variability) where *I*^2^ values equal to 25%, 50% and 75% were considered as low, moderate and high heterogeneity, respectively [204]. Publication bias was assessed visually with funnel plots and statistically by applying the Egger’s regression test [205]. Further assessment of the possible associations between the overall effects of the ETs and/or ANCs supplementation and the duration of the intervention was examined using random-effects meta-regression analysis.

Quality of evidence was assessed based on the GRADE system [198]. Level of evidence was downgraded from high to moderate in the presence of serious risk of bias across studies or serious risk of reporting bias, and downgraded to low if both were present.

Subgroup analyses were conducted to explore potential factors that may introduce heterogeneity into the studies and influence the inter-individual variability in the response to supplementation with the ET- and/or ANC-containing products. We selected those factors that were investigated previously [28,29] and were most clearly reported in the selected articles (Table 5). Briefly, we included factors that might be attributed to some of the individuals’ characteristics, such as baseline BMI, sex, smoking habits and medication/health status. Age or ethnicity could not be assessed due to unclear reporting. We also included stratification by the country in which the study was carried out, the source and form of administration of the ETs and/or ANCs, as well as the type of diet reported to be followed during the intervention. For each subgroup, the pooled effects (SDM and DM) and the significance of these values were estimated. Statistical comparisons between subgroups were performed by applying a random-effects analysis and calculation of the between-categories Q statistic and the corresponding *p*-values. A *p*-value <0.05 was statistically significant. Differences with a *p*-value <0.1 and ≥0.05 were reported as marginal.

## Figures and Tables

**Table 1:** Summary of the most significant global effects of foods and food products containing ETs and ANCs on biomarkers of cardiometabolic risk.

	SDM (*p*-Value)	95% CI	*n*	N_T_	*I*^2^ (%)	GRADE ^1^		DM (*p*-Value)	95% CI	*n*	N_T_	*I*^2^ (%)	GRADE
WC	−0.30 (0.008)	(−0.52, −0.08)	23	1023	63.4	Moderate ^2^	WC (cm)	−1.22 (0.005)	(−2.07, −0.36)	22	972	37.5	Moderate ^2^
T-C	−0.17 (0.001)	(−0.27, −0.07)	109	3991	54.5	Moderate ^2^	T-C (mmol/L)	−0.10 (0.013)	(−0.18, −0.02)	103	3673	70.6	Moderate ^2^
SBP	−0.20 (0.000)	(−0.28, −0.12)	95	3539	25.0	High	SBP (mm Hg)	−1.56 (0.000)	(−2.13, −0.99)	83	3175	0.00	High
DBP	−0.19 (0.000)	(−0.26, −0.11)	99	3790	27.9	High	DBP (mm Hg)	−1.42 (0.000)	(−2.08, −0.76)	90	3473	41.6	Moderate ^2^
HDL-C	+0.11 (0.034)	(0.01, 0.21)	99	3581	53.1	Low ^2,3^	HDL-C (mmol/L)	+0.03 (0.062)	(0.00, 0.05)	92	3239	61.6	Moderate ^2^
FMD	+0.20 (NS)	(−0.17, 0.57)	22	563	73.8	Low ^2,3^	FMD (%)	+0.64 (0.027)	(0.07, 1.20)	21	547	82.2	Low ^2,3^

Significant: *p*-value 0.05; Marginally Significant (0.05 ≤ *p*-value 0.1). SDM: standardized difference in means; DM: Difference in means; CI: confidence intervals; *n*: total number of studies; N_T_: total number of participants; *I*^2^: Heterogeneity Index; WC: Waist Circumference; T-C: Total Cholesterol; HDL-C: High Density Lipoprotein Cholesterol; SBP: Systolic Blood Pressure; DBP: Diastolic Blood Pressure; FMD: Flow Mediated Dilation. ^1^ GRADE quality of evidence downgraded from high to moderate or low in the presence of serious risk of bias across studies and (or) serious risk of reporting bias; ^2^ serious risk of bias across studies: more than 50% of the studies had unclear allocation concealment, no double-blind studies and unclear reporting of dropouts; ^3^ serious risk of reporting bias: Eggert *p*-value 0.05 and more than 50% of small studies with limited number of participants.

**Table 2:** Summary of the most significant effects of the foods and food products containing ETs (pomegranate and nuts) and those containing ANCs (berries, red grapes and red wine) on biomarkers of cardiometabolic risk.

	ET-Containing Products (Pomegranate, Nuts)						ANC-Containing Products (Berries, Red Grapes, Red Wine)				
	SDM (*p*-Value)	*n*		DM (*p*-Value)	*n*		SDM (*p*-Value)	*n*		DM (*p*-Value)	*n*
WC	−0.70 (0.025)	7	WC (cm)	−1.53 (0.031)	6	WC	−0.12 (NS)	16	WC (cm)	−0.75 (NS)	16
T-C	−0.18 (0.006)	28	T-C (mmol/L)	−0.09 (0.000)	26	T-C	−0.17 (0.008)	81	T-C (mmol/L)	−0.10 (0.094)	77
HDL-C	+0.10 (NS)	23	HDL-C (mmol/L)	+0.03 (NS)	21	HDL-C	+0.12 (NS)	76	HDL-C (mmol/L)	+0.03 (NS)	71
LDL-C	−0.19 (0.031)	26	LDL-C (mmol/L)	−0.11 (0.000)	24	LDL-C	0.05 (NS)	71	LDL-C (mmol/L)	−0.03 (NS)	68
TAGs	−0.24 (0.025)	26	TAGS (mmol/L)	−0.11 (0.000)	24	TAGs	+0.004 (NS)	71	TAGS (mmol/L)	+0.02 (NS)	64
SBP	−0.11 (NS)	21	SBP (mm Hg)	−1.89 (NS)	15	SBP	−0.23 (0.000)	74	SBP (mm Hg)	−2.19 (0.000)	68
DBP	−0.14 (NS)	20	DBP (mm Hg)	−1.28 (NS)	18	DBP	−0.20 (0.000)	79	DBP (mm Hg)	−1.58 (0.000)	72
FMD	+0.62 (0.014)	3	FMD (%)	+0.39 (NS)	3	FMD	+0.12 (NS)	19	FMD (%)	+0.53 (NS)	18
Glucose	−0.24 (0.052)	16	Glucose (mmol/L)	−0.12 (0.01)	15	Glucose	−0.05 (NS)	22	Glucose (mmol/L)	+0.001 (NS)	45

Significant: *p*-value 0.05; Marginally Significant (0.05 ≤ *p*-value 0.1). WC: Waist Circumference; T-C: Total Cholesterol; LDL-C: Low Density Lipoprotein Cholesterol; HDL-C: High Density Lipoprotein Cholesterol; TAGs: Triglycerides; SBP: Systolic Blood Pressure; DBP: Diastolic Blood Pressure; FMD: Flow Mediated Dilation; *n*: total number of studies included in the analysis; SDM: standardized difference in means; DM: Difference in means.

**Table 3:** Summary of the most significant effects of the foods and food products containing ETs (pomegranate and nuts) and those containing ANCs (berries, red grapes and red wine) on biomarkers of cardiometabolic risk in overweight and (or) obese individuals (baseline BMI: ≥ 25.0 Kg/m^2^).

BMI ≥ 25.0 kg/m^2^		ET-Containing Products (Pomegranate, Nuts)						ANC-Containing Products (Berries, Red Grapes, Red Wine)			
	SDM (*p*-Value)	*n*		DM (*p*-Value)	*n*		SDM (*p*-Value)	*n*		DM (*p*-Value)	*n*
WC	−0.70 (NS)	4	WC (cm)	−1.71 (0.047)	3	WC	−0.11 (NS)	10	WC (cm)	−0.70 (NS)	10
T-C	−0.12 (NS)	20	T-C (mmol/L)	−0.08 (0.000)	18	T-C	−0.34 (0.003)	35	T-C (mmol/L)	−0.23 (0.009)	35
LDL-C	−0.15 (NS)	20	LDL-C (mmol/L)	−0.11, (0.000)	18	LDL-C	−0.13 (NS)	30	LDL-C (mmol/L)	−0.11 (NS)	29
TAGs	−0.21 (NS)	19	TAGs (mmol/L)	−0.11 (0.000)	17	TAGs	−0.05 (NS)	28	TAGs (mmol/L)	−0.05 (NS)	27
SBP	−0.26 (0.012)	14	SBP (mm Hg)	−3.10 (0.033)	11	SBP	−0.25 (0.000)	43	SBP (mm Hg)	−1.54 (0.000)	38
DBP	−0.08 (NS)	15	DBP (mm Hg)	−0.55 (NS)	14	DBP	−0.22 (0.002)	30	DBP (mm Hg)	−1.62 (0.000)	29
FMD	+0.62 (0.014)	3	FMD (%)	+0.39 (NS)	3	FMD	−0.20 (NS)	6	FMD (%)	−0.65 (NS)	6
Glucose	−0.19 (0.058)	14	Glucose (mmol/L)	−0.18 (0.017)	13	Glucose	−0.13 (NS)	22	Glucose (mmol/L)	−0.02 (NS)	20

Significant: *p*-value 0.05; Marginally Significant (0.05 ≤ *p*-value 0.1). WC: Waist Circumference; T-C: Total Cholesterol; LDL-C: Low Density Lipoprotein Cholesterol; TAGs: Triglycerides; SBP: Systolic Blood Pressure; DBP: Diastolic Blood Pressure; FMD: Flow Mediated Dilation; *n*: total number of studies included in the analysis; SDM: standardized difference in means; DM: Difference in means.

**Table 4:** Comparative summary of the most significant effects on biomarkers of cardiometabolic risk of: (**a**) the ET-containing products after separation into the two main sources examined: pomegranate vs. nuts; (**b**) the ANC-containing products after stratification by the source of bioactive compounds: berries vs. red wine and red grapes.

**(a)**	**ET-Containing Products**								
**Source**	**Pomegranate**				**Nuts**				
	**SDM (*p*-Value)**	***n***	**DM (*p*-Value)**	***n***	**SDM (*p*-Value)**	***n***	**DM (*p*-Value)**	***n***	**Comparison between Subgroups (Q Statistic, *p*-Value)**
WC (cm)	−0.20 (NS)	1	−3.90 (NS)	1	−0.78 (0.027)	6	−1.51 (0.038)	5	SDM: 0.40, NS DM: 0.08, NS
T-C (mmol/L)	−0.04 (NS)	11	−0.02 (NS)	11	−0.32 (0.000)	17	−0.098 (0.000)	15	SDM: 10.83, 0.001 DM: 3.31, 0.069
LDL-C (mmol/L)	−0.07 (NS)	10	−0.05 (NS)	10	−0.26 (0.047)	16	−0.11 (0.000)	14	SDM: 1.31, NS DM: 0.66, NS
HDL-C (mmol/L)	+0.11 (NS)	10	+0.01 (NS)	10	+0.14 (NS)	13	+0.03 (0.029)	11	SDM: 0.09, NS DM: 0.30, NS
TAGs (mmol/L)	−0.05 (NS)	10	−0.01 (NS)	10	−0.33 (0.031)	16	−0.11 (0.000)	14	SDM: 1.44, NS DM: 1.10, NS
SBP (mm Hg)	−0.09 (NS)	8	−0.26 (NS)	6	−0.13 (NS)	13	−1.63 (NS)	9	SDM: 0.03, NS DM: 0.03, NS
DBP (mm Hg)	−0.46 (0.000)	8	−4.31 (0.000)	8	+0.06 (NS)	12	+0.58 (0.004)	10	SDM: 12.95, 0.000 DM: 17.32, 0.000
FMD (%)	+0.71 (NS)	1	+0.05 (NS)	1	+0.58 (0.058)	2	+1.04 (0.053)	2	SDM: 0.07, NS DM: 3.37, NS
Glucose (mmol/L)	−0.10 (NS)	7	−0.09 (NS)	7	−0.36 (0.079)	8	−0.14 (0.061)	8	SDM: 1.17, NS DM: 0.10, NS
**(b)**	**ANC-Containing Products**								
**Source**	**Berries**				**Red Wine/Red Grapes**				
	**SDM (*p*-Value)**	***n***	**DM (*p*-Value)**	***n***	**SDM (*p*-Value)**	***n***	**DM (*p*-Value)**	***n***	**Comparison between Subgroups (Q Statistic, *p*-Value)**
T-C (mmol/L)	−0.21 (0.021)	38	−0.16 (0.093)	35	−0.14 (NS)	44	−0.06 (NS)	43	SDM: 0.30, NS DM: 0.70, NS
SBP (mm Hg)	−0.25 (0.000)	38	−2.41 (0.000)	34	−0.21 (0.000)	36	−3.31 (0.014)	34	SDM: 0.11, NS DM: 0.35, NS
DBP (mm Hg)	−0.25 (0.001)	42	−1.57 (0.002)	37	−0.16 (0.000)	39	−1.50 (0.002)	35	SDM: 0.80, NS DM: 0.06, NS
FMD (%)	+0.46 (NS)	9	+1.39 (0.011)	8	−0.19 (NS)	10	−0.73 (NS)	10	SDM: 2.89, NS DM: 5.68, NS
Hb1Ac	−0.63 (0.044)	7	−0.20 (0.040)	6	+0.97 (0.038)	7	+0.26 (0.026)	7	SDM: 8.59, 0.003 DM: 9.41, 0.002

WC: Waist Circumference; T-C: Total Cholesterol; LDL-C: Low Density Lipoprotein Cholesterol; HDL-C: High Density Lipoprotein Cholesterol; TAGs: Triglycerides; SBP: Systolic Blood Pressure; DBP: Diastolic Blood Pressure; FMD: Flow Mediated Dilation; Hb1Ac: Glycated Haemoglobin; *n*: total number of studies included in the analysis; SDM: standardized difference in means (relative values); DM: Difference in means (units as indicated per each biomarker).

**Table 5:** Potential factors influencing the heterogeneity in the responses to the supplementation with ellagitannins and/or anthocyanins-containing products investigated in this meta-analysis.

Factors					
Baseline BMI	25.0 ^a^ (normal and (or) underweight)			≥25.0 (overweight and (or) obese)	
Sex	Women			Men	
Smoking	Non-smokers			Smokers	
Country where the study was conducted	East Asian countries (Japan, Korea, China)	All-other- countries-but-not-East Asian	North America (USA, Canada)	European countries	
				Non-Mediterranean countries (Denmark, Norway, Finland, The Netherlands, Germany, Poland, UK, Scotland, France, Czech Republic)	Mediterranean countries (Italy, Spain, Greece)
Medication	Yes			No	
Health status	Healthy individuals ^b^		Individuals ‘at a risk‘ of disease ^c^		Individuals with a reported disease ^d^
Main source of compounds	Ellagitannins			Anthocyannins	
	Pomegranate		Nuts	Berries	Red wine and red grapes
Diet during intervention	Controlled diet (specifically indicated to have restriction for the consumption of polyphenols or plant foods)			Usual diet (no changes in the usual diet of the participants or NR)	

^a^ BMI cut-off values as established by the WHO; ^b^ Includes individuals specifically reported as healthy and not medicated (in some cases medication was not reported, NR); ^c^ Includes individuals not medicated that were overweight and (or) obese, or specifically indicated to be borderline, mild condition or ‘at risk’ of a disease; ^d^ Includes individuals with one or more than one of the following disorders: dyslipidemia, glucose disorders or type-2 diabetes, blood pressure disorders (hypertension), medicated obesity, metabolic syndrome (most cases were also medicated but in some cases medication was NR).

## Supplementary Materials

The following are available online at http://www.mdpi.com/1422-0067/19/3/694/s1.

## Abbreviations

LDL-C	Low density lipoprotein cholesterol
HDL-C	High density lipoprotein cholesterol
ANCs	Anthocyanins
ETs	Ellagitannins
FW	Fresh weight
RCTs	Randomized-controlled trials
BMI	Body mass index
SDM	Standardized difference in means
DM	Difference in means
WC	Waist circumference
T-C	Total cholesterol
SBP	Systolic blood pressure
DBP	Diastolic blood pressure
FMD	Flow mediated dilation
TAGs	Triglycerides
HbA1c	Glycated hemoglobin
HOMA-IR	Homeostatic Model Assessment of Insulin Resistance
WHO	World health organization
NR	Not reported
GRADE	Grading of Recommendations Assessment, Development and Evaluation
