# Supplementary material for: Meta-Analysis of the Effects of Foods and Derived Products Containing Ellagitannins and Anthocyanins on Cardiometabolic Biomarkers: Analysis of Factors Influencing Variability of the Individual Responses
Source: Int J Mol Sci. 2018 Feb 28;19(3):694. doi: 10.3390/ijms19030694 (PMC5877555; doi:10.3390/ijms19030694)

Supplementary Figure S40. Overall effects of the ellagitannins and/or anthocyanins supplementation and the duration of the intervention (days) using random-effects meta-regression analysis on high density lipoprotein- cholesterol (HDL-C) (slope and 2-tailed *P*-value).

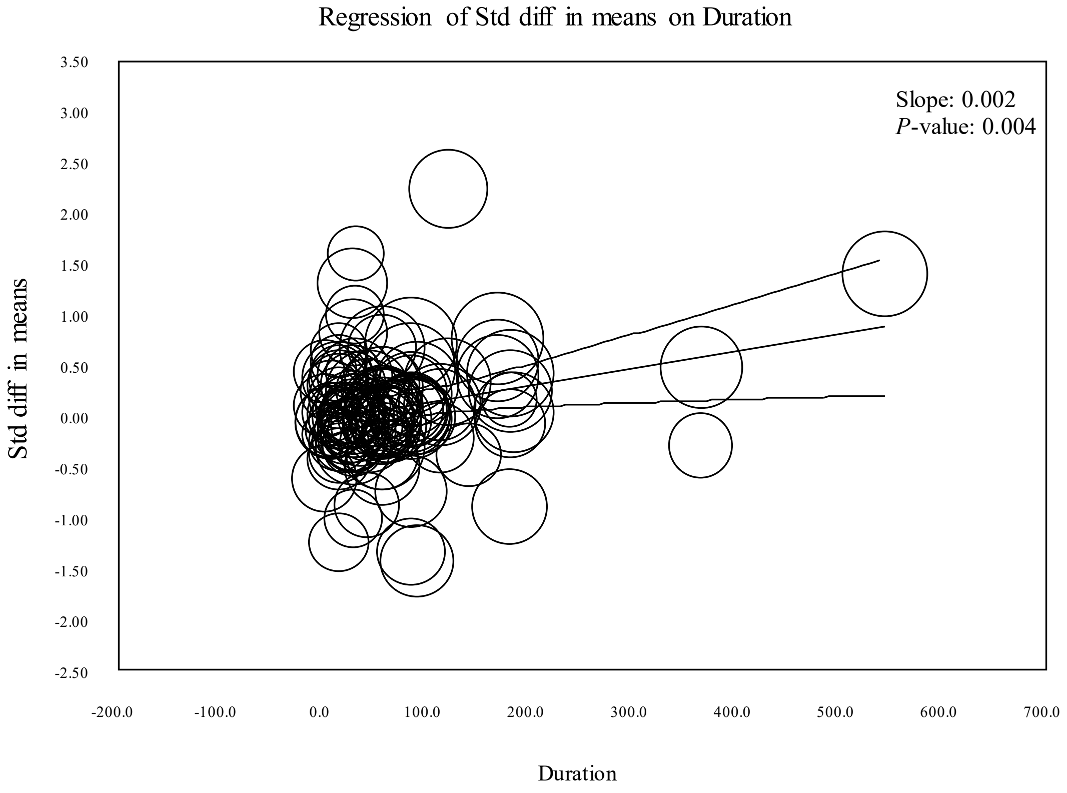

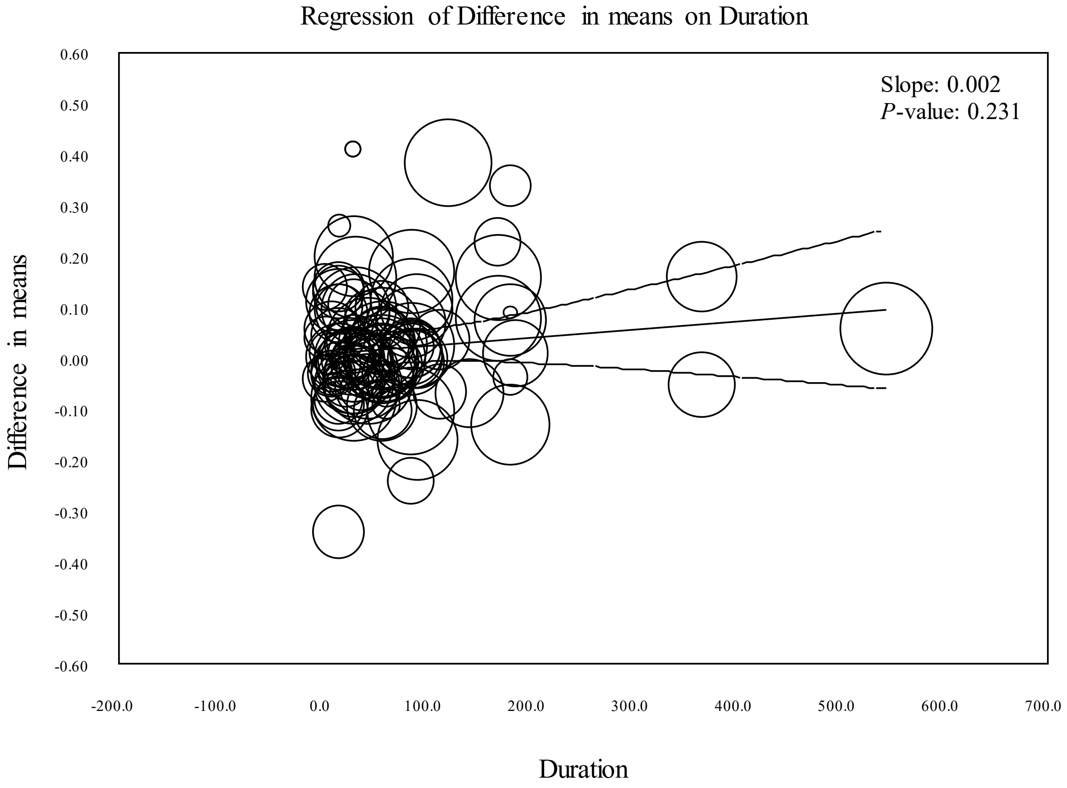

Supplement: Supplementary file 1 [file ijms-19-00694-s001.zip › Supplementary Figure S40.pdf]
