# Supplementary material for: Meta-Analysis of the Effects of Foods and Derived Products Containing Ellagitannins and Anthocyanins on Cardiometabolic Biomarkers: Analysis of Factors Influencing Variability of the Individual Responses
Source: Int J Mol Sci. 2018 Feb 28;19(3):694. doi: 10.3390/ijms19030694 (PMC5877555; doi:10.3390/ijms19030694)

Supplementary Figure S1.- Forest plot of the meta-analysis evaluating the effects of a prolonged supplementation with ellagitannins and anthocyanins-containing products on human BMI. A total of 51 studies (displayed in alphabetical order) were analysed. Pooled results are shown at the bottom using a random-effects model. SDM: standardized difference in means, SE: standard error, 95% CI: lower and upper confidence limits for the average SDM, RW: relative weight.

**First autor & year**	**SDM**	**SE**	**95% CI**		**RW**
Asgary 2014	-0.072	0.316	-0.692	0.548	1.73
Banini 2006	0.021	0.438	-0.837	0.879	0.90
Basu 2014	0.000	0.365	-0.716	0.716	1.30
Basu 2014	0.000	0.365	-0.716	0.716	1.30
Chiva-Blanch 2012	-0.023	0.324	-0.659	0.613	1.65
Chiva-Blanch 2012	-0.023	0.324	-0.659	0.613	1.65
Chiva-Blanch 2012	-0.027	0.372	-0.756	0.701	1.25
Chiva-Blanch 2012	-0.027	0.372	-0.756	0.701	1.25
Curtis 2009	-0.028	0.277	-0.571	0.516	2.25
Davinelli 2015	0.167	0.318	-0.457	0.791	1.71
Guo 2014	-0.093	0.427	-0.929	0.743	0.95
Hokayem 2013	0.079	0.325	-0.558	0.716	1.64
Hollis 2013	0.055	0.280	-0.494	0.604	2.21
Hosseini 2016	-0.179	0.309	-0.785	0.427	1.81
Jimenez 2008	-0.123	0.375	-0.858	0.613	1.23
Johnson 2015	0.254	0.310	-0.354	0.862	1.80
Katz 2012	0.566	0.322	-0.066	1.198	1.67
Khan 2014	0.018	0.305	-0.580	0.616	1.86
Khan 2014	0.015	0.309	-0.590	0.620	1.82
Kianbakht 2013	-0.175	0.224	-0.614	0.264	3.45
Lee 2014	0.000	0.258	-0.506	0.506	2.60
Lekakis 2005	-0.011	0.251	-0.504	0.481	2.75
Li 2010	-0.306	0.279	-0.853	0.242	2.22
López-Uriarte 2010	-0.260	0.284	-0.816	0.297	2.15
Lynn 2012	-0.016	0.289	-0.582	0.550	2.08
Ma 2010	0.258	0.439	-0.602	1.118	0.90
McAnulty 2014	-0.065	0.400	-0.849	0.720	1.08
Novotny 2015	-0.046	0.267	-0.571	0.478	2.42
Parham 2015	-0.398	0.412	-1.206	0.410	1.02
Park 2004	-0.068	0.317	-0.689	0.553	1.73
Park 2014	-0.181	0.228	-0.629	0.266	3.32
Qin 2009	-0.029	0.183	-0.387	0.329	5.20
Queipo-Ortuno 2012	0.000	0.632	-1.240	1.240	0.43
Queipo-Ortuno 2012	-0.035	0.633	-1.275	1.205	0.43
Riso 2013	-0.038	0.471	-0.962	0.886	0.78
Stull 2010	0.000	0.354	-0.694	0.694	1.38
Tey 2013	0.000	0.239	-0.469	0.469	3.02
Tey 2013	0.000	0.232	-0.456	0.456	3.20
Tsang 2012	-0.528	0.385	-1.282	0.226	1.17
Urquiaga 2015	-0.054	0.342	-0.724	0.617	1.48
Velliquette 2015	-0.000	0.354	-0.694	0.694	1.38
Velliquette 2015	-0.055	0.343	-0.727	0.618	1.47
Wang 2012	0.285	0.267	-0.238	0.808	2.44
Wang 2012	-0.391	0.263	-0.906	0.124	2.51
Wien 2010	-0.025	0.273	-0.560	0.510	2.32
Wu 2014	-0.081	0.316	-0.701	0.539	1.73
Wu 2015	-0.120	0.386	-0.876	0.636	1.17
Yubero 2013	-0.026	0.258	-0.532	0.480	2.60
Yubero 2013	0.000	0.258	-0.506	0.506	2.60
Zhang 2015	0.089	0.253	-0.407	0.584	2.71
Zhu 2013	-0.143	0.166	-0.468	0.182	6.31
**Intervention effect**	**-0.042**	**0.042**	**-0.123**	**0.040**	

Outcome: BMI

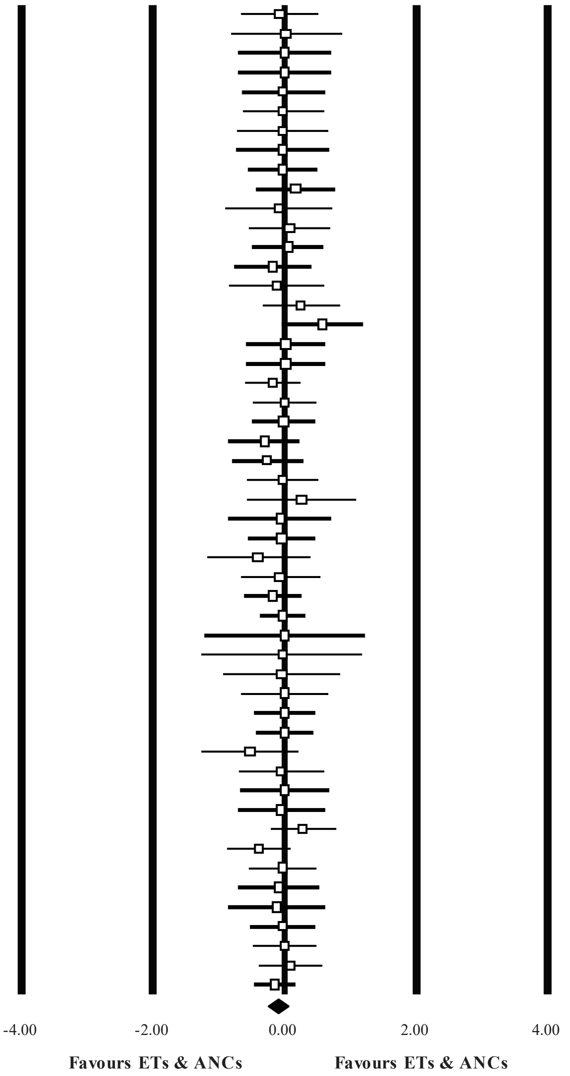

Test for overall effect Z=-1,005; *P*=0.315; Ns=1194; Nc=1149

Heterogeneity Tau^2=0.000; Q=14,7; df=50 (*P*=1.000); I^2=0.000

Supplementary Figure S2.- Forest plot of the meta-analysis evaluating the effects of a prolonged supplementation with ellagitannins and anthocyanins-containing products on human BMI. A total of 47 studies (displayed in alphabetical order) were analysed. Pooled results are shown at the bottom using a random-effects model. DM: Difference in means, SE: standard error, 95% CI: lower and upper confidence limits for the average DM, RW: relative weight.

First autor & year	DM	SE	95%CI		RW
Asgary 2014	-0.255	1.131	-2.473	1.963	0.00
Banini 2006	0.100	2.124	-4.062	4.262	0.00
Basu 2014	0.000	1.556	-3.049	3.049	0.00
Basu 2014	0.000	2.263	-4.435	4.435	0.00
Chiva-Blanch 2012	-0.100	1.420	-2.883	2.683	0.00
Chiva-Blanch 2012	-0.100	1.395	-2.835	2.635	0.00
Chiva-Blanch 2012	-0.100	1.266	-2.582	2.382	0.00
Chiva-Blanch 2012	-0.100	1.274	-2.597	2.397	0.00
Curtis 2009	-0.100	1.001	-2.062	1.862	0.00
Davinelli 2015	0.800	1.458	-2.058	3.658	0.00
Guo 2014	-0.200	0.964	-2.089	1.689	0.00
Hokayem 2013	0.200	0.864	-1.494	1.894	0.00
Hollis 2013	0.100	0.479	-0.840	1.040	0.01
Hosseini 2016	-0.800	1.387	-3.519	1.919	0.00
Jimenez 2008	-0.500	1.581	-3.599	2.599	0.00
Johnson 2015	1.600	1.962	-2.246	5.446	0.00
Katz 2012	0.400	0.224	-0.038	0.838	0.06
Khan 2014	0.100	1.767	-3.363	3.563	0.00
Khan 2014	0.100	2.086	-3.989	4.189	0.00
Lee 2014	0.000	0.124	-0.243	0.243	0.19
Lekakis 2005	-0.200	4.108	-8.252	7.852	0.00
Li 2010	-0.700	0.606	-1.888	0.488	0.01
Lynn 2012	-0.090	1.643	-3.310	3.130	0.00
Ma 2010	0.300	0.509	-0.697	1.297	0.01
McAnulty 2014	-0.290	1.821	-3.859	3.279	0.00
Novotny 2015	-0.200	1.131	-2.417	2.017	0.00
Parham 2015	-0.500	0.513	-1.506	0.506	0.01
Park 2004	-0.200	0.932	-2.026	1.626	0.00
Park 2014	-0.180	0.226	-0.624	0.264	0.06
Qin 2009	-0.100	0.641	-1.356	1.156	0.01
Queipo-Ortuno 2012	0.000	1.952	-3.826	3.826	0.00
Queipo-Ortuno 2012	-0.100	1.923	-3.869	3.669	0.00
Riso 2013	-0.100	1.227	-2.504	2.304	0.00
Stull 2010	0.000	1.312	-2.571	2.571	0.00
Tey 2013	0.000	0.188	-0.368	0.368	0.08
Tey 2013	0.000	0.204	-0.400	0.400	0.07
Urquiaga 2015	-0.200	1.284	-2.716	2.316	0.00
Velliquette 2015	-0.000	1.236	-2.423	2.423	0.00
Velliquette 2015	-0.200	1.263	-2.676	2.276	0.00
Wang 2012	0.010	0.009	-0.008	0.028	33.49
Wang 2012	-0.010	0.007	-0.023	0.003	65.38
Wien 2010	-0.700	7.043	-14.503	13.103	0.00
Wu 2015	-1.000	3.252	-7.373	5.373	0.00
Yubero 2013	-0.010	0.101	-0.207	0.187	0.29
Yubero 2013	0.000	0.101	-0.197	0.197	0.29
Zhang 2015	0.300	0.851	-1.368	1.968	0.00
Zhu 2013	-0.300	1.070	-2.397	1.797	0.00
**Intervention effect**	**-0.003**	**0.005**	**-0.014**	**0.007**	

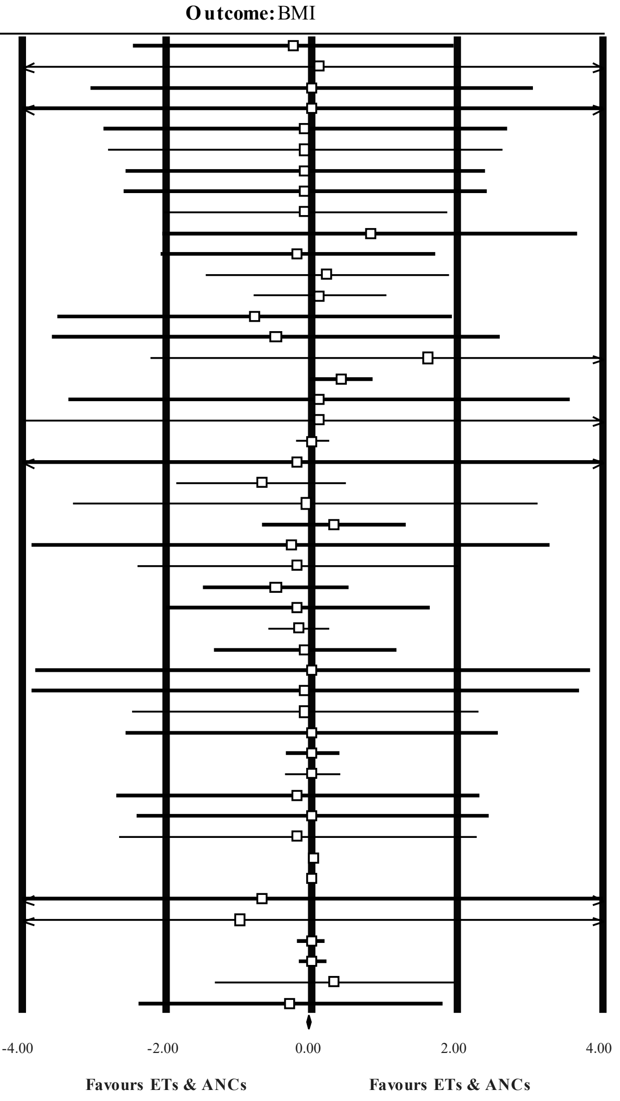

Test for overall effect Z=-0.592; *P*=0.554; Ns=1095; Nc=1050

Heterogeneity Tau²=0.000; Q=11,7; df=46 (*P*=1.000); I²=0.000

Supplementary Figure S3. Funnel plots and Egger statistics (intercept and 2-tailed *P*-value) of the meta-analysis evaluating the effects of a prolonged supplementation with ellagitannins and anthocyanins-containing products on human BMI.

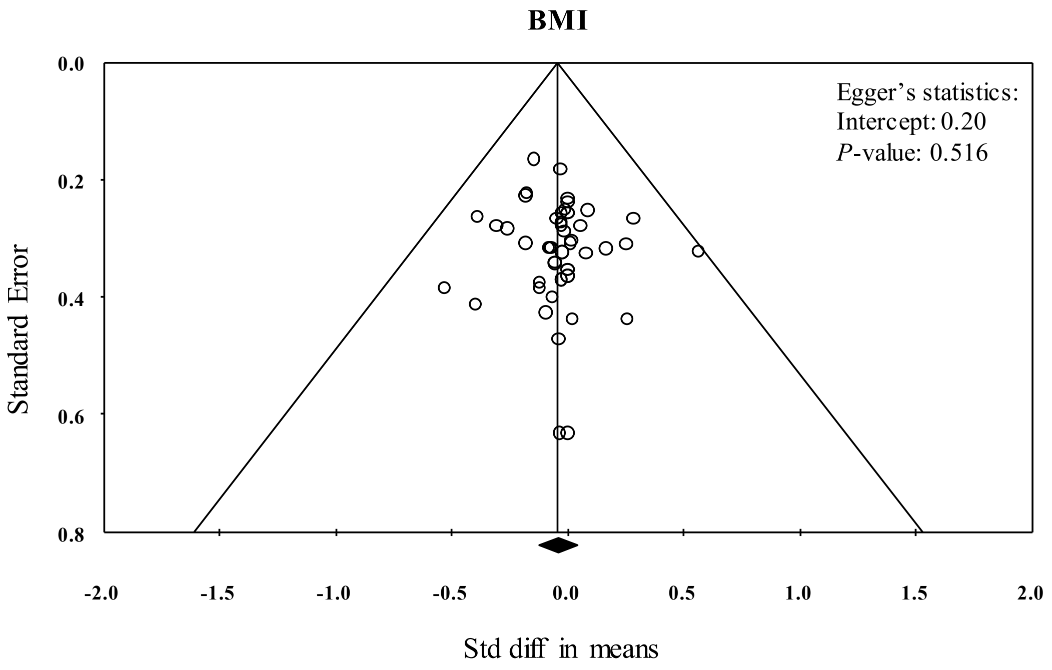

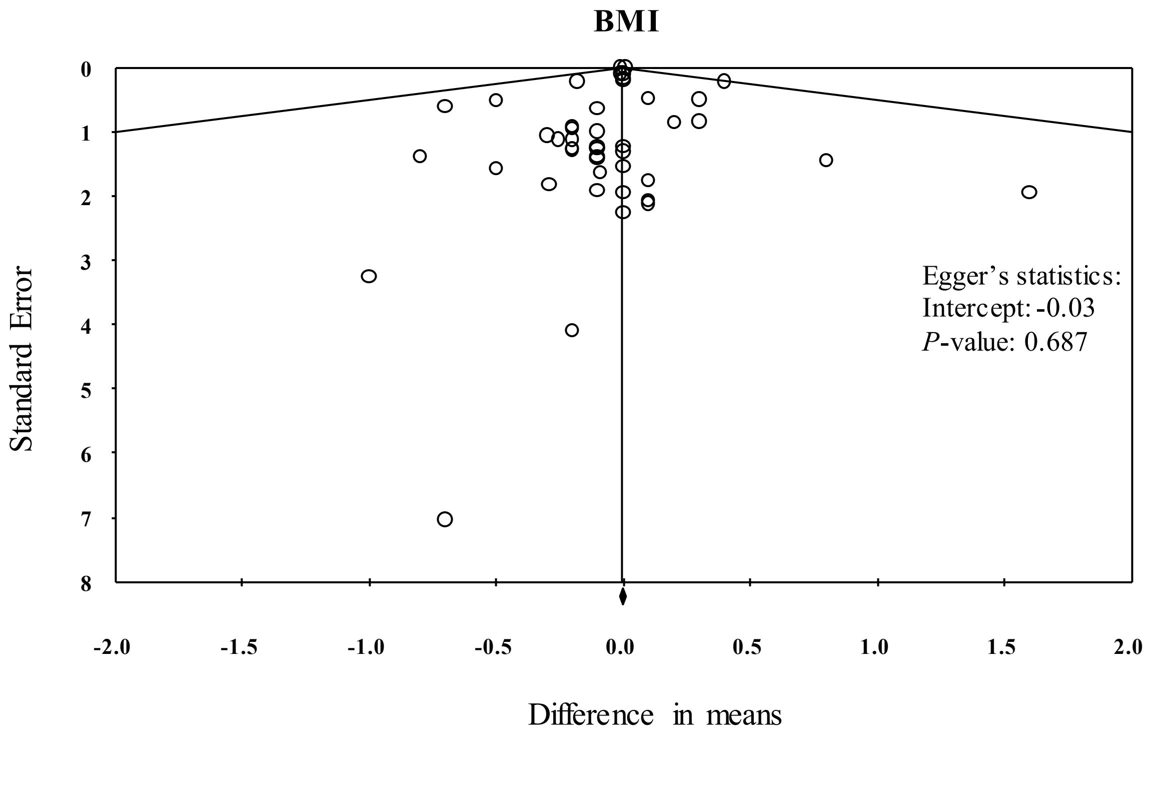

Supplementary Figure S4. Forest plot of the meta-analysis evaluating the effects of a prolonged supplementation with ellagitannins and anthocyanins-containing products on WC. A total of 23 studies (displayed in alphabetical order) were analysed. Pooled results are shown at the bottom using a random-effects model. SDM: standardized difference in means, SE: standard error, 95% CI: lower and upper confidence limits for the average SDM, RW: relative weight.

First autor & year	SDM	SE	95%CI		RW
Basu 2010a	-0.026	0.289	-0.593	0.540	4.97
Basu 2010b	0.328	0.390	-0.436	1.092	3.90
Basu 2014	-0.679	0.376	-1.415	0.057	4.04
Basu 2014	-0.860	0.382	-1.608	-0.112	3.98
Davinelli 2015	0.098	0.318	-0.525	0.721	4.64
Gulati 2014	-1.385	0.287	-1.948	-0.821	4.99
Guo 2014	0.186	0.427	-0.652	1.023	3.56
Hokayem 2013	-0.045	0.325	-0.682	0.592	4.57
Hollis 2013	0.032	0.280	-0.517	0.581	5.07
Johnson 2015	-0.447	0.321	-1.075	0.182	4.61
Johnston 2013	-2.471	0.405	-3.264	-1.677	3.76
Katz 2012	-0.128	0.409	-0.929	0.673	3.73
Krikorian 2012	-0.056	0.437	-0.913	0.800	3.48
Lee 2014	-0.481	0.262	-0.994	0.033	5.28
Ma 2010	-0.058	0.426	-0.894	0.778	3.57
Qin 2009	-0.054	0.183	-0.412	0.303	6.22
Queipo-Ortuno 2012	-0.057	0.633	-1.297	1.183	2.19
Queipo-Ortuno 2012	-0.043	0.633	-1.283	1.197	2.19
Urquiaga 2015	-0.010	0.342	-0.681	0.660	4.38
Wien 2010	-0.161	0.273	-0.697	0.374	5.15
Wu 2015	-0.180	0.386	-0.936	0.577	3.94
Zhang 2015	-0.190	0.253	-0.687	0.306	5.39
Zhu 2013	-0.134	0.166	-0.459	0.191	6.40
**Intervention effect**	**-0.296**	**0.064**	**-0.515**	**-0.078**	

Test for overall effect Z=-2.659; *P*=0.008; Ns=528; Nc=498

Heterogeneity Tau²=0.167; Q=60.1; df=22 (*P*=0.000); I²=63.4

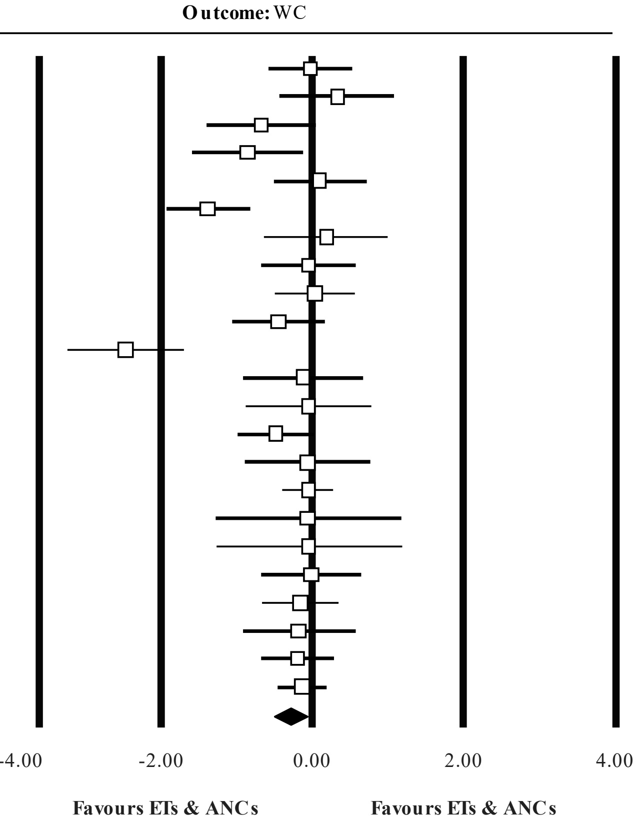

Supplementary Figure S5.- Forest plot of the meta-analysis evaluating the effects of a prolonged supplementation with ellagitannins and anthocyanins-containing products on human WC. A total of 22 studies (displayed in alphabetical order) were analysed. Pooled results are shown at the bottom using a random-effects model. DM: Difference in means, SE: standard error, 95% CI: lower and upper confidence limits for the average DM, RW: relative weight.

First autor & year	DM	SE	95%CI		RW
Basu 2010a	-0.100	1.100	-2.255	2.055	9.31
Basu 2010b	3.302	3.589	-3.733	10.337	1.38
Basu 2014	-5.000	2.764	-10.417	0.417	2.24
Basu 2014	-7.000	3.480	-13.821	-0.179	1.47
Davinelli 2015	0.900	2.945	-4.871	6.671	2.00
Gulati 2014	-3.600	1.876	-7.276	0.076	4.36
Guo 2014	1.300	2.968	-4.517	7.117	1.97
Hokayem 2013	-0.400	2.825	-5.936	5.136	2.15
Hollis 2013	0.255	2.080	-3.821	4.331	3.68
Johnson 2015	-11.500	7.096	-25.408	2.408	0.37
Johnston 2013	-2.600	0.321	-3.229	-1.971	20.36
Katz 2012	-0.400	1.273	-2.895	2.095	7.74
Krikorian 2012	-0.600	4.548	-9.515	8.315	0.88
Lee 2014	-0.620	0.333	-1.273	0.033	20.19
Ma 2010	-0.300	2.216	-4.643	4.043	3.30
Qin 2009	-0.500	1.614	-3.663	2.663	5.53
Queipo-Ortuno 2012	-0.800	8.940	-18.322	16.722	0.23
Queipo-Ortuno 2012	-0.600	8.956	-18.154	16.954	0.23
Urquiaga 2015	-0.900	28.072	-55.920	54.120	0.02
Wu 2015	-3.900	8.537	-20.631	12.831	0.26
Zhang 2015	-1.500	1.961	-5.343	2.343	4.06
Zhu 2013	-1.000	1.213	-3.377	1.377	8.25
**Intervention effect**	**-1.219**	**0.209**	**-2.073**	**-0.365**	

Test for overall effect Z=-2.799; P=0.005; Ns=503; Nc=469

Heterogeneity Tau^2=0.828; Q=33.6; df=21 (P=0.040); I^2=37.5

Outcome: WC

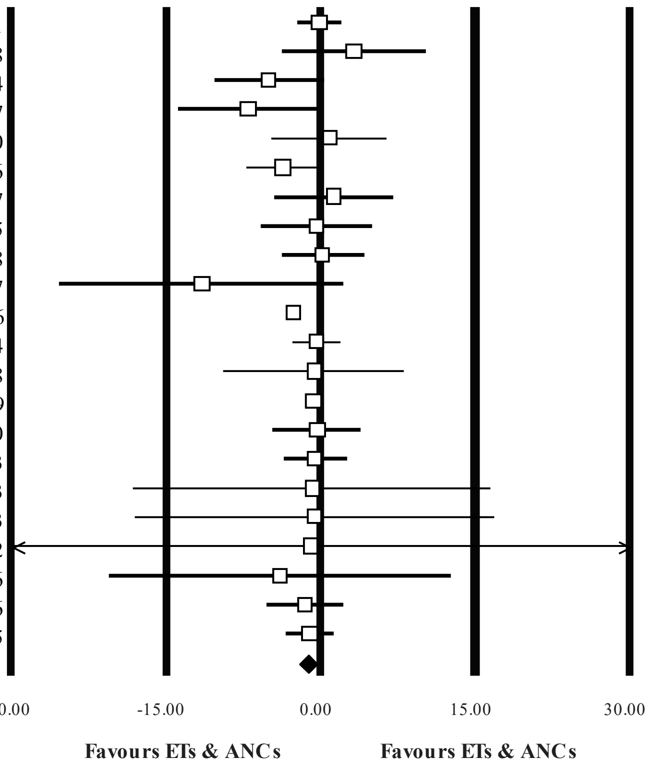

Supplementary Figure S6. Funnel plots and Egger statistics (intercept and 2-tailed *P*-value) of the meta-analysis evaluating the effects of a prolonged supplementation with ellagitannins and anthocyanins-containing products on waist circumference (WC).

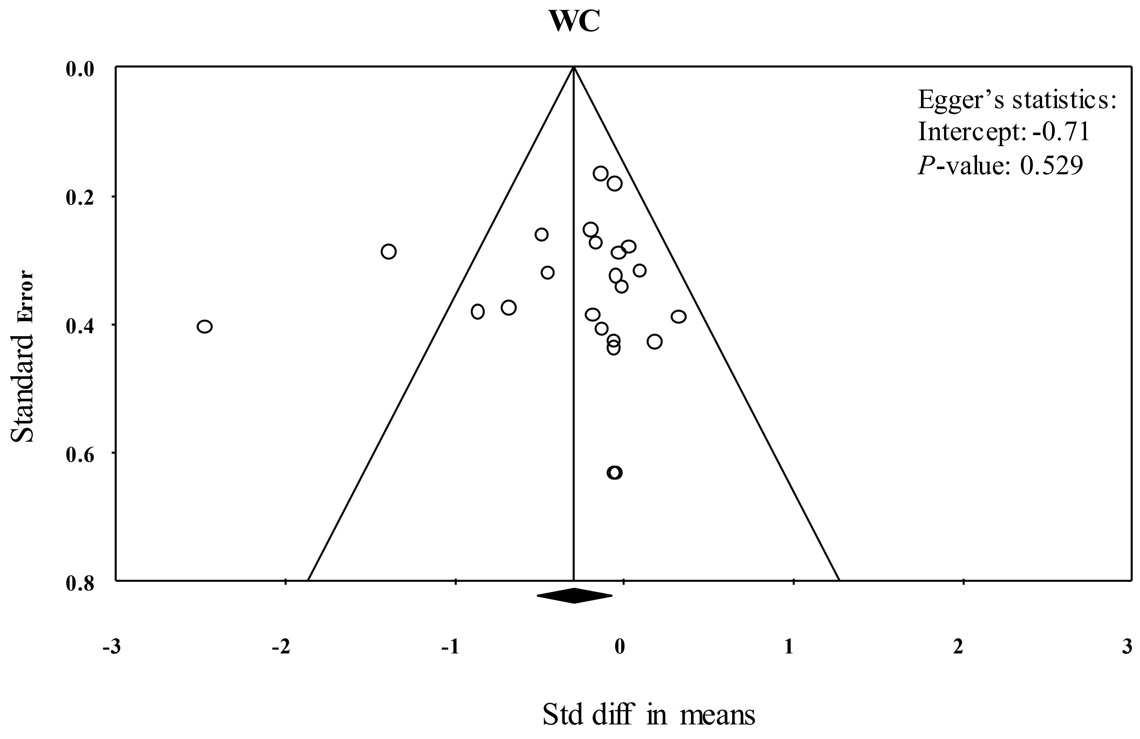

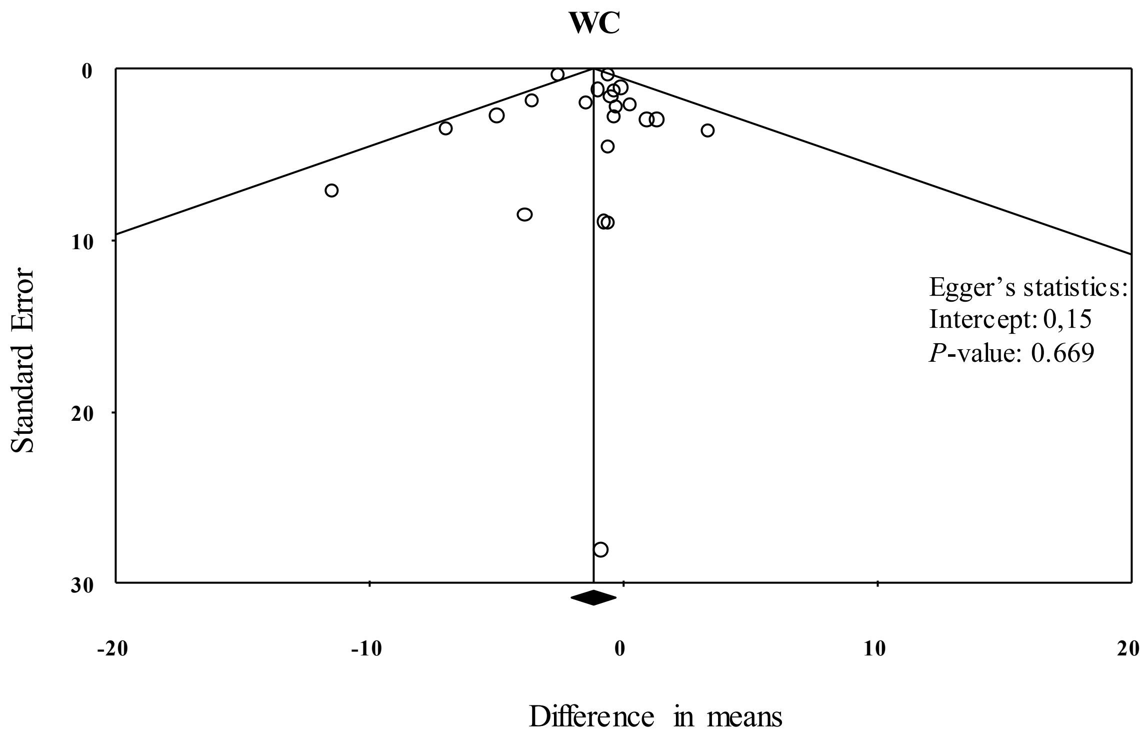

Supplementary Figure S7. Forest plot of the meta-analysis evaluating the effects of a prolonged supplementation with ellagitannins and anthocyanins-containing products on T-C. A total of 109 studies (displayed in alphabetical order) were analysed. Pooled results are shown at the bottom using a random-effects model. SDM: standardized difference in means, SE: standard error, 95% CI: lower and upper confidence limits for the average SDM, RW: relative weight.

Outcome: T-C

First autor & year	SDM	SE	95%CI		RW
Abu-Amsha Caccetta 2001	0.108	0.486	1.061	-0.845	0.46
Abu-Amsha Caccetta 2001	0.059	0.486	1.011	-0.894	0.38
Asgary 2013	-0.503	0.455	0.388	-1.394	-0.56
Asgary 2014	0.273	0.439	1.134	-0.587	0.78
Banini 2006	-0.928	0.459	-0.029	-1.827	-1.28
Basu 2010a	0.000	0.289	0.566	-0.566	0.37
Basu 2010b	-0.676	0.398	0.104	-1.456	-0.93
Basu 2011	-0.323	0.362	0.386	-1.032	-0.29
Basu 2014	0.754	0.378	1.495	0.014	1.75
Basu 2014	-4.048	0.637	-2.798	-5.297	-5.26
Bommannan Santhakumar 2015	0.392	0.562	1.493	-0.709	0.84
Bommannan Santhakumar 2015	0.033	0.556	1.123	-1.058	0.31
Burton-Freeman 2010	-0.229	0.410	0.575	-1.033	-0.10
Caldu 1998	0.114	0.472	1.038	-0.811	0.48
Castilla 2006	0.460	0.353	1.152	-0.232	1.24
Castilla 2008	-0.469	0.532	0.573	-1.512	-0.46
Cerdá 2066	0.007	0.365	0.723	-0.709	0.35
Clifton 2004	-0.010	0.333	0.643	-0.663	0.33
Curtis 2009	0.372	0.280	0.920	-0.176	1.18
Davinelli 2015	-0.023	0.318	0.600	-0.646	0.31
Din 2011	-0.342	0.368	0.379	-1.063	-0.32
Dohadwala 2011	-0.049	0.426	0.786	-0.885	0.22
Duthie 2006	-0.153	0.451	0.730	-1.037	0.03
Erlund 2008	-0.289	0.239	0.179	-0.756	-0.26
Estruch 2011	0.055	0.447	0.931	-0.822	0.39
Flammer 2013	-0.167	0.216	0.256	-0.591	0.01
Foster 2012	-1.219	0.265	-0.699	-1.738	-2.30
Goldberg 1996	0.255	0.428	1.094	-0.584	0.76
Gorinstein 2006	1.683	0.534	2.731	0.636	2.86
Gorinstein 2066	3.451	0.724	4.870	2.031	4.46
Gulati 2014	-0.243	0.259	0.265	-0.751	-0.15
Guo 2014	-0.533	0.434	0.317	-1.384	-0.63
Haddad 2014	-0.038	0.500	0.942	-1.018	0.22
Hansen 2005	-0.504	0.344	0.169	-1.177	-0.66
Hansen 2005	-0.549	0.356	0.149	-1.247	-0.73
Hansen 2005	-0.308	0.331	0.340	-0.957	-0.27
Hassellund 2013	-0.308	0.387	0.452	-1.067	-0.25
Heber 2007	0.055	0.424	0.886	-0.777	0.40
Heber 2007	-0.030	0.423	0.798	-0.858	0.25
Hijmering 2007	0.278	0.450	1.160	-0.604	0.77
Hokayem 2013	-0.084	0.325	0.553	-0.721	0.18
Hollis 2013	0.013	0.280	0.562	-0.536	0.40
Hosseini 2016	0.455	0.313	1.068	-0.158	1.30
Huebbe 2012	-0.077	0.606	1.111	-1.264	0.14
Jenkins 2008	0.335	0.381	1.081	-0.411	0.96
Karlsen 2010	-0.232	0.255	0.268	-0.731	-0.13
Katz 2012	-0.036	0.302	0.555	-0.627	0.29
Kay 2002	-0.069	0.707	1.317	-1.456	0.13
Khan 2014	-0.131	0.309	0.475	-0.736	0.09
Khan 2014	0.000	0.305	0.598	-0.598	0.36
Kianbakht 2014	-2.284	0.287	-1.721	-2.847	-4.51
Krikorian 2010	-0.439	0.442	0.427	-1.306	-0.46
Lee 2014	-0.517	0.262	-0.003	-1.032	-0.76
Lekakis 2005	-0.029	0.251	0.463	-0.521	0.32
Li 2010	-0.292	0.274	0.245	-0.828	-0.26
Lynn 2014	-0.536	0.312	0.077	-1.148	-0.75
Ma 2010	-0.267	0.439	0.593	-1.128	-0.16
Mukuddem-Petersen 2007	-0.228	0.306	0.372	-0.827	-0.11
Naissides 2006	-0.208	0.360	0.499	-0.914	-0.07
Naissides 2006	-0.211	0.367	0.509	-0.930	-0.07
Natella 2011	-0.071	0.578	1.061	-1.203	0.15
Nigdikar 1998	0.392	0.532	1.434	-0.650	0.87
Nigdikar 1998	-0.537	0.536	0.514	-1.587	-0.56
Nigdikar 1998	-0.087	0.527	0.947	-1.120	0.13
Novotny 2015	-0.252	0.268	0.274	-0.778	-0.17
Olmedilla-Alonso 2008	-0.163	0.401	0.623	-0.949	0.02
Park 2014	-0.107	0.228	0.341	-0.554	0.15
Pérez-Jímenez 2008	-0.370	0.377	0.369	-1.109	-0.37
Preuss 2000	-0.088	0.460	0.812	-0.989	0.14
Preuss 2000	-0.342	0.463	0.565	-1.249	-0.29
Puupponen-Pimiä 2013	-0.249	0.366	0.469	-0.967	-0.15
Qin 2009	-0.103	0.183	0.255	-0.461	0.17
Queipo-Ortuno 2012	-0.063	0.633	1.177	-1.303	0.15
Queipo-Ortuno 2012	-0.599	0.646	0.668	-1.866	-0.57
Rahbar 2015	-0.893	0.313	-0.280	-1.506	-1.49
Rahbar 2015	-0.516	0.297	0.065	-1.097	-0.72
Razavi 2013	-0.691	0.339	-0.027	-1.354	-1.03
Riso 2013	0.000	0.471	0.924	-0.924	0.29
Santhakumar 2015	0.033	0.556	1.123	-1.058	0.31
Shema-Didi 2014	-0.076	0.251	0.415	-0.568	0.22
Sivaprakasapillai 2010	-0.476	0.478	0.461	-1.413	-0.50
Sivaprakasapillai 2010	0.304	0.474	1.233	-0.625	0.79
Spaccarotella 2008	-0.287	0.439	0.573	-1.148	-0.20
Stull 2010	-0.058	0.354	0.637	-0.752	0.22
Sumner 2005	0.471	0.321	1.101	-0.159	1.31
Tey 2013	-0.127	0.349	0.557	-0.810	0.09
Tey 2013	-0.113	0.329	0.532	-0.758	0.12
Tomé-Carneiro 2012	-0.189	0.283	0.366	-0.745	-0.04
Tome-Carneiro 2013	-0.176	0.434	0.675	-1.028	-0.01
Tsang 2005	0.601	0.466	1.515	-0.312	1.30
Tsang 2012	0.227	0.379	0.970	-0.516	0.75
Umar 2005	-0.281	0.449	0.600	-1.161	-0.18
Vaisman 2015	0.164	0.355	0.859	-0.532	0.66
Vaisman 2015	-0.431	0.426	0.404	-1.267	-0.46
Velliquette 2015	-0.100	0.354	0.595	-0.795	0.14
Velliquette 2015	-0.158	0.344	0.516	-0.831	0.03
Vidlar 2010	-0.101	0.310	0.508	-0.709	0.15
Wang 2012	-0.232	0.261	0.280	-0.744	-0.13
Wang 2012	0.019	0.265	0.539	-0.501	0.42
Whelan 2004	0.000	0.535	1.048	-1.048	0.27
Wien 2010	-0.475	0.277	0.068	-1.017	-0.65
Wu 2014	-0.372	0.319	0.253	-0.997	-0.41
Wu 2015	0.010	0.385	0.765	-0.745	0.34
Yubero 2013	-1.645	0.299	-1.060	-2.231	-3.10
Zern 2005	0.091	0.447	0.968	-0.786	0.45
Zern 2005	0.095	0.408	0.896	-0.705	0.49
Zhang 2015	0.171	0.253	0.667	-0.326	0.77
Zhu 2011	-0.022	0.166	0.303	-0.346	0.37
Zhu 2013	-0.048	0.166	0.276	-0.373	0.31
**Intervention effect**	**-0.173**	**0.033**	**-0.272**	**-0.074**	

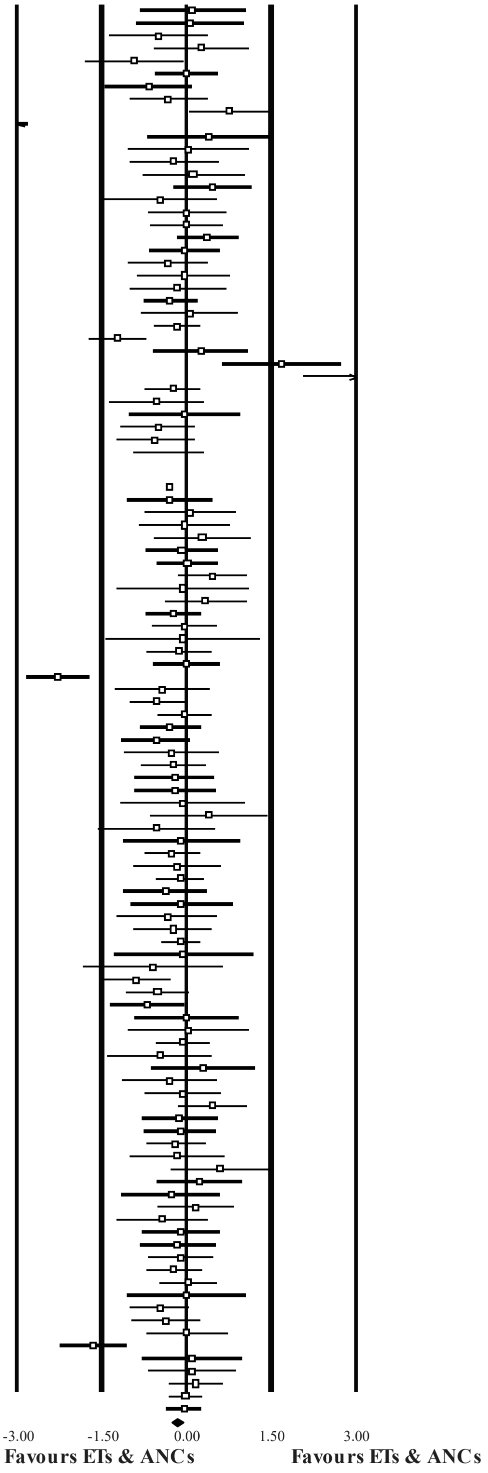

Test for overall effect Z=-3.421; P=0.001; Ns=2060; Nc=1931

Heterogeneity Tau^2=0.139; Q=237.4; df=108 (P=0.000); I^2=54.5

Supplementary Figure S8.- Forest plot of the meta-analysis evaluating the effects of a prolonged supplementation with ellagitannins and anthocyanins-containing products on T-C. A total of 103 studies (displayed in alphabetical order) were analysed. Pooled results are shown at the bottom using a random-effects model. DM: Difference in means, SE: standard error, 95% CI: lower and upper confidence limits for the average DM, RW: relative weight.

First autor & year	DM	SE	95% CI		RW
Abu-Amsha Caccetta 2001	0.120	0.531	1.161	-0.921	0.45
Abu-Amsha Caccetta 2001	0.070	0.568	1.184	-1.044	0.40
Asgary 2013	-0.524	0.513	0.482	-1.530	0.47
Asgary 2014	0.264	0.411	1.070	-0.542	0.65
Banini 2006	-0.895	0.438	-0.036	-1.754	0.59
Basu 2010a	0.000	0.355	0.696	-0.696	0.79
Basu 2010b	-0.500	0.325	0.138	-1.138	0.87
Basu 2011	-0.274	0.298	0.311	-0.859	0.96
Basu 2014	0.207	0.093	0.389	0.025	1.86
Basu 2014	-0.828	0.076	-0.678	-0.977	1.92
Bommannan Santhakumar 2015	0.280	0.552	1.362	-0.802	0.42
Bommannan Santhakumar 2015	0.020	0.411	0.826	-0.786	0.65
Burton-Freeman 2010	-0.179	1.611	2.979	-3.337	0.06
Caldu 1998	0.112	0.483	1.059	-0.835	0.52
Castilla 2006	0.430	0.340	1.097	-0.237	0.83
Castilla 2008	-0.155	0.171	0.179	-0.489	1.50
Cerdá 2066	0.006	0.359	0.710	-0.697	0.78
Clifton 2004	-0.010	0.345	0.666	-0.686	0.82
Curtis 2009	0.300	0.208	0.707	-0.107	1.33
Davinelli 2015	-0.020	0.240	0.451	-0.491	1.18
Din 2011	-0.130	0.139	0.142	-0.402	1.66
Dohadwala 2011	-0.052	0.447	0.824	-0.927	0.58
Duthie 2006	-0.100	0.304	0.495	-0.695	0.94
Estruch 2011	0.052	0.425	0.885	-0.782	0.62
Foster 2012	-0.084	0.017	-0.052	-0.116	2.06
Goldberg 1996	0.260	0.446	1.133	-0.613	0.58
Gorinstein 2006	0.600	0.164	0.921	0.279	1.54
Gorinstein 2066	1.230	0.164	1.551	0.909	1.54
Gulati 2014	-0.119	0.204	0.282	-0.520	1.34
Guo 2014	-0.310	0.260	0.199	-0.820	1.10
Haddad 2014	-0.040	0.582	1.101	-1.181	0.38
Hansen 2005	-0.230	0.154	0.072	-0.532	1.58
Hansen 2005	-0.240	0.153	0.060	-0.540	1.59
Hansen 2005	-0.150	0.160	0.164	-0.464	1.55
Heber 2007	0.272	1.976	4.144	-3.601	0.04
Heber 2007	-0.021	0.300	0.568	-0.610	0.95
Hijmering 2007	0.260	0.431	1.105	-0.585	0.61
Hokayem 2013	-0.070	0.260	0.440	-0.580	1.10
Hollis 2013	0.010	0.220	0.441	-0.421	1.27
Hosseini 2016	0.442	0.318	1.066	-0.181	0.90
Huebbe 2012	-0.070	0.605	1.115	-1.255	0.36
Jenkins 2008	0.260	0.288	0.824	-0.304	1.00
Karlsen 2010	-0.150	0.164	0.172	-0.472	1.53
Katz 2012	-0.021	0.175	0.322	-0.363	1.48
Kay 2002	-0.060	0.559	1.035	-1.155	0.41
Khan 2014	-0.100	0.262	0.413	-0.613	1.10
Khan 2014	0.000	0.259	0.508	-0.508	1.11
Kianbakht 2014	-1.863	0.188	-1.495	-2.231	1.42
Krikorian 2010	-0.222	0.228	0.225	-0.669	1.24
Lee 2014	-0.275	0.137	-0.006	-0.544	1.66
Lekakis 2005	-0.150	1.255	2.310	-2.610	0.10
Li 2010	-0.296	0.266	0.226	-0.818	1.08
Lynn 2014	-0.390	0.225	0.052	-0.832	1.25
Ma 2010	-0.134	0.220	0.296	-0.565	1.27
Naissides 2006	-0.200	0.324	0.436	-0.836	0.88
Naissides 2006	-0.130	0.218	0.296	-0.556	1.28
Natella 2011	-0.053	0.422	0.775	-0.880	0.63
Nigdikar 1998	0.350	0.573	1.473	-0.773	0.39
Nigdikar 1998	-0.500	0.493	0.466	-1.466	0.50
Nigdikar 1998	-0.110	0.673	1.208	-1.428	0.30
Novotny 2015	-0.080	0.179	0.270	-0.430	1.46
Olmedilla-Alonso 2008	-0.176	0.429	0.664	-1.016	0.61
Park 2014	-0.101	0.217	0.324	-0.526	1.29
Pérez-Jímenez 2008	-0.494	0.551	0.585	-1.573	0.42
Preuss 2000	-0.078	0.374	0.656	-0.811	0.74
Preuss 2000	-0.310	0.372	0.420	-1.040	0.74
Puupponen-Pimiä 2013	-0.200	0.289	0.367	-0.767	0.99
Qin 2009	-0.098	0.172	0.240	-0.436	1.49
Queipo-Ortuno 2012	-0.153	1.488	2.764	-3.069	0.07
Queipo-Ortuno 2012	-1.208	1.383	1.502	-3.918	0.08
Rahbar 2015	-0.489	0.158	-0.179	-0.798	1.56
Rahbar 2015	-0.419	0.228	0.028	-0.865	1.24
Riso 2013	0.000	0.591	1.158	-1.158	0.38
Santhakumar 2015	0.020	0.411	0.826	-0.786	0.65
Shema-Didi 2014	-0.077	0.244	0.402	-0.555	1.17
Sivaprakasapillai 2010	-0.129	0.129	0.124	-0.382	1.70
Sivaprakasapillai 2010	0.103	0.175	0.446	-0.239	1.48
Spaccarotella 2008	-0.160	0.243	0.317	-0.637	1.17
Stull 2010	-0.050	0.297	0.533	-0.633	0.96
Sumner 2005	0.465	0.317	1.087	-0.156	0.90
Tey 2013	-0.120	0.341	0.548	-0.788	0.83
Tey 2013	-0.110	0.331	0.538	-0.758	0.86
Tomé-Carneiro 2012	-0.168	0.231	0.285	-0.622	1.22
Tome-Carneiro 2013	-0.181	0.435	0.672	-1.034	0.60
Tsang 2005	0.500	0.471	1.423	-0.423	0.54
Tsang 2012	0.180	0.276	0.720	-0.360	1.04
Umar 2005	-0.260	0.409	0.542	-1.062	0.65
Vaisman 2015	0.129	0.278	0.674	-0.416	1.03
Vaisman 2015	-0.334	0.331	0.314	-0.982	0.86
Velliquette 2015	-0.103	0.386	0.653	-0.860	0.71
Velliquette 2015	-0.155	0.336	0.504	-0.814	0.84
Vidlar 2010	-0.070	0.230	0.381	-0.521	1.23
Wang 2012	-0.230	0.260	0.279	-0.739	1.11
Wang 2012	0.020	0.268	0.545	-0.505	1.07
Whelan 2004	0.000	0.323	0.633	-0.633	0.88
Wu 2014	-0.155	0.132	0.103	-0.414	1.69
Wu 2015	0.016	0.513	1.021	-0.990	0.47
Yubero 2013	-0.598	0.108	-0.386	-0.809	1.80
Zern 2005	0.100	0.467	1.016	-0.816	0.54
Zern 2005	0.100	0.425	0.933	-0.733	0.62
Zhang 2015	0.180	0.257	0.684	-0.324	1.12
Zhu 2011	-0.018	0.148	0.272	-0.308	1.61
Zhu 2013	-0.040	0.147	0.248	-0.328	1.62
**Intervention effect**	**-0.099**	**0.040**	**-0.177**	**-0.020**	

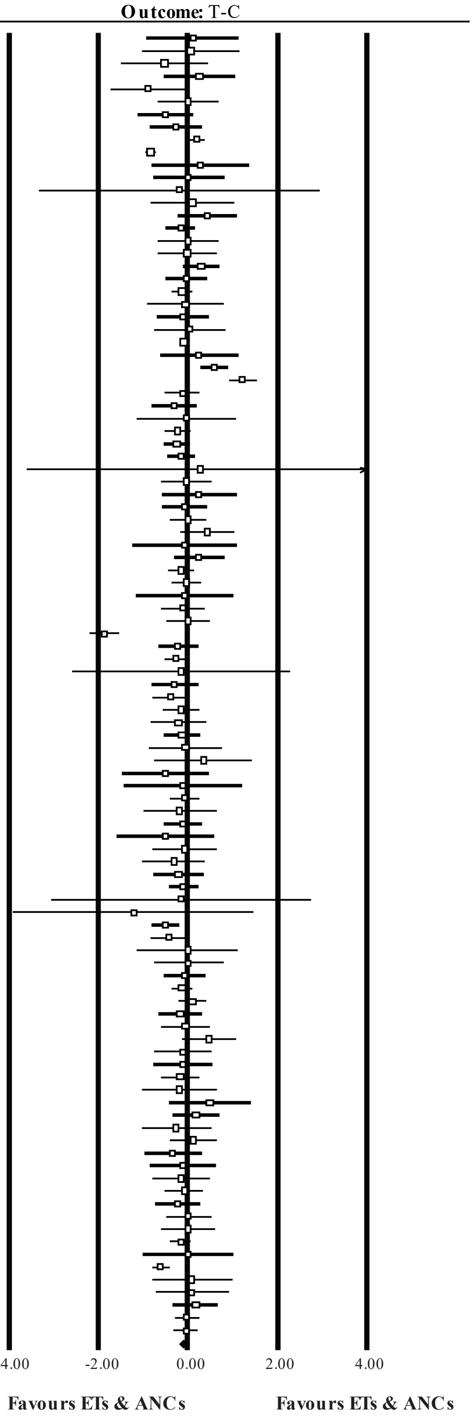

Test for overall effect Z=-2.417; *P*=0.013; Ns=1902; Nc=1771

Heterogeneity Tau^2=0.078; Q=349.4; df=102 (*P*=0.000); I^2=70.6

Supplementary Figure S9. Funnel plots and Egger statistics (intercept and 2-tailed *P*-value) of the meta-analysis evaluating the effects of a prolonged supplementation with ellagitannins and anthocyanins-containing products on Total Cholesterol (T-C).

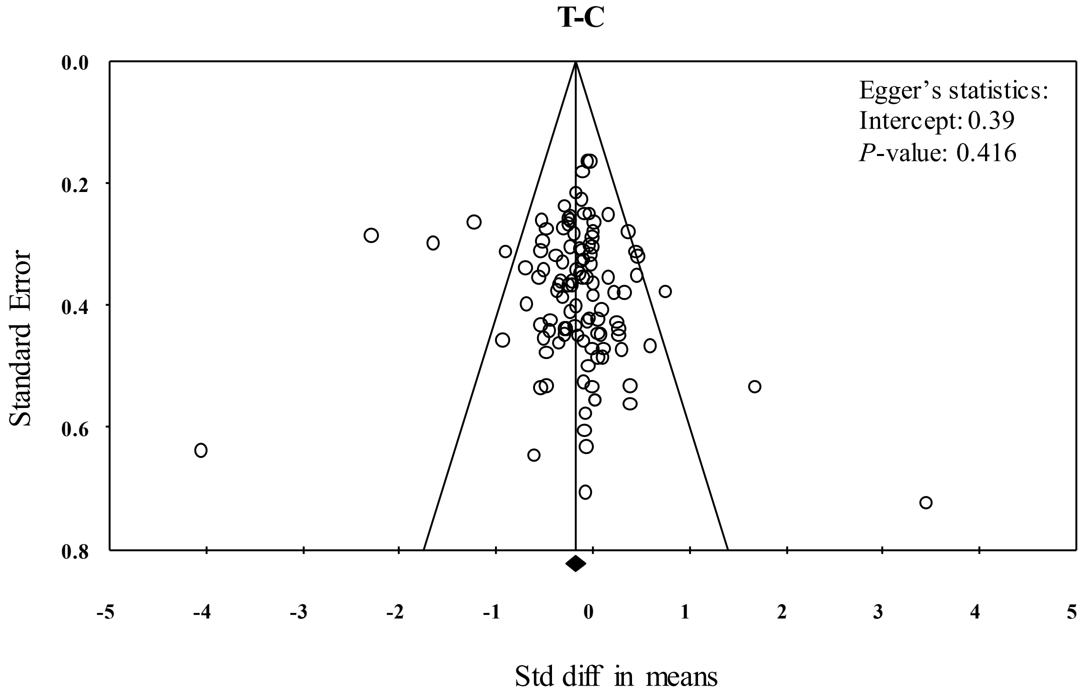

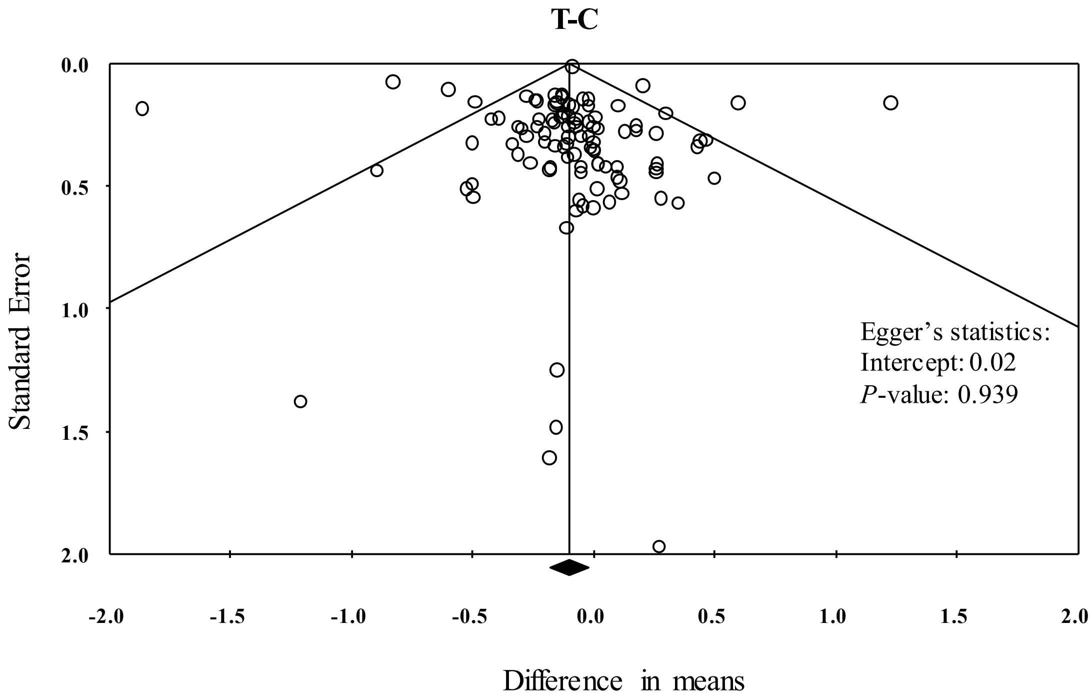

Supplementary Figure S10. Forest plot of the meta-analysis evaluating the effects of a prolonged supplementation with ellagitannins and anthocyanins-containing products on LDL-C. A total of 97 studies (displayed in alphabetical order) were analysed. Pooled results are shown at the bottom using a random-effects model. SDM: standardized difference in means, SE: standard error, 95% CI: lower and upper confidence limits for the average SDM, RW: relative weight.

First autor & year	SDM	SE	95%CI		RW
Abu-Amsha Caccetta 2001	0.088	0.486	-0.865	1.041	0.52
Abu-Amsha Caccetta 2001	-0.028	0.486	-0.980	0.925	0.52
Asgary 2013	-0.323	0.450	-1.206	0.560	0.61
Asgary 2014	0.074	0.437	-0.783	0.930	0.64
Banini 2006	0.373	0.441	-0.492	1.238	0.63
Basu 2010a	0.132	0.289	-0.435	0.699	1.47
Basu 2010b	-0.736	0.400	-1.520	0.048	0.77
Basu 2011	-0.338	0.362	-1.047	0.372	0.94
Basu 2014	0.539	0.372	-0.189	1.268	0.89
Basu 2014	3.589	0.590	2.433	4.746	0.35
Bommannan Santhakumar 2015	0.245	0.558	-0.849	1.340	0.39
Bommannan Santhakumar 2015	0.714	0.574	-0.411	1.838	0.37
Brennan 2010	-0.305	0.521	-1.325	0.715	0.45
Burton-Freeman 2010	0.017	0.408	-0.784	0.817	0.74
Caldu 1998	0.106	0.472	-0.819	1.030	0.55
Castilla 2006	-1.124	0.372	-1.854	-0.395	0.89
Castilla 2007	-1.027	0.535	-2.075	0.021	0.43
Cerdá 2006	0.111	0.365	-0.605	0.827	0.92
Clifton 2004	0.022	0.333	-0.631	0.675	1.11
Curtis 2009	-0.266	0.279	-0.812	0.280	1.59
Davinelli 2015	0.000	0.318	-0.623	0.623	1.22
Din 2011	-0.378	0.368	-1.100	0.344	0.91
Dohadwala 2011	-0.030	0.426	-0.865	0.806	0.68
Duthie 2006	-0.250	0.451	-1.134	0.635	0.60
Estruch 2011	0.119	0.448	-0.758	0.997	0.61
Foster 2012	-1.508	0.238	-1.974	-1.042	2.18
Gorinstein 2006	3.350	0.712	1.955	4.745	0.24
Gorinstein 2006	6.400	1.135	4.175	8.625	0.10
Gulati 2014	-0.463	0.262	-0.976	0.050	1.80
Guo 2014	-0.538	0.434	-1.388	0.313	0.65
Hansen 2005	0.191	0.339	-0.473	0.856	1.07
Hansen 2005	0.329	0.352	-0.361	1.018	0.99
Hansen 2005	-0.049	0.329	-0.694	0.596	1.14
Hassellund 2013	-0.374	0.389	-1.135	0.388	0.82
Heber 2007	0.271	0.425	-0.563	1.105	0.68
Heber 2007	-0.226	0.423	-1.056	0.604	0.69
Hijmering 2007	0.354	0.451	-0.530	1.238	0.60
Hokayem 2013	-0.456	0.329	-1.101	0.189	1.14
Hollis 2013	0.053	0.280	-0.496	0.602	1.57
Hosseini 2016	-0.457	0.313	-1.069	0.156	1.26
Huebbe 2012	-0.082	0.606	-1.269	1.106	0.34
Jenkins 2008	0.390	0.382	-0.358	1.138	0.85
Katz 2012	-0.037	0.408	-0.837	0.763	0.74
Kay 2002	-0.078	0.707	-1.464	1.309	0.25
Kianbakht 2014	-1.222	0.244	-1.700	-0.745	2.07
Lee 2014	-0.168	0.259	-0.675	0.339	1.84
Lekakis 2005	-0.092	0.251	-0.584	0.400	1.95
Li 2010	-0.006	0.272	-0.540	0.528	1.66
Ma 2010	-0.017	0.437	-0.873	0.840	0.64
Mukuddem-Petersen 2007	0.000	0.305	-0.598	0.598	1.32
Nigdikar 1998	0.180	0.528	-0.855	1.215	0.44
Nigdikar 1998	-0.023	0.527	-1.056	1.010	0.44
Nigdikar 1998	0.136	0.528	-0.899	1.170	0.44
Novotny 2015	0.264	0.269	-0.262	0.791	1.71
Park 2014	-0.117	0.228	-0.564	0.331	2.36
Pérez-Jímenez 2008	-0.225	0.376	-0.961	0.511	0.87
Preuss 2000	-0.049	0.460	-0.949	0.852	0.58
Preuss 2000	-0.268	0.462	-1.173	0.636	0.58
Puupponen-Pimiä 2013	-0.150	0.366	-0.867	0.566	0.92
Qin 2009	-0.516	0.186	-0.880	-0.152	3.57
Queipo-Ortuno 2012	-0.117	0.633	-1.358	1.123	0.31
Queipo-Ortuno 2012	-0.284	0.636	-1.530	0.961	0.30
Rahbar 2015	-0.955	0.315	-1.571	-0.338	1.24
Rahbar 2015	-0.576	0.298	-1.160	0.007	1.39
Razavi 2013	-0.851	0.343	-1.524	-0.178	1.04
Riso 2013	0.000	0.471	-0.924	0.924	0.55
Santhakumar 2015	0.470	0.564	-0.635	1.575	0.39
Shema-Didi 2014	0.133	0.251	-0.359	0.625	1.95
Sivaprakasapillai 2010	-1.186	0.511	-2.187	-0.184	0.47
Sivaprakasapillai 2010	0.255	0.473	-0.673	1.183	0.55
Spaccarotella 2008	-0.216	0.438	-1.074	0.643	0.64
Stull 2010	-0.027	0.354	-0.722	0.667	0.98
Sumner 2005	-0.345	0.319	-0.971	0.281	1.21
Tey 2013	-0.012	0.348	-0.694	0.671	1.01
Tey 2013	-0.008	0.329	-0.653	0.637	1.14
Tomé-Carneiro 2012	-0.133	0.283	-0.688	0.422	1.53
Tome-Carneiro 2013	-0.096	0.434	-0.947	0.754	0.65
Tsang 2005	0.209	0.458	-0.688	1.106	0.59
Tsang 2012	0.013	0.378	-0.728	0.754	0.86
Umar 2005	-0.202	0.448	-1.081	0.677	0.61
Vaisman 2015	0.181	0.355	-0.515	0.876	0.98
Vaisman 2015	0.092	0.422	-0.735	0.919	0.69
Velliquette 2015	-0.108	0.355	-0.803	0.587	0.98
Velliquette 2015	-0.007	0.343	-0.680	0.665	1.05
Vidlar 2010	0.953	0.330	0.307	1.600	1.13
Wang 2012	0.346	0.262	-0.169	0.860	1.79
Wang 2012	0.012	0.265	-0.508	0.532	1.75
Wien 2010	-0.543	0.278	-1.087	0.002	1.59
Wu 2014	-0.541	0.322	-1.172	0.090	1.19
Wu 2015	0.092	0.385	-0.664	0.847	0.83
Yubero 2013	-0.886	0.273	-1.422	-0.350	1.65
Zern 2005	0.112	0.430	-0.731	0.954	0.67
Zhang 2015	0.147	0.253	-0.350	0.643	1.92
Zhu 2011	-0.788	0.172	-1.125	-0.451	4.17
Zhu 2013	-0.769	0.172	-1.105	-0.433	4.18
Zunino 2014	-0.009	0.408	-0.809	0.791	0.74
**Intervention effect**	**-0.099**	**0.061**	**-0.220**	**0.021**	

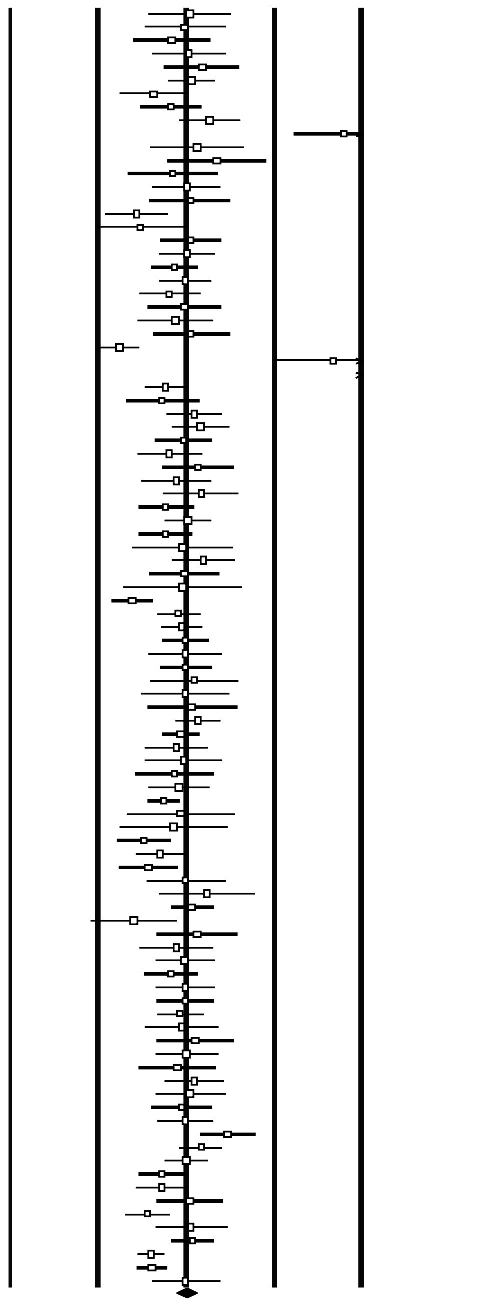

Test for overall effect Z=-1.620; *P*=0.105; Ns=1811; Nc=1681

Heterogeneity Tau^2=0.217; Q=270.6; df=96 (*P*=0.000); I^2=64.5

Supplementary Figure S11.- Forest plot of the meta-analysis evaluating the effects of a prolonged supplementation with ellagitannins and anthocyanins-containing products on LDL-C. A total of 92 studies (displayed in alphabetical order) were analysed. Pooled results are shown at the bottom using a random-effects model. DM: Difference in means, SE: standard error, 95% CI: lower and upper confidence limits for the average DM, RW: relative weight.

First autor & year	DM	SE	95% CI		RW
Abu-Amsha Caccetta 2001	0.090	0.481	-0.852	1.032	0.06
Abu-Amsha Caccetta 2001	-0.030	0.513	-1.036	0.976	0.05
Asgary 2013	-0.381	0.575	-1.509	0.747	0.04
Asgary 2014	0.049	0.294	-0.528	0.626	0.16
Banini 2006	0.179	0.200	-0.212	0.571	0.35
Basu 2010a	0.100	0.218	-0.328	0.528	0.29
Basu 2010b	-0.400	0.305	-0.998	0.198	0.15
Basu 2011	-0.239	0.249	-0.726	0.248	0.23
Basu 2014	0.133	0.083	-0.031	0.297	2.00
Basu 2014	0.644	0.070	0.506	0.781	2.84
Bommannan Santhakumar 2015	0.270	0.592	-0.890	1.430	0.04
Bommannan Santhakumar 2015	0.860	0.615	-0.345	2.065	0.04
Brennan 2010	-0.150	0.398	-0.930	0.629	0.09
Burton-Freeman 2010	0.091	1.317	-2.490	2.671	0.01
Caldu 1998	0.090	0.425	-0.742	0.923	0.08
Castilla 2006	-1.038	0.327	-1.679	-0.397	0.13
Castilla 2007	-0.320	0.149	-0.613	-0.027	0.63
Cerdá 2006	0.091	0.316	-0.530	0.711	0.14
Clifton 2004	0.020	0.317	-0.601	0.641	0.14
Curtis 2009	-0.200	0.191	-0.575	0.175	0.38
Davinelli 2015	0.000	0.274	-0.536	0.536	0.19
Din 2011	-0.160	0.154	-0.463	0.143	0.59
Dohadwala 2011	-0.026	0.382	-0.774	0.722	0.10
Duthie 2006	-0.160	0.297	-0.742	0.422	0.16
Estruch 2011	0.106	0.391	-0.661	0.873	0.09
Foster 2012	-0.111	0.015	-0.141	-0.081	60.03
Gorinstein 2006	0.670	0.092	0.490	0.850	1.65
Gorinstein 2006	1.280	0.092	1.100	1.460	1.65
Gulati 2014	-0.162	0.152	-0.461	0.137	0.60
Guo 2014	-0.218	0.175	-0.561	0.126	0.45
Hansen 2005	0.070	0.124	-0.173	0.313	0.91
Hansen 2005	0.140	0.149	-0.152	0.432	0.63
Hansen 2005	-0.020	0.135	-0.284	0.244	0.77
Heber 2007	0.222	0.335	-0.434	0.879	0.12
Heber 2007	-0.182	0.348	-0.863	0.500	0.12
Hijmering 2007	0.220	0.285	-0.338	0.778	0.17
Hokayem 2013	-0.080	0.054	-0.186	0.026	4.79
Hollis 2013	0.040	0.217	-0.386	0.466	0.30
Hosseini 2016	-0.324	0.235	-0.786	0.137	0.25
Huebbe 2012	-0.060	0.476	-0.993	0.873	0.06
Jenkins 2008	0.240	0.243	-0.237	0.717	0.24
Katz 2012	-0.021	0.233	-0.478	0.436	0.26
Kay 2002	-0.050	0.446	-0.924	0.824	0.07
Kianbakht 2014	-1.127	0.224	-1.567	-0.688	0.28
Lee 2014	-0.090	0.138	-0.360	0.180	0.74
Lekakis 2005	-0.270	0.665	-1.573	1.033	0.03
Li 2010	-0.003	0.290	-0.571	0.565	0.17
Ma 2010	-0.007	0.188	-0.375	0.360	0.40
Nigdikar 1998	0.160	0.504	-0.827	1.147	0.06
Nigdikar 1998	-0.020	0.484	-0.969	0.929	0.06
Nigdikar 1998	0.140	0.545	-0.928	1.208	0.05
Novotny 2015	0.070	0.135	-0.195	0.335	0.77
Park 2014	-0.057	0.112	-0.277	0.163	1.11
Pérez-Jímenez 2008	-0.287	0.499	-1.265	0.690	0.06
Preuss 2000	-0.048	0.423	-0.877	0.781	0.08
Preuss 2000	-0.180	0.292	-0.753	0.393	0.16
Puupponen-Pimiä 2013	-0.100	0.252	-0.593	0.393	0.22
Qin 2009	-0.492	0.174	-0.834	-0.151	0.46
Queipo-Ortuno 2012	-0.083	0.619	-1.296	1.130	0.04
Queipo-Ortuno 2012	-0.191	0.614	-1.396	1.013	0.04
Rahbar 2015	-0.767	0.225	-1.208	-0.326	0.28
Rahbar 2015	-0.477	0.248	-0.964	0.010	0.23
Riso 2013	0.000	0.489	-0.958	0.958	0.06
Santhakumar 2015	0.860	0.883	-0.871	2.591	0.02
Shema-Didi 2014	0.110	0.207	-0.297	0.516	0.32
Sivaprakasapillai 2010	-0.300	0.114	-0.522	-0.077	1.08
Sivaprakasapillai 2010	0.078	0.150	-0.216	0.371	0.62
Spaccarotella 2008	-0.130	0.264	-0.647	0.387	0.20
Stull 2010	-0.020	0.270	-0.549	0.509	0.19
Sumner 2005	-0.310	0.282	-0.863	0.242	0.18
Tey 2013	-0.160	4.696	-9.365	9.045	0.00
Tey 2013	-0.110	4.428	-8.789	8.569	0.00
Tomé-Carneiro 2012	-0.085	0.173	-0.424	0.253	0.47
Tome-Carneiro 2013	-0.079	0.368	-0.799	0.642	0.10
Tsang 2005	0.200	0.406	-0.597	0.997	0.08
Tsang 2012	0.010	0.277	-0.533	0.553	0.18
Umar 2005	-0.140	0.309	-0.745	0.465	0.15
Vaisman 2015	0.131	0.253	-0.364	0.626	0.22
Vaisman 2015	0.063	0.298	-0.520	0.647	0.16
Velliquette 2015	-0.103	0.368	-0.824	0.617	0.10
Velliquette 2015	-0.008	0.348	-0.690	0.674	0.12
Vidlar 2010	0.580	0.197	0.194	0.966	0.36
Wang 2012	0.270	0.207	-0.136	0.676	0.33
Wang 2012	0.010	0.217	-0.416	0.436	0.30
Wu 2014	-0.138	0.081	-0.296	0.020	2.14
Wu 2015	0.152	0.573	-0.970	1.275	0.04
Yubero 2013	-0.404	0.130	-0.660	-0.149	0.82
Zern 2005	0.100	0.368	-0.622	0.822	0.10
Zhang 2015	0.110	0.178	-0.239	0.459	0.44
Zhu 2011	-0.356	0.084	-0.520	-0.191	1.98
Zhu 2013	-0.360	0.084	-0.524	-0.196	1.98
**Intervention effect**	**-0.032**	**0.043**	**-0.117**	**0.052**	

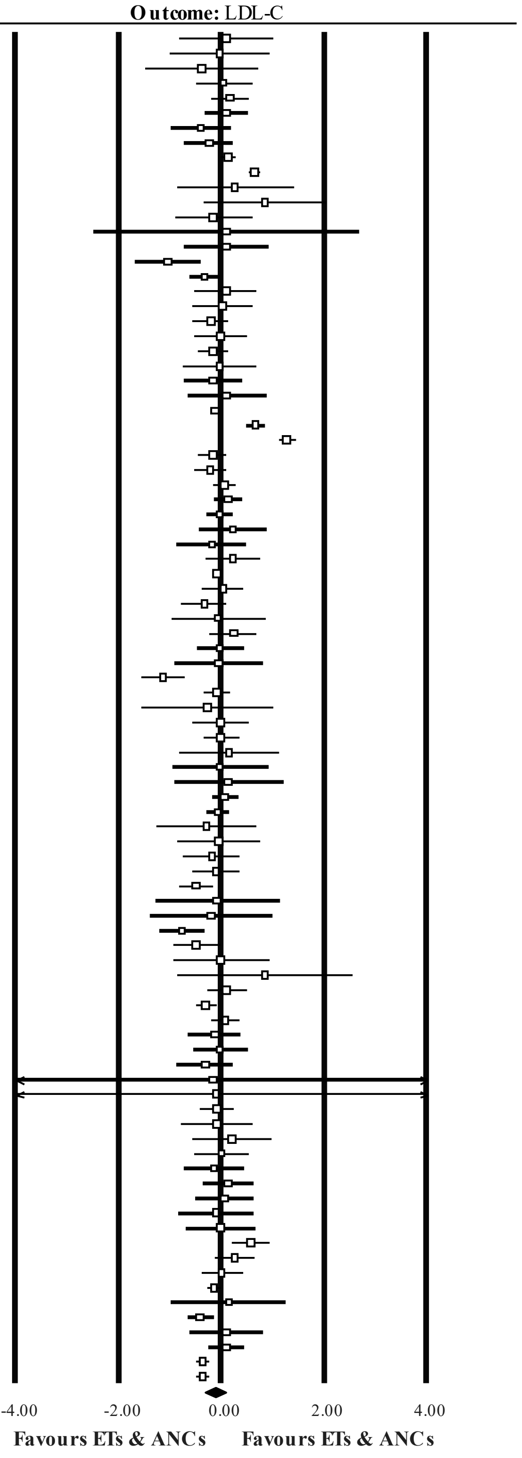

Test for overall effect Z=-0.748; *P*=0.454; Ns=1718; Nc=1589

Heterogeneity Tau^2=91.0; Q=523.8; df=91 (*P*=0.000); I^2=82.6

Supplementary Figure S12. Funnel plots and Egger statistics (intercept and 2-tailed *P*-value) of the meta-analysis evaluating the effects of a prolonged supplementation with ellagitannins and anthocyanins-containing products on low density lipoprotein- cholesterol (LDL-C).

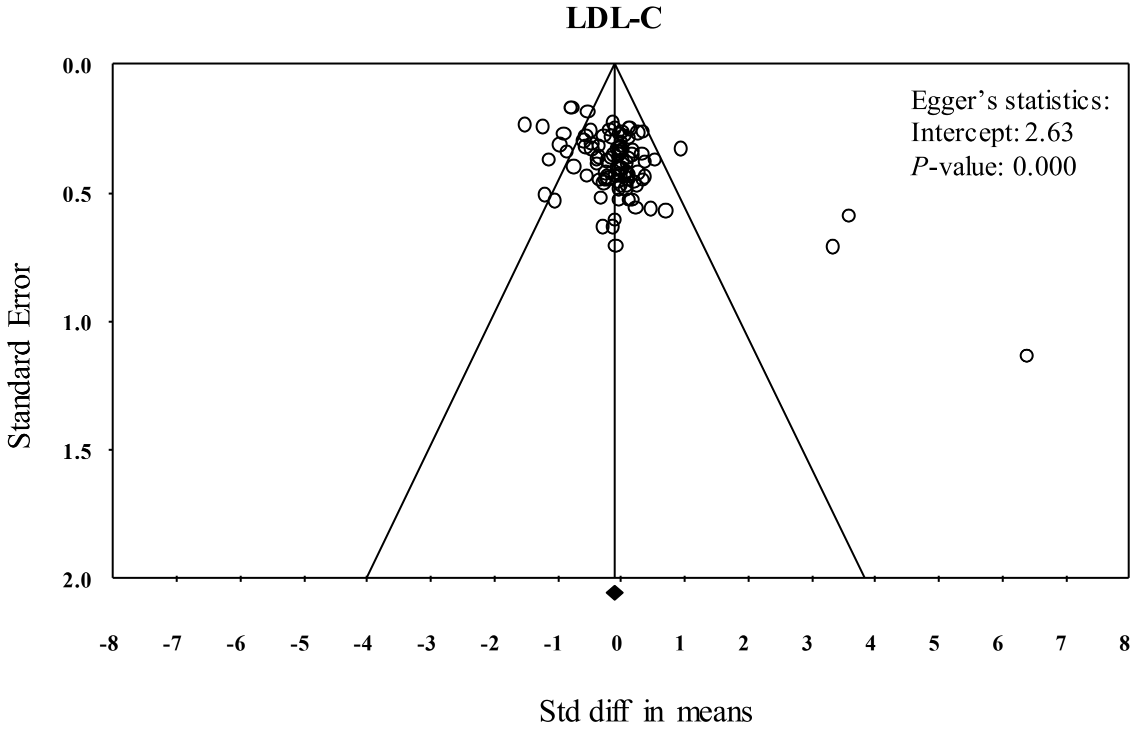

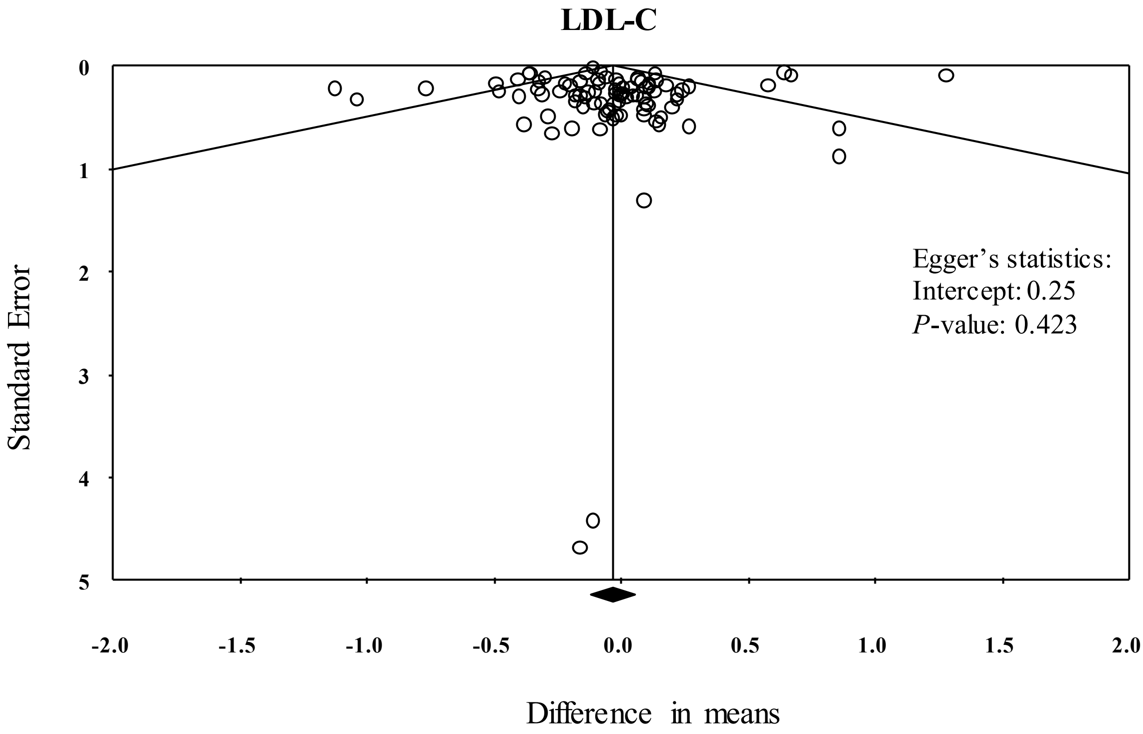

Supplementary Figure S13. Forest plot of the meta-analysis evaluating the effects of a prolonged supplementation with ellagitannins and anthocyanins-containing products on HDL-C. A total of 99 studies (displayed in alphabetical order) were analysed. Pooled results are shown at the bottom using a random-effects model. SDM: standardized difference in means, SE: standard error, 95% CI: lower and upper confidence limits for the average SDM, RW: relative weight.

First autor & year	SDM	SE	95% CI		RW
Abu-Amsha Caccetta 2001	-0.359	0.490	-1.319	0.601	-0.77
Abu-Amsha Caccetta 2001	-0.252	0.488	-1.208	0.704	-0.60
Asgary 2013	0.049	0.449	-0.831	0.928	-0.11
Asgary 2014	0.021	0.437	-0.835	0.878	-0.16
Banini 2006	-0.051	0.438	-0.910	0.807	-0.29
Basu 2010a	0.000	0.289	-0.566	0.566	-0.24
Basu 2010b	0.676	0.398	-0.104	1.456	1.05
Basu 2011	0.135	0.360	-0.571	0.840	0.05
Basu 2014	0.250	0.367	-0.468	0.968	0.27
Basu 2014	-1.333	0.404	-2.125	-0.542	-2.67
Bommannan Santhakumar 2015	0.170	0.557	-0.923	1.262	0.09
Bommannan Santhakumar 2015	0.430	0.563	-0.673	1.533	0.48
Burton-Freeman 2010	-0.739	0.422	-1.567	0.089	-1.53
Caldu 1998	0.181	0.472	-0.744	1.107	0.12
Castilla 2006	0.388	0.352	-0.301	1.078	0.55
Castilla 2007	0.003	0.500	-0.977	0.983	-0.18
Cerdá 2006	-0.139	0.366	-0.856	0.577	-0.49
Clifton 2004	0.094	0.334	-0.560	0.747	-0.04
Curtis 2009	0.000	0.277	-0.544	0.544	-0.24
Davinelli 2015	-0.025	0.318	-0.647	0.598	-0.28
Din 2011	-0.058	0.365	-0.774	0.657	-0.33
Dohadwala 2011	-0.222	0.428	-1.061	0.616	-0.60
Duthie 2010	-0.496	0.471	-1.419	0.428	-1.02
Erlund 2008	0.673	0.244	0.195	1.151	1.29
Estruch 2011	0.007	0.447	-0.870	0.883	-0.18
Flammer 2013	0.339	0.217	-0.087	0.765	0.54
Foster 2012	0.909	0.222	0.474	1.344	1.88
Fuhrman 1995	0.366	0.490	-0.595	1.326	0.42
Goldberg 1996	0.042	0.426	-0.794	0.878	-0.12
Gorinstein 2006	1.000	0.487	0.045	1.955	1.47
Gorinstein 2006	1.600	0.528	0.566	2.634	2.33
Gulati 2014	0.556	0.263	0.040	1.071	0.99
Guo 2014	0.311	0.429	-0.529	1.152	0.36
Hansen 2005	1.315	0.373	0.584	2.046	2.32
Hansen 2005	-0.179	0.350	-0.866	0.508	-0.58
Hansen 2005	-0.308	0.331	-0.957	0.340	-0.86
Hassellund 2013	0.821	0.401	0.035	1.607	1.32
Heber 2007	-0.129	0.424	-0.961	0.703	-0.43
Heber 2007	-0.227	0.423	-1.057	0.603	-0.61
Hijmering 2007	0.274	0.450	-0.608	1.156	0.28
Hokayem 2013	-0.086	0.325	-0.723	0.552	-0.41
Hollis 2013	0.032	0.280	-0.517	0.581	-0.17
Hosseini 2016	0.398	0.312	-0.213	1.009	0.60
Huebbe 2012	-0.138	0.606	-1.326	1.051	-0.35
Katz 2012	-0.016	0.408	-0.816	0.784	-0.23
Kay 2002	-0.177	0.708	-1.565	1.212	-0.36
Kianbakht 2014	2.237	0.285	1.679	2.796	4.62
Lee 2014	0.250	0.259	-0.258	0.758	0.31
Lekakis 2005	0.394	0.254	-0.103	0.891	0.64
Li 2010	-0.091	0.272	-0.625	0.443	-0.45
Ma 2010	-0.378	0.441	-1.242	0.486	-0.86
Mukuddem-Petersen 2007	-0.498	0.310	-1.106	0.109	-1.28
Nigdikar 1998	0.651	0.540	-0.408	1.710	0.83
Nigdikar 1998	0.533	0.536	-0.517	1.583	0.65
Nigdikar 1998	0.419	0.533	-0.625	1.463	0.48
Novotny 2015	0.094	0.268	-0.430	0.619	-0.04
Park 2014	-0.294	0.229	-0.743	0.156	-0.95
Pérez-Jímenez 2008	-0.212	0.376	-0.948	0.524	-0.62
Preuss 2000	-0.098	0.460	-0.999	0.804	-0.36
Preuss 2000	0.193	0.461	-0.710	1.095	0.14
Puupponen-Pimiä 2013	-0.374	0.368	-1.095	0.348	-0.94
Qin 2009	0.477	0.185	0.115	0.840	0.90
Queipo-Ortuno 2012	-0.208	0.634	-1.451	1.035	-0.44
Queipo-Ortuno 2012	-0.168	0.634	-1.410	1.073	-0.38
Rahbar 2015	-0.032	0.298	-0.616	0.553	-0.31
Rahbar 2015	-0.081	0.292	-0.653	0.492	-0.41
Razavi 2013	0.095	0.329	-0.550	0.740	-0.03
Riso 2013	0.000	0.471	-0.924	0.924	-0.19
Santhakumar 2015	0.430	0.563	-0.673	1.533	0.48
Shema-Didi 2014	0.352	0.253	-0.143	0.847	0.55
Sivaprakasapillai 2010	-0.283	0.474	-1.211	0.646	-0.66
Sivaprakasapillai 2010	-1.000	0.500	-1.980	-0.020	-1.80
Spaccarotella 2008	-0.054	0.437	-0.910	0.803	-0.29
Stull 2010	-0.083	0.354	-0.778	0.611	-0.38
Sumner 2005	-0.000	0.317	-0.621	0.621	-0.23
Tey 2013	0.065	0.348	-0.618	0.748	-0.09
Tey 2013	0.059	0.329	-0.585	0.704	-0.11
Tomé-Carneiro 2012	0.035	0.283	-0.520	0.589	-0.17
Tome-Carneiro 2013	-0.287	0.436	-1.142	0.567	-0.71
Tsang 2006	0.309	0.459	-0.590	1.209	0.34
Tsang 2012	0.119	0.378	-0.622	0.861	0.01
Umar 2005	-0.421	0.452	-1.307	0.465	-0.92
Urquiaga 2015	0.116	0.342	-0.555	0.786	0.01
Vaisman 2015	0.070	0.422	-0.756	0.897	-0.07
Vaisman 2015	-0.734	0.366	-1.451	-0.016	-1.65
Velliquette 2015	-0.061	0.343	-0.734	0.611	-0.35
Velliquette 2015	-0.047	0.354	-0.741	0.648	-0.31
Vidlar 2010	-1.149	0.334	-1.805	-0.494	-2.56
Whelan 2004	0.000	0.535	-1.048	1.048	-0.17
Wien 2010	0.117	0.273	-0.418	0.653	0.01
Wu 2014	0.316	0.318	-0.307	0.940	0.43
Wu 2015	-0.064	0.385	-0.819	0.691	-0.33
Yubero 2013	0.145	0.259	-0.362	0.652	0.08
Zern 2005	0.000	0.447	-0.877	0.877	-0.19
Zern 2005	0.000	0.408	-0.800	0.800	-0.20
Zhang 2015	0.226	0.254	-0.271	0.723	0.26
Zhu 2011	0.724	0.171	0.389	1.059	1.53
Zhu 2013	0.761	0.171	0.425	1.097	1.63
Zunino 2014	-0.223	0.410	-1.026	0.579	-0.61
**Intervention effect**	**0.111**	**0.052**	**0.009**	**0.214**	

Outcome: HDL-C

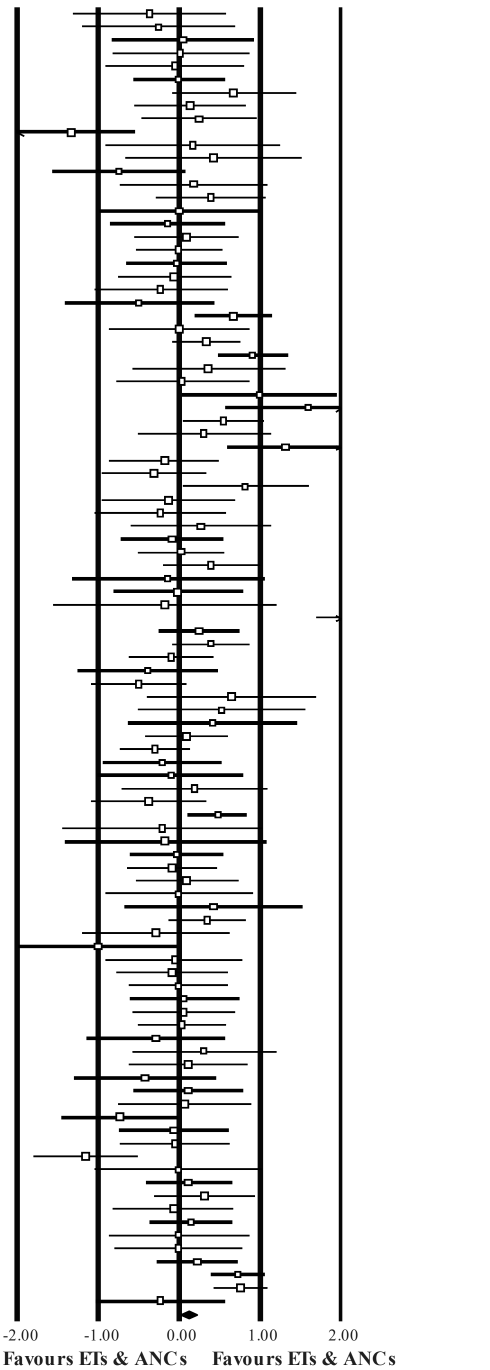

Test for overall effect Z=2.124; P=0.034; Ns=1858; Nc=1723

Heterogeneity Tau^2=0.133; Q=208.9; df=98 (P=0.000); I^2=53.1

Supplementary Figure S14.- Forest plot of the meta-analysis evaluating the effects of a prolonged supplementation with ellagitannins and anthocyanins-containing products on HDL-C. A total of 92 studies (displayed in alphabetical order) were analysed. Pooled results are shown at the bottom using a random-effects model. DM: Difference in means, SE: standard error, 95% CI: lower and upper confidence limits for the average DM, RW: relative weight.

First autor & year	DM	SE	95%CI		RW
Abu-Amsha Caccetta 2001	-0.090	0.127	0.160	-0.340	0.79
Abu-Amsha Caccetta 2001	-0.080	0.142	0.199	-0.359	0.68
Asgary 2013	0.019	0.151	0.315	-0.277	0.62
Asgary 2014	0.004	0.083	0.166	-0.158	1.29
Banini 2006	-0.018	0.156	0.287	-0.323	0.59
Basu 2010a	0.000	0.028	0.056	-0.056	2.17
Basu 2010b	0.100	0.125	0.345	-0.145	0.81
Basu 2011	0.034	0.082	0.195	-0.128	1.29
Basu 2014	0.026	0.036	0.096	-0.045	2.06
Basu 2014	-0.103	0.031	-0.042	-0.165	2.13
Bommannan Santhakumar 2015	0.090	0.557	1.182	-1.002	0.06
Bommannan Santhakumar 2015	0.410	0.479	1.348	-0.528	0.08
Burton-Freeman 2010	-0.027	2.754	5.371	-5.425	0.00
Caldu 1998	0.046	0.119	0.278	-0.186	0.86
Castilla 2006	0.112	0.090	0.288	-0.064	1.19
Castilla 2007	0.001	0.156	0.306	-0.304	0.59
Cerdá 2006	-0.061	0.169	0.270	-0.393	0.52
Clifton 2004	0.030	0.105	0.236	-0.176	1.00
Curtis 2009	0.000	0.098	0.192	-0.192	1.08
Davinelli 2015	-0.010	0.120	0.226	-0.246	0.85
Din 2011	-0.010	0.063	0.113	-0.133	1.60
Dohadwala 2011	-0.052	0.102	0.149	-0.252	1.03
Duthie 2010	-0.140	0.121	0.098	-0.378	0.84
Estruch 2011	0.003	0.167	0.329	-0.324	0.53
Foster 2012	0.200	0.118	0.430	-0.031	0.87
Fuhrman 1995	0.260	0.343	0.932	-0.412	0.15
Goldberg 1996	0.010	0.129	0.263	-0.243	0.77
Gorinstein 2006	0.100	0.046	0.190	0.010	1.89
Gorinstein 2006	0.160	0.046	0.250	0.070	1.89
Gulati 2014	0.078	0.036	0.149	0.006	2.05
Guo 2014	0.106	0.129	0.360	-0.148	0.77
Hansen 2005	0.200	0.051	0.301	0.099	1.79
Hansen 2005	-0.050	0.098	0.141	-0.241	1.09
Hansen 2005	-0.050	0.053	0.055	-0.155	1.76
Heber 2007	-0.028	0.106	0.180	-0.237	0.99
Heber 2007	-0.060	0.108	0.151	-0.272	0.97
Hijmering 2007	0.090	0.156	0.396	-0.216	0.59
Hokayem 2013	-0.030	0.116	0.197	-0.257	0.89
Hollis 2013	0.010	0.090	0.186	-0.166	1.19
Hosseini 2016	0.098	0.076	0.247	-0.051	1.39
Huebbe 2012	-0.030	0.143	0.250	-0.310	0.67
Katz 2012	-0.003	0.067	0.129	-0.134	1.53
Kay 2002	-0.040	0.152	0.259	-0.339	0.61
Kianbakht 2014	0.384	0.034	0.452	0.317	2.08
Lee 2014	0.037	0.038	0.112	-0.038	2.02
Lekakis 2005	0.230	0.144	0.513	-0.053	0.66
Li 2010	-0.031	0.088	0.141	-0.203	1.21
Ma 2010	-0.067	0.078	0.085	-0.220	1.36
Nigdikar 1998	0.140	0.132	0.398	-0.118	0.75
Nigdikar 1998	0.130	0.124	0.374	-0.114	0.81
Nigdikar 1998	0.090	0.106	0.297	-0.117	1.00
Novotny 2015	0.010	0.075	0.158	-0.138	1.40
Park 2014	-0.093	0.072	0.049	-0.235	1.44
Pérez-Jímenez 2008	-0.065	0.119	0.169	-0.299	0.86
Preuss 2000	-0.041	0.188	0.327	-0.410	0.44
Preuss 2000	0.039	0.089	0.213	-0.135	1.20
Puupponen-Pimiä 2013	-0.100	0.105	0.106	-0.306	1.00
Qin 2009	0.116	0.044	0.202	0.031	1.93
Queipo-Ortuno 2012	-0.091	0.274	0.447	-0.628	0.23
Queipo-Ortuno 2012	-0.067	0.264	0.450	-0.584	0.25
Rahbar 2015	-0.007	0.065	0.121	-0.135	1.56
Rahbar 2015	-0.020	0.074	0.125	-0.164	1.42
Riso 2013	0.000	0.133	0.261	-0.261	0.74
Santhakumar 2015	0.410	0.479	1.348	-0.528	0.08
Shema-Didi 2014	0.110	0.072	0.252	-0.031	1.44
Sivaprakasapillai 2010	-0.026	0.041	0.055	-0.107	1.97
Sivaprakasapillai 2010	-0.078	0.034	-0.010	-0.145	2.08
Spaccarotella 2008	-0.010	0.081	0.149	-0.169	1.31
Stull 2010	-0.020	0.098	0.173	-0.213	1.08
Sumner 2005	-0.000	0.099	0.195	-0.195	1.07
Tey 2013	0.020	0.101	0.219	-0.179	1.04
Tey 2013	0.020	0.106	0.228	-0.188	0.99
Tomé-Carneiro 2012	0.010	0.087	0.180	-0.160	1.23
Tome-Carneiro 2013	-0.052	0.084	0.113	-0.216	1.27
Tsang 2006	0.100	0.148	0.389	-0.189	0.64
Tsang 2012	0.060	0.191	0.435	-0.315	0.43
Umar 2005	-0.100	0.113	0.122	-0.322	0.91
Urquiaga 2015	0.036	0.101	0.233	-0.161	1.05
Vaisman 2015	0.023	0.152	0.320	-0.274	0.61
Vaisman 2015	-0.240	0.148	0.049	-0.530	0.64
Velliquette 2015	-0.031	0.166	0.294	-0.356	0.54
Velliquette 2015	-0.023	0.171	0.312	-0.358	0.51
Vidlar 2010	-0.145	0.052	-0.044	-0.246	1.79
Whelan 2004	0.000	0.107	0.210	-0.210	0.98
Wu 2014	0.026	0.026	0.077	-0.025	2.20
Wu 2015	-0.036	0.201	0.359	-0.431	0.39
Yubero 2013	0.033	0.059	0.148	-0.082	1.67
Zern 2005	0.000	0.158	0.309	-0.309	0.58
Zern 2005	0.000	0.091	0.178	-0.178	1.17
Zhang 2015	0.070	0.074	0.215	-0.075	1.42
Zhu 2011	0.171	0.038	0.245	0.096	2.02
Zhu 2013	0.160	0.036	0.230	0.090	2.06
**Intervention effect**	**0.026**	**0.014**	**-0.001**	**0.053**	

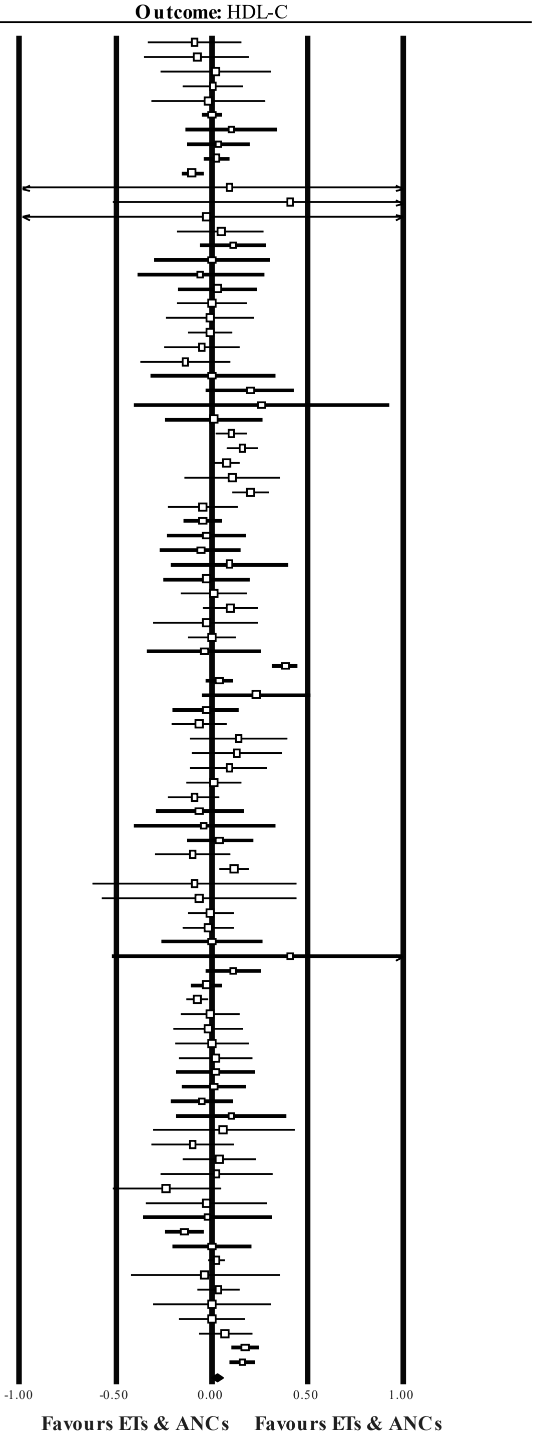

Test for overall effect Z=1.867; *P*=0.062; Ns=1692; Nc=1547

Heterogeneity Tau^2=0.008; Q=237.2; df=91 (*P*=0.000); I^2=61.6

Supplementary Figure S15. Funnel plots and Egger statistics (intercept and 2-tailed *P*-value) of the meta-analysis evaluating the effects of a prolonged supplementation with ellagitannins and anthocyanins-containing products on high density lipoprotein- cholesterol (HDL-C).

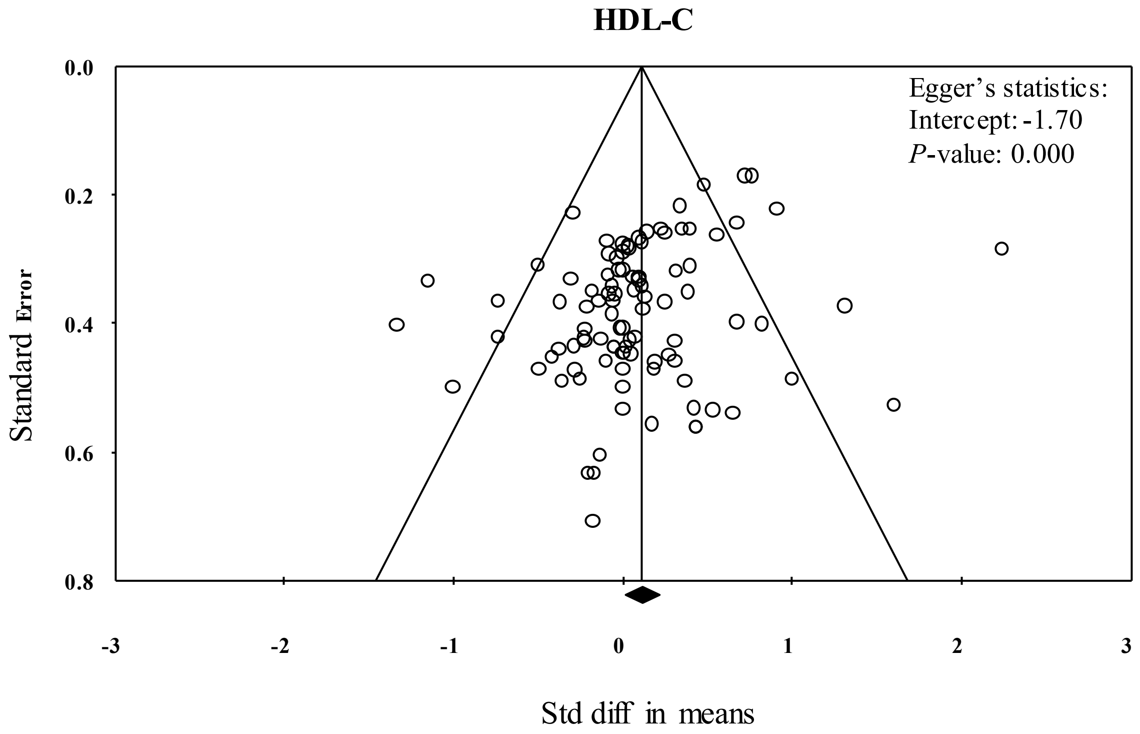

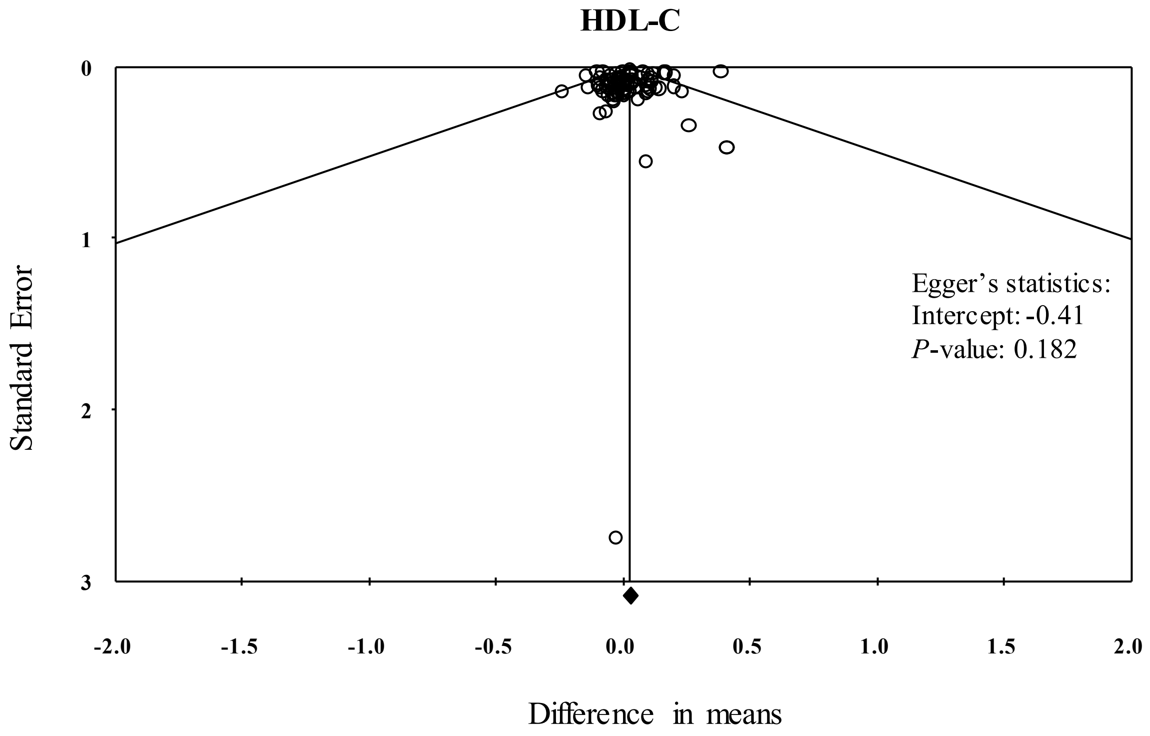

Supplementary Figure S16. Forest plot of the meta-analysis evaluating the effects of a prolonged supplementation with ellagitannins and anthocyanins-containing products on TAGs. A total of 97 studies (displayed in alphabetical order) were analysed. Pooled results are shown at the bottom using a random-effects model. SDM: standardized difference in means, SE: standard error, 95% CI: lower and upper confidence limits for the average SDM, RW: relative weight.

First autor & year	SDM	SE	95% CI		RW
Abu-Amsha Caccetta 2001	0.296	0.489	-0.661	1.254	0.49
Abu-Amsha Caccetta 2001	0.098	0.486	-0.855	1.050	0.50
Asgary 2013	0.329	0.470	-0.592	1.251	0.53
Asgary 2014	0.174	0.438	-0.684	1.032	0.61
Banini 2006	-0.140	0.438	-0.999	0.719	0.61
Basu 2010a	-0.132	0.289	-0.699	0.435	1.40
Basu 2010b	-0.068	0.387	-0.827	0.692	0.78
Basu 2011	0.896	0.377	0.157	1.635	0.83
Basu 2014	-1.345	0.404	-2.137	-0.552	0.72
Basu 2014	-0.142	0.366	-0.859	0.574	0.88
Bommannan Santhakumar 2015	0.658	0.571	-0.462	1.777	0.36
Bommannan Santhakumar 2015	-0.361	0.561	-1.460	0.738	0.37
Burton-Freeman 2010	2.523	0.547	1.451	3.595	0.39
Caldu 1998	0.109	0.472	-0.816	1.033	0.53
Castilla 2006	-0.085	0.349	-0.769	0.599	0.96
Castilla 2007	-0.469	0.532	-1.512	0.573	0.41
Cerdá 2006	0.043	0.365	-0.672	0.759	0.88
Clifton 2004	-0.041	0.333	-0.694	0.612	1.06
Curtis 2007	-0.175	0.278	-0.720	0.369	1.52
Davinelli 2015	-1.596	0.362	-2.306	-0.886	0.89
Din 2011	0.136	0.366	-0.581	0.852	0.88
Dohadwala 2011	-0.051	0.426	-0.887	0.785	0.64
Duthie 2010	0.397	0.455	-0.495	1.290	0.57
Erlund 2008	-0.376	0.239	-0.845	0.094	2.05
Estruch 2011	0.140	0.448	-0.738	1.018	0.58
Flammer 2013	-0.370	0.218	-0.796	0.056	2.48
Foster 2012	-1.939	0.262	-2.451	-1.426	1.71
Goldberg 1996	0.367	0.430	-0.476	1.210	0.63
Gorinstein 2006	1.300	0.506	0.309	2.291	0.46
Gorinstein 2006	6.300	1.121	4.104	8.496	0.09
Gulati 2014	-0.874	0.270	-1.404	-0.344	1.61
Guo 2014	0.084	0.427	-0.752	0.920	0.64
Haddad 2014	-0.212	0.501	-1.195	0.771	0.47
Hassellund 2013	-0.608	0.394	-1.380	0.164	0.76
Heber 2007	-0.331	0.426	-1.166	0.504	0.65
Heber 2007	-0.003	0.423	-0.832	0.825	0.66
Hijmering 2007	-0.025	0.448	-0.902	0.853	0.58
Hokayem 2013	0.276	0.326	-0.364	0.916	1.10
Hollis 2013	0.055	0.280	-0.494	0.604	1.49
Hosseini 2016	-0.420	0.312	-1.032	0.191	1.20
Jenkins 2008	0.055	0.378	-0.686	0.796	0.82
Jin 2011	0.015	0.447	-0.861	0.892	0.59
Karlsen 2010	0.280	0.255	-0.221	0.780	1.80
Katz 2012	-0.202	0.409	-1.005	0.600	0.70
Kay 2002	0.253	0.710	-1.138	1.645	0.23
Kianbakht 2014	-1.003	0.237	-1.468	-0.538	2.08
Lee 2014	-0.270	0.259	-0.778	0.238	1.74
Lekakis 2005	-0.115	0.251	-0.608	0.377	1.86
Li 2010	-0.410	0.275	-0.949	0.129	1.55
Ma 2010	-0.221	0.438	-1.080	0.638	0.61
Mukuddem-Petersen 2007	-0.233	0.306	-0.833	0.367	1.25
Naissides 2006	0.239	0.367	-0.481	0.959	0.87
Naissides 2006	-0.021	0.359	-0.726	0.683	0.91
Natella 2011	1.074	0.618	-0.136	2.285	0.31
Novotny 2015	-0.472	0.271	-1.004	0.059	1.60
Park 2014	0.361	0.230	-0.089	0.811	2.22
Pérez-Jiménez 2008	-0.384	0.377	-1.123	0.355	0.82
Preuss 2000	-0.118	0.460	-1.019	0.784	0.55
Preuss 2000	-0.855	0.480	-1.796	0.086	0.51
Qin 2009	-0.034	0.183	-0.392	0.324	3.52
Queipo-Ortuno 2012	-0.084	0.633	-1.324	1.156	0.29
Queipo-Ortuno 2012	-0.051	0.633	-1.291	1.189	0.29
Rahbar 2015	-0.120	0.298	-0.705	0.465	1.32
Rahbar 2015	-0.036	0.292	-0.608	0.536	1.38
Razavi 2013	0.639	0.337	-0.022	1.300	1.03
Riso 2013	-0.181	0.472	-1.107	0.745	0.53
Santhakumar 2015	0.361	0.561	-0.738	1.460	0.37
Shema-Didi 2014	-0.342	0.253	-0.837	0.153	1.84
Sivaprakasapillai 2010	0.117	0.472	-0.808	1.042	0.53
Sivaprakasapillai 2010	-0.351	0.475	-1.282	0.580	0.52
Spaccarotella 2008	0.000	0.437	-0.856	0.856	0.61
Stull 2010	0.117	0.355	-0.578	0.812	0.93
Sumner 2005	0.200	0.318	-0.423	0.823	1.16
Tey 2013	-0.113	0.349	-0.796	0.571	0.97
Tey 2013	-0.137	0.329	-0.782	0.509	1.08
Tomé-Carneiro 2012	0.117	0.283	-0.438	0.672	1.46
Tome-Carneiro 2013	0.057	0.434	-0.793	0.907	0.62
Tsang 2005	0.619	0.467	-0.296	1.534	0.54
Tsang 2012	-0.093	0.378	-0.834	0.649	0.82
Umar 2005	-0.291	0.450	-1.172	0.590	0.58
Urquiaga 2015	0.299	0.344	-0.374	0.973	0.99
Vaisman 2015	0.089	0.354	-0.605	0.784	0.93
Vaisman 2015	0.119	0.422	-0.708	0.946	0.66
Velliquette 2015	-0.031	0.343	-0.704	0.641	1.00
Velliquette 2015	-0.313	0.356	-1.011	0.386	0.92
Vidlar 2010	0.386	0.311	-0.225	0.996	1.21
Wang 2012	0.346	0.262	-0.169	0.860	1.70
Wang 2012	0.012	0.265	-0.508	0.532	1.67
Whelan 2004	0.143	0.535	-0.906	1.192	0.41
Wien 2010	-0.428	0.276	-0.969	0.113	1.54
Wu 2014	-0.337	0.318	-0.961	0.287	1.16
Wu 2015	-0.425	0.389	-1.189	0.338	0.77
Zern 2005	0.095	0.447	-0.782	0.972	0.59
Zern 2005	0.449	0.413	-0.361	1.260	0.69
Zhang 2015	-0.276	0.254	-0.774	0.222	1.82
Zhu 2011	-0.120	0.166	-0.445	0.205	4.27
Zhu 2013	-0.122	0.166	-0.447	0.203	4.27
**Intervention effect**	**-0.065**	**0.055**	**-0.174**	**0.043**	

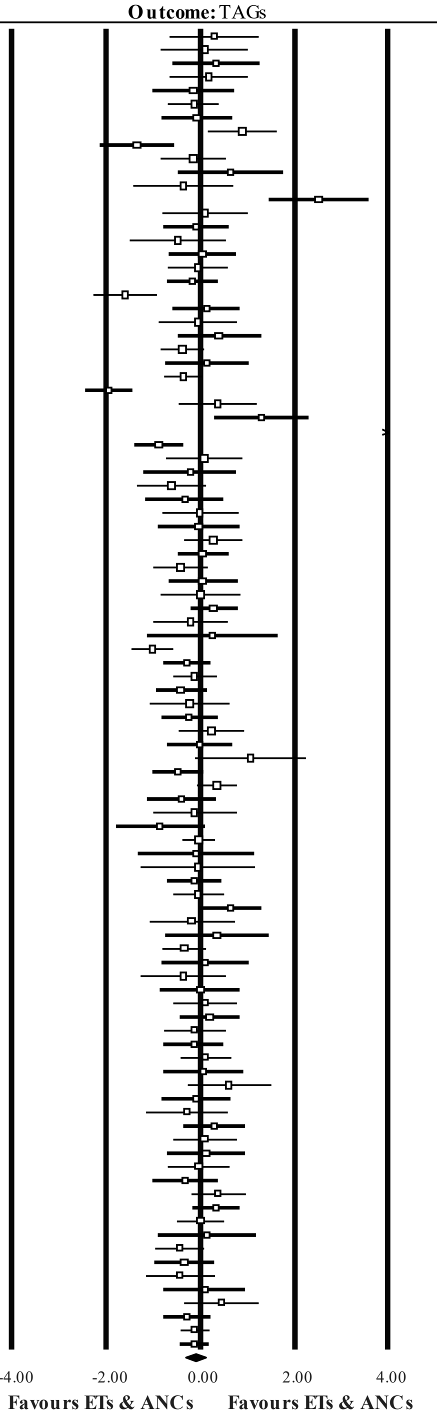

Test for overall effect Z=-1.182; *P*=0.237; Ns=1859; Nc=1743

Heterogeneity Tau^2=0.157; Q=227.7; df=96 (*P*=0.000); I^2=57.8

Supplementary Figure S17.- Forest plot of the meta-analysis evaluating the effects of a prolonged supplementation with ellagitannins and anthocyanins-containing products on TAGs. A total of 88 studies (displayed in alphabetical order) were analysed. Pooled results are shown at the bottom using a random-effects model. DM: Difference in means, SE: standard error, 95% CI: lower and upper confidence limits for the average DM, RW: relative weight.

First autor & year	DM	SE	95%CI		RW
Abu-Amsha Caccetta 2001	0.270	0.392	-0.498	1.038	0.06
Abu-Amsha Caccetta 2001	0.120	0.521	-0.900	1.140	0.04
Asgary 2013	0.139	0.243	-0.336	0.615	0.17
Asgary 2014	0.202	0.491	-0.761	1.165	0.04
Banini 2006	-0.054	0.172	-0.391	0.283	0.33
Basu 2010a	-0.100	0.218	-0.528	0.328	0.21
Basu 2010b	-0.100	0.600	-1.275	1.075	0.03
Basu 2011	0.112	0.050	0.015	0.209	4.04
Basu 2014	-0.271	0.075	-0.419	-0.123	1.75
Basu 2014	-0.023	0.069	-0.158	0.113	2.08
Bommannan Santhakumar 2015	0.220	0.187	-0.146	0.586	0.28
Bommannan Santhakumar 2015	-0.130	0.186	-0.494	0.234	0.29
Burton-Freeman 2010	0.070	0.171	-0.264	0.404	0.34
Caldu 1998	0.115	0.454	-0.775	1.006	0.05
Castilla 2006	-0.030	0.252	-0.525	0.465	0.16
Castilla 2007	-0.155	0.171	-0.489	0.179	0.34
Cerdá 2006	0.028	0.209	-0.381	0.437	0.23
Clifton 2004	-0.040	0.301	-0.629	0.549	0.11
Curtis 2007	-0.100	0.147	-0.388	0.188	0.46
Davinelli 2015	-1.215	0.257	-1.718	-0.713	0.15
Din 2011	0.100	0.269	-0.428	0.628	0.14
Dohadwala 2011	-0.034	0.278	-0.579	0.511	0.13
Duthie 2010	0.160	0.169	-0.172	0.492	0.35
Estruch 2011	0.102	0.288	-0.462	0.666	0.12
Foster 2012	-0.109	0.013	-0.134	-0.084	62.25
Goldberg 1996	0.350	0.372	-0.379	1.079	0.07
Gorinstein 2006	0.130	0.046	0.040	0.220	4.70
Gorinstein 2006	0.630	0.046	0.540	0.720	4.70
Gulati 2014	-0.360	0.207	-0.766	0.046	0.23
Guo 2014	0.056	0.270	-0.472	0.585	0.14
Haddad 2014	-0.240	0.590	-1.397	0.917	0.03
Heber 2007	-0.229	0.338	-0.892	0.433	0.09
Heber 2007	-0.005	0.632	-1.243	1.234	0.02
Hijmering 2007	-0.015	0.340	-0.681	0.651	0.09
Hokayem 2013	0.130	0.151	-0.166	0.426	0.43
Hollis 2013	0.040	0.185	-0.322	0.402	0.29
Hosseini 2016	-0.434	0.335	-1.091	0.223	0.09
Jenkins 2008	0.040	0.268	-0.485	0.565	0.14
Jin 2011	0.010	0.297	-0.572	0.592	0.11
Karlsen 2010	0.130	0.118	-0.101	0.361	0.71
Katz 2012	-0.099	0.200	-0.492	0.293	0.25
Kay 2002	0.300	0.733	-1.136	1.736	0.02
Kianbakht 2014	-0.320	0.067	-0.452	-0.188	2.19
Lee 2014	-0.418	0.400	-1.202	0.366	0.06
Lekakis 2005	-0.410	0.784	-1.946	1.126	0.02
Li 2010	-0.329	0.227	-0.774	0.117	0.19
Ma 2010	-0.114	0.226	-0.557	0.329	0.19
Naissides 2006	0.130	0.196	-0.255	0.515	0.26
Naissides 2006	-0.010	0.191	-0.384	0.364	0.27
Natella 2011	0.350	0.168	0.021	0.679	0.35
Novotny 2015	-0.100	0.130	-0.354	0.154	0.59
Park 2014	0.293	0.185	-0.070	0.655	0.29
Pérez-Jímenez 2008	-0.225	0.264	-0.742	0.292	0.14
Preuss 2000	-0.056	0.220	-0.488	0.375	0.20
Preuss 2000	-0.745	0.400	-1.530	0.040	0.06
Qin 2009	-0.034	0.179	-0.385	0.317	0.31
Queipo-Ortuno 2012	-0.203	1.598	-3.335	2.929	0.00
Queipo-Ortuno 2012	-0.116	1.562	-3.177	2.944	0.00
Rahbar 2015	-0.109	0.275	-0.647	0.430	0.13
Rahbar 2015	-0.034	0.299	-0.619	0.551	0.11
Riso 2013	-0.100	0.260	-0.610	0.410	0.15
Santhakumar 2015	0.130	0.186	-0.234	0.494	0.29
Shema-Didi 2014	-0.362	0.270	-0.892	0.167	0.14
Sivaprakasapillai 2010	0.023	0.094	-0.162	0.207	1.11
Sivaprakasapillai 2010	-0.068	0.098	-0.260	0.125	1.03
Spaccarotella 2008	0.000	2.027	-3.973	3.973	0.00
Stull 2010	0.100	0.292	-0.472	0.672	0.12
Sumner 2005	0.237	0.357	-0.463	0.937	0.08
Tey 2013	-0.040	0.142	-0.318	0.238	0.49
Tey 2013	-0.060	0.175	-0.404	0.284	0.32
Tomé-Carneiro 2012	0.086	0.189	-0.284	0.456	0.28
Tome-Carneiro 2013	0.056	0.415	-0.758	0.871	0.06
Tsang 2005	0.200	0.186	-0.165	0.565	0.29
Tsang 2012	-0.042	0.209	-0.451	0.367	0.23
Umar 2005	-0.170	0.280	-0.718	0.378	0.13
Urquiaga 2015	0.315	0.357	-0.385	1.015	0.08
Vaisman 2015	0.070	0.269	-0.458	0.598	0.14
Vaisman 2015	0.080	0.278	-0.465	0.625	0.13
Velliquette 2015	-0.017	0.172	-0.354	0.320	0.34
Velliquette 2015	-0.110	0.145	-0.393	0.174	0.47
Vidlar 2010	0.225	0.178	-0.123	0.573	0.31
Wang 2012	0.270	0.207	-0.136	0.676	0.23
Wang 2012	0.010	0.217	-0.416	0.436	0.21
Whelan 2004	0.100	0.334	-0.554	0.754	0.09
Wu 2014	-0.090	0.085	-0.257	0.076	1.38
Wu 2015	-0.341	0.442	-1.207	0.525	0.05
Zern 2005	0.100	0.507	-0.894	1.094	0.04
Zern 2005	0.200	0.206	-0.205	0.605	0.23
**Intervention effect**	**-0.071**	**0.033**	**-0.071**	**0.059**	

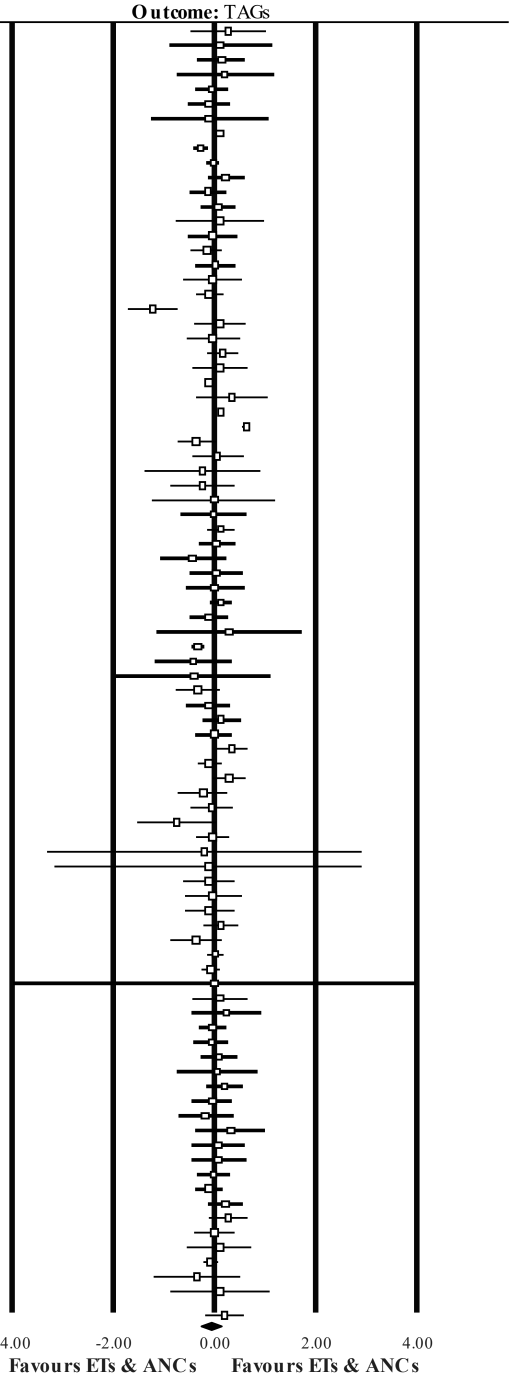

Test for overall effect Z=-0.177; *P*=0.086; Ns=1525; Nc=1404

Heterogeneity Tau^2=0.045; Q=360.4; df=87 (*P*=0.000); I^2=75.9

Supplementary Figure S18. Funnel plots and Egger statistics (intercept and 2-tailed *P*-value) of the meta-analysis evaluating the effects of a prolonged supplementation with ellagitannins and anthocyanins-containing products on triacylglycerols (TAGs).

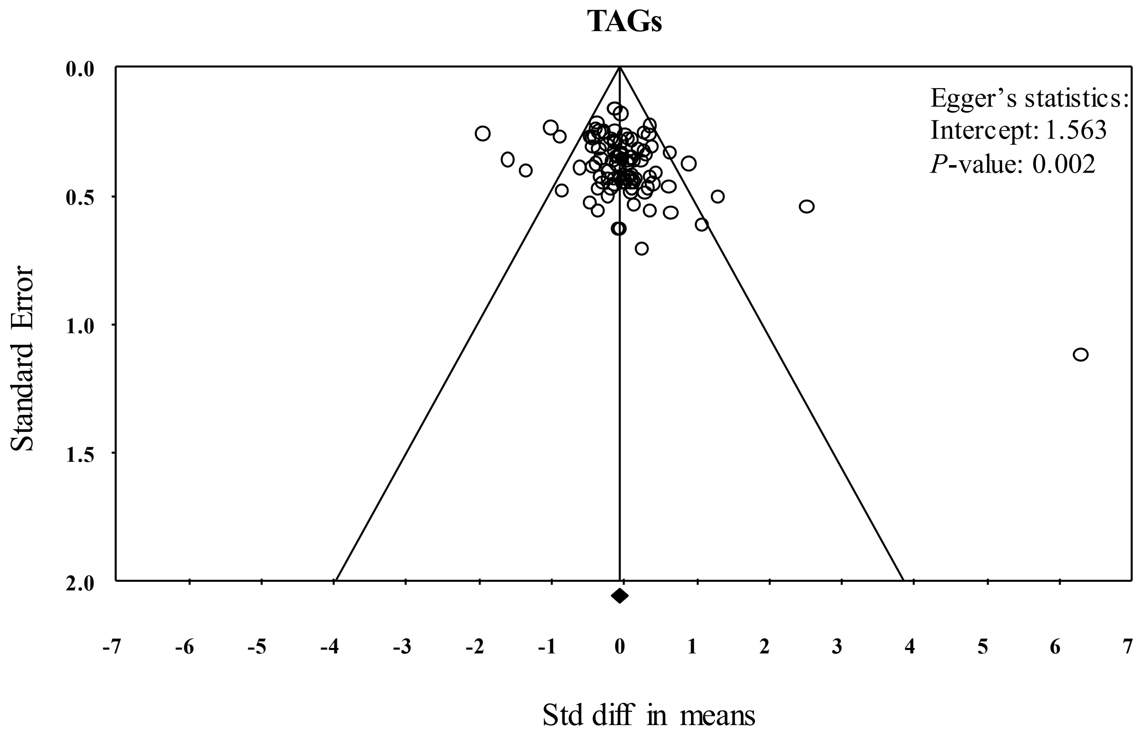

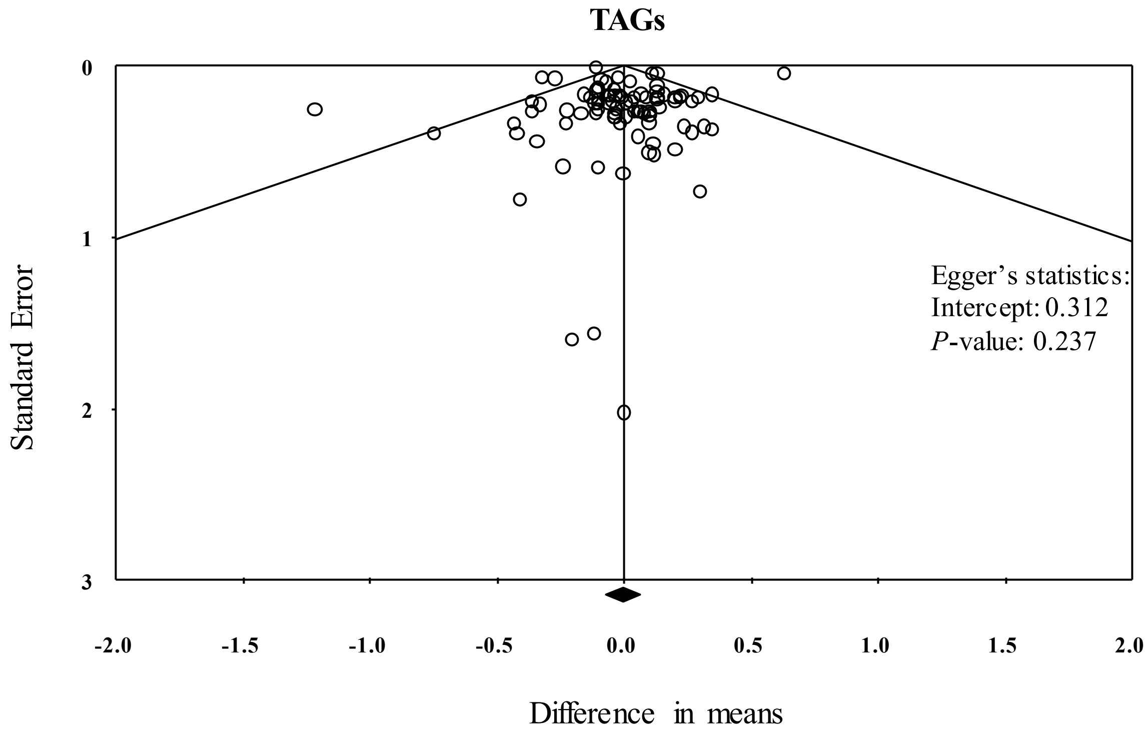

Supplementary Figure S19. Forest plot of the meta-analysis evaluating the effects of a prolonged supplementation with ellagitannins and anthocyanins-containing products on SBP. A total of 95 studies (displayed in alphabetical order) were analysed. Pooled results are shown at the bottom using a random-effects model. SDM: standardized difference in means, SE: standard error, 95% CI: lower and upper confidence limits for the average SDM, RW: relative weight.

First autor & year	SDM	SE	95%CI		RW
Asgary 2014	-0.437	0.442	-1.303	0.430	-0.48
Banini 2006	0.140	0.438	-0.719	0.999	0.72
Basu 2010a	-0.448	0.293	-1.021	0.126	-0.70
Basu 2010b	-0.191	0.388	-0.952	0.570	0.03
Basu 2011	-0.420	0.363	-1.132	0.292	-0.53
Basu 2014	-0.258	0.367	-0.977	0.460	-0.13
Basu 2014	-0.203	0.366	-0.920	0.515	0.00
Botden 2012	0.000	0.378	-0.741	0.741	0.48
Botden 2012	0.000	0.365	-0.716	0.716	0.50
Chiva-Blanch 2012	-0.444	0.376	-1.182	0.293	-0.57
Chiva-Blanch 2012	-0.175	0.325	-0.812	0.462	0.08
Chiva-Blanch 2012	-0.120	0.372	-0.849	0.609	0.20
Chiva-Blanch 2012	-0.120	0.325	-0.756	0.517	0.22
Clifton 2004	0.000	0.333	-0.653	0.653	0.53
Cook 2015	-0.324	0.538	-1.378	0.731	-0.21
Curtis 2009	0.499	0.282	-0.053	1.051	2.07
Davinelli 2015	0.080	0.318	-0.543	0.703	0.77
Del Bo 2013	-0.035	0.633	-1.275	1.205	0.26
Del Bo 2014	-1.488	0.565	-2.596	-0.381	-2.16
Din 2011	0.688	0.376	-0.049	1.424	2.12
Dohadwala 2011	0.000	0.426	-0.836	0.836	0.44
Draijer 2015	-1.076	0.391	-1.842	-0.310	-2.01
Draijer 2015	-0.260	0.367	-0.979	0.458	-0.14
Estruch 2011	-0.258	0.449	-1.138	0.622	-0.11
Flammer 2013	-0.198	0.216	-0.622	0.226	0.02
Foster 2012	-0.643	0.216	-1.067	-0.219	-1.53
Guo 2014	-0.256	0.428	-1.095	0.583	-0.11
Hansen 2005	-0.239	0.339	-0.904	0.426	-0.09
Hansen 2005	-0.245	0.351	-0.933	0.443	-0.10
Hansen 2005	-0.232	0.330	-0.879	0.414	-0.07
Hassellund 2012	-0.139	0.557	-1.230	0.953	0.11
Hokayem 2013	-0.220	0.326	-0.858	0.419	-0.04
Johnson 2015	-0.250	0.311	-0.860	0.361	-0.12
Katz 2012	-0.350	0.411	-1.156	0.456	-0.32
Khan 2014	-0.521	0.314	-1.136	0.094	-0.87
Khan 2014	-0.227	0.306	-0.827	0.373	-0.06
Lamport 2016	0.191	0.305	-0.406	0.787	1.10
Lee 2014	-0.044	0.258	-0.550	0.462	0.50
Lekakis 2005	-0.083	0.251	-0.576	0.409	0.39
López-Uriarte 2010	-0.338	0.285	-0.896	0.220	-0.39
Lynn 2012	0.333	0.291	-0.237	0.902	1.55
Lynn 2014	-0.263	0.297	-0.846	0.319	-0.17
Ma 2010	0.840	0.456	-0.053	1.734	2.12
Mathew 2012	-0.855	0.480	-1.796	0.085	-1.26
Mathew 2012	0.627	0.471	-0.295	1.550	1.64
McAnulty 2005	-0.151	0.448	-1.028	0.727	0.11
McAnulty 2014	-0.247	0.402	-1.035	0.540	-0.10
McKay 2010	-0.565	0.254	-1.062	-0.068	-1.14
Mukuddem-Petersen 2007	0.380	0.308	-0.223	0.983	1.62
Naissides 2006	-0.389	0.369	-1.113	0.335	-0.45
Naissides 2006	0.007	0.373	-0.725	0.739	0.50
Novotny 2015	-1.940	0.324	-2.576	-1.305	-4.63
Ohguro 2012	-0.232	0.326	-0.870	0.407	-0.07
Olmedilla-Alonso 2008	-0.266	0.402	-1.054	0.522	-0.14
Parham 2015	-1.140	0.471	-2.063	-0.217	-1.84
Pérez-Jímenez 2008	-0.039	0.375	-0.774	0.696	0.39
Puupponen-Pimiä 2013	-0.303	0.367	-1.023	0.417	-0.24
Qin 2009	-0.167	0.183	-0.525	0.191	0.14
Queipo-Ortuno 2012	-0.324	0.637	-1.571	0.924	-0.18
Queipo-Ortuno 2012	-0.657	0.649	-1.930	0.615	-0.67
Ras 2013	-0.230	0.242	-0.704	0.243	-0.09
Razavi 2013	-0.117	0.329	-0.762	0.529	0.23
Riso 2013	-0.063	0.472	-0.987	0.862	0.28
Rodriguez-Mateos 2014	-0.230	0.637	-1.480	1.019	-0.04
Rodriguez-Mateos 2014	0.486	0.646	-0.780	1.752	1.03
Rodriguez-Mateos 2014	-0.831	0.674	-2.153	0.491	-0.89
Ruel 2013	-0.441	0.342	-1.112	0.230	-0.61
Ruel 2013	0.631	0.346	-0.048	1.310	2.12
Shema-Didi 2014	-0.467	0.255	-0.966	0.032	-0.83
Sivaprakasapillai 2010	-0.750	0.488	-1.706	0.206	-1.04
Sivaprakasapillai 2010	-0.849	0.492	-1.813	0.116	-1.22
Spaak 2008	0.420	0.397	-0.358	1.197	1.42
Spaccarotella 2008	-0.109	0.437	-0.966	0.748	0.20
Stull 2010	-0.750	0.366	-1.468	-0.032	-1.32
Sumner 2005	-0.047	0.317	-0.668	0.575	0.43
Terauchi 2014a	-0.306	0.262	-0.819	0.208	-0.31
Terauchi 2014a	-0.464	0.260	-0.973	0.046	-0.81
Terauchi 2014b	-0.247	0.257	-0.752	0.257	-0.14
Terauchi 2014b	-0.495	0.260	-1.005	0.015	-0.90
Tey 2013	-0.200	0.240	-0.670	0.271	0.01
Tey 2013	-0.179	0.233	-0.635	0.278	0.09
Tome-Carneiro 2013	0.085	0.434	-0.766	0.935	0.61
Tsang 2012	-0.703	0.389	-1.467	0.060	-1.15
Urquiaga 2010	-0.184	0.343	-0.855	0.488	0.05
Vaisman 2015	0.393	0.358	-0.308	1.094	1.48
Vaisman 2015	-0.231	0.423	-1.060	0.598	-0.06
Watson 2015	-0.185	0.334	-0.840	0.470	0.05
Watson 2015	-0.061	0.333	-0.714	0.593	0.37
Whelan 2004	0.115	0.535	-0.934	1.164	0.56
Wien 2010	-0.079	0.273	-0.614	0.456	0.38
Williams 2004	0.142	0.558	-0.952	1.235	0.59
Wu 2015	0.818	0.401	0.032	1.604	2.31
Zhang 2015	0.208	0.253	-0.288	0.705	1.31
Zhu 2013	-0.230	0.166	-0.556	0.095	-0.10
Zunino 2014	-0.851	0.426	-1.686	-0.015	-1.39
**Intervention effect**	**-0.204**	**0.041**	**-0.284**	**-0.124**	

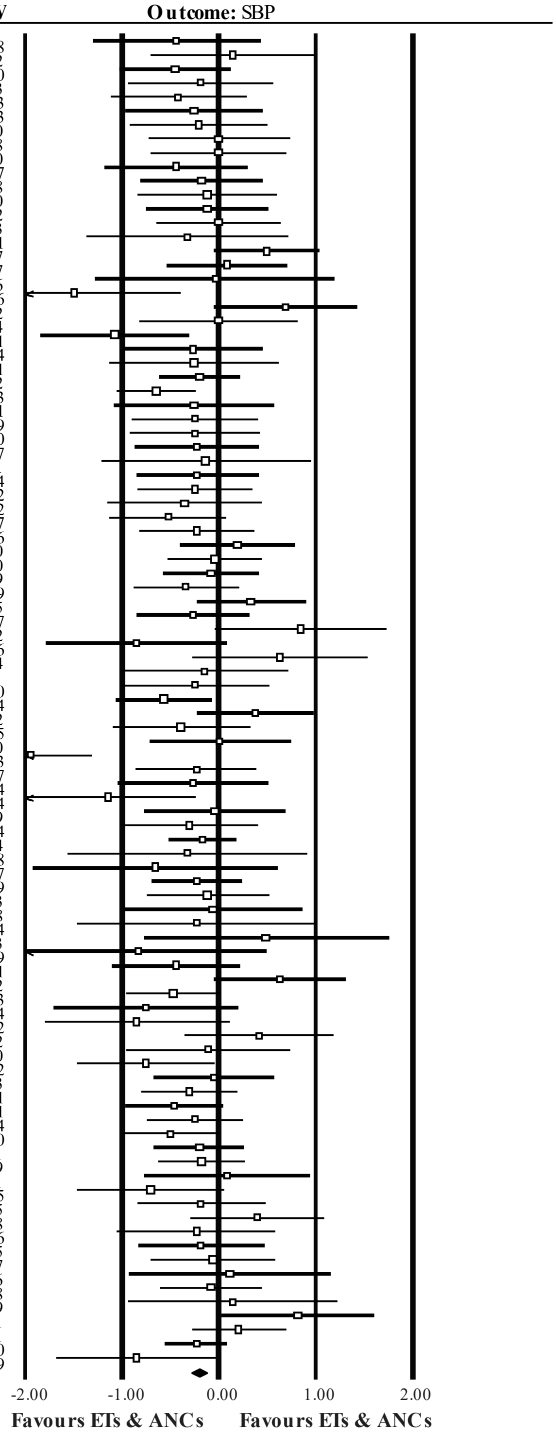

Test for overall effect Z=-5.013; *P*=0.000; Ns=1812; Nc=1717

Heterogeneity Tau^2=0.037; Q=125.3; df=94 (*P*=0.017); I^2=25.0

Supplementary Figure S20.- Forest plot of the meta-analysis evaluating the effects of a prolonged supplementation with ellagitannins and anthocyanins-containing products on SBP. A total of 83 studies (displayed in alphabetical order) were analysed. Pooled results are shown at the bottom using a random-effects model. DM: Difference in means, SE: standard error, 95% CI: lower and upper confidence limits for the average DM, RW: relative weight.

First autor & year	DM	SE	95%CI		RW
Asgary 2014	-0.437	0.442	-1.303	0.430	0.71
Banini 2006	0.140	0.438	-0.719	0.999	0.72
Basu 2010a	-0.448	0.293	-1.021	0.126	1.35
Basu 2010b	-0.191	0.388	-0.952	0.570	0.88
Basu 2011	-0.420	0.363	-1.132	0.292	0.98
Basu 2014	-0.258	0.367	-0.977	0.460	0.97
Basu 2014	-0.203	0.366	-0.920	0.515	0.97
Botden 2012	0.000	0.378	-0.741	0.741	0.92
Botden 2012	0.000	0.365	-0.716	0.716	0.97
Chiva-Blanch 2012	-0.444	0.376	-1.182	0.293	0.93
Chiva-Blanch 2012	-0.175	0.325	-0.812	0.462	1.16
Chiva-Blanch 2012	-0.120	0.372	-0.849	0.609	0.95
Chiva-Blanch 2012	-0.120	0.325	-0.756	0.517	1.16
Clifton 2004	0.000	0.333	-0.653	0.653	1.12
Cook 2015	-0.324	0.538	-1.378	0.731	0.51
Curtis 2009	0.499	0.282	-0.053	1.051	1.42
Davinelli 2015	0.080	0.318	-0.543	0.703	1.20
Del Bo 2013	-0.035	0.633	-1.275	1.205	0.38
Del Bo 2014	-1.488	0.565	-2.596	-0.381	0.47
Din 2011	0.688	0.376	-0.049	1.424	0.93
Dohadwala 2011	0.000	0.426	-0.836	0.836	0.76
Draijer 2015	-1.076	0.391	-1.842	-0.310	0.87
Draijer 2015	-0.260	0.367	-0.979	0.458	0.97
Estruch 2011	-0.258	0.449	-1.138	0.622	0.70
Flammer 2013	-0.198	0.216	-0.622	0.226	1.98
Foster 2012	-0.643	0.216	-1.067	-0.219	1.98
Guo 2014	-0.256	0.428	-1.095	0.583	0.75
Hansen 2005	-0.239	0.339	-0.904	0.426	1.09
Hansen 2005	-0.245	0.351	-0.933	0.443	1.04
Hansen 2005	-0.232	0.330	-0.879	0.414	1.14
Hassellund 2012	-0.139	0.557	-1.230	0.953	0.48
Hokayem 2013	-0.220	0.326	-0.858	0.419	1.16
Johnson 2015	-0.250	0.311	-0.860	0.361	1.24
Katz 2012	-0.350	0.411	-1.156	0.456	0.80
Khan 2014	-0.521	0.314	-1.136	0.094	1.22
Khan 2014	-0.227	0.306	-0.827	0.373	1.27
Lamport 2016	0.191	0.305	-0.406	0.787	1.28
Lee 2014	-0.044	0.258	-0.550	0.462	1.60
Lekakis 2005	-0.083	0.251	-0.576	0.409	1.66
López-Uriarte 2010	-0.338	0.285	-0.896	0.220	1.40
Lynn 2012	0.333	0.291	-0.237	0.902	1.36
Lynn 2014	-0.263	0.297	-0.846	0.319	1.32
Ma 2010	0.840	0.456	-0.053	1.734	0.68
Mathew 2012	-0.855	0.480	-1.796	0.085	0.62
Mathew 2012	0.627	0.471	-0.295	1.550	0.64
McAnulty 2005	-0.151	0.448	-1.028	0.727	0.70
McAnulty 2014	-0.247	0.402	-1.035	0.540	0.84
McKay 2010	-0.565	0.254	-1.062	-0.068	1.63
Mukuddem-Petersen 2007	0.380	0.308	-0.223	0.983	1.26
Naissides 2006	-0.389	0.369	-1.113	0.335	0.96
Naissides 2006	0.007	0.373	-0.725	0.739	0.94
Novotny 2015	-1.940	0.324	-2.576	-1.305	1.17
Ohguro 2012	-0.232	0.326	-0.870	0.407	1.16
Olmedilla-Alonso 2008	-0.266	0.402	-1.054	0.522	0.83
Parham 2015	-1.140	0.471	-2.063	-0.217	0.64
Pérez-Jímenez 2008	-0.039	0.375	-0.774	0.696	0.93
Puupponen-Pimiä 2013	-0.303	0.367	-1.023	0.417	0.96
Qin 2009	-0.167	0.183	-0.525	0.191	2.35
Queipo-Ortuno 2012	-0.324	0.637	-1.571	0.924	0.38
Queipo-Ortuno 2012	-0.657	0.649	-1.930	0.615	0.36
Ras 2013	-0.230	0.242	-0.704	0.243	1.74
Razavi 2013	-0.117	0.329	-0.762	0.529	1.14
Riso 2013	-0.063	0.472	-0.987	0.862	0.64
Rodriguez-Mateos 2014	-0.230	0.637	-1.480	1.019	0.37
Rodriguez-Mateos 2014	0.486	0.646	-0.780	1.752	0.37
Rodriguez-Mateos 2014	-0.831	0.674	-2.153	0.491	0.34
Ruel 2013	-0.441	0.342	-1.112	0.230	1.08
Ruel 2013	0.631	0.346	-0.048	1.310	1.06
Shema-Didi 2014	-0.467	0.255	-0.966	0.032	1.63
Sivaprakasapillai 2010	-0.750	0.488	-1.706	0.206	0.60
Sivaprakasapillai 2010	-0.849	0.492	-1.813	0.116	0.59
Spaak 2008	0.420	0.397	-0.358	1.197	0.85
Spaccarotella 2008	-0.109	0.437	-0.966	0.748	0.73
Stull 2010	-0.750	0.366	-1.468	-0.032	0.97
Sumner 2005	-0.047	0.317	-0.668	0.575	1.21
Terauchi 2014a	-0.306	0.262	-0.819	0.208	1.57
Terauchi 2014a	-0.464	0.260	-0.973	0.046	1.58
Terauchi 2014b	-0.247	0.257	-0.752	0.257	1.61
Terauchi 2014b	-0.495	0.260	-1.005	0.015	1.58
Tey 2013	-0.200	0.240	-0.670	0.271	1.75
Tey 2013	-0.179	0.233	-0.635	0.278	1.82
Tome-Carneiro 2013	0.085	0.434	-0.766	0.935	0.74
Tsang 2012	-0.703	0.389	-1.467	0.060	0.88
Urquiaga 2010	-0.184	0.343	-0.855	0.488	1.07
Vaisman 2015	0.393	0.358	-0.308	1.094	1.01
Vaisman 2015	-0.231	0.423	-1.060	0.598	0.77
Watson 2015	-0.185	0.334	-0.840	0.470	1.12
Watson 2015	-0.061	0.333	-0.714	0.593	1.12
Whelan 2004	0.115	0.535	-0.934	1.164	0.51
Wien 2010	-0.079	0.273	-0.614	0.456	1.49
Williams 2004	0.142	0.558	-0.952	1.235	0.48
Wu 2015	0.818	0.401	0.032	1.604	0.84
Zhang 2015	0.208	0.253	-0.288	0.705	1.64
Zhu 2013	-0.230	0.166	-0.556	0.095	2.56
Zunino 2014	-0.851	0.426	-1.686	-0.015	0.76
**Intervention effect**	**-1.561**	**0.289**	**-2.129**	**-0.994**	

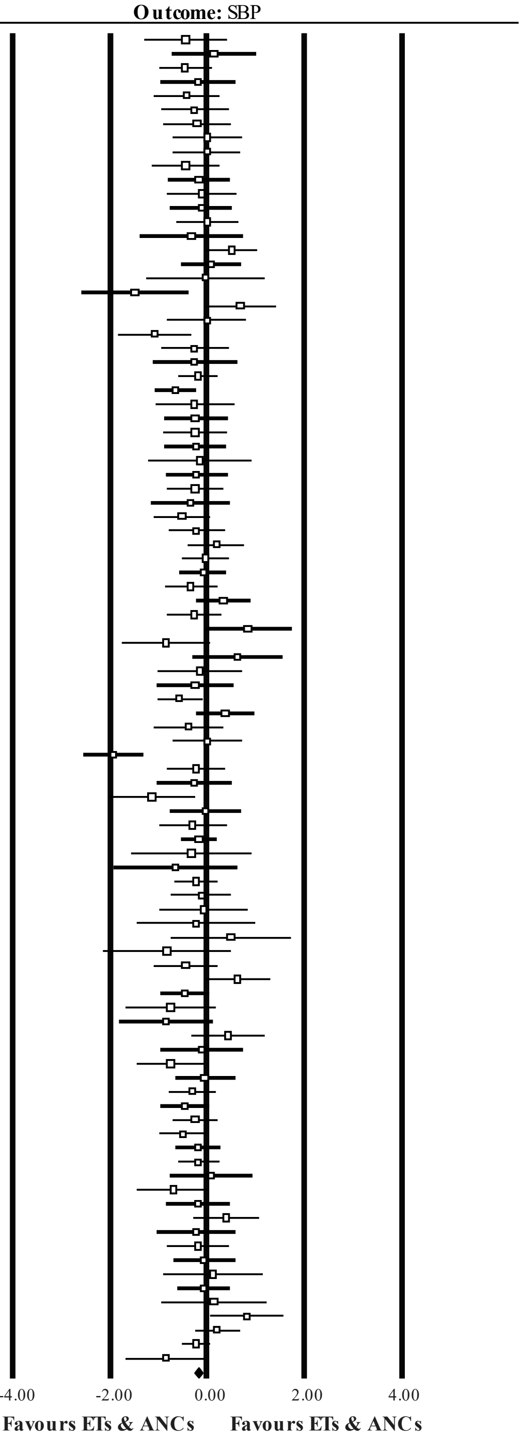

Test for overall effect Z=-5.394; P=0.000; Ns=1630; Nc=1545

Heterogeneity Tau^2=0.000; Q=69.0; df=82 (P=0.846); I^2=0.00

Supplementary Figure S21. Funnel plots and Egger statistics (intercept and 2-tailed *P*-value) of the meta-analysis evaluating the effects of a prolonged supplementation with ellagitannins and anthocyanins-containing products on systolic blood pressure (SBP).

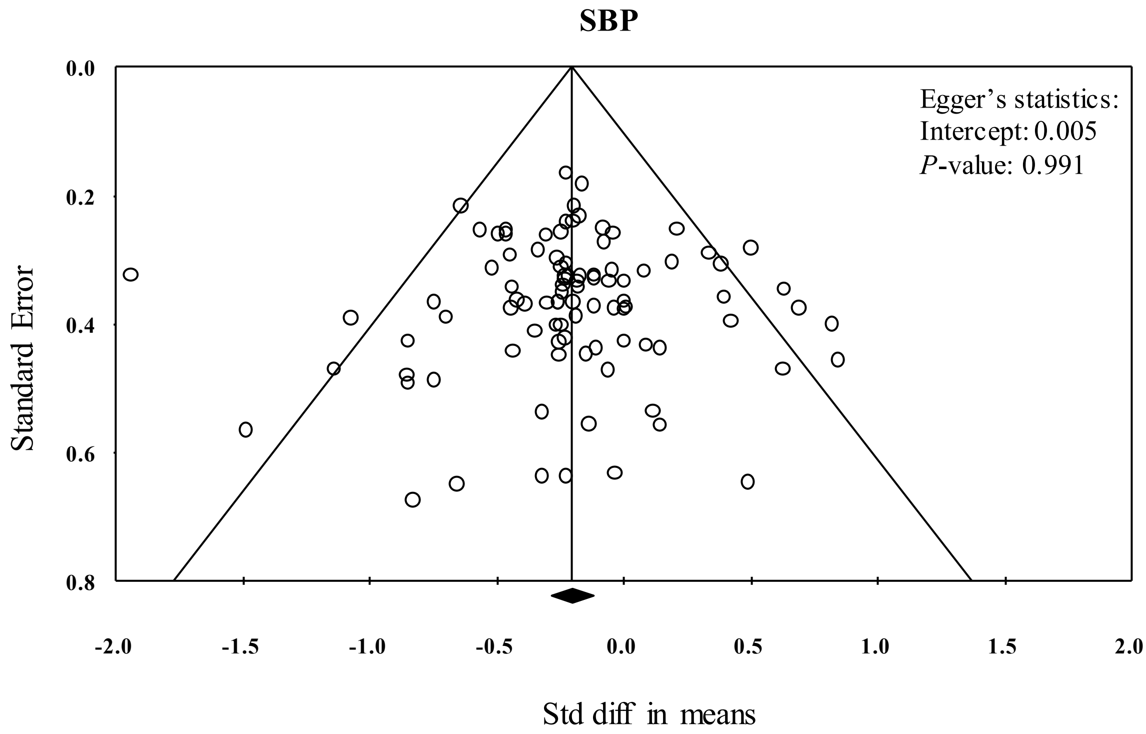

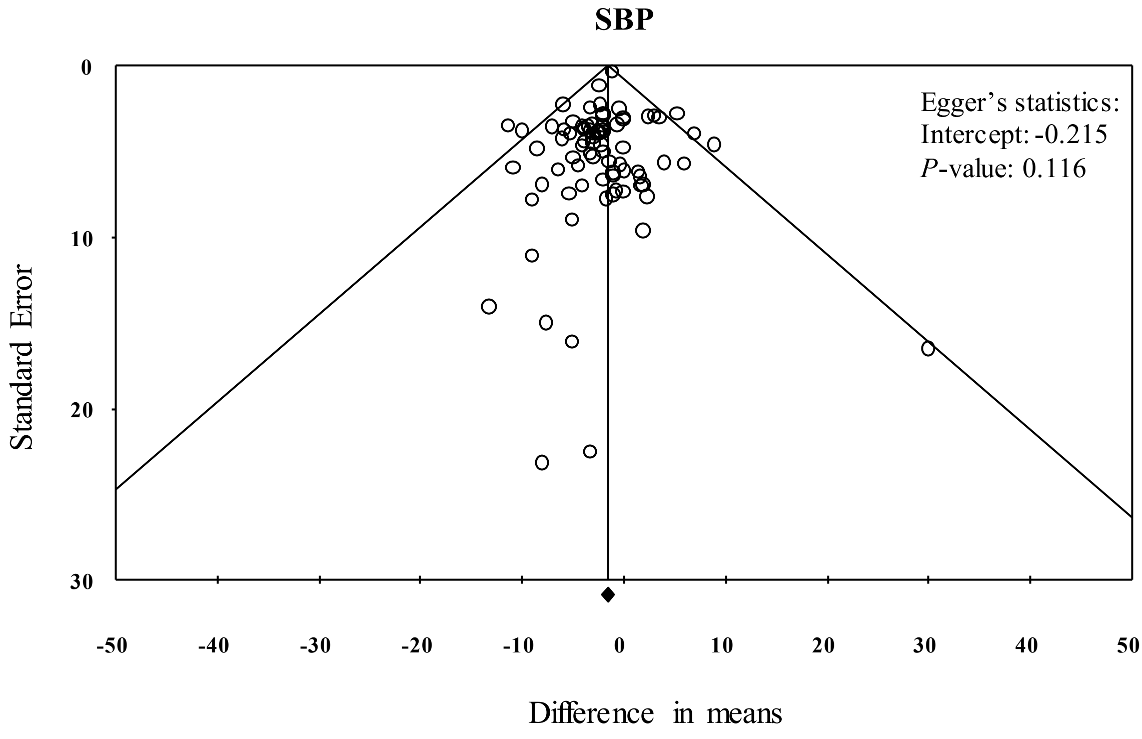

Supplementary Figure S22. Forest plot of the meta-analysis evaluating the effects of a prolonged supplementation with ellagitannins and anthocyanins-containing products on DBP. A total of 99 studies (displayed in alphabetical order) were analysed. Pooled results are shown at the bottom using a random-effects model. SDM: standardized difference in means, SE: standard error, 95% CI: lower and upper confidence limits for the average SDM, RW: relative weight.

First autor & year	SDM	SE	95% CI		RW
Asgary 2014	-0.493	0.443	-1.362	0.377	0.69
Banini 2006	0.000	0.438	-0.858	0.858	0.70
Basu 2010a	-0.479	0.293	-1.053	0.095	1.29
Basu 2010b	-0.117	0.388	-0.877	0.643	0.86
Basu 2011	0.073	0.633	-1.167	1.313	0.37
Basu 2014	0.000	0.365	-0.716	0.716	0.94
Basu 2014	-2.530	0.490	-3.490	-1.570	0.58
Botden 2012	0.000	0.378	-0.741	0.741	0.89
Botden 2012	0.000	0.365	-0.716	0.716	0.94
Chiva-Blanch 2012	-0.333	0.374	-1.067	0.400	0.91
Chiva-Blanch 2012	-0.208	0.325	-0.846	0.430	1.11
Chiva-Blanch 2012	-0.211	0.373	-0.941	0.520	0.91
Chiva-Blanch 2012	-0.100	0.325	-0.736	0.537	1.12
Clifton 2004	-0.089	0.472	-1.013	0.836	0.62
Cook 2015	-0.743	0.553	-1.826	0.341	0.47
Curtis 2009	0.116	0.278	-0.428	0.660	1.38
Davinelli 2015	0.054	0.318	-0.569	0.677	1.15
Del Bo 2013	-0.307	0.636	-1.554	0.940	0.37
Del Bo 2014	-0.190	0.501	-1.172	0.792	0.56
Din 2011	0.162	0.366	-0.554	0.879	0.94
Dohadwala 2011	0.094	0.305	-0.504	0.692	1.22
Draijer 2015	-0.235	0.366	-0.953	0.483	0.93
Draijer 2015	0.415	0.369	-0.308	1.138	0.92
Erlund 2008	-0.487	0.241	-0.959	-0.015	1.65
Flammer 2013	-0.219	0.251	-0.712	0.274	1.57
Foster 2012	0.529	0.235	0.069	0.990	1.69
Guo 2014	-0.059	0.251	-0.552	0.433	1.57
Hansen 2005	-0.241	0.242	-0.715	0.233	1.64
Hansen 2005	-0.208	0.284	-0.764	0.348	1.35
Hansen 2005	-0.233	0.339	-0.898	0.433	1.05
Hassellund 2012	0.111	0.427	-0.725	0.947	0.74
Hokayem 2013	0.468	0.329	-0.178	1.113	1.10
Jenkins 2008	-0.075	0.378	-0.816	0.666	0.89
Johnson 2015	-0.554	0.316	-1.174	0.066	1.16
Katz 2012	-0.350	0.298	-0.934	0.235	1.26
Khan 2014	-0.022	0.359	-0.726	0.682	0.96
Khan 2014	-0.719	0.390	-1.483	0.046	0.85
Lamport 2016	0.258	0.305	-0.340	0.856	1.22
Lekakis 2005	-0.056	0.251	-0.548	0.435	1.57
López-Uriarte 2010	-0.211	0.473	-1.137	0.716	0.62
Lynn 2012	-1.078	0.412	-1.885	-0.270	0.78
Lynn 2014	-0.172	0.318	-0.795	0.450	1.15
Ma 2010	0.730	0.451	-0.155	1.614	0.67
McAnulty 2005	-0.209	0.302	-0.801	0.384	1.23
McAnulty 2014	-0.697	0.412	-1.505	0.111	0.78
McKay 2010	-0.297	0.250	-0.787	0.193	1.58
Mukuddem-Petersen 2007	0.349	0.217	-0.077	0.775	1.85
Naissides 2006	-0.407	0.370	-1.132	0.317	0.92
Naissides 2006	0.125	0.374	-0.608	0.857	0.91
Novotny 2015	-1.936	0.324	-2.571	-1.301	1.12
Ohguro 2012	0.607	0.436	-0.248	1.461	0.71
Olmedilla-Alonso 2008	-0.154	0.401	-0.940	0.632	0.81
Parham 2015	0.000	0.437	-0.856	0.856	0.71
Park 2004	-0.223	0.318	-0.845	0.400	1.15
Pérez-Jímenez 2008	-0.330	0.377	-1.068	0.408	0.90
Puupponen-Pimiä 2013	-0.023	0.365	-0.739	0.693	0.94
Puupponen-Pimiä 2013	-0.136	0.557	-1.228	0.956	0.47
Puupponen-Pimiä 2013	-0.491	0.329	-1.136	0.154	1.10
Qin 2009	-0.074	0.183	-0.432	0.284	2.19
Queipo-Ortuno 2012	-0.451	0.354	-1.145	0.243	0.98
Queipo-Ortuno 2012	-0.697	0.339	-1.361	-0.033	1.05
Ras 2013	-0.283	0.474	-1.211	0.646	0.62
Razavi 2013	-0.419	0.333	-1.071	0.233	1.08
Riso 2013	-0.409	0.292	-0.980	0.163	1.30
Rodriguez-Mateos 2014	0.294	0.644	-0.969	1.557	0.36
Rodriguez-Mateos 2014	-0.245	0.641	-1.502	1.011	0.36
Rodriguez-Mateos 2014	-0.191	0.634	-1.433	1.052	0.37
Rodriguez-Mateos 2014	-0.914	0.665	-2.217	0.389	0.34
Rodriguez-Mateos 2014	-0.543	0.644	-1.806	0.719	0.36
Ruel 2013	0.236	0.339	-0.429	0.902	1.05
Ruel 2013	-0.267	0.340	-0.933	0.399	1.05
Shema-Didi 2014	-0.300	0.252	-0.794	0.194	1.56
Shema-Didi 2014	-0.070	0.557	-1.161	1.021	0.47
Sivaprakasapillai 2010	0.000	0.447	-0.877	0.877	0.68
Sivaprakasapillai 2010	0.000	0.333	-0.653	0.653	1.08
Spaak 2008	0.046	0.556	-1.045	1.136	0.47
Spaccarotella 2008	-0.180	0.438	-1.038	0.678	0.70
Stull 2010	-0.601	0.362	-1.311	0.109	0.95
Sumner 2005	-0.608	0.262	-1.122	-0.094	1.49
Terauchi 2014a	-0.519	0.261	-1.030	-0.009	1.50
Terauchi 2014a	-0.303	0.262	-0.817	0.211	1.49
Terauchi 2014b	-0.120	0.365	-0.836	0.597	0.94
Tey 2013	-0.170	0.240	-0.641	0.300	1.66
Tey 2013	-0.074	0.233	-0.530	0.382	1.71
Tome-Carneiro 2013	0.195	0.435	-0.657	1.047	0.71
Tsang 2012	-1.079	0.677	-2.405	0.248	0.33
Urquiaga 2015	-0.213	0.343	-0.885	0.459	1.03
Vaisman 2015	0.183	0.355	-0.513	0.879	0.98
Vaisman 2015	-0.747	0.435	-1.600	0.106	0.71
Watson 2015	0.171	0.335	-0.486	0.828	1.07
Watson 2015	0.014	0.333	-0.640	0.667	1.08
Whelan 2004	-0.053	0.535	-1.101	0.995	0.50
Wien 2010	-0.126	0.273	-0.662	0.409	1.41
Williams 2004	0.267	0.306	-0.334	0.867	1.21
Williams 2004	-0.067	0.309	-0.672	0.538	1.20
Wu 2015	-0.416	0.259	-0.924	0.092	1.51
Zhang 2015	0.199	0.253	-0.298	0.696	1.55
Zhu 2013	-0.042	0.166	-0.366	0.283	2.37
**Intervention effect**	**-0.185**	**0.040**	**-0.264**	**-0.106**	

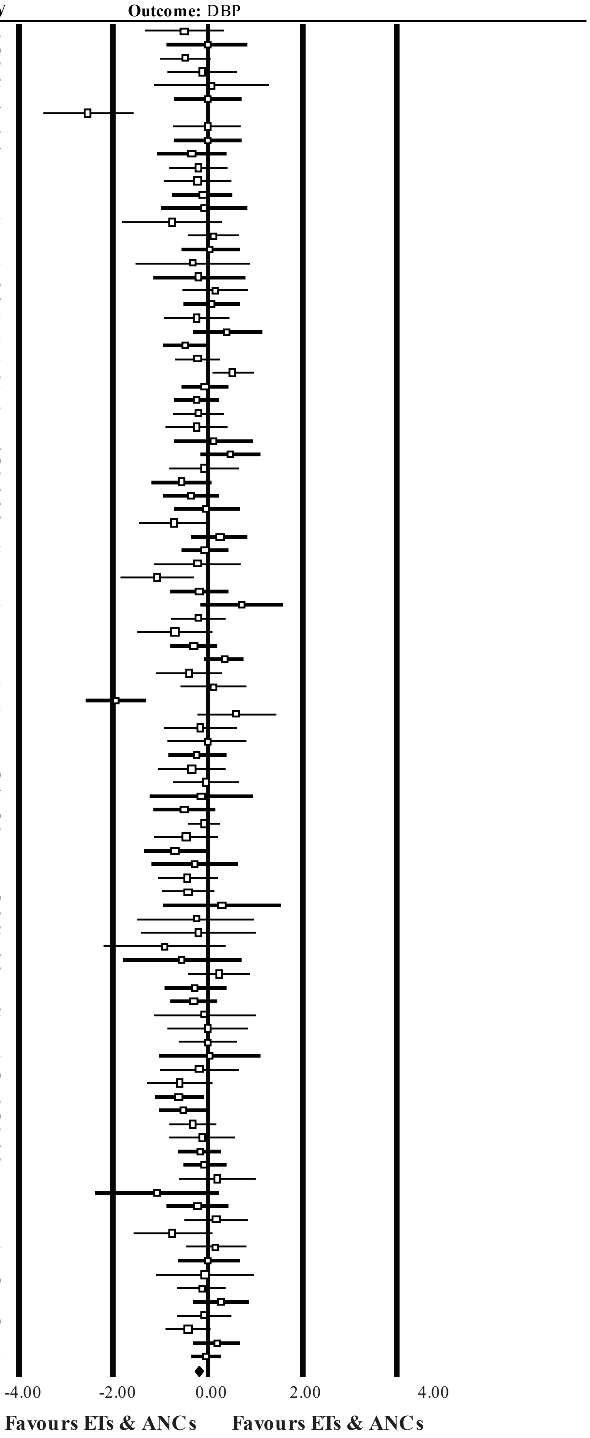

Test for overall effect Z=-4.600; P=0.000; Ns=1959; Nc=1831

Heterogeneity Tau^2=0.042; Q=135.8; df=98 (P=0.007); I^2=27.9

Supplementary Figure S23.- Forest plot of the meta-analysis evaluating the effects of a prolonged supplementation with ellagitannins and anthocyanins-containing products on DBP. A total of 90 studies (displayed in alphabetical order) were analysed. Pooled results are shown at the bottom using a random-effects model. DM: Difference in means, SE: standard error, 95% CI: lower and upper confidence limits for the average DM, RW: relative weight.

First autor & year	DM	SE	95% CI		RW
Asgary 2014	-3.640	3.570	-10.638	3.358	0.76
Banini 2006	0.000	4.547	-8.913	8.913	0.50
Basu 2010a	-3.200	1.930	-6.983	0.583	1.92
Basu 2010b	-1.100	3.901	-8.746	6.546	0.65
Basu 2011	1.000	8.124	-14.923	16.923	0.17
Basu 2014	0.000	0.577	-1.132	1.132	4.45
Basu 2014	-4.000	0.577	-5.132	-2.868	4.45
Botden 2012	0.000	2.878	-5.642	5.642	1.08
Botden 2012	0.000	2.781	-5.450	5.450	1.14
Chiva-Blanch 2012	-3.000	3.656	-10.165	4.165	0.73
Chiva-Blanch 2012	-2.000	2.956	-7.793	3.793	1.04
Chiva-Blanch 2012	-2.000	3.628	-9.111	5.111	0.74
Chiva-Blanch 2012	-1.000	2.902	-6.688	4.688	1.07
Clifton 2004	-0.700	3.883	-8.311	6.911	0.66
Curtis 2009	1.000	2.481	-3.862	5.862	1.36
Davinelli 2015	0.700	4.025	-7.188	8.588	0.62
Del Bo 2013	-2.600	4.848	-12.102	6.902	0.44
Draijer 2015	-0.900	15.946	-32.154	30.354	0.04
Draijer 2015	2.500	15.617	-28.108	33.108	0.05
Flammer 2013	-2.800	3.270	-9.210	3.610	0.88
Foster 2012	0.600	0.209	0.190	1.010	5.01
Guo 2014	-3.000	12.530	-27.557	21.557	0.07
Hansen 2005	-1.700	1.817	-5.262	1.862	2.06
Hansen 2005	-1.000	1.454	-3.850	1.850	2.63
Hassellund 2012	1.000	3.742	-6.334	8.334	0.70
Hokayem 2013	3.200	2.454	-1.611	8.011	1.38
Jenkins 2008	-0.500	2.575	-5.547	4.547	1.29
Johnson 2015	-5.000	2.697	-10.286	0.286	1.20
Katz 2012	-2.640	2.322	-7.192	1.912	1.50
Khan 2014	-0.200	3.454	-6.969	6.569	0.81
Khan 2014	-6.070	3.189	-12.320	0.180	0.92
Lamport 2016	3.500	3.631	-3.616	10.616	0.74
Lekakis 2005	-0.800	3.440	-7.543	5.943	0.81
López-Uriarte 2010	-2.000	4.796	-11.400	7.400	0.45
Lynn 2012	-16.000	5.459	-26.699	-5.301	0.36
Lynn 2014	-2.000	3.453	-8.768	4.768	0.81
Ma 2010	4.100	2.455	-0.713	8.913	1.38
McAnulty 2005	-3.000	4.331	-11.489	5.489	0.54
McAnulty 2014	-5.540	4.247	-13.865	2.785	0.56
McKay 2010	-2.500	1.932	-6.286	1.286	1.91
Mukuddem-Petersen 2007	3.000	1.913	-0.750	6.750	1.94
Naissides 2006	-3.000	3.061	-9.000	3.000	0.98
Naissides 2006	1.100	3.423	-5.609	7.809	0.82
Novotny 2015	-8.200	1.907	-11.937	-4.463	1.94
Ohguro 2012	5.300	3.712	-1.976	12.576	0.71
Olmedilla-Alonso 2008	-2.300	5.338	-12.761	8.161	0.37
Parham 2015	0.000	3.399	-6.662	6.662	0.83
Park 2004	-2.650	4.058	-10.603	5.303	0.61
Pérez-Jímenez 2008	-3.800	4.512	-12.643	5.043	0.51
Puupponen-Pimiä 2013	-0.200	3.180	-6.432	6.032	0.92
Puupponen-Pimiä 2013	-1.000	3.774	-8.398	6.398	0.69
Qin 2009	-0.800	1.935	-4.592	2.992	1.91
Queipo-Ortuno 2012	-3.000	2.324	-7.555	1.555	1.49
Queipo-Ortuno 2012	-3.000	1.415	-5.774	-0.226	2.69
Ras 2013	-3.000	5.000	-12.800	6.800	0.42
Riso 2013	-3.600	2.603	-8.701	1.501	1.27
Rodriguez-Mateos 2014	2.000	7.514	-12.728	16.728	0.19
Rodriguez-Mateos 2014	-1.667	7.514	-16.395	13.061	0.19
Rodriguez-Mateos 2014	-2.000	7.113	-15.942	11.942	0.22
Rodriguez-Mateos 2014	-11.900	9.027	-29.593	5.793	0.14
Rodriguez-Mateos 2014	-7.400	9.160	-25.354	10.554	0.13
Ruel 2013	1.400	2.002	-2.524	5.324	1.83
Ruel 2013	-2.200	2.785	-7.658	3.258	1.14
Shema-Didi 2014	-5.000	3.819	-12.485	2.485	0.68
Shema-Didi 2014	-0.500	4.517	-9.353	8.353	0.50
Sivaprakasapillai 2010	0.000	10.607	-20.789	20.789	0.10
Sivaprakasapillai 2010	0.000	2.925	-5.733	5.733	1.06
Spaak 2008	0.500	5.744	-10.759	11.759	0.32
Spaccarotella 2008	-0.950	2.306	-5.469	3.569	1.51
Stull 2010	-1.200	0.734	-2.639	0.239	4.12
Sumner 2005	-5.200	2.246	-9.601	-0.799	1.57
Terauchi 2014a	-5.250	2.668	-10.479	-0.021	1.22
Terauchi 2014a	-2.900	2.463	-7.727	1.927	1.38
Terauchi 2014b	-0.900	3.030	-6.838	5.038	1.00
Tey 2013	-1.800	2.721	-7.132	3.532	1.18
Tey 2013	-0.600	2.097	-4.709	3.509	1.72
Tome-Carneiro 2013	2.000	4.650	-7.114	11.114	0.48
Tsang 2012	-8.000	6.957	-21.635	5.635	0.23
Urquiaga 2015	-1.500	2.621	-6.636	3.636	1.25
Vaisman 2015	1.300	2.626	-3.846	6.446	1.25
Vaisman 2015	-6.900	3.518	-13.794	-0.006	0.78
Watson 2015	1.000	2.721	-4.333	6.333	1.18
Watson 2015	0.170	2.604	-4.933	5.273	1.27
Whelan 2004	-0.500	4.192	-8.716	7.716	0.58
Williams 2004	4.000	4.576	-4.969	12.969	0.49
Williams 2004	-1.000	4.629	-10.073	8.073	0.48
Wu 2015	-3.100	2.155	-7.324	1.124	1.66
Zhang 2015	1.600	1.967	-2.255	5.455	1.87
Zhu 2013	-0.400	1.675	-3.683	2.883	2.26
**Intervention effect**	**-1.417**	**0.338**	**-2.079**	**-0.756**	

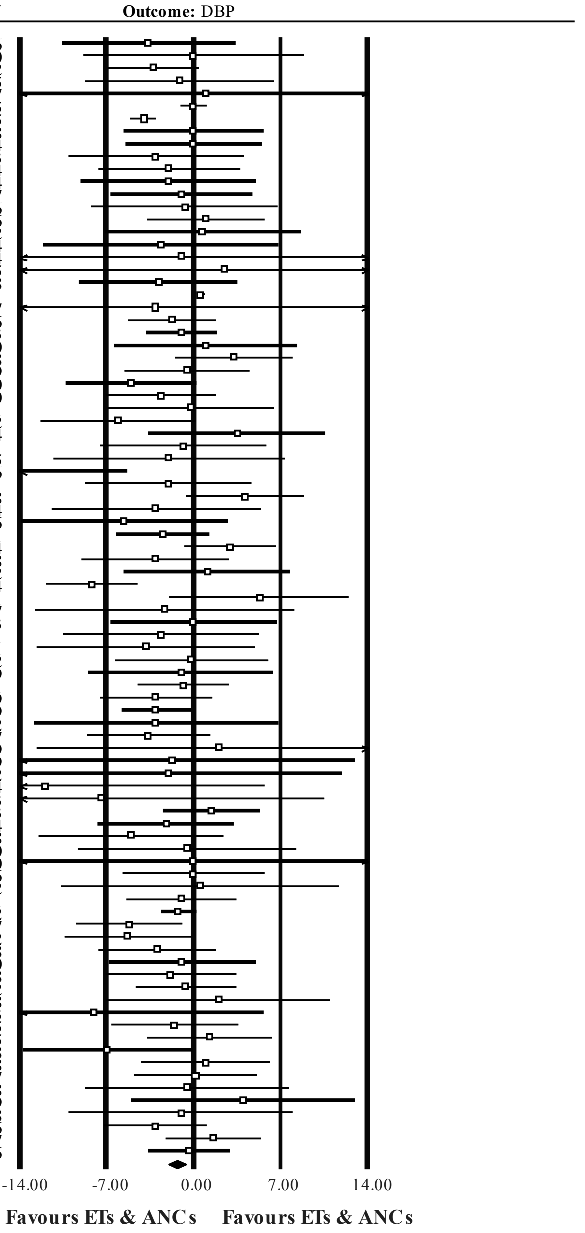

Test for overall effect Z=-4.198; P=0.000; Ns=1782; Nc=1655

Heterogeneity Tau^2=2.301; Q=152.4; df=89 (P=0.000); I^2=41.6

Supplementary Figure S24. Funnel plots and Egger statistics (intercept and 2-tailed *P*-value) of the meta-analysis evaluating the effects of a prolonged supplementation with ellagitannins and anthocyanins-containing products on diastolic blood pressure (DBP).

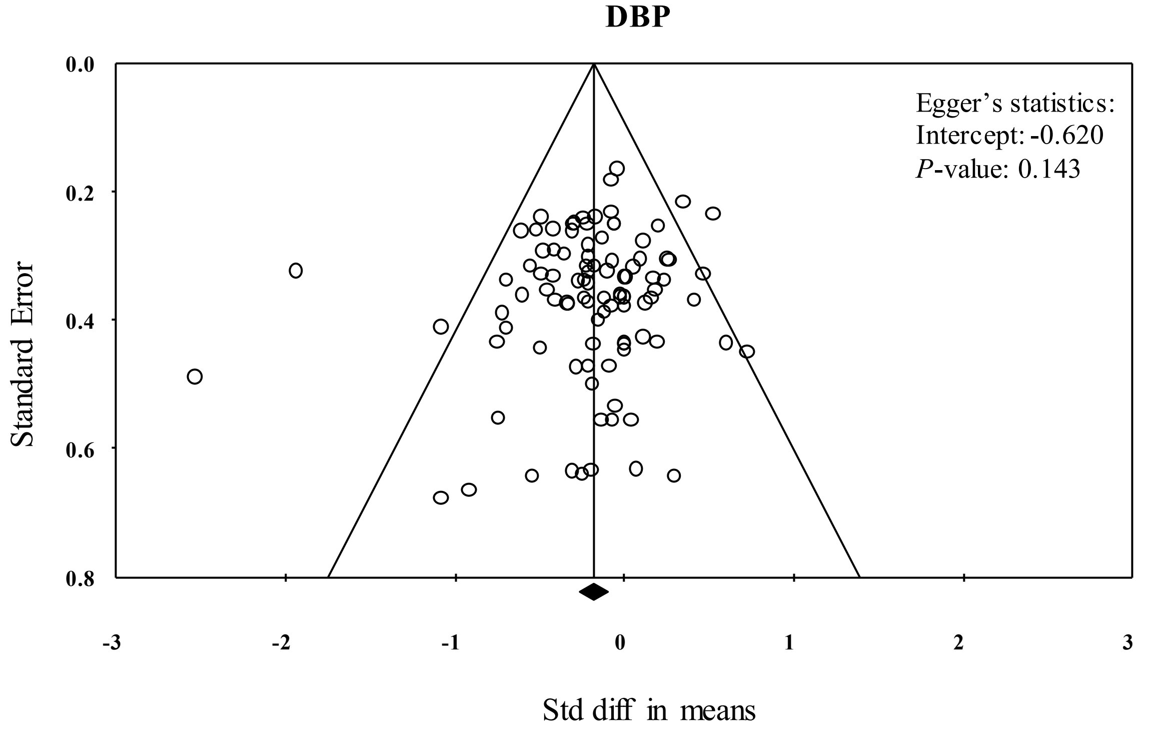

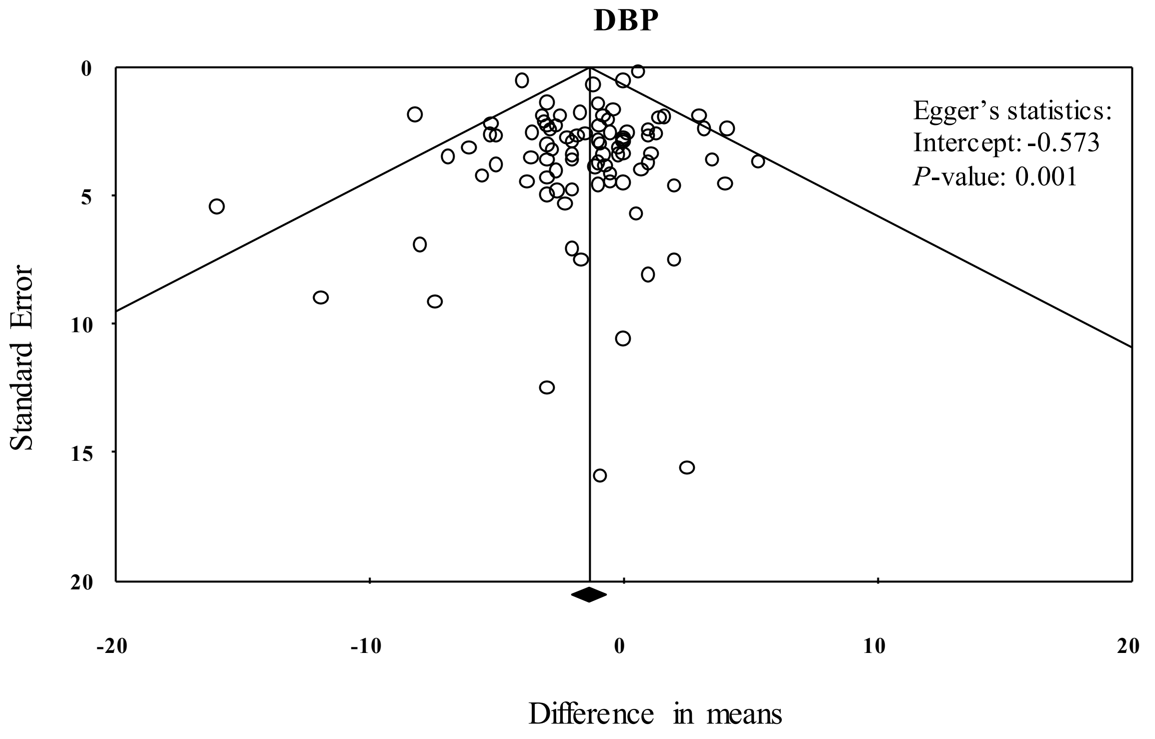

Supplementary Figure S25. Forest plot of the meta-analysis evaluating the effects of a prolonged supplementation with ellagitannins and anthocyanins-containing products on FMD. A total of 22 studies (displayed in alphabetical order) were analysed. Pooled results are shown at the bottom using a random-effects model. SDM: standardized difference in means, SE: standard error, 95% CI: lower and upper confidence limits for the average SDM, RW: relative weight.

First autor & year	SDM	SE	95% CI		RW
Asgary 2014	0.714	0.451	-0.169	1.597	3.92
Clifton 2004	0.370	0.336	-0.289	1.029	7.05
Cuevas 2000	0.258	0.820	-1.348	1.865	1.19
Cuevas 2000	1.991	1.109	-0.182	4.164	0.65
Del Bo 2013	-0.458	0.641	-1.713	0.798	1.94
Del Bo 2014	-2.202	0.634	-3.444	-0.960	1.98
Hijmering 2007	-0.565	0.456	-1.459	0.330	3.82
Karatzi 2004	-1.452	0.584	-2.597	-0.307	2.34
Karatzi 2004	-1.270	0.567	-2.382	-0.158	2.48
Katz 2012	0.550	0.416	-0.265	1.365	4.61
Khan 2014	0.721	0.318	0.097	1.346	7.86
Khan 2014	0.612	0.312	0.001	1.224	8.18
Lekakis 2005	0.795	0.381	0.048	1.542	5.49
Ma 2010	0.607	0.447	-0.269	1.483	3.99
Rodriguez-Mateos 2014	0.791	0.664	-0.511	2.093	1.81
Rodriguez-Mateos 2014	0.575	0.648	-0.694	1.845	1.90
Rodriguez-Mateos 2014	1.156	0.700	-0.216	2.527	1.63
Spaak 2006	-0.133	0.557	-1.225	0.958	2.57
Vaisman 2008	-0.266	0.423	-1.096	0.564	4.45
Vaisman 2015	0.633	0.363	-0.078	1.345	6.05
Whelan 2004	-0.651	0.597	-1.821	0.519	2.24
Zhu 2013	1.323	0.183	0.965	1.681	23.87
**Intervention effect**	**0.204**	**0.189**	**-0.166**	**0.574**	

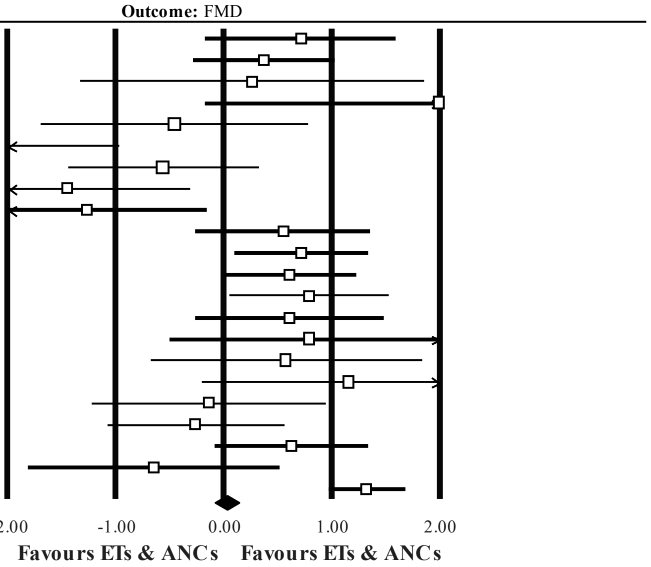

Test for overall effect Z=1.080; P=0.280; Ns=278; Nc=285

Heterogeneity Tau^2=0.520; Q=80.1; df=21 (P=0.000); I^2=73.8

Supplementary Figure S26.- Forest plot of the meta-analysis evaluating the effects of a prolonged supplementation with ellagitannins and anthocyanins-containing products on FMD. A total of 21 studies (displayed in alphabetical order) were analysed. Pooled results are shown at the bottom using a random-effects model. DM: Difference in means, SE: standard error, 95% CI: lower and upper confidence limits for the average DM, RW: relative weight.

First autor & year	DM	SE	95% CI		RW
Asgary 2014	0.050	0.032	-0.012	0.112	94.59
Clifton 2004	0.500	0.442	-0.367	1.367	0.49
Cuevas 2000	2.700	8.529	-14.016	19.416	0.00
Cuevas 2000	9.500	4.356	0.963	18.037	0.01
Del Bo 2013	-0.110	0.196	-0.495	0.275	2.47
Hijmering 2007	-1.800	1.600	-4.936	1.336	0.04
Karatzi 2004	-4.030	1.394	-6.762	-1.298	0.05
Karatzi 2004	-3.120	1.252	-5.574	-0.666	0.06
Katz 2012	1.100	0.817	-0.501	2.701	0.14
Khan 2014	2.000	0.843	0.347	3.653	0.13
Khan 2014	1.600	0.776	0.079	3.121	0.16
Lekakis 2005	1.453	0.645	0.189	2.718	0.23
Ma 2010	1.000	0.720	-0.411	2.411	0.18
Rodriguez-Mateos 2014	1.200	0.931	-0.625	3.025	0.11
Rodriguez-Mateos 2014	0.967	0.959	-0.913	2.847	0.10
Rodriguez-Mateos 2014	1.400	0.787	-0.143	2.943	0.15
Spaak 2006	-0.650	2.568	-5.683	4.383	0.01
Vaisman 2008	-0.900	1.642	-4.119	2.319	0.04
Vaisman 2015	1.910	1.167	-0.376	4.196	0.07
Whelan 2004	-1.160	1.206	-3.524	1.204	0.07
Zhu 2013	2.660	0.327	2.019	3.301	0.89
**Intervention effect**	**0.635**	**0.287**	**0.073**	**1.197**	

Outcome: FMD

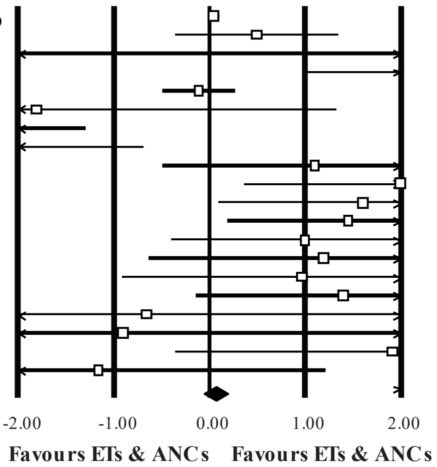

Test for overall effect Z=2.214; *P*=0.027; Ns=270; Nc=277

Heterogeneity Tau^2=0.841; Q=112.1; df=20 (*P*=0.000); I^2=82.2

Supplementary Figure S27. Funnel plots and Egger statistics (intercept and 2-tailed *P*-value) of the meta-analysis evaluating the effects of a prolonged supplementation with ellagitannins and anthocyanins-containing products on flow mediated dilation (FMD).

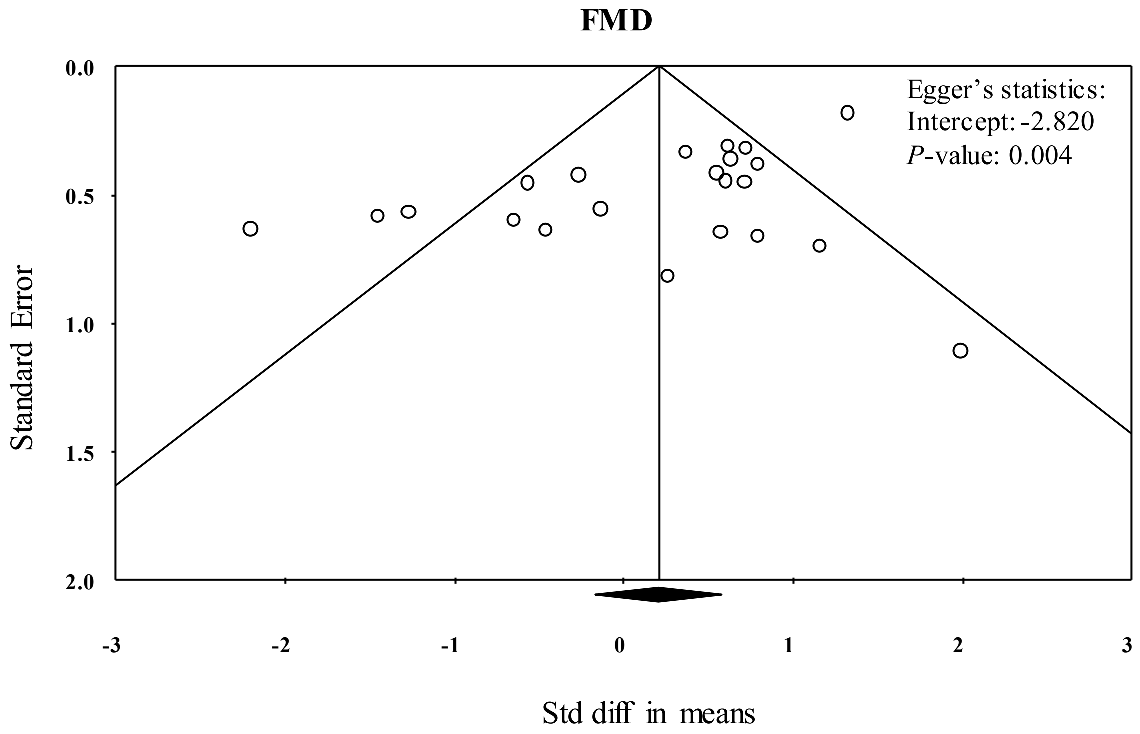

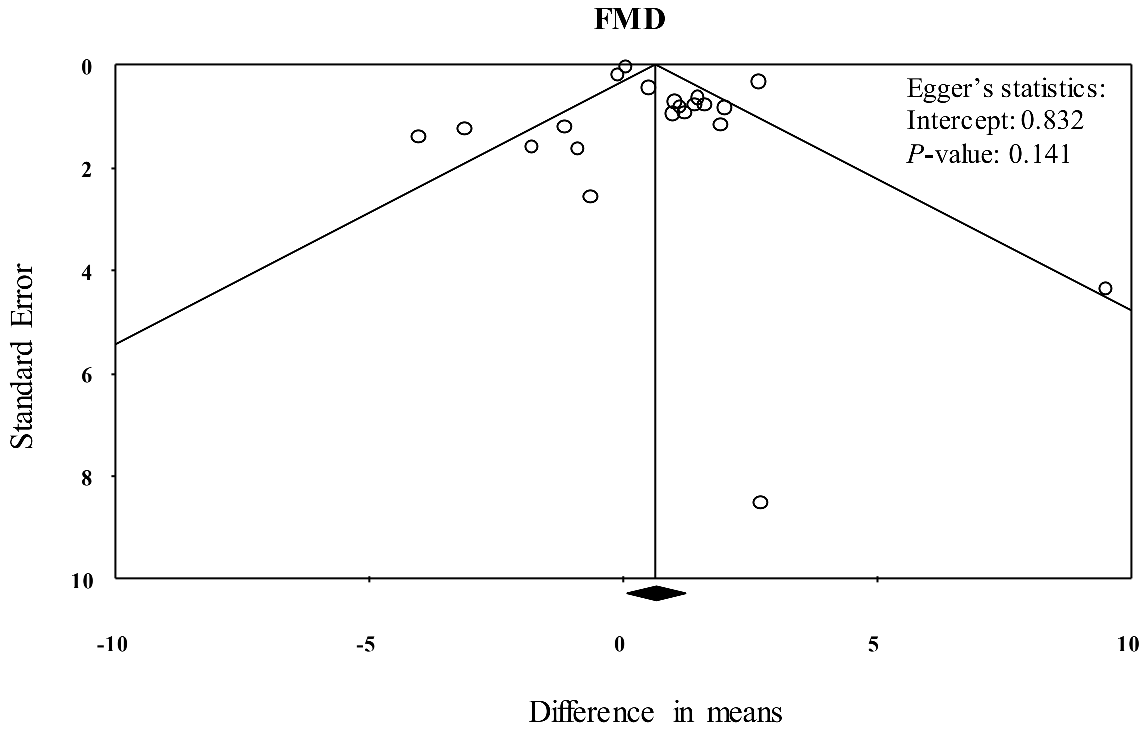

Supplementary Figure S28. Forest plot of the meta-analysis evaluating the effects of a prolonged supplementation with ellagitannins and anthocyanins-containing products on glucose. A total of 67 studies (displayed in alphabetical order) were analysed. Pooled results are shown at the bottom using a random-effects model. SDM: standardized difference in means, SE: standard error, 95% CI: lower and upper confidence limits for the average SDM, RW: relative weight.

First autor & year	SDM	SE	95% CI		RW
Banini 2006	0.317	0.440	1.180	-0.546	0.92
Basu 2010a	0.295	0.290	0.864	-0.275	2.11
Basu 2010b	-0.184	0.388	0.577	-0.945	1.18
Basu 2011	0.536	0.366	1.253	-0.181	1.33
Basu 2014	-0.660	0.375	0.075	-1.395	1.27
Basu 2014	-0.447	0.370	0.278	-1.171	1.30
Bommannan Santhakumar 2015	0.324	0.560	1.422	-0.773	0.57
Bommannan Santhakumar 2015	0.537	0.566	1.646	-0.573	0.56
Brennan 2010	0.170	0.519	1.187	-0.848	0.66
Castilla 2006	0.123	0.349	0.808	-0.561	1.46
Cerdá 2006	0.078	0.365	0.794	-0.638	1.33
Cook 2015	-0.160	0.535	0.889	-1.209	0.62
Curtis 2009	-0.392	0.280	0.157	-0.941	2.27
Edirisinghe 2011	0.000	0.408	0.800	-0.800	1.07
Ellis 2011	0.577	0.417	1.394	-0.239	1.03
Hassellund 2013	0.926	0.405	1.720	0.131	1.08
Heber 2007	0.383	0.425	1.216	-0.450	0.99
Heber 2007	-0.085	0.424	0.747	-0.916	0.99
Hijmering 2007	-1.712	0.523	-0.687	-2.737	0.65
Hoggard 2013	-3.394	1.105	-1.229	-5.559	0.15
Hokayem 2013	0.150	0.325	0.787	-0.488	1.68
Hollis 2013	0.385	0.283	0.939	-0.169	2.23
Hosseini 2016	-0.531	0.314	0.084	-1.147	1.81
Jin 2011	0.165	0.448	1.043	-0.713	0.89
Johnston 2013	-0.331	0.307	0.271	-0.933	1.89
Katz 2012	-0.165	0.409	0.637	-0.966	1.06
Kay 2002	-0.564	0.721	0.849	-1.977	0.34
Kianbakht 2013	-0.942	0.245	-0.461	-1.422	2.96
Kolehmainen 2012	-0.284	0.389	0.479	-1.047	1.17
Krikorian 2010	-0.381	0.591	0.777	-1.539	0.51
Krikorian 2012	-0.439	0.442	0.427	-1.306	0.91
Lee 2014	0.080	0.258	0.587	-0.426	2.67
Lekakis 2005	-0.042	0.251	0.450	-0.534	2.82
Li 2010	-0.892	0.285	-0.333	-1.452	2.19
Ma 2010	0.339	0.440	1.201	-0.524	0.92
Moazen 2013	-0.017	0.334	0.638	-0.671	1.60
Naissides 2006	0.614	0.374	1.348	-0.120	1.27
Naissides 2006	0.303	0.361	1.012	-0.405	1.36
Novotny 2015	-1.007	0.284	-0.451	-1.564	2.21
Parham 2015	-0.653	0.448	0.226	-1.532	0.89
Park 2014	-0.022	0.228	0.425	-0.469	3.43
Pérez-Jímenez 2008	-0.144	0.375	0.591	-0.879	1.26
Qin 2009	-0.078	0.183	0.280	-0.436	5.34
Queipo-Ortuno 2012	-0.205	0.634	1.037	-1.448	0.44
Queipo-Ortuno 2012	-0.018	0.632	1.221	-1.258	0.44
Rahbar 2015	0.227	0.299	0.814	-0.359	1.99
Rahbar 2015	0.675	0.300	1.263	0.087	1.98
Riso 2013	-0.050	0.471	0.874	-0.974	0.80
Santhakumar 2015	0.537	0.566	1.646	-0.573	0.56
Stull 2010	0.000	0.354	0.694	-0.694	1.42
Sumner 2005	0.021	0.317	0.642	-0.600	1.77
Tomé-Carneiro 2012	0.257	0.284	0.814	-0.299	2.21
Tome-Carneiro 2013	-0.140	0.434	0.711	-0.991	0.94
Tsang 2012	-0.404	0.382	0.344	-1.153	1.22
Urquiaga 2015	-0.183	0.343	0.489	-0.854	1.52
Vaisman 2015	-0.178	0.355	0.518	-0.873	1.41
Vaisman 2015	-0.041	0.422	0.785	-0.868	1.00
Watson 2015	0.080	0.481	1.023	-0.863	0.77
Watson 2015	0.104	0.473	1.031	-0.823	0.80
Whelan 2004	-0.606	0.553	0.478	-1.690	0.58
Wien 2010	-0.008	0.273	0.527	-0.542	2.39
Williams 2004	-0.599	0.572	0.521	-1.719	0.54
Wu 2014	-1.644	0.366	-0.927	-2.361	1.33
Yubero 2013	-0.107	0.258	0.399	-0.614	2.66
Zhang 2015	0.199	0.253	0.696	-0.297	2.77
Zhu 2011	0.063	0.166	0.388	-0.261	6.49
Zunino 2014	-0.536	0.416	0.279	-1.350	1.03
**Intervention effect**	**-0.101**	**0.060**	**-0.219**	**0.017**	

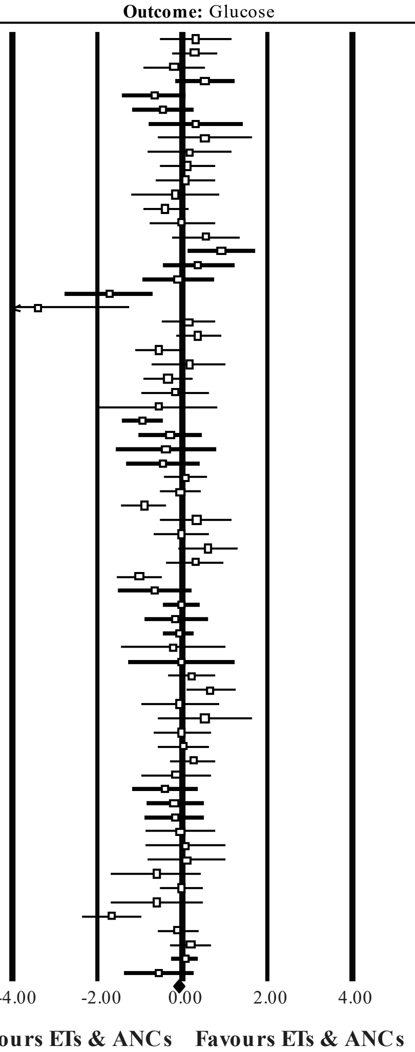

Test for overall effect Z=-1.671; P=0.095; Ns=1236; Nc=1133

Heterogeneity Tau^2=0.103; Q=122.3; df=66 (P=0.000); I^2=46.1

Supplementary Figure S29.- Forest plot of the meta-analysis evaluating the effects of a prolonged supplementation with ellagitannins and anthocyanins-containing products on glucose. A total of 60 studies (displayed in alphabetical order) were analysed. Pooled results are shown at the bottom using a random-effects model. DM: Difference in means, SE: standard error, 95% CI: lower and upper confidence limits for the average DM, RW: relative weight.

First autor & year	DM	SE	95% CI		RW
Banini 2006	0.161	0.281	0.712	-0.390	0.28
Basu 2010a	0.200	0.196	0.584	-0.184	0.57
Basu 2010b	-0.100	0.264	0.417	-0.617	0.31
Basu 2011	0.256	0.165	0.579	-0.068	0.80
Basu 2014	-0.110	0.061	0.010	-0.230	5.81
Basu 2014	-0.111	0.087	0.060	-0.281	2.89
Bommannan Santhakumar 2015	0.200	0.493	1.166	-0.766	0.09
Bommannan Santhakumar 2015	0.400	0.369	1.123	-0.323	0.16
Brennan 2010	0.126	0.419	0.948	-0.695	0.12
Castilla 2006	0.130	0.356	0.827	-0.567	0.17
Cerdá 2006	0.204	0.906	1.979	-1.571	0.03
Curtis 2009	-0.200	0.134	0.064	-0.464	1.21
Edirisinghe 2011	0.000	0.251	0.491	-0.491	0.35
Ellis 2011	0.200	0.141	0.477	-0.077	1.09
Heber 2007	0.144	0.175	0.487	-0.199	0.71
Heber 2007	-0.055	0.260	0.454	-0.564	0.32
Hijmering 2007	-2.300	0.555	-1.212	-3.388	0.07
Hokayem 2013	0.100	0.217	0.525	-0.325	0.46
Hollis 2013	0.330	0.224	0.770	-0.110	0.43
Hosseini 2016	-0.394	0.224	0.046	-0.833	0.43
Johnston 2013	-0.349	0.322	0.282	-0.980	0.21
Katz 2012	-0.072	0.178	0.277	-0.421	0.69
Kay 2002	-0.310	0.373	0.421	-1.041	0.16
Kianbakht 2013	-2.158	0.480	-1.216	-3.099	0.09
Kolehmainen 2012	-0.200	0.267	0.323	-0.723	0.31
Krikorian 2012	-0.222	0.228	0.225	-0.669	0.42
Lee 2014	0.033	0.106	0.242	-0.175	1.93
Lekakis 2005	-0.400	2.669	4.832	-5.632	0.00
Li 2010	-0.481	0.142	-0.203	-0.759	1.09
Ma 2010	0.395	0.509	1.392	-0.603	0.08
Moazen 2013	-0.059	1.264	2.418	-2.536	0.01
Naissides 2006	0.180	0.113	0.402	-0.042	1.71
Naissides 2006	0.090	0.132	0.349	-0.169	1.25
Novotny 2015	-0.160	0.099	0.034	-0.354	2.22
Parham 2015	-0.888	0.594	0.276	-2.051	0.06
Park 2014	-0.190	1.981	3.692	-4.072	0.01
Pérez-Jiménez 2008	-0.065	0.188	0.304	-0.434	0.62
Qin 2009	-0.160	0.341	0.508	-0.828	0.19
Queipo-Ortuno 2012	-0.239	0.781	1.291	-1.769	0.04
Queipo-Ortuno 2012	-0.017	0.725	1.404	-1.438	0.04
Rahbar 2015	0.132	0.186	0.497	-0.232	0.63
Rahbar 2015	0.467	0.204	0.868	0.067	0.52
Riso 2013	-0.100	1.049	1.956	-2.156	0.02
Santhakumar 2015	0.400	0.369	1.123	-0.323	0.16
Stull 2010	0.000	0.142	0.278	-0.278	1.08
Sumner 2005	0.055	0.774	1.571	-1.461	0.04
Tomé-Carneiro 2012	0.610	0.613	1.812	-0.592	0.06
Tome-Carneiro 2013	-0.276	1.300	2.272	-2.824	0.01
Tsang 2012	-0.170	0.168	0.159	-0.499	0.77
Urquiaga 2015	-0.073	0.126	0.174	-0.319	1.38
Vaisman 2015	-0.132	0.247	0.353	-0.617	0.36
Vaisman 2015	-0.027	0.289	0.540	-0.594	0.26
Watson 2015	0.155	0.308	0.759	-0.449	0.23
Watson 2015	0.090	0.274	0.627	-0.447	0.29
Whelan 2004	-0.400	0.578	0.733	-1.533	0.07
Williams 2004	-0.400	0.521	0.622	-1.422	0.08
Wu 2014	-0.111	0.021	-0.069	-0.153	48.02
Yubero 2013	-0.015	0.035	0.054	-0.084	17.61
Zhang 2015	0.150	0.176	0.495	-0.195	0.70
Zhu 2011	0.100	0.266	0.621	-0.422	0.31
**Intervention effect**	**-0.033**	**0.001**	**-0.093**	**0.027**	

Outcome: Glucose

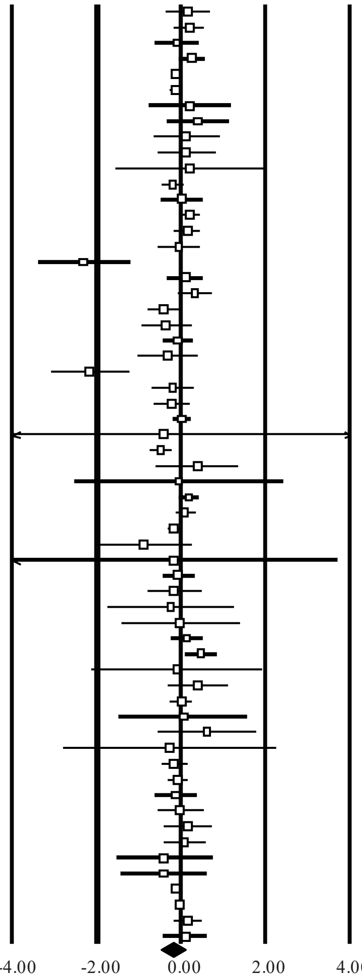

Test for overall effect Z=-1.086; *P*=0.277; Ns=1156; Nc=1054

Heterogeneity Tau^2=0.012; Q=99.4; df=59 (*P*=0.001); I^2=40.6

Supplementary Figure S30. Funnel plots and Egger statistics (intercept and 2-tailed *P*-value) of the meta-analysis evaluating the effects of a prolonged supplementation with ellagitannins and anthocyanins-containing products on glucose.

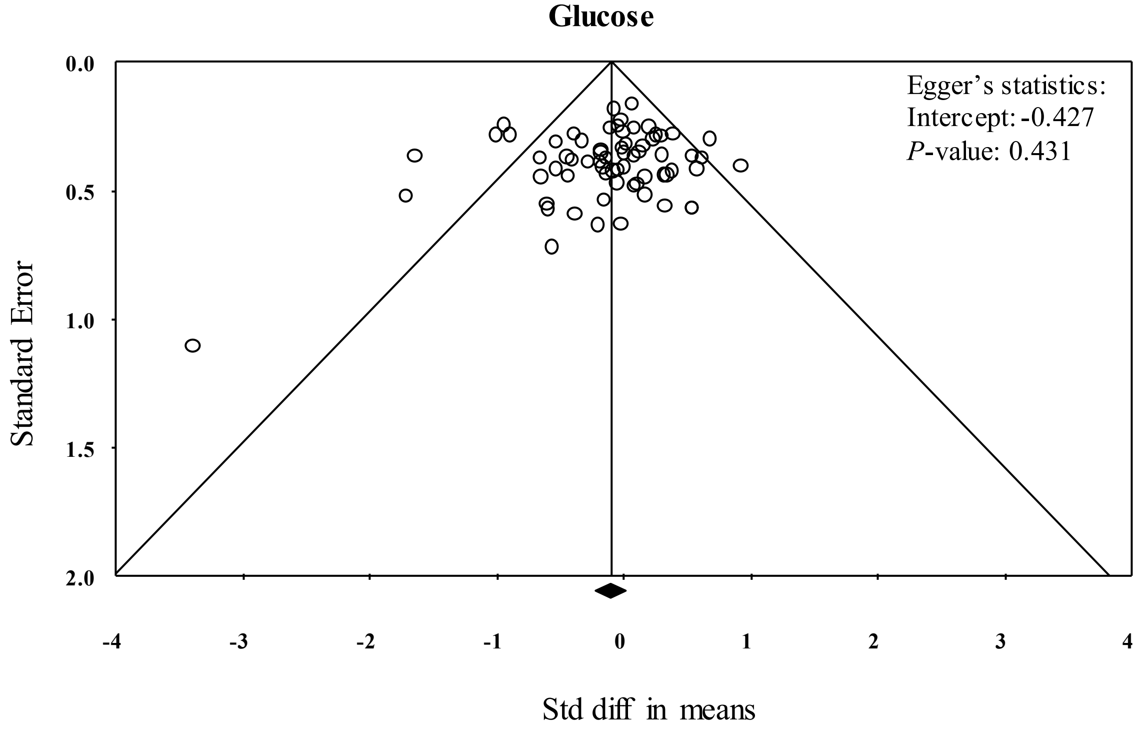

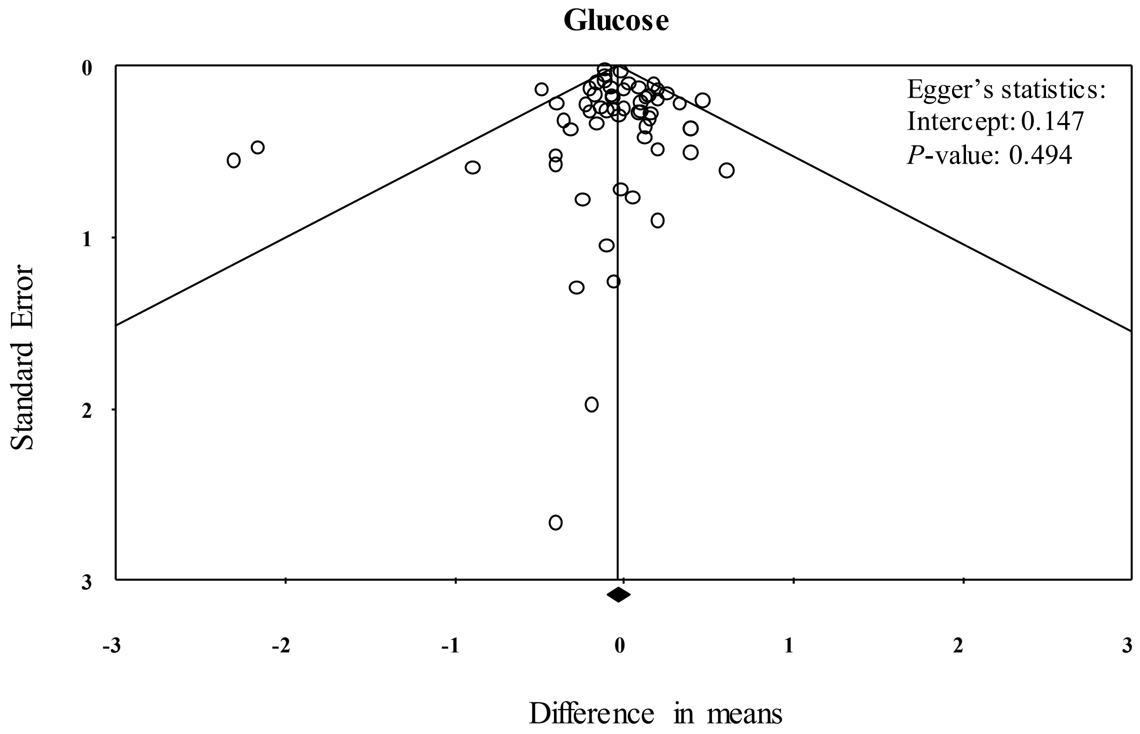

Supplementary Figure S31. Forest plot of the meta-analysis evaluating the effects of a prolonged supplementation with ellagitannins and anthocyanins-containing products on insulin. A total of 34 studies (displayed in alphabetical order) were analysed. Pooled results are shown at the bottom using a random-effects model. SDM: standardized difference in means, SE: standard error, 95% CI: lower and upper confidence limits for the average SDM, RW: relative weight.

First autor & year	SDM	SE	95% CI		RW
Banini 2006	0.599	0.529	-0.438	1.636	1.21
Basu 2014	-0.081	0.365	-0.797	0.635	2.54
Basu 2014	1.480	0.659	0.189	2.771	0.78
Brennan 2010	-0.041	0.251	-0.533	0.452	5.38
Edirisinghe 2004	0.071	0.268	-0.453	0.595	4.74
Ellis 2011	0.363	0.412	-0.443	1.170	2.00
Hassellund 2013	-0.608	0.394	-1.380	0.164	2.18
Hoggard 2013	0.169	0.253	-0.328	0.665	5.29
Hokayem 2012	-2.302	0.471	-3.225	-1.379	1.53
Hosseini 2016	0.760	0.452	-0.127	1.646	1.66
Jin 2011	-0.180	0.273	-0.714	0.355	4.56
Johnston 2013	-0.441	0.364	-1.154	0.272	2.56
Johnston 2013	-0.114	0.307	-0.715	0.487	3.61
Katz 2012	-0.908	0.324	-1.543	-0.273	3.23
Kolehmainen 2015	-1.140	0.350	-1.827	-0.454	2.76
Krikorian 2010	0.346	0.440	-0.517	1.209	1.75
Krikorian 2012	0.030	0.354	-0.664	0.724	2.70
Lee 2014	-0.119	0.438	-0.978	0.740	1.77
Lekakis 2005	0.070	0.166	-0.254	0.395	12.37
Li 2010	-0.091	0.258	-0.597	0.416	5.08
Ma 2010	-2.038	0.871	-3.746	-0.330	0.45
Naissides 2006	0.129	0.366	-0.589	0.847	2.53
Naissides 2006	1.265	0.532	0.222	2.308	1.20
Naissides 2006	-1.151	0.441	-2.015	-0.287	1.75
Novotny 2011	0.151	0.388	-0.610	0.911	2.25
Park 2014	-0.279	0.380	-1.023	0.466	2.35
Stull 2010	-0.154	0.273	-0.690	0.381	4.54
Tsang 2012	-0.096	0.408	-0.896	0.705	2.03
Vaisman 2015	0.566	0.429	-0.276	1.408	1.84
Wien 2010	0.094	0.408	-0.706	0.895	2.03
Wu 2014	1.203	0.562	0.101	2.304	1.07
Zhang 2015	1.233	0.488	0.276	2.189	1.42
Zhu 2011	-0.463	0.231	-0.915	-0.010	6.35
Zunino 2014	0.779	0.367	0.059	1.499	2.51
**Intervention effect**	**-0.042**	**0.105**	**-0.248**	**0.164**	

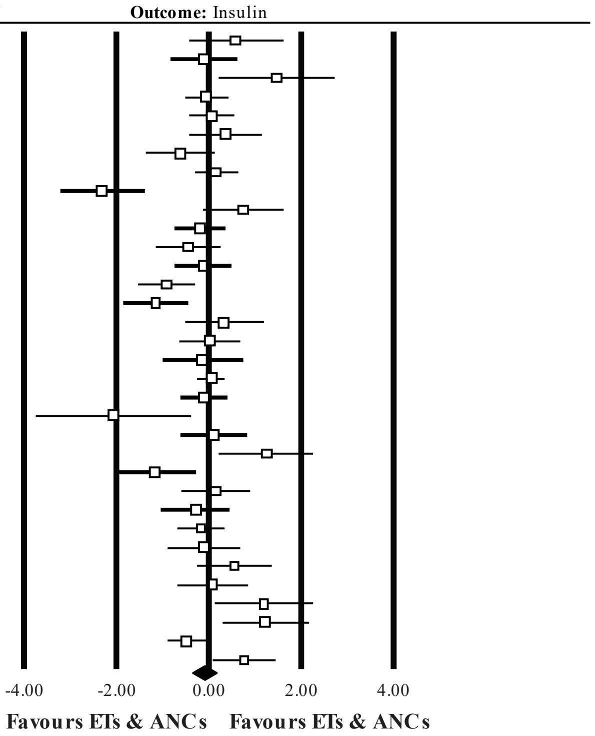

Test for overall effect Z=-0.402; *P*=0.688; Ns=612; Nc=624

Heterogeneity Tau²=0.229; Q=97.3; df=33 (*P*=0.000); I²=66.1

Supplementary Figure S32.- Forest plot of the meta-analysis evaluating the effects of a prolonged supplementation with ellagitannins and anthocyanins-containing products on insulin. A total of 24 studies (displayed in alphabetical order) were analysed. Pooled results are shown at the bottom using a random-effects model. DM: Difference in means, SE: standard error, 95% CI: lower and upper confidence limits for the average DM, RW: relative weight.

First autor & year	DM	SE	95% CI		RW
Basu 2014	-0.300	1.318	-2.883	2.283	0.81
Brennan 2010	-0.900	5.811	-12.290	10.490	0.04
Edirisinghe 2004	0.210	1.005	-1.761	2.181	1.38
Hoggard 2013	0.260	0.363	-0.451	0.971	10.63
Hokayem 2012	-3.800	0.952	-5.666	-1.934	1.54
Hosseini 2016	7.000	4.026	-0.892	14.892	0.09
Jin 2011	-1.200	3.002	-7.084	4.684	0.16
Johnston 2013	-0.870	1.044	-2.916	1.176	1.28
Katz 2012	-3.700	1.370	-6.386	-1.014	0.74
Kolehmainen 2015	-0.400	0.133	-0.661	-0.139	78.98
Krikorian 2010	2.000	2.860	-3.605	7.605	0.17
Krikorian 2012	0.333	3.751	-7.018	7.684	0.10
Lee 2014	-0.700	3.723	-7.998	6.598	0.10
Li 2010	-0.330	0.940	-2.173	1.513	1.58
Naissides 2006	0.560	1.535	-2.448	3.568	0.59
Novotny 2011	1.200	3.423	-5.509	7.909	0.12
Park 2014	-1.724	2.661	-6.940	3.492	0.20
Stull 2010	-3.250	5.742	-14.504	8.004	0.04
Tsang 2012	-1.400	5.970	-13.101	10.301	0.04
Vaisman 2015	4.800	3.710	-2.472	12.072	0.10
Wien 2010	1.067	4.025	-6.821	8.955	0.09
Wu 2014	3.290	1.416	0.515	6.065	0.70
Zhu 2011	-4.550	2.242	-8.944	-0.156	0.28
Zunino 2014	5.400	2.441	0.615	10.185	0.23
**Intervention effect**	**-0.348**	**0.382**	**-1.097**	**0.401**	

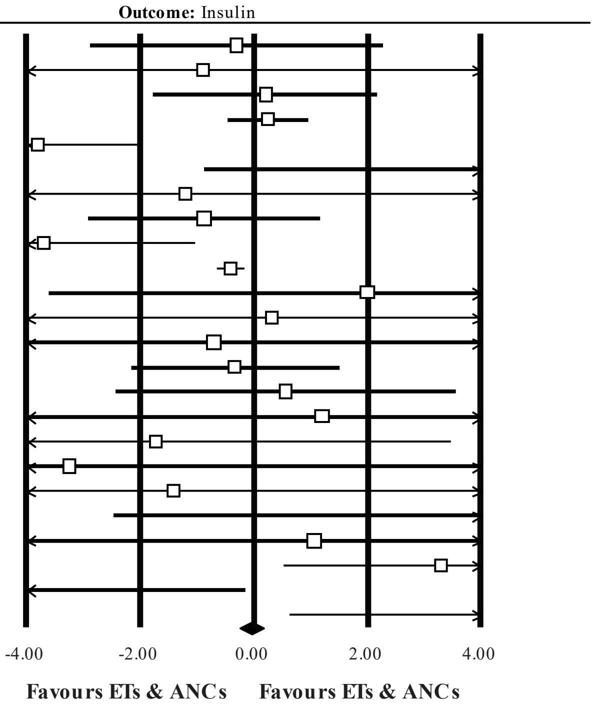

Test for overall effect Z=-0.911; *P*=0.362; Ns=446; Nc=454

Heterogeneity Tau²=0.869; Q=45.6; df=23 (*P*=0.003); I^2=49.6

Supplementary Figure S33. Funnel plots and Egger statistics (intercept and 2-tailed *P*-value) of the meta-analysis evaluating the effects of a prolonged supplementation with ellagitannins and anthocyanins-containing products on insulin.

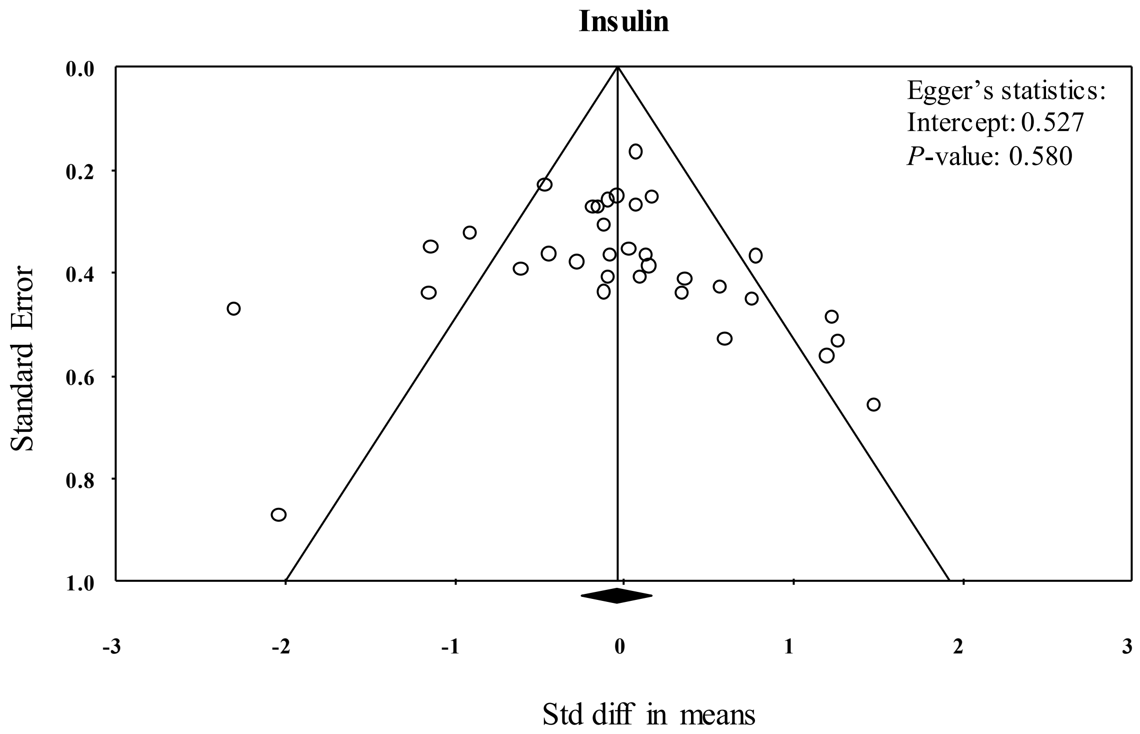

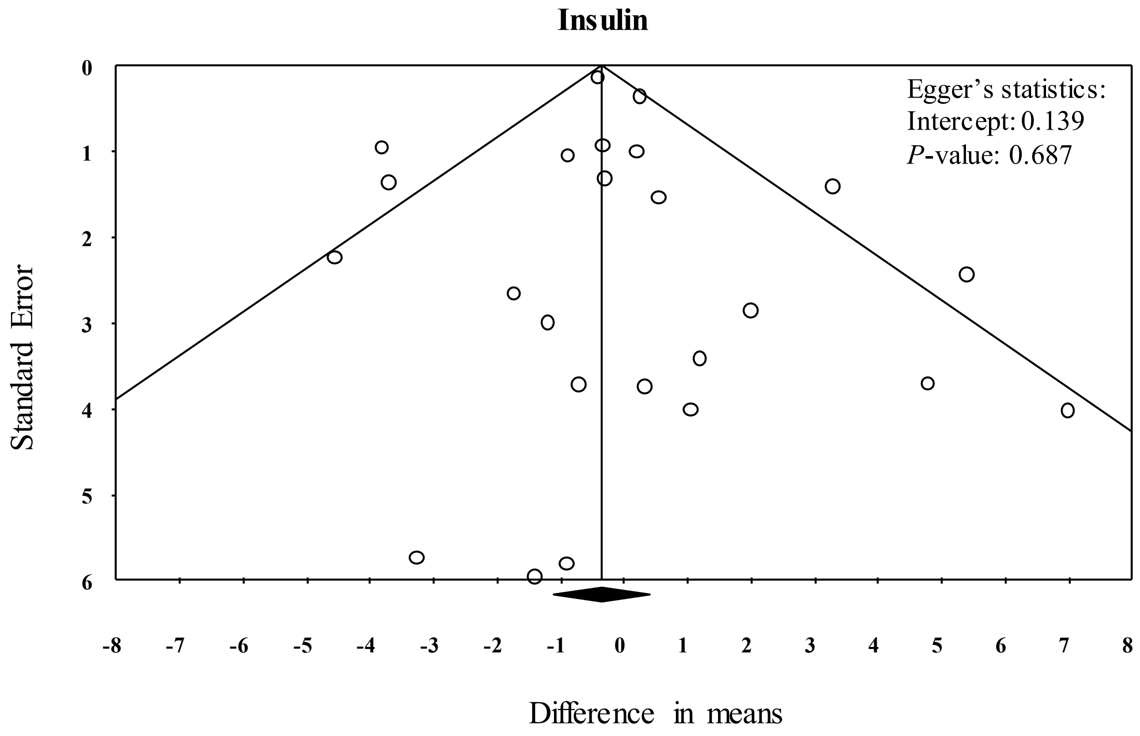

Supplementary Figure S34. Forest plot of the meta-analysis evaluating the effects of a prolonged supplementation with ellagitannins and anthocyanins-containing products on Hb1Ac. A total of 20 studies (displayed in alphabetical order) were analysed. Pooled results are shown at the bottom using a random-effects model. SDM: standardized difference in means, SE: standard error, 95% CI: lower and upper confidence limits for the average SDM, RW: relative weight.

First autor & year	SDM	SE	95% CI		RW
Banini 2006	0.750	0.452	-0.135	1.635	3.03
Basu 2010a	0.204	0.290	-0.364	0.772	7.36
Basu 2014	-3.000	0.532	-4.043	-1.957	2.18
Basu 2014	-0.632	0.374	-1.366	0.101	4.41
Hassellund 2013	-0.126	0.386	-0.882	0.630	4.15
Hokayem 2013	5.000	0.659	3.708	6.292	1.42
Johnston 2013	-0.148	0.305	-0.746	0.451	6.62
Kianbakht 2013	-0.996	0.349	-1.679	-0.312	5.08
Lee 2014	0.351	0.260	-0.159	0.861	9.12
Lekakis 2005	-0.021	0.251	-0.513	0.472	9.79
Ma 2010	0.000	0.437	-0.856	0.856	3.23
Moazen 2013	-0.375	0.337	-1.035	0.285	5.44
Parham 2015	1.015	0.464	0.106	1.925	2.87
Pérez-Jimenez 2008	1.072	0.392	0.303	1.841	4.01
Sumner 2005	-0.168	0.318	-0.790	0.454	6.12
Tome-Carneiro 2013	0.267	0.435	-0.587	1.120	3.26
Urquiaga 2015	0.333	0.344	-0.341	1.008	5.22
Vaisman 2015	0.000	0.422	-0.826	0.826	3.47
Vaisman 2015	0.000	0.354	-0.694	0.694	4.92
Wien 2010	-0.067	0.273	-0.602	0.468	8.29
**Intervention effect**	**0.121**	**0.202**	**-0.275**	**0.517**	

Outcome: Hb1Ac

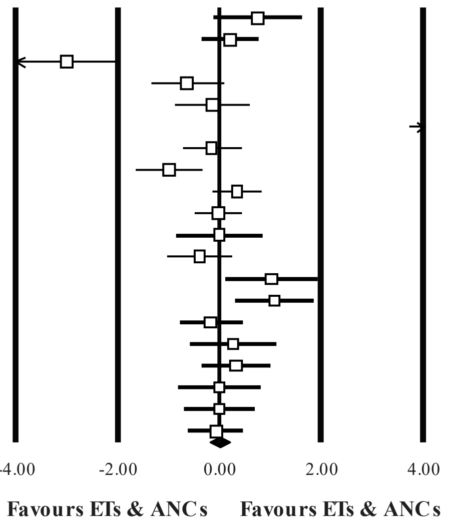

Test for overall effect Z=0.600; *P*=0.549; Ns=387; Nc=345

Heterogeneity Tau^2=0.668; Q=120.6; df=19 (*P*=0.000); I^2=82.2

Supplementary Figure S35.- Forest plot of the meta-analysis evaluating the effects of a prolonged supplementation with ellagitannins and anthocyanins-containing products on Hb1Ac. A total of 19 studies (displayed in alphabetical order) were analysed. Pooled results are shown at the bottom using a random-effects model. DM: Difference in means, SE: standard error, 95% CI: lower and upper confidence limits for the average DM, RW: relative weight.

First autor & year	DM	SE	95% CI		RW
Banini 2006	0.600	0.412	-0.208	1.408	0.24
Basu 2010a	0.100	0.142	-0.178	0.378	2.06
Basu 2014	-0.300	0.052	-0.401	-0.199	15.54
Basu 2014	-0.100	0.052	-0.201	0.001	15.54
Hokayem 2013	0.500	0.032	0.436	0.564	39.25
Johnston 2013	-0.070	0.145	-0.353	0.213	1.98
Kianbakht 2013	-0.800	0.295	-1.378	-0.222	0.48
Lee 2014	0.080	0.059	-0.035	0.195	11.99
Lekakis 2005	-0.200	2.387	-4.879	4.479	0.01
Ma 2010	0.000	0.131	-0.257	0.257	2.41
Moazen 2013	-0.620	0.572	-1.741	0.501	0.13
Parham 2015	0.400	0.172	0.063	0.737	1.40
Pérez-Jimenez 2008	0.470	0.158	0.160	0.780	1.66
Sumner 2005	-0.240	0.464	-1.148	0.668	0.19
Tome-Carneiro 2013	0.300	0.502	-0.685	1.285	0.16
Urquiaga 2015	0.100	0.103	-0.101	0.301	3.94
Vaisman 2015	0.000	0.193	-0.379	0.379	1.11
Vaisman 2015	0.000	0.152	-0.297	0.297	1.80
Wien 2010	-0.150	0.611	-1.348	1.048	0.11
**Intervention effect**	**0.047**	**0.096**	**-0.143**	**0.236**	

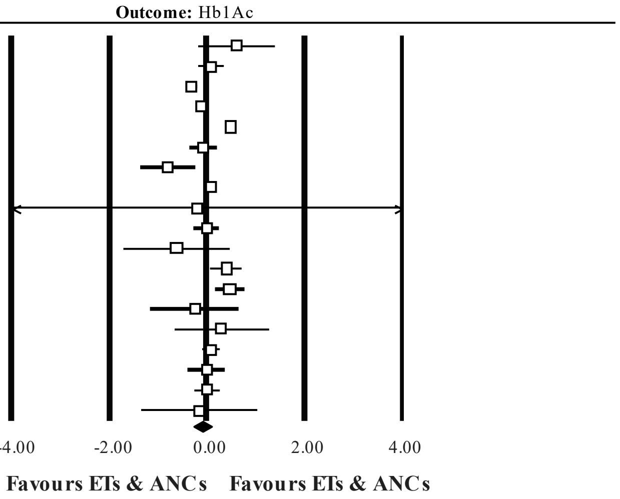

Test for overall effect Z=0.482; P=0.629; Ns=374; Nc=331

Heterogeneity Tau^2=0.120; Q=242.9; df=18 (P=0.000); I^2=92.6

Supplementary Figure S36. Funnel plots and Egger statistics (intercept and 2-tailed *P*-value) of the meta-analysis evaluating the effects of a prolonged supplementation with ellagitannins and anthocyanins-containing products on glycated haemoglobin (Hb1Ac).

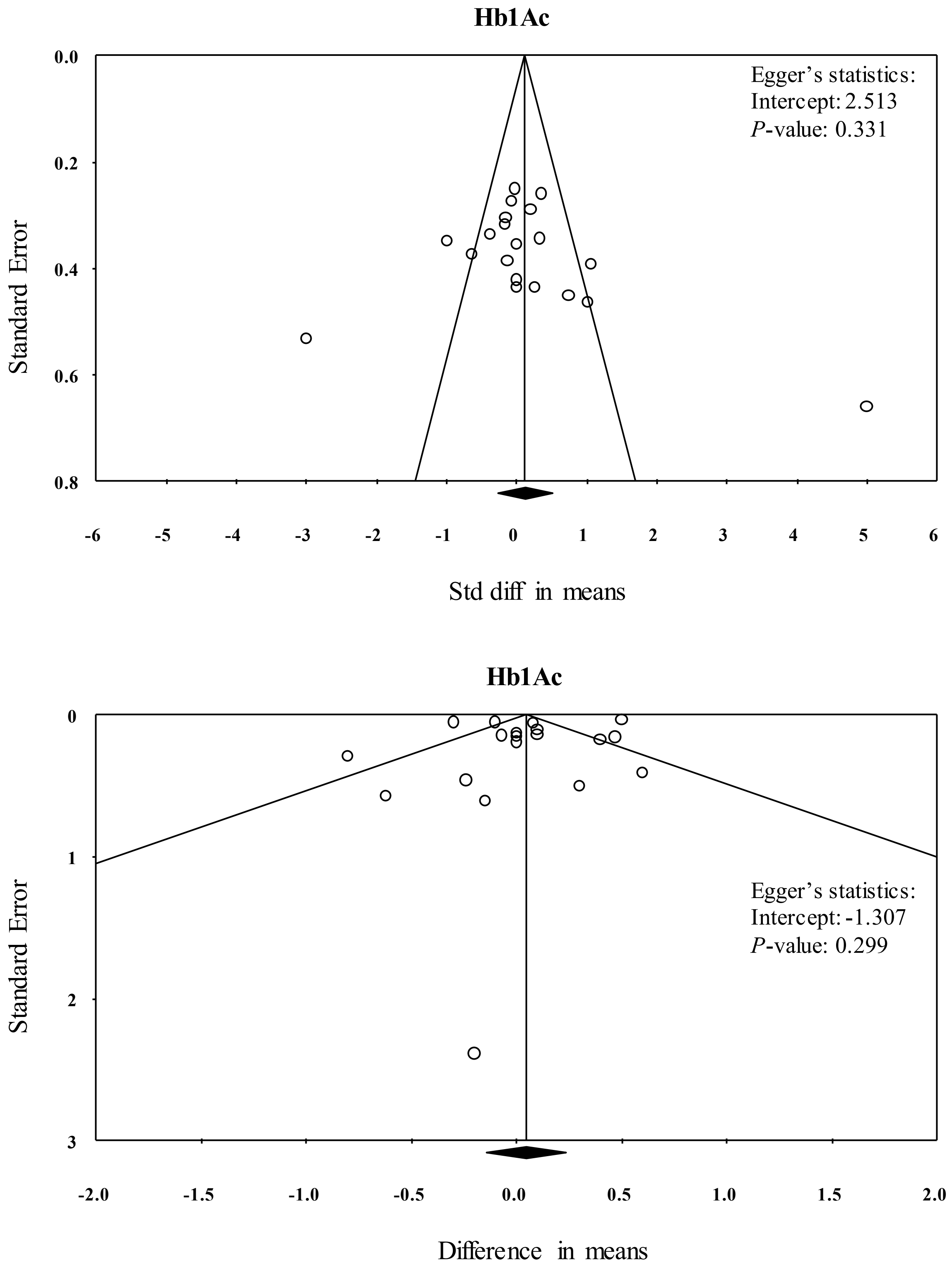

Supplementary Figure S37. Forest plot of the meta-analysis evaluating the effects of a prolonged supplementation with ellagitannins and anthocyanins-containing products on HOMA-IR. A total of 18 studies (displayed in alphabetical order) were analysed. Pooled results are shown at the bottom using a random-effects model. SDM: standardized difference in means, SE: standard error, 95% CI: lower and upper confidence limits for the average SDM, RW: relative weight.

Outcome: HOMA-IR

First autor & year	SDM	SE	95% CI		RW
Basu 2010a	0.468	0.293	-0.106	1.042	5.75
Basu 2014	-0.203	0.366	-0.921	0.514	3.68
Basu 2014	-1.988	0.446	-2.863	-1.113	2.48
Hassellund 2013	-0.762	0.399	-1.544	0.020	3.10
Hosseini 2016	-1.100	0.331	-1.749	-0.451	4.50
Katz 2012	-0.047	0.408	-0.848	0.753	2.96
Lee 2014	-0.002	0.258	-0.508	0.504	7.40
Lekakis 2005	-0.116	0.251	-0.609	0.376	7.81
Ma 2010	0.499	0.444	-0.370	1.369	2.51
Naissides 2006	0.167	0.367	-0.551	0.886	3.67
Novotny 2015	-0.303	0.269	-0.830	0.224	6.82
Parham 2015	-0.208	0.438	-1.067	0.651	2.57
Park 2014	0.473	0.231	0.020	0.926	9.24
Tsang 2012	-0.467	0.383	-1.218	0.284	3.36
Urquiaga 2015	-0.064	0.342	-0.735	0.606	4.22
Zhang 2015	-0.350	0.234	-0.809	0.110	8.99
Zhu 2011	0.061	0.166	-0.264	0.385	18.00
Zunino 2014	0.217	0.409	-0.586	1.019	2.94
**Intervention effect**	**-0.172**	**0.122**	**-0.411**	**0.066**	

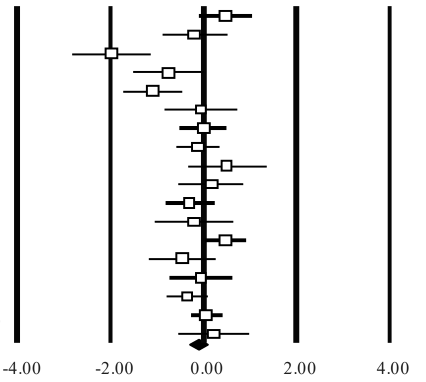

Favours ETs & ANCs **Favours ETs & ANCs**

Test for overall effect Z=-1.416; *P*=0.157; Ns=428; Nc=412

Heterogeneity Tau^2=0.158; Q=46.5; df=17 (*P*=0.000); I^2=63.4

Supplementary Figure S38.- Forest plot of the meta-analysis evaluating the effects of a prolonged supplementation with ellagitannins and anthocyanins-containing products on HOMA-IR. A total of 15 studies (displayed in alphabetical order) were analysed. Pooled results are shown at the bottom using a random-effects model. DM: Difference in means, SE: standard error, 95% CI: lower and upper confidence limits for the average DM, RW: relative weight.

First autor & year	DM	SE	95% CI		RW
Basu 2010a	0.900	0.556	-0.189	1.989	3.54
Basu 2014	-0.200	0.354	-0.894	0.494	8.73
Basu 2014	-0.900	0.234	-1.358	-0.442	20.01
Hosseini 2016	-1.100	0.404	-1.892	-0.308	6.70
Katz 2012	-0.200	1.726	-3.582	3.182	0.37
Lee 2014	-0.010	1.403	-2.760	2.740	0.56
Lekakis 2005	-0.460	0.902	-2.227	1.307	1.35
Ma 2010	0.400	0.350	-0.286	1.086	8.93
Naissides 2006	0.180	0.382	-0.568	0.928	7.51
Novotny 2015	-0.120	0.241	-0.592	0.352	18.85
Parham 2015	-0.180	0.378	-0.921	0.561	7.66
Park 2014	1.160	0.559	0.065	2.255	3.51
Tsang 2012	-0.372	0.352	-1.062	0.318	8.82
Urquiaga 2015	-0.120	0.626	-1.347	1.107	2.79
Zunino 2014	0.760	1.269	-1.728	3.248	0.68
**Intervention effect**	**-0.118**	**0.168**	**-0.4487**	**0.212**	

Outcome: HOMA-IR

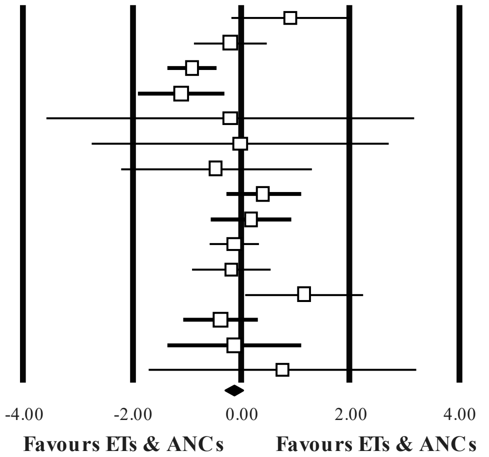

Test for overall effect Z=-0.702; *P*=0.483; Ns=305; Nc=288

Heterogeneity Tau^2=0.182; Q=28.7; df=14 (*P*=0.012); I^2=51.2

Supplementary Figure S39. Funnel plots and Egger statistics (intercept and 2-tailed *P*-value) of the meta-analysis evaluating the effects of a prolonged supplementation with ellagitannins and anthocyanins-containing products on homeostatic model assessment of insulin resistance (HOMA-IR).

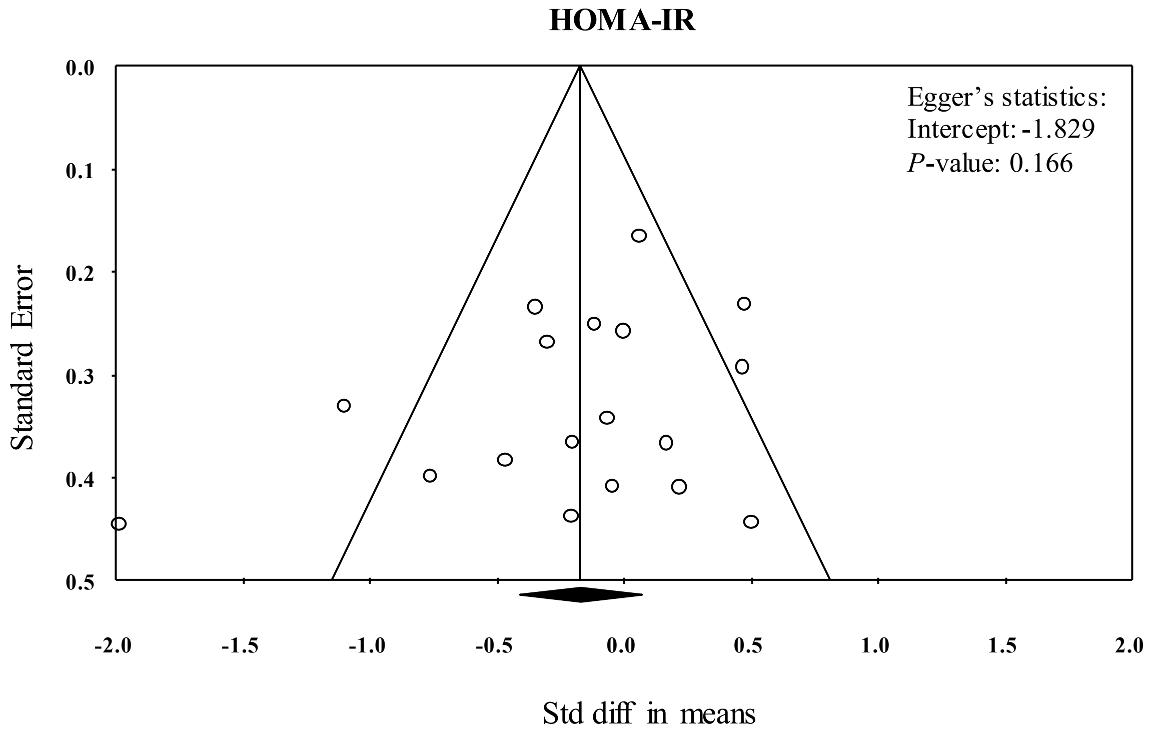

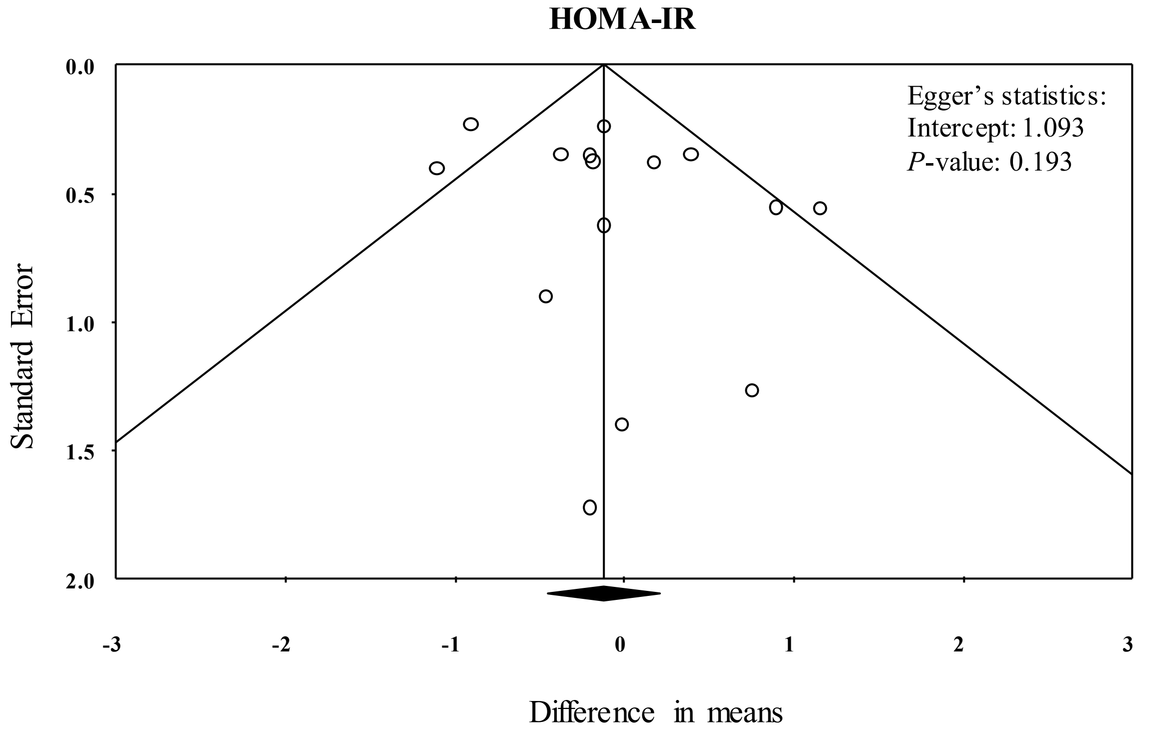

Supplement: Supplementary file 1 [file ijms-19-00694-s001.zip › Supplementary Figures S1-S39.pdf]
