# Supplementary material for: Meta-Analysis of the Effects of Foods and Derived Products Containing Ellagitannins and Anthocyanins on Cardiometabolic Biomarkers: Analysis of Factors Influencing Variability of the Individual Responses
Source: Int J Mol Sci. 2018 Feb 28;19(3):694. doi: 10.3390/ijms19030694 (PMC5877555; doi:10.3390/ijms19030694)
Supplement: Supplementary file 1 [file ijms-19-00694-s001.zip › Supplementary Table S1.pdf]

Table S1. Characteristics of the randomized clinical trials included in this meta-analysis.

First author, year (ref)	N	BMI (< 25 Kg/m², >25 Kg/m², mixed)	Age (range of years)	Gender (male, female, mixed)	Country where the study was conducted	Health status (healthy, disease, mixed)	Menopausal status (pre-, post, mixed)	Smoking (non-smokers, smokers, mixed)	Medication (yes, no)	Background diet (usual, controlled)	Source description	Days of intervention	Risk of biass (score*)
Abu-Amsha Caccetta, 2001 [35]	18	Mixed	25 - 71	Male	Australia	Healthy	NA	Smokers	No	NR	Red wine	63	5
Asgary, 2013 [36]	40	<25	9 - 16	Mixed	Iran	Hyper-lipidemia	No	NR	No	Controlled	Cornus mas	42	8
Asgary, 2014 [37]	21	>25	34 - 63	Mixed	Iran	Hyper-tension	NR	Non-smokers	No	Usual	Pomegranate	14	6
Banini, 2006 [38]	52	Mixed	37 - 75	Mixed	USA	Diabetes	NR	NR	NR	Usual	Juice and wine from muscadine grapes	28	3
Basu, 2014 [39]	60	>25	NR	Mixed	USA	Healthy	Mixed	Non-smokers	No	Usual	Strawberry powder	84	6
Basu, 2011 [40]	31	>25	44 - 60	Mixed	USA	Metabolic syndrome	NR	NR	No	Controlled	Cranberry juice	56	6
Basu, 2010 [41]	48	>25	45 - 55	Mixed	USA	Metabolic syndrome	NR	Non-smokers	No	Controlled	Blueberry	56	5.5
Basu, 2010 [42]	27	>25	NR	Mixed	USA	Metabolic syndrome	NR	Non-smokers	Yes	Usual	Strawberry	56	5
Bommannan Santhakumar, 2015 [43]	13	Mixed	NR	Mixed	Australia	Healthy	NR	Non-smokers	No	Usual	Plums	28	6
Botden, 2012 [44]	61	NR	30 - 65	Male	Chile	Metabolic syndrome	NA	NR	No	Usual	Red wine	112	6.5
Brennan, 2010 [45]	20	>25	40 - 75	Mixed	Israel	Metabolic syndrome	NR	NR	Yes	Controlled	Walnuts	Acute study	5
Burton-Freeman, 2010 [46]	26	>25	NR	Mixed	USA	Hyperlipidemia	NR	Non-smokers	No	Usual	Strawberry	42	5.5
Caldu, 1998 [47]	18	Mixed	25 - 50	Male	Spain	Healthy	NA	Mixed	No	Usual	Red wine	14	4
Castilla, 2008 [48]	32	NR	33 - 79	Mixed	Spain	Hemodialysis patients	NR	NR	No	Controlled	Red grapes	14	5
Castilla, 2006 [49]	38	NR	55 - 65	Mixed	Spain	Mixed (healthy and hemo-dyalisis patients)	NR	NR	Yes	Controlled	Red grape juice	14	5

Cerdá, 2006 [50]	30	>25	50 - 70	Male	Spain	Chronic obstructive pulmonary	NA	Mixed	Yes	Controlled	Pomegranate juice	35	6
Chiva-Blanch, 2012 [51]	73	>25	55 - 75	Male	Spain	Hypertensive	NA	Mixed	Yes	Usual	Red wine	28	7
Clifton, 2004 [52]	36	Mixed	34 - 70	Mixed	Australia	OW	NR	Mixed	Yes	Usual	Grape seed extract	28	7
Cook, 2015 [53]	14	Mixed	NR	Male	UK	Healthy	NA	NR	NR	Usual	Blackcurrant extract	7	3
Cuevas, 2000 [54]	11	<25	20 - 28	Male	Chile	Healthy	NA	Non-smokers	No	Controlled	Red wine	60	4
Curtis, 2009 [55]	57	<25	52 - 64	Female	UK	Healthy	Post-	Non-smokers	No	Usual	Elderberry	84	9
Davinelli, 2015 [56]	42	>25	45 - 65	Mixed	Italy	Healthy	NR	Smokers	No	Usual	Maqui	68	6
Del Bo, 2014 [57]	16	<25	20 - 30	Male	Italy	Healthy	NA	Smokers	No	Controlled	Blueberries	17	4
Del Bo, 2013 [58]	10	<25	NR	Male	Italy	Healthy	NA	Non-Smokers	No	Controlled	Blueberries	2	4.5
Din, 2011 [59]	30	<25	NR	Male	UK	Healthy	NA	Mixed	No	Usual	Walnuts	28	4
Dohadwala, 2011 [60]	47	>25	NR	Mixed	USA	Coronary artery disease	NR	Mixed	Yes	Controlled	Cranberry	28	5.5
Draijer, 2015 [61]	60	NR	NR	Mixed	The Nether-lands	Mildly hypertensive	NR	Non-Smokers	No	Usual	Red grapes	28	4
Duthie, 2006 [62]	20	Mixed	18 - 40	Female	Scotland (UK)	Healthy	Pre-	Mixed	No	Usual	Cranberry juice	14	5
Edirisinghe, 2011 [63]	26	>25	NR	Mixed	United States	Healthy	NR	Non-smokers	No	NR	Strawberry	Acute study	5
Ellis, 2011 [64]	26	>25	40 - 75	Mixed	USA	OW	NR	Non-smokers	No	Usual	Strawberry	24	4.5
Erlund, 2008 [65]	71	NR	NR	Mixed	Finland	Patients with CV risk factor	NR	Non-smokers	No	Usual	Berries	56	5
Estruch, 2011 [66]	42	NR	30 - 50	Male	Spain	Healthy	NA	Mixed	No	Controlled	Red wine	28	5
Flammer, 2013 [67]	84	Mixed	40 - 60	Mixed	USA	Peripheral endothelial dysfunction	NR	Mixed	Yes	NR	Cranberry	120	6
Foster, 2012 [68]	123	>25	18 - 75	Mixed	USA	OW	NR	NR	No	Controlled	Almond	182	4
Fuhrman, 1995 [69]	17	NR	25 - 45	Male	Israel	Healthy	NA	Non-smokers	No	Usual	Red wine	14	2
Goldberg, 1996	24	NR	26 - 45	Male	Canada	Healthy	NA	NR	No	Usual	Red wine	28	3

[70]													
Gorinstein, 2006 [71]	57	NR	39 - 72	NR	Israel	Coronary atherosclerosis	NR	NR	Yes	Usual	Grapefruit	30	4.5
Gulati, 2014 [72]	60	Mixed	NR	Mixed	India	Metabolic syndrome	NR	NR	NR	Controlled	Pistachio	168	3.5
Guo, 2014 [73]	44	>25	18 - 25	Mixed	China	NAFLD	NR	Non-smoker	No	Usual	Bayberry juice	28	6.5
Haddad, 2014 [74]	16	Mixed	23 - 44	Mixed	USA	Healthy	NR	Non-smokers	No	Controlled	Walnut	Acute study	5
Hansen, 2005 [75]	69	Mixed	38 - 74	Mixed	Denmark	Healthy	Mixed	Mixed	No	Controlled	Red wine	28	6
Hassellund 2013 [76]	31	Mixed	35 - 51	Male	Norway	OW	NA	Mixed	Yes	Usual	Bilberries	28	6.5
Heber, 2007 [77]	64	>25	35 - 65	Mixed	USA	Healthy	NR	Mixed	No	Controlled	Pomegranate	30	4
Hijmering, 2007 [78]	20	NR	24 - 45	Mixed	The Nether-lands	Healthy	NR	Non-smokers	No	Usual	Red wine	Acute study	5
Hoggard, 2013 [79]	8	>25	NR	Male	UK	Diabetes	NA	NR	No	Controlled	Bilberry	16	6
Hokayem, 2013 [80]	38	Mixed	30 - 65	Mixed	France	Healthy	Mixed	NR	No	Controlled	Red grape powder	63	8
Hollis, 2013 [81]	86	>25	18 - 50	Mixed	USA	Healthy	NR	Non-smokers	No	NR	Concord grape juice	84	4
Hosseini, 2016 [82]	48	>25	30 - 60	Mixed	Iran	OW	NR	NR	NR	Usual	Pomegranate	30	8.5
Huebbe, 2012 [83]	11	>25	NR	Male	Germany	OW	NA	Non-smokers	Yes	Usual	Red grapes	7	4.5
Jenkins, 2008 [84]	30	Mixed	38-75	Mixed	Canada	Hyperlipidemia	Post-	NR	Yes	Controlled	Strawberry	30	3.5
Jímenez, 2008 [85]	43	Mixed	NR	Mixed	Spain	Hyper-cholesterolemia	NR	Non-smokers	NR	Usual	Red grapes	112	2
Jin, 2011 [86]	20	Mixed	NR	Mixed	UK	Healthy	NR	NR	No	Controlled	Blackcurrant juice	2	5
Johnson, 2015 [87]	48	Mixed	NR	Female	USA	Stage I hypertension	Post-	Mixed	No	Usual	Blueberries	28	6
Johnston, 2013 [88]	51	>25	20 - 65	Mixed	USA	Healthy	NR	Non-smokers	No	Controlled	Peanuts	56	4.5
Karatzi, 2004 [89]	15	>25	NR	Male	Greece	Coronary artery disease	NA	NR	Yes	Controlled	Red grapes	15	8
Karlsen, 2010 [90]	62	>25	30 - 68	Mixed	Norway	OW	Post-	Mixed	No	Usual	Bilberry	30	6

Katz, 2012 [91]	46	>25	NR	Mixed	USA	OW	NR	Non-smokers	Yes	Usual	Walnuts	56	6
Kay, 2002 [92]	8	<25	38 - 54	Male	Canada	Healthy	NA	Non-smokers	No	Usual	Blueberries	Acute study	2
Khan, 2014 [93]	66	Mixed	53	Mixed	UK	Healthy	NR	Mixed	No	Controlled	Blackcurrant Juice	42	7
Kianbakht, 2013 [94]	86	>25	20 - 60	Mixed	Iran	Disease	NR	Non-Smokers	NR	Usual	Berries	120	8
Kolehmainen, 2015 [95]	27	>25	43 - 57	Mixed	Finland	OW	NR	NR	No	Controlled	Bilberries	56	3.5
Krikorian, 2012 [96]	21	NR	68 - 90	Mixed	USA	Mixed (healthy & hypertensive)	NR	NR	Yes	NR	Concord grape	112	6
Krikorian, 2010 [97]	9	NR	71 - 81	Mixed	USA	Age-related memory decline	NR	NR	NR	Usual	Blueberry	84	4
Lamport, 2016 [98]	25	<25	40 - 50	Female	UK	Healthy	Pre-	Non-smokers	NR	NR	Grapes	84	6.5
Lee, 2014 [99]	61	Mixed	35 - 65	Mixed	South Korea	Disease	NR	NR	Yes	Usual	Mixed nuts (raw walnuts, raw pine nuts, roasted peanuts)	42	5.5
Lekakis, 2005 [100]	30	Mixed	50 - 55	Mixed	China	Disease	NR	NR	Yes	Usual	Blackcurrant	168	6
Li, 2010 [101]	70	>25	20 - 65	Mixed	USA	Healthy	NR	NR	No	Controlled	Pistacchio	42	5.5
López-Uriarte, 2010 [102]	50	>25	NR	Mixed	Spain	Metabolic syndrome	NR	NR	Yes	Controlled	Nuts	84	9
Lynn, 2012 [103]	51	Mixed	30 - 50	Mixed	UK	Healthy	NR	Non-smokers	NR	Usual	Pomegranate	30	4.5
Lynn, 2014 [104]	46	Mixed	30 - 50	Mixed	UK	Healthy	NR	Non-smokers	NR	Usual	Pomegranate	30	4.5
Ma, 2010 [105]	24	>25	NR	Mixed	USA	Diabetes	NR	Non-smokers	Yes	Usual	Walnuts	140	5.5
Mathew, 2012 [106]	19	<25	18 - 34	Male	UK	Healthy	NA	Non-smokers	No	Controlled	Pomegranate	Acute study	6
McAnulty, 2014 [107]	20	Mixed	18 - 50	Mixed	USA	OW	Post-	NR	No	Usual	Blueberries	42	9
McAnulty, 2005 [108]	25	>25	18 - 35	NR	USA	OW	NR	Smokers	NR	Controlled	Blueberries	21	4
McKay, 2010 [109]	65	>25	30-70	Mixed	USA	Metabolic syndrome	Mixed	Non-smokers	No	Usual	Hibiscus tea	42	8
Moazen, 2013 [110]	40	Mixed	35 - 60	Mixed	Iran	Diabetes	NR	Non-smokers	No	Usual	Strawberry drink	42	7.5

Mukuddem-Petersen, 2007 [111]	64	>25	40 - 50	Mixed	South africa	Metabolic syndrome	NR	Mixed	Yes	Controlled	Walnuts	56	5.5
Naissides, 2004 [112]	17	Mixed	50 - 70	Female	Australia	Hyper-cholesterolemia	Post-	Non-smokers	No	Usual	Red wine	42	4
Naissides, 2006 [113]	45	Mixed	50-70	Female	Australia	Disease	Post-	Non-smokers	No	Usual	Red wine	42	4
Natella, 2011 [114]	12	NR	24-35	Mixed	Italy	Healthy	NR	NR	No	Usual	Red wine	Acute study	4
Nigdikar, 1998 [115]	30	NR	35 - 65	Male	UK	Healthy	NA	Non-smokers	NR	Usual	Red grapes	14	5
Novotny, 2015 [116]	56	Mixed	25 - 65	Mixed	USA	Healthy	NR	Non-smokers	No	Controlled	Cranberry juice	56	8
Ohguro, 2012 [117]	40	NR	55 - 69	Mixed	Japan	Disease (eye)	NR	NR	Yes	NR	Blackcurrant	730	8
Olmedilla-Alonso, 2008 [118]	25	>25	46 - 62	Mixed	Spain	OW	Post-	Mixed	Yes	Usual	Walnuts	35	6
Parham, 2015 [119]	48	>25	NR	Mixed	Iran	Diabetes	NR	NR	NR	NR	Pistachio	84	4
Park, 2014 [120]	77	>25	20 - 65	Female	Korea	Healthy	NR	NR	No	Usual	Pomegranate vinegar	56	7.5
Park, 2004 [121]	40	>25	41 - 48	Male	South Korea	Healthy	NA	Mixed	NR	Controlled	Concord grape juice	56	5
Preuss, 2000 [122]	40	NR	NR	NR	USA	Hyper-cholesterolemia	NR	NR	NR	NR	Grape seed extract	56	5
Puupponen-Pimiä, 2013 [123]	37	>25	NR	Mixed	Finland	Metabolic syndrome	NR	NR	Yes	Usual	Blackcurrant and bilberry	96	6,5
Qin, 2009 [124]	120	>25	40 - 65	Mixed	China	Dyslipidemia	NR	NR	NR	Usual	Blackcurrant and bilberry	84	7.5
Queipo-OrtuNo, 2012 [125]	10	>25	45 - 50	Male	Spain	Healthy	No	NR	No	Controlled	Red wine	60	9
Rahbar, 2015 [126]	100	Mixed	20 - 70	Mixed	Iran	Hyper-cholesterolemia	NR	NR	No	Usual	Grapes	56	5
Ras, 2013 [127]	70	Mixed	35 - 75	Mixed	The Nether-lands	Healthy	Post-	NR	No	Controlled	Red grape seed	56	6.5
Razavi, 2013 [128]	48	NR	21-64	Mixed	Iran	Hyperlipidemia	NR	Non-smokers	Yes	Usual	Red grape seeds	56	8
Riso, 2013 [129]	18	Mixed	NR	Male	Italy	Patients with CV risk factor	NA	Mixed	No	Usual	Blueberries	42	5.5

Rodriguez-Mateos, 2014 [130]	21	<25	18 - 40	Male	UK	Healthy	NA	NR	No	Controlled	Blueberry	Acute study	8
Ruel, 2013 [131]	35	>25	NR	Male	Canada	OW	NA	Non-Smokers	No	Usual	Cranberries	28	6
Santhakumar, 2015 [132]	13	<25	18 - 65	Mixed	Australia	Healthy	NR	Non-Smokers	No	NR	Plum	28	5.5
Shema-Didi, 2014 [133]	101	>25	NR	Mixed	Israel	Hemodialy-sis patients	NR	NR	Yes	NR	Pomegranate	365	8
Sivaprakasapillai, 2010 [134]	27	>25	25 - 80	Mixed	USA	OW	NR	Non-smokers	No	Usual	Grape seed extract	28	7.5
Spaak, 2006 [135]	13	<25	24 - 47	Mixed	Canada	Healthy	NR	Non-smokers	NR	Controlled	Red wine	Acute study	6.5
Spaccarotella, 2008 [136]	22	Mixed	45 - 75	Male	USA	Healthy	NA	Non-smokers	NR	Usual	Walnuts	56	3
Stull, 2010 [137]	32	>25	46 - 57	Mixed	USA	OW	NR	NR	NR	Usual	Blueberry	42	9
Sumner, 2005 [138]	45	>25	NR	Mixed	USA	Coronary heart disease	NR	Non-smokers	Yes	NR	Pomegranate	90	8
Terauchi, 2014 [139]	96	NR	40 - 60	Female	Japan	NR	Mixed	NR	NR	NR	Red grape seed	28	6
Tey, 2013 [140]	107	>25	18 - 65	Mixed	New Zealand	Healthy	NR	Non-smokers	No	Usual	Hazelnuts	84	6.5
Tomé-Carneiro, 2012 [141]	75	>25	45 - 72	Mixed	Spain	Patients with CV risk factor	NR	Mixed	Yes	Usual	Grapes	184	9.5
Tome-Carneiro, 2013 [142]	35	>25	NR	Male	Spain	Hypertensive and coronary artery disease	NA	NR	Yes	Usual	Red Grape extract	365	7
Tsang, 2005 [143]	25	<25	23 - 50	Mixed	UK	Healthy	NR	Non-smokers	No	Usual	Red wine	14	5
Tsang, 2012 [144]	28	>25	40 - 65	Mixed	UK	Healthy	NR	NR	No	Controlled	Pomegranate juice	28	8
Umar, 2005 [145]	20	NR	20 - 54	Mixed	France	Healthy	NR	NR	No	NR	Armanac *vs* vodka	14	3
Urquiaga, 2015 [146]	47	NR	30 - 65	Mixed	Chile	Metabolic syndrome	NR	NR	No	Usual	Red wine	112	6,5
Vaisman, 2015 [147]	50	>25	35 - 70	Mixed	Israel	Healthy	NR	Mixed	No	Usual	Red grapes	84	5
Velliquette, 2015 [148]	49	>25	40 - 60	Mixed	USA	OW and obese	NR	NR	No	Controlled	Raspberry and grape	Berries	8.5
Vidlar, 2010 [149]	42	Mixed	45 - 70	Male	Czec Republic	Mixed (prostate related problems)	NA	NR	Yes	Usual	Cranberry fruit	90	4

Wang, 2012 [150]	90	>25	>45	Mixed	China	Metabolic syndrome	NR	NR	NR	Usual	Pistachio	84	3.5
Watson, 2015 [151]	36	<25	18 - 34	NR	New Zealand	Healthy	NR	Non-smokers	NR	Usual	Blackcurrant ext racts	Acute study	8
Whelan, 2004 [152]	14	>25	30 - 70	Male	New Zealand	Coronary artery disease	NA	Non-smokers	Yes	Controlled	Wine	21	6
Wien, 2010 [153]	65	>25	42 - 65	Mixed	USA	OW	NR	NR	No	Usual	Almonds	56	5
Williams, 2004 [154]	13	NR	48 - 70	Male	New Zealand	Coronary artery disease	NA	Non-smokers	Yes	Controlled	Red wine	Acute study	4
Wu, 2014 [155]	40	Mixed	50 - 65	Mixed	Germany	Healthy	Post-	Non-smokers	No	Controlled	Walnuts	56	4.5
Wu, 2015 [156]	33	NR	≥30	Mixed	USA	Disease	NR	Mixed	Yes	NR	Pomegranate	180	7
Yubero, 2013 [157]	60	>25	34 - 65	Mixed	Spain	Healthy	NR	NR	NR	Controlled	Red grape	28	7
Zern, 2005 [158]	44	>25	NR	Female	USA	Healthy	Pre-	NR	No	Usual	Red grape powder	28	8
Zhang, 2015 [159]	74	NR	25 - 65	Mixed	China	Nonalcoholic fatty liver disease	NR	NR	No	Usual	Bilberry and blackcurrant	84	9.5
Zhu, 2013 [160]	150	>25	40 - 65	Mixed	China	Hyper-cholesterolemia	NR	Non-smokers	NR	Usual	Bilberry and blackcurrant	150	7.5
Zhu, 2011 [161]	150	Mixed	40 - 60	Mixed	China	Hyper-cholesterolemia	NR	Non-smokers	NR	Usual	Bilberry and blackcurrant	84	5
Zunino, 2014 [162]	33	>25	20 - 60	Mixed	USA	OW	NR	Non-smokers	No	Usual	Red grape	21	6.5

N, Number of participants; BMI, Body mass index; CV, cardiovascular; NA; not applicable; NR, not reported; OW, overweight; NAFLD, Non-alcoholic fatty liver disease.

*Risk of bias score: low risk (≥8 and ≤10), moderate risk (≥5 and <8), high risk (<5).
