# Supplementary material for: Meta-Analysis of the Effects of Foods and Derived Products Containing Ellagitannins and Anthocyanins on Cardiometabolic Biomarkers: Analysis of Factors Influencing Variability of the Individual Responses
Source: Int J Mol Sci. 2018 Feb 28;19(3):694. doi: 10.3390/ijms19030694 (PMC5877555; doi:10.3390/ijms19030694)
Supplement: Supplementary file 1 [file ijms-19-00694-s001.zip › Supplementary Table S2.pdf]

Supplementary Table S2. Overall effect estimations (SDM) of the impact of ellagitannins and (or) anthocyanins containing foods and products on a range of cardiovascular risk associated biomarkers.

	n	N_T	N_S	N_C	SDM	95% CI	Z	*P*-value	Tau²	Q	df (*P*-value)	I^2 (%)	Egger's regression	
													Intercept	*P*-value (2-tailed)
BMI	51	2343	1194	1149	-0.042	-0.123, 0.040	-1.005	*NS*	0.000	14.7	50 (*1.000*)	0.000	0.203	*NS*
WC	23	1026	528	498	-0.296	-0.515, -0.078	-2.659	*0.008*	0.167	60.1	22 (*0.000*)	63.4	-0.707	*NS*
T-C	109	3991	2060	1931	-0.173	-0.272, -0.074	-3.421	*0.001*	0.139	237.4	108 (*0.000*)	54.5	0.389	*NS*
LDL-C	97	3492	1811	1681	-0.099	-0.220, 0.021	-1.620	*NS*	0.217	270.6	96 (*0.000*)	64.5	2.637	*0.000*
HDL-C	99	3581	1858	1723	0.111	0.009, 0.214	2.124	*0.034*	0.133	208.9	98 (*0.000*)	53.1	-1.698	*0.000*
TAGs	97	3602	1859	1743	-0.065	-0.174, 0.043	-1.182	*NS*	0.157	227.7	96 (*0.000*)	57.8	1.560	*0.002*
SBP	95	3539	1812	1717	-0.204	-0.284, -0.124	-5.013	*0.000*	0.037	125.3	94 (*0.017*)	25.0	0.005	*NS*
DBP	99	3790	1959	1831	-0.185	-0.264, -0.106	-4.600	*0.000*	0.042	135.8	98 (*0.007*)	27.9	-0.579	*NS*
FMD	22	563	278	285	0.204	-0.166, 0.574	1.080	*NS*	0.520	80.1	21 (*0.000*)	73.8	-2.820	*0.004*
Glucose	67	2369	1236	1133	-0.101	-0.219, 0.017	-1.671	*0.095*	0.103	122.3	66 (*0.000*)	46.1	-0.427	*NS*
Insulin	34	1236	612	624	-0.042	-0.248, 0.164	-0.402	*NS*	0.229	97.3	33 (*0.000*)	66.1	0.527	*NS*
Hb1Ac	20	732	387	345	0.121	-0.275, 0.517	0.600	*NS*	0.668	120.6	19 (*0.000*)	82.2	2.513	*NS*
HOMA-IR	18	840	428	412	-0.172	-0.411, 0.066	-1.416	*NS*	0.158	46.5	17 (*0.000*)	63.4	-1.892	*NS*

BMI: Body Mass Index; WC: Waist Circumference; T-C: Total Cholesterol; LDL-C: Low density Lipoprotein Cholesterol; HDL-C: High Density Lipoprotein Cholesterol; TAGs: Triacylglycerols; SBP: Systolic Blood Pressure; DBP: Diastolic Blood Pressure; FMD: Flow Mediated Dilation; Hb1Ac: Glycated Haemoglobin; HOMA-IR: Homeostatic Model Assessment of Insulin Resistance; n: total number of studies included in the analysis; N_T: number of total participants; N_S: number of participants in the supplemented group; N_C: number of participants in the control group; SDM: standardized difference in means; 95% CI: lower and upper confidence limits for the average SDM; df: degrees of freedom; Z: statistic for testing the significance of the average SDM; Tau²: between-studies variance; Q: heterogeneity statistic; I^2: heterogeneity index. *NS*: not significant, *P*-values < 0.05 were significant and indicated. *P*-values: 0.05 ≤ *P*-value < 0.1 were considered marginally significant and are also indicated.
