# Supplementary material for: Meta-Analysis of the Effects of Foods and Derived Products Containing Ellagitannins and Anthocyanins on Cardiometabolic Biomarkers: Analysis of Factors Influencing Variability of the Individual Responses
Source: Int J Mol Sci. 2018 Feb 28;19(3):694. doi: 10.3390/ijms19030694 (PMC5877555; doi:10.3390/ijms19030694)
Supplement: Supplementary file 1 [file ijms-19-00694-s001.zip › Supplementary Table S3.pdf]

**Supplementary Table S3.** Overall effect size estimations (DM) of the impact of ellagitannins and (or) anthocyanins containing foods and products on a range of cardiovascular risk associated biomarkers.

| | n | $N_T$ | $N_S$ | $N_C$ | DM | 95% CI | Z | *P*-value | Tau² | Q | df (*P*-value) | $I^2$ (%) | Egger's regression | |
|---|---|---|---|---|---|---|---|---|---|---|---|---|---|---|
| | | | | | | | | | | | | | Intercept | *P*-value (2-tailed) |
| BMI (Kg/m²) | 47 | 2145 | 1095 | 1050 | -0.003 | -0.014, 0.007 | -0.592 | *NS* | 0.000 | 11.7 | 46 (*1.000*) | 0.00 | -0.031 | *NS* |
| WC (cm) | 22 | 972 | 503 | 469 | -1.219 | -2.073, -0.365 | -2.799 | *0.005* | 0.828 | 33.6 | 21 (*0.040*) | 37.5 | 0.152 | *NS* |
| T-C (mmol/L) | 103 | 3673 | 1902 | 1771 | -0.099 | -0.177, -0.020 | -2.417 | *0.013* | 0.078 | 349.4 | 102 (*0.000*) | 70.6 | 0.017 | *NS* |
| LDL-C (mmol/L) | 92 | 3307 | 1718 | 1589 | -0.032 | -0.117, 0.052 | -0.748 | *NS* | 91.0 | 523.8 | 91 (*0.000*) | 82.6 | 0.256 | *NS* |
| HDL-C (mmol/L) | 92 | 3239 | 1692 | 1547 | 0.026 | -0.001, 0.053 | 1.867 | *0.062* | 0.008 | 237.2 | 91 (*0.000*) | 61.6 | -0.412 | *NS* |
| TAGs (mmol/L) | 88 | 2929 | 1525 | 1404 | -0.006 | -0.071, 0.059 | -0.177 | *0.086* | 0.045 | 360.4 | 87 (*0.000*) | 75.9 | 0.312 | *NS* |
| SBP (mm Hg) | 83 | 3175 | 1630 | 1545 | -1.561 | -2.129, -0.994 | -5.394 | *0.000* | 0.000 | 69.0 | 82 (*0.846*) | 0.00 | -0.214 | *NS* |
| DBP (mm Hg) | 90 | 3437 | 1782 | 1655 | -1.417 | -2.079, -0.756 | -4.198 | *0.000* | 2.301 | 152.4 | 89 (*0.000*) | 41.6 | -0.590 | *0.0005* |
| FMD (%) | 21 | 547 | 270 | 277 | 0.635 | 0.073, 1.197 | 2.214 | *0.027* | 0.841 | 112.1 | 20 (*0.000*) | 82.2 | 0.831 | *NS* |
| Glucose (mmol/L) | 60 | 2210 | 1156 | 1054 | -0.033 | -0.093, 0.027 | -1.086 | *NS* | 0.012 | 99.4 | 59 (*0.001*) | 40.6 | 0.147 | *NS* |
| Insulin (mIU/L) | 24 | 900 | 446 | 454 | -0.348 | -1.097, 0.401 | -0.911 | *NS* | 0.869 | 45.6 | 23 (0.003) | 49.6 | 0.139 | *NS* |
| Hb1Ac | 19 | 705 | 374 | 331 | 0.047 | -0.143, 0.236 | 0.482 | *NS* | 0.120 | 242.9 | 18 (*0.000*) | 92.6 | -1.307 | *NS* |
| HOMA-IR | 15 | 593 | 305 | 288 | -0.118 | -0.448, 0.212 | -0.702 | *NS* | 0.182 | 28.7 | 14 (*0.012*) | 51.2 | 1.093 | *NS* |

BMI: Body Mass Index; WC: Waist Circumference; T-C: Total Cholesterol; LDL-C: Low density Lipoprotein Cholesterol; HDL-C: High Density Lipoprotein Cholesterol; TAGs: Triacylglycerols; SBP: Systolic Blood Pressure; DBP: Diastolic Blood Pressure; FMD: Flow Mediated Dilation; Hb1Ac: Glycated Haemoglobin; HOMA-IR: Homeostatic Model Assessment of Insulin Resistance; n: total number of studies included in the analysis; $N_T$: number of total participants; $N_S$: number of participants in the supplemented group; $N_C$: number of participants in the control group; DM: difference in means; 95% CI: lower and upper confidence limits for the average DM; df: degrees of freedom; Z: statistic for testing the significance of the average DM; Tau²: between-studies variance; Q: heterogeneity statistic; $I^2$: heterogeneity index. *NS*: not significant, *P*-values $< 0.05$ were significant and indicated. *P*-values: $0.05 \leq P\text{-value} < 0.1$ were considered marginally significant and are also indicated.
