# Supplementary material for: Meta-Analysis of the Effects of Foods and Derived Products Containing Ellagitannins and Anthocyanins on Cardiometabolic Biomarkers: Analysis of Factors Influencing Variability of the Individual Responses
Source: Int J Mol Sci. 2018 Feb 28;19(3):694. doi: 10.3390/ijms19030694 (PMC5877555; doi:10.3390/ijms19030694)
Supplement: Supplementary file 1 [file ijms-19-00694-s001.zip › Supplementary Table S4.pdf]

Supplementary Table S4. Effect estimations (SDM) and effect size estimations (DM) of the impact of ellagitannins (ETs: pomegranate, nuts) and of anthocyanins (ANCs: berries, red grapes, red wine) containing foods and products on a range of cardiovascular risk associated biomarkers.

	**SDM**	***P*-value**	**n**	I^2	**Q-value**	***P*-value**	**DM**	***P*-value**	**n**	I^2	**Q-value**	***P*-value**
BMI (Kg/m^2)*												
ETs	-0.076	*NS*	17	0.00	0.358	*NS*	-0.003	*NS*	14	0.00	0.001	*NS*
ANCS	-0.023	*NS*	34	0.00			-0.006	*NS*	33	0.00		
WC (cm)												
ETs	-0.693	*0.025*	7	83.0	6.696	*0.010*	-1.527	*0.031*	6	75.9	0.663	*NS*
ANCS	-0.118	*NS*	16	0.00			-0.749	*NS*	16	0.00		
T-C (mmol/L)												
ETs	-0.184	*0.006*	28	18.1	0.005	*NS*	-0.087	*0.000*	26	0.00	0.131	*NS*
ANCS	-0.172	*0.008*	81	60.9			-0.102	*0.094*	77	76.6		
LDL-C (mmol/L)												
ETs	-0.188	*0.031*	26	46.9	0.700	*NS*	-0.107	*0.000*	24	0.00	0.062	*NS*
ANCS	0.054	*NS*	71	68.7			-0.029	*NS*	68	85.7		
HDL-C (mmol/L)												
ETs	0.104	*NS*	23	24.9	0.111	*NS*	0.026	*0.066*	21	0.00	0.191	*NS*
ANCS	0.117	*0.073*	76	58.0			0.027	*NS*	71	68.5		
TAGs (mmol/L)												
ETs	-0.235	*0.025*	26	63.0	4.061	*0.044*	-0.106	*0.000*	24	0.00	1.443	*NS*
ANCS	0.004	*NS*	71	54.2			0.016	*NS*	64	75.6		
SBP (mm Hg)												
ETs	-0.114	*NS*	21	51.1	1.191	*NS*	-1.855	*NS*	15	38.1	2.854	*0.091*
ANCS	-0.228	*0.000*	74	12.4			-2.188	*0.000*	68	0.00		
DBP (mm Hg)												
ETs	-0.141	*NS*	20	38.0	0.410	*NS*	-1.282	*NS*	18	46.2	0.962	*NS*
ANCS	-0.199	*0.000*	79	25.2			-1.580	*0.000*	72	9.3		
FMD (%)												
ETs	0.619	*0.014*	3	0.00	0.836	*NS*	0.388	*NS*	3	40.8	0.011	*NS*
ANCS	0.123	*NS*	19	77.4			0.530	*NS*	18	81.4		
Glucose (mmol/L)												
ETs	-0.241	*0.052*	16	52.0	1.922	*NS*	-0.121	*0.010*	15	14.2	3.100	*0.078*
ANCS	-0.051	*NS*	51	42.7			0.001	*NS*	45	39.6		
Insulin (mIU/L)												
ETs	-0.128	*NS*	12	48.5	0.320	*NS*	-0.214	*NS*	10	45.8	0.003	*NS*
ANCS	0.006	*NS*	22	72.1			-0.364	*NS*	14	55.2		
Hb1Ac												
ETs	0.105	*NS*	6	24.5	0.060	*NS*	0.071	*NS*	6	9.10	0.001	*NS*
ANCS	0.127	*NS*	14	88.6			0.040	*NS*	13	94.9		
HOMA-IR												
ETs	-0.114	*NS*	7	66.8	0.149	*NS*	-0.077	*NS*	7	56.8	0.017	*NS*
ANCS	-0.208	*NS*	11	64.3			-0.148	*NS*	8	50.9		

BMI: Body Mass Index; WC: Waist Circumference; T-C: Total Cholesterol; LDL-C: Low density Lipoprotein Cholesterol; HDL-C: High Density Lipoprotein Cholesterol; TAGs: Triacylglycerols; SBP: Systolic Blood Pressure; DBP: Diastolic Blood Pressure; FMD: Flow Mediated Dilation; Hb1Ac: Glycated Haemoglobin; HOMA-IR: Homeostatic Model Assessment of Insulin Resistance; SDM: standardized difference in means; DM: Difference in means; n: total number of studies included in the analysis; I^2: heterogeneity index; Q-value: between-categories Q statistic. *: Units refer to the size effect estimation (DM); *NS*: not significant, *P*-values < 0.05 were significant and indicated. *P*-values: 0.05 ≤ *P*-value < 0.1 were considered marginally significant and are also indicated.
