# Supplementary material for: Meta-Analysis of the Effects of Foods and Derived Products Containing Ellagitannins and Anthocyanins on Cardiometabolic Biomarkers: Analysis of Factors Influencing Variability of the Individual Responses
Source: Int J Mol Sci. 2018 Feb 28;19(3):694. doi: 10.3390/ijms19030694 (PMC5877555; doi:10.3390/ijms19030694)
Supplement: Supplementary file 1 [file ijms-19-00694-s001.zip › Supplementary Table S5.pdf]

Supplementary Table S5. Stratification by the baseline BMI pf the study participants: influence on the effect estimations (SDM) and size effect estimations (DM) of the impact of ellagitannins (ETs: pomegranate, nuts) and of anthocyanins (ANCs: berries, red grapes, red wine) containing foods and products on a range of cardiovascular risk associated biomarkers.

	**ETs**				**ANCs**			
**BMI**	**< 25.0 Kg/m^2**		**≥ 25.0 Kg/m^2**		**< 25.0 Kg/m^2**		**≥ 25.0 Kg/m^2**	
	SDM (S, n, I^2)	DM (S, n, I^2)	SDM (S, n, I^2)	DM (S, n, I^2)	SDM (S, n, I^2)	DM (S, n, I^2)	SDM (S, n, I^2)	DM (S, n, I^2)
BMI (Kg/m^2)*	-	-	-0.09 (*NS*, 13, 0.00)	-0.003 (*NS*, 11, 0.00)	-0.03 (*NS*, 1, 0.00)	-0.10 (*NS*, 1, 0.00)	-0.05 (*NS*, 21, 0.00)	-0.008 (*NS*, 20, 0.00)
WC (cm)	-	-	-0.70 (*NS*, 4, 88.8)	-1.71 (*0.047*, 3, 46.6)	-	-	-0.11 (*NS*, 10, 0.00)	-0.70 (*NS*, 10, 0.00)
T-C (mmol/L)	-0.34 (*NS*, 1, 0.00)	-0.13 (*NS*, 1, 0.00)	-0.12 (*NS*, 20, 33.8)	-0.08 (*0.000*, 18, 0.00)	+0.14 (*NS*, 6, 0.00)	+0.14 (*NS*, 6, 0.00)	-0.34 (*0.003*, 35, 74.1)	-0.23 (*0.009*, 35, 81.3)
LDL-C (mmol/L)	-0.38 (*NS*, 1, 0.00)	-0.16 (*NS*, 1, 0.00)	-0.15 (*NS*, 20, 56.9)	-0.11, (*0.000*, 18, 0.00)	-0.10 (*NS*, 5, 0.00)	-0.11 (*NS*, 5, 0.00)	-0.13 (*NS*, 30, 69.7)	-0.11 (*NS*, 29, 82.7)
HDL-C (mmol/L)	-0.06 (*NS*, 1, 0.00)	-0.01 (*NS*, 1, 0.00)	+0.06 (*NS*, 17, 36.2)	+0.008 (*NS*, 15, 0.00)	+0.10 (*NS*, 5, 0.00)	+0.02 (*NS*, 5, 0.00)	+0.02 (*NS*, 31, 72.5)	+0.02 (*NS*, 30, 82.0)
TAGs (mmol/L)	+0.13 (*NS*, 1, 0.00)	+0.10 (*NS*, 1, 0.00)	-0.21 (*NS*, 19, 70.2)	-0.11 (*0.000*, 17, 0.00)	+0.14 (*NS*, 5, 0.00)	+0.06 (*NS*, 5, 0.00)	-0.05 (*NS*, 28, 65.6)	-0.05 (*NS*, 27, 60.0)
SBP (mm Hg)	+0.18 (*NS*, 3, 72.6)	-	-0.26 (*0.012*, 14, 35.2)	-3.10 (*0.033*, 11, 39.6)	+0.03 (*NS*, 13, 45.1)	+1.38 (*NS*, 9, 0.00)	-0.25 (*0.000*, 43, 0.58)	-1.54 (*0.000*, 38, 0.00)
DBP (mm Hg)	-	-	-0.08 (*NS*, 15, 38.5)	-0.55 (*NS*, 14, 31.1)	+0.01 (*NS*, 12, 0.00)	+0.33 (*NS*, 11, 0.00)	-0.22 (*0.002*, 30, 22.9)	-1.62 (*0.000*, 29, 21.9)
FMD (%)	-	-	+0.62 (*0.014*, 3, 0.00)	+0.39 (*NS*, 3, 40.8)	+0.14 (*NS*, 8, 66.5)	+0.75 (*NS*, 7, 50.0)	-0.20 (*NS*, 6, 89.5)	-0.65 (*NS*, 6, 89.7)
Glucose (mmol/L)	-	-	-0.19 (*0.058*, 14, 11.0)	-0.18 (*0.017*, 13, 9.2)	-0.14 (*NS*, 5, 0.00)	-0.08 (*NS*, 5, 0.00)	-0.13 (*NS*, 22, 45.1)	-0.02 (*NS*, 20, 44.8)
Insulin (mIU/L)	-	-	-0.17 (*NS*, 10, 45.5)	-1.03 (*NS*, 8, 19.5)	-	-	+0.14 (*NS*, 9, 65.3)	+0.06 (*NS*, 7, 44.5)
Hb1Ac	-	-	+0.03 (*NS*, 5, 25.3)	+0.06 (*NS*, 5, 26.7)	-	-	-0.55 (*NS*, 7, 83.3)	-0.12 (*NS*, 7, 68.9)
HOMA-IR	-	-	-0.14 (*NS*, 6, 72.3)	-0.08 (*NS*, 6, 64.0)	-	-	-0.35 (*NS*, 4, 86.5)	-0.10 (*NS*, 4, 72.7)

BMI: Body Mass Index; WC: Waist Circumference; T-C: Total Cholesterol; LDL-C: Low density Lipoprotein Cholesterol; HDL-C: High Density Lipoprotein Cholesterol; TAGs: Triacylglycerols; SBP: Systolic Blood Pressure; DBP: Diastolic Blood Pressure; FMD: Flow Mediated Dilation; Hb1Ac: Glycated Haemoglobin; HOMA-IR: Homeostatic Model Assessment of Insulin Resistance; SDM: standardized difference in means; DM: Difference in means; n: total number of studies included in the analysis; I^2: heterogeneity index; Q-value: between-categories Q statistic. *: Units refer to the size effect estimation (DM); S: Significance of the analysis; *NS*: not significant, *P*-values < 0.05 were significant and indicated. *P*-values: 0.05 ≤ *P*-value < 0.1 were considered marginally significant and are also indicated.
