# Supplementary material for: Meta-Analysis of the Effects of Foods and Derived Products Containing Ellagitannins and Anthocyanins on Cardiometabolic Biomarkers: Analysis of Factors Influencing Variability of the Individual Responses
Source: Int J Mol Sci. 2018 Feb 28;19(3):694. doi: 10.3390/ijms19030694 (PMC5877555; doi:10.3390/ijms19030694)
Supplement: Supplementary file 1 [file ijms-19-00694-s001.zip › Supplementary Table S6.pdf]

Supplementary Table S6. Stratification by sex: influence on the effects estimations (SDM, DM) of the impact of ellagitannins (ETs: pomegranate, nuts) and of anthocyanins (ANCs: berries, red grapes, red wine) containing foods and products on a range of cardiovascular risk associated biomarkers.

	**ETs**				**ANCs**			
**Sex**	**Women**		**Men**		**Women**		**Men**	
	SDM<br>(S, n, I^2)	DM<br>(S, n, I^2)	SDM<br>(S, n, I^2)	DM<br>(S, n, I^2)	SDM<br>(S, n, I^2)	DM<br>(S, n, I^2)	SDM<br>(S, n, I^2)	DM<br>(S, n, I^2)
BMI<br>(Kg/m^2)*	-0.18<br>(*NS*, 1, 0.00)	-0.18<br>(*NS*, 1, 0.00)	-	-	+0.10<br>(*NS*, 2, 0.00)	+0.25<br>(*NS*, 2, 0.00)	-0.04<br>(*NS*, 9, 0.00)	-0.13<br>(*NS*, 9, 0.00)
WC<br>(cm)	-	-	-	-	-0.45<br>(*NS*, 1, 0.00)	-11.5<br>(*NS*, 1, 0.00)	-0.05<br>(*NS*, 2, 0.00)	-0.7<br>(*NS*, 2, 0.00)
T-C<br>(mmol/L)	-0.11<br>(*NS*, 1, 0.00)	-0.10<br>(*NS*, 1, 0.00)	-0.20<br>(*NS*, 3, 0.00)	-0.12<br>(*NS*, 3, 0.00)	+0.04<br>(*NS*, 6, 0.00)	+0.03<br>(*NS*, 6, 0.00)	-0.05<br>(*NS*, 17, 0.00)	-0.03<br>(*NS*, 16, 0.00)
LDL-C<br>(mmol/L)	-0.12<br>(*NS*, 1, 0.00)	-0.06<br>(*NS*, 1, 0.00)	-0.15<br>(*NS*, 3, 0.00)	-0.12<br>(*NS*, 3, 0.00)	-0.18<br>(*NS*, 3, 0.00)	-0.14<br>(*NS*, 3, 0.00)	+0.08<br>(*NS*, 16, 0.00)	+0.17<br>(*NS*, 15, 0.00)
HDL-C<br>(mmol/L)	-0.29<br>(*NS*, 1, 0.00)	-0.09<br>(*NS*, 1, 0.00)	-0.09<br>(*NS*, 3, 0.00)	-0.01<br>(*NS*, 3, 0.00)	-0.08<br>(*NS*, 4, 0.00)	-0.03<br>(*NS*, 4, 0.00)	-0.008<br>(*NS*, 19, 15.8)	-0.03<br>(*NS*, 18, 0.00)
TAGs<br>(mmol/L)	+0.36<br>(*NS*, 1, 0.00)	+0.29<br>(*NS*, 1, 0.00)	+0.07<br>(*NS*, 3, 0.00)	+0.06<br>(*NS*, 3, 0.00)	+0.10<br>(*NS*, 6, 0.00)	+0.06<br>(*NS*, 6, 0.00)	+0.11<br>(*NS*, 14, 0.00)	+0.15<br>(*NS*, 13, 0.00)
SBP<br>(mm Hg)	-	-	+0.11<br>(*NS*, 4, 61.6)	-1.40<br>(*NS*, 1, 0.00)	-0.18<br>(*NS*, 9, 26.4)	-2.47<br>(*NS*, 9, 19.1)	-0.15<br>(*NS*, 21, 0.00)	-0.40<br>(*NS*, 19, 0.00)
DBP<br>(mm Hg)	-	-	+0.02<br>(*NS*, 2, 0.00)	-0.95<br>(*NS*, 1, 0.00)	-0.19<br>(*0.092*, 8, 7.4)	-1.81<br>(*0.087*, 8, 8.4)	-0.19<br>(*0.017*, 24, 0.00)	-1.70<br>(*0.012*, 22, 0.00)
FMD<br>(%)	-	-	-	-	-	-	-0.24<br>(*NS*, 10, 69.6)	-0.20<br>(*NS*, 9, 69.6)
Glucose<br>(mmol/L)	-0.02<br>(*NS*, 1, 0.00)	-0.19<br>(*NS*, 1, 0.00)	+0.08<br>(*NS*, 1, 0.00)	+0.20<br>(*NS*, 1, 0.00)	+0.14<br>(*NS*, 3, 61.9)	+0.03<br>(*NS*, 3, 58.9)	-0.21<br>(*NS*, 11, 42.5)	-0.12<br>(*NS*, 8, 0.00)
Insulin<br>(mIU/L)	-0.04<br>(*NS*, 4, 76.1)	-0.01<br>(*NS*, 2, 0.00)	-0.16<br>(*NS*, 2, 63.7)	+0.26<br>(*NS*, 1, 0.00)	+0.06<br>(*NS*, 3, 84.1)	+0.56<br>(*NS*, 1, 0.00)	-0.16<br>(*NS*, 2, 63.7)	+0.26<br>(*NS*, 1, 0.00)
Hb1Ac	-	-	-	-	-	-	+0.16<br>(*NS*, 3, 0.00)	+0.11<br>(*NS*, 2, 0.00)
HOMA-IR	+0.47<br>(*0.041*, 1, 0.00)	+1.16<br>(*0.038*, 1, 0.00)	-	-	+0.17<br>(*NS*, 1, 0.00)	+0.18<br>(*NS*, 1, 0.00)	-0.38<br>(*NS*, 2, 43.3)	-0.12<br>(*NS*, 1, 0.00)

BMI: Body Mass Index; WC: Waist Circumference; T-C: Total Cholesterol; LDL-C: Low density Lipoprotein Cholesterol; HDL-C: High Density Lipoprotein Cholesterol; TAGs: Triacylglycerols; SBP: Systolic Blood Pressure; DBP: Diastolic Blood Pressure; FMD: Flow Mediated Dilation; Hb1Ac: Glycated Haemoglobin; HOMA-IR: Homeostatic Model Assessment of Insulin Resistance; SDM: standardized difference in means; DM: Difference in means; n: total number of studies included in the analysis; I^2: heterogeneity index; Q-value: between-categories Q statistic. *: Units refer to the size effect estimation (DM); S: Significance of the analysis; *NS*: not significant, *P*-values < 0.05 were significant and indicated. *P*-values: 0.05 ≤ *P*-value < 0.1 were considered marginally significant and are also indicated.
