# Supplementary material for: Meta-Analysis of the Effects of Foods and Derived Products Containing Ellagitannins and Anthocyanins on Cardiometabolic Biomarkers: Analysis of Factors Influencing Variability of the Individual Responses
Source: Int J Mol Sci. 2018 Feb 28;19(3):694. doi: 10.3390/ijms19030694 (PMC5877555; doi:10.3390/ijms19030694)
Supplement: Supplementary file 1 [file ijms-19-00694-s001.zip › Supplementary Table S7.pdf]

Supplementary Table S7. Stratification by the smoking habits: influence on the effects estimations (SDM, DM) of the impact of ellagitannins (ETs: pomegranate, nuts) and of anthocyanins (ANCs: berries, red grapes, red wine) containing foods and products on a range of cardiovascular risk associated biomarkers.

	ETs				ANCs			
**Sex**	**Non-Smokers**		**Smokers**		**Non-Smokers**		**Smokers**	
	SDM (S, n, I^2)	DM (S, n, I^2)	SDM (S, n, I^2)	DM (S, n, I^2)	SDM (S, n, I^2)	DM (S, n, I^2)	SDM (S, n, I^2)	DM (S, n, I^2)
BMI (Kg/m²)*	+0.08 (*NS*, 6, 0.00*	+0.12 (*NS*, 5, 0.00)	-	-	-0.08 (*NS*, 9, 0.00)	-0.06 (*NS*, 8, 0.00)	+0.17 (*NS*, 1, 0.00)	+0.80 (*NS*, 1, 0.00)
WC (cm)	-0.89 (*NS*, 3, 91.1)	-1.72 (*0.047*, 3, 46.6)	-	-	-0.15 (*NS*, 7, 26.5)	-0.83 (*NS*, 7, 24.7)	+0.10 (*NS*, 1, 0.00)	+0.90 (*NS*, 1, 0.00)
T-C (mmol/L)	-0.11 (*NS*, 10, 0.00)	-0.10 (*NS*, 10, 0.00)	-	-	-0.15 (*NS*, 30, 52.9)	-0.05 (*NS*, 28, 75.1)	+0.03 (*NS*, 3, 0.00)	+0.01 (*NS*, 3, 0.00)
LDL-C (mmol/L)	-0.17 (*NS*, 8, 0.00)	-0.11 (*0.081*, 8, 0.00)	-	-	-0.02 (*NS*, 27, 71.8)	-0.002 (*NS*, 25, 82.8)	+0.01 (*NS*, 3, 0.00)	+0.01 (*NS*, 3, 0.00)
HDL-C (mmol/L)	+0.03 (*NS*, 8, 0.00)	+0.01 (*NS*, 8, 0.00)	-	-	+0.13 (*NS*, 30, 51.2)	+0.03 (*NS*, 27, 59.6)	-0.15 (*NS*, 3, 0.00)	-0.06 (*NS*, 3, 0.00)
TAGs (mmol/L)	-0.09 (*NS*, 9, 0.00)	-0.07 (*NS*, 9, 0.00)	-	-	-0.003 (*NS*, 26, 49.4)	-0.05 (NS, 22, 0.00)	-0.44 (*NS*, 3, 84.5)	-0.32 (*NS*, 3, 0.00)
SBP (mm Hg)	-0.05 (*NS*, 10, 27.7)	-0.18 (*NS*, 8, 0.00)	-	-	-0.26 (*0.008*, 29, 53.3)	-1.99 (*0.025*, 27, 12.5)	-0.42 (*NS*, 3, 66.3)	+1.80 (*NS*, 1, 0.00)
DBP (mm Hg)	-0.27 (*0.035*, 9, 32.0)	-2.14 (*0.092*, 9, 49.0)	-	-	-0.19 (*0.041*, 30, 58.8)	-1.28 (*0.043*, 28, 44.6)	-0.10 (*NS*, 3, 0.00)	-1.01 (*NS*, 2, 0.00)
FMD (%)	+0.62 (*0.014*, 3, 0.00)	+0.39 (*NS*, 3, 40.8)	-	-	+0.19 (*NS*, 7, 80.8)	+0.53 (*NS*, 7, 90.3)	-1.61 (*0.000*, 3, 0.00)	-3.53 (*0.000*, 2, 0.00)
Glucose (mmol/L)	-0.37 (*NS*, 5, 75.8)	-0.11 (*0.000*, 5, 0.00)	-	-	-0.16 (*NS*, 25, 56.4)	-0.04 (*NS*, 24, 60.5)	-	-
Insulin (mIU/L)	-0.36 (*NS*, 5, 73.3)	-0.45 (*NS*, 3, 84.3)	-	-	+0.17 (*NS*, 10, 66.7)	+0.21 (*NS*, 6, 46.3)	-	-
Hb1Ac	-0.13 (*NS*, 3, 0.00)	-0.04 (*NS*, 3, 0.00)	-	-	-0.58 (*NS*, 6, 89.0)	-0.10 (*NS*, 6, 84.9)	-	-
HOMA-IR	+0.20 (*NS*, 2, 0.00)	+0.38 (*NS*, 2, 0.00)	-	-	-0.17 (*NS*, 7, 75.3)	-0.11 (*NS*, 6, 64.7)	-	-

BMI: Body Mass Index; WC: Waist Circumference; T-C: Total Cholesterol; LDL-C: Low density Lipoprotein Cholesterol; HDL-C: High Density Lipoprotein Cholesterol; TAGs: Triacylglycerols; SBP: Systolic Blood Pressure; DBP: Diastolic Blood Pressure; FMD: Flow Mediated Dilation; Hb1Ac: Glycated Haemoglobin; HOMA-IR: Homeostatic Model Assessment of Insulin Resistance; SDM: standardized difference in means; DM: Difference in means; n: total number of studies included in the analysis; I^2: heterogeneity index; Q-value: between-categories Q statistic. *: Units refer to the size effect estimation (DM); S: Significance of the analysis; *NS*: not significant, *P*-values < 0.05 were significant and indicated. *P*-values: 0.05 ≤ *P*-value < 0.1 were considered marginally significant and are also indicated.
