# Supplementary material for: Meta-Analysis of the Effects of Foods and Derived Products Containing Ellagitannins and Anthocyanins on Cardiometabolic Biomarkers: Analysis of Factors Influencing Variability of the Individual Responses
Source: Int J Mol Sci. 2018 Feb 28;19(3):694. doi: 10.3390/ijms19030694 (PMC5877555; doi:10.3390/ijms19030694)
Supplement: Supplementary file 1 [file ijms-19-00694-s001.zip › Supplementary Table S8.pdf]

Supplementary Table S8. Stratification by the background diet during supplementation: influence on the effects estimations (SDM, DM) of the impact of ellagitannins (ETs: pomegranate, nuts) and of anthocyanins (ANCs: berries, red grapes, red wine) containing foods and products on a range of cardiovascular risk associated biomarkers.

	**ETs**				**ANCs**			
**Diet followed during supplementation**	**Usual**		**Controlled**		**Usual**		**Controlled**	
	SDM<br>(S, n, I^2)	DM<br>(S, n, I^2)	SDM<br>(S, n, I^2)	DM<br>(S, n, I^2)	SDM<br>(S, n, I^2)	DM<br>(S, n, I^2)	SDM<br>(S, n, I^2)	DM<br>(S, n, I^2)
BMI<br>(Kg/m²)*	-0.01<br>(*NS*, 11, 0.00)	-0.01<br>(*NS*, 11, 0.00)	-0.27<br>(*0.076*, 4, 0.00)	-0.70<br>(*NS*, 1, 0.00)	-0.03<br>(*NS*, 21, 0.00)	-0.02<br>(*NS*, 20, 0.00)	-0.02<br>(*NS*, 12, 0.00)	-0.01<br>(*NS*, 12, 0.00)
WC<br>(cm)	-0.26<br>(*NS*, 4, 0.00)	-0.60<br>(*0.060*, 3, 0.00)	-1.89<br>(*0.000*, 3, 79.1)	-2.63<br>(*0.000*, 2, 0.00)	-0.15<br>(*0.088*, 10, 5.27)	-1.27<br>(*NS*, 10, 8.21)	-0.04<br>(*NS*, 4, 0.00)	-0.15<br>(*NS*, 4, 0.00)
T-C<br>(mmol/L)	-0.18<br>(*0.028*, 15, 0.00)	-0.13<br>(*0.026*, 14, 0.00)	-0.27<br>(*0.057*, 10, 44.32)	-0.08<br>(*0.000*, 9, 0.00)	-0.13<br>(*0.096*, 53, 55.11)	-0.06<br>(*NS*, 51, 76.81)	-0.32<br>(*0.073* 19, 79.38)	-0.22<br>(*NS*, 19, 80.41)
LDL-C (mmol/L)	-0.12<br>(*NS*, 13, 0.00)	-0.06<br>(*NS*, 12, 0.00)	-0.30<br>(*NS*, 10, 71.56)	-0.11<br>(*0.000*, 9, 0.00)	0.06<br>(*NS*, 45, 75.23)	0.03<br>(*NS*, 44, 88.83)	-0.31<br>(*0.027*, 17, 58.41)	-0.22<br>(*0.016*, 16, 63.19)
HDL-C (mmol/L)	+0.02<br>(*NS*, 11, 0.00)	+0.01<br>(*NS*, 10, 0.00)	+0.13<br>(*NS*, 9, 61.64)	+0.04<br>(*0.056*, 8, 0.00)	+0.10<br>(*NS*, 51, 56.37)	+0.03<br>(*NS*, 48, 58.33)	+0.18<br>(*NS*, 16, 73.13)	+0.04<br>(*NS*, 16, 83.56)
TAGs<br>(mmol/L)	-0.03<br>(*NS*, 13, 0.00)	+0.01<br>(*NS*, 12, 0.00)	-0.47<br>(*0.000*, 10, 76.15)	-0.11<br>(*0.000*, 9, 0.00)	0.08<br>(*NS*, 45, 61.74)	0.04<br>(*NS*, 40, 80.39)	-0.10<br>(*NS*, 17, 40.13)	-0.04<br>(*NS*, 17, 46.76)
SBP<br>(mm Hg)	-0.01<br>(*NS*, 11, 13.89)	-0.34<br>(*NS*, 9, 4.99)	-0.27<br>(*NS*, 6, 63.42)	-3.20<br>(*NS*, 2, 56.16)	-0.19<br>(*0.000*, 42, 0.00)	-1.90<br>(*0.001*, 39, 0.00)	-0.30<br>(*0.002*, 27, 36.02)	-2.57<br>(*0.003*, 25, 0.00)
DBP<br>(mm Hg)	-0.17<br>(*NS*, 11, 6.89)	-1.62<br>(*NS*, 9, 38.90)	+0.16<br>(*NS*, 4, 52.52)	+0.75<br>(*NS*, 4, 11.51)	-0.17<br>(*0.002*, 42, 18.13)	-1.30<br>(*0.001*, 38, 15.22)	-0.24<br>(*0.024*, 31, 32.59)	-2.09<br>(*0.000*, 28, 0.00)
FMD<br>(%)	+0.62<br>(*0.014*, 3, 0.00)	+0.39<br>(*NS*, 3, 40.84)	-	-	+0.44<br>(*NS*, 5, 83.06)	+1.23<br>(*0.084*, 5, 69.65)	+0.08<br>(*NS*, 13, 66.35)	+0.55<br>(*NS*, 12, 62.56)
Glucose<br>(mmol/L)	-0.06<br>(*NS*, 6, 0.00)	-0.04<br>(*NS*, 5, 0.00)	-0.38<br>(*NS*, 8, 67.16)	-0.15<br>(*0.038*, 8, 30.32)	-0.06<br>(*NS*, 31, 39.00)	-0.01<br>(*NS*, 29, 53.56)	-0.16<br>(*NS*, 14, 52.01)	-0.02<br>(*NS*, 12, 0.00)
Insulin<br>(mIU/L)	-0.29<br>(*NS*, 7, 57.58)	-0.76<br>(*NS*, 5, 44.81)	0.00<br>(*NS*, 6, 22.84)	+0.34<br>(*NS*, 5, 35.83)	+0.25<br>(*NS*, 13, 66.29)	+0.45<br>(*NS*, 6, 55.05)	-0.61<br>(*NS*, 5, 85.96)	-0.80<br>(*NS*, 5, 75.50)
Hb1Ac	+0.13<br>(*NS*, 3, 0.00)	+0.06<br>(*NS*, 3, 0.00)	-0.15<br>(*NS*, 1, 0.00)	-0.07<br>(*NS*, 1, 0.00)	-0.21<br>(*NS*, 11, 81.41)	-0.03<br>(*NS*, 11, 75.94)	+2.56<br>(*NS*, 2, 97.75)	+0.32<br>(*NS*, 2, 86.80)
HOMA-IR	-0.04<br>(*NS*, 5, 75.75)	0.08<br>(*NS*, 5, 69.32)	-0.47<br>(*NS*, 1, 0.00)	-0.37<br>(*NS*, 1, 0.00)	-0.23<br>(*NS*, 8, 66.60)	-0.32<br>(*NS*, 6, 38.36)	+0.07<br>(*NS*, 2, 73.40)	-0.27<br>(*NS*, 2, 64.73)

BMI: Body Mass Index; WC: Waist Circumference; T-C: Total Cholesterol; LDL-C: Low density Lipoprotein Cholesterol; HDL-C: High Density Lipoprotein Cholesterol; TAGs: Triacylglycerols; SBP: Systolic Blood Pressure; DBP: Diastolic Blood Pressure; FMD: Flow Mediated Dilation; Hb1Ac: Glycated Haemoglobin; HOMA-IR: Homeostatic Model Assessment of Insulin Resistance; SDM: standardized difference in means; DM: Difference in means; n: total number of studies included in the analysis; I^2: heterogeneity index; Q-value: between-categories Q statistic. *: Units refer to the size effect estimation (DM); S: Significance of the analysis; *NS*: not significant, *P*-values < 0.05 were significant and indicated. *P*-values: 0.05 ≤ *P*-value < 0.1 were considered marginally significant and are also indicated.
