# Supplementary material for: Meta-Analysis of the Effects of Foods and Derived Products Containing Ellagitannins and Anthocyanins on Cardiometabolic Biomarkers: Analysis of Factors Influencing Variability of the Individual Responses
Source: Int J Mol Sci. 2018 Feb 28;19(3):694. doi: 10.3390/ijms19030694 (PMC5877555; doi:10.3390/ijms19030694)
Supplement: Supplementary file 1 [file ijms-19-00694-s001.zip › Supplementary Table S11.pdf]

Supplementary Table S11. Stratification by the source of bioactive compounds: influence on the effects estimations (SDM) and effect size estimation (DM) of the impact of ETs containing pomegranate and nuts, and ANCs containing berries, and red grapes/wine on a range of cardiovascular risk associated biomarkers.

	**ETs**					**ANCs**				
**Source**	**Pomegranate**		**Nuts**			**Berries**		**Red Wine / Red grapes**		
	SDM<br>(S, n, I^2)	DM<br>(S, n, I^2)	SDM<br>(S, n, I^2)	DM<br>(S, n, I^2)	Comparison between subgroups<br>(Q statistic, S)	SDM<br>(S, n, I^2)	DM<br>(S, n, I^2)	SDM<br>(S, n, I^2)	DM<br>(S, n, I^2)	Comparison between subgroups<br>(Q statistic, S)
BMI<br>(Kg/m^2)*	-0.18<br>(*NS*, 5, 0.00)	-0.20<br>(*NS*, 4, 0.00)	-0.04<br>(*NS*, 12, 0.00)	-0.01<br>(*NS*, 10, 0.00)	SDM: 0.82, *NS*<br>DM: 0.78, *NS*	-0.05<br>(*NS*, 16, 0.00)	+0.03<br>(*NS*, 15, 0.00)	-0.14<br>(*NS*, 15, 0.00)	-0.05<br>(*NS*, 15, 0.00)	SDM: 0.01, *NS*<br>DM: 0.01, *NS*
WC<br>(cm)	-0.20<br>(*NS*, 1, 0.00)	-3.90<br>(*NS*, 1, 0.00)	-0.78<br>(*0.027*, 6, 85.10)	-1.51<br>(*0.038*, 5, 80.70)	SDM: 0.40, *NS*<br>DM: 0.08, *NS*	-0.15<br>(*0.088*, 10, 5.43)	-0.94<br>(*NS*, 10, 14.15)	-0.02<br>(*NS*, 6, 0.00)	-0.09<br>(*NS*, 6, 0.00)	SDM: 0.56, *NS*<br>DM: 0.22, *NS*
T-C<br>(mmol/L)	-0.04<br>(*NS*, 11, 0.00)	-0.02<br>(*NS*, 11, 0.00)	-0.32<br>(*0.000*, 17, 2.57)	-0.098<br>(*0.000*, 15, 0.00)	SDM: 10.83, *0.001*<br>DM: 3.31, *0.069*	-0.21<br>(*0.021*, 38, 67.59)	-0.16<br>(*0.093*, 35, 81.53)	-0.14<br>(*NS*, 44, 52.96)	-0.06<br>(*NS*, 43, 67.94)	SDM: 0.30, *NS*<br>DM: 0.70, *NS*
LDL-C<br>(mmol/L)	-0.07<br>(*NS*, 10, 0.00)	-0.05<br>(*NS*, 10, 0.00)	-0.26<br>(*0.047*, 16, 62.28)	-0.11<br>(*0.000*, 14, 0.00)	SDM: 1.31, *NS*<br>DM: 0.66, *NS*	-0.05<br>(*NS*, 31, 74.63)	-0.05<br>(*NS*, 30, 83.56)	-0.06<br>(*NS*, 40, 62.56)	-0.01<br>(*NS*, 38, 87.17)	SDM: 0.07, *NS*<br>DM: 0.13, *NS*
HDL-C<br>(mmol/L)	+0.11<br>(*NS*, 10, 0.00)	+0.01<br>(*NS*, 10, 0.00)	+0.14<br>(*NS*, 13, 44.66)	+0.03<br>(*0.029*, 11, 0.00)	SDM: 0.09, *NS*<br>DM: 0.30, *NS*	+0.17<br>(*NS*, 33, 74.13)	+0.04<br>(*NS*, 30, 82.66)	+0.05<br>(*NS*, 43, 5.60)	+0.02<br>(*NS*, 41, 17.09)	SDM: 1.06, *0.03*<br>DM: 0.52, *NS*
TAGs<br>(mmol/L)	-0.05<br>(*NS*, 10, 0.00)	-0.01<br>(*NS*, 10, 0.00)	-0.33<br>(*0.031*, 16, 71.93)	-0.11<br>(*0.000*, 14, 0.00)	SDM: 1.44, *NS*<br>DM: 1.10, *NS*	-0.10<br>(*NS*, 34, 62.44)	-0.05<br>(*NS*, 28, 60.50)	+0.13<br>(*NS*, 37, 34.63)	+0.07<br>(*NS*, 36, 72.18)	SDM: 3.83, *0.05*<br>DM: 2.46, *NS*
SBP<br>(mm Hg)	-0.09<br>(*NS*, 8, 59.78)	-0.26<br>(*NS*, 6, 56.47)	-0.13<br>(*NS*, 13, 48.51)	-1.63<br>(*NS*, 9, 27.99)	SDM: 0.03, *NS*<br>DM: 0.03, *NS*	-0.25<br>(*0.000*, 38, 37.16)	-2.41<br>(*0.000*, 34, 0.00)	-0.21<br>(*0.000*, 36, 0.00)	-3.31<br>(*0.014*, 34, 0.00)	SDM: 0.11, *NS*<br>DM: 0.35, *NS*
DBP<br>(mm Hg)	-0.46<br>(*0.000*, 8, 0.00)	-4.31<br>(*0.000*, 8, 0.00)	+0.06<br>(*NS*, 12, 0.00)	+0.58<br>(*0.004*, 10, 0.00)	SDM: 12.95, *0.000*<br>DM: 17.32, *0.000*	-0.25<br>(*0.001*, 42, 48.42)	-1.57<br>(*0.002*, 37, 38.28)	-0.16<br>(*0.000*, 39, 26.88)	-1.50<br>(*0.002*, 35, 0.00)	SDM: 0.80, *NS*<br>DM: 0.06, *NS*
FMD<br>(%)	+0.71<br>(*NS*, 1, 0.00)	+0.05<br>(*NS*, 1, 0.00)	+0.58<br>(*0.058*, 2, 0.00)	+1.04<br>(*0.053*, 2, 40.84)	SDM: 0.07, *NS*<br>DM: 3.37, *NS*	+0.46<br>(*NS*, 9, 77.27)	+1.39<br>(*0.011*, 8, 80.08)	-0.19<br>(*NS*, 10, 58.02)	-0.73<br>(*NS*, 10, 64.95)	SDM: 2.89, *NS*<br>DM: 5.68, *NS*
Glucose<br>(mmol/L)	-0.10<br>(*NS*, 7, 0.00)	-0.09<br>(*NS*, 7, 0.00)	-0.36<br>(*0.079*, 9, 67.48)	-0.14<br>(*0.061*, 8, 42.92)	SDM: 1.17, *NS*<br>DM: 0.10, *NS*	-0.05<br>(*NS*, 30, 51.12)	-0.01<br>(*NS*, 26, 39.54)	-0.04<br>(*NS*, 21, 27.67)	+0.01<br>(*NS*, 19, 40.85)	SDM: 0.00, *NS*<br>DM: 0.04, *NS*
Insulin<br>(mIU/L)	+0.09<br>(*NS*, 3, 39.96)	+1.17<br>(*NS*, 3, 41.19)	-0.20<br>(*NS*, 9, 53.19)	-0.39<br>(*NS*, 7, 53.60)	SDM: 0.62, *NS*<br>DM: 0.25, *NS*	-0.01<br>(*NS*, 14, 59.16)	-0.29<br>(*0.082*, 8, 4.07)	-0.04<br>(*NS*, 8, 83.85)	+1.05<br>(*NS*, 6, 75.14)	SDM: 0.04, *NS*<br>DM: 0.03, *NS*
Hb1Ac	-0.17<br>(*NS*, 1, 0.00)	-0.24<br>(*NS*, 1, 0.00)	+0.17<br>(*NS*, 5, 30.72)	+0.08<br>(*NS*, 5, 20.65)	SDM: 0.62, *NS*<br>DM: 0.45, *NS*	-0.63<br>(*0.044*, 7, 82.51)	-0.20<br>(*0.040*, 6, 69.89)	+0.97<br>(*0.038*, 7, 88.49)	+0.26<br>(*0.026*, 7, 78.75)	SDM: 8.59, *0.003*<br>DM: 9.41, *0.002*
HOMA-IR	-0.42<br>(*NS*, 3, 87.63)	-0.17<br>(*NS*, 3, 81.41)	+0.04<br>(*NS*, 4, 0.00)	+0.12<br>(*NS*, 4, 0.00)	SDM: 0.56, *NS*<br>DM: 0.22, *NS*	-0.32<br>(*0.098*, 8, 73.43)	-0.23<br>(*NS*, 5, 65.74)	+0.09<br>(*NS*, 3, 0.00)	+0.14<br>(*NS*, 3, 0.00)	SDM: 1.25, *NS*<br>DM: 0.64, *NS*

BMI: Body Mass Index; WC: Waist Circumference; T-C: Total Cholesterol; LDL-C: Low density Lipoprotein Cholesterol; HDL-C: High Density Lipoprotein Cholesterol; TAGs: Triacylglycerols; SBP: Systolic Blood Pressure; DBP: Diastolic Blood Pressure; FMD: Flow Mediated Dilation; Hb1Ac: Glycated Haemoglobin; HOMA-IR: Homeostatic Model Assessment of Insulin Resistance; SDM: standardized difference in means; DM: Difference in means; n: total number of studies included in the analysis; I^2: heterogeneity index; Q-value: between-categories Q statistic. *: Units refer to the size effect estimation (DM); S: Significance of the analysis; *NS*: not significant, *P*-values < 0.05 were significant and indicated. *P*-values: 0.05 ≤ *P*-value < 0.1 were considered marginally significant and are also indicated.
