# Supplementary material for: Meta-Analysis of the Effects of Foods and Derived Products Containing Ellagitannins and Anthocyanins on Cardiometabolic Biomarkers: Analysis of Factors Influencing Variability of the Individual Responses
Source: Int J Mol Sci. 2018 Feb 28;19(3):694. doi: 10.3390/ijms19030694 (PMC5877555; doi:10.3390/ijms19030694)
Supplement: Supplementary file 1 [file ijms-19-00694-s001.zip › Supplementary Tables S9a-S9b.pdf]

Supplementary Table S9a. Stratification by the health status of the study participants: influence on the effects estimations (SDM, DM) of the impact of ellagitannins (ETs: pomegranate, nuts) containing foods and products on a range of cardiovascular risk associated biomarkers.

Health status	ETs Healthy		At risk		Disease	
	SDM (S, n, I^2)	DM (S, n, I^2)	SDM (S, n, I^2)	DM (S, n, I^2)	SDM (S, n, I^2)	DM (S, n, I^2)
BMI (Kg/m²)*	-0.12 (*NS*, 7, 0.00)	-0.07 (*NS*, 5, 0.00)	-0.09 (*NS*, 2, 0.00)	-0.80 (*NS*, 2, 0.00)	-0.01 (*NS*, 8, 18.81)	-0.00 (*NS*, 7, 21.99)
WC (cm)	-2.47 (*0.000*, 1, 0.00)	-2.60 (*0.000*, 1, 0.00)	-0.15 (*NS*, 2, 0.00)	-0.40 (*NS*, 1, 0.00)	-0.57 (*0.065*, 4, 71.04)	-0.71 (*0.029*, 4, 0.00)
T-C (mmol/L)	-0.21 (*0.041*, 10, 0.00)	-0.15 (*0.028*, 10, 0.00)	-0.30 (*NS*, 5, 78.78)	-0.08 (*0.000*, 4, 0.00)	-0.10 (*NS*, 13, 0.00)	-0.10 (*NS*, 12, 0.00)
LDL-C (mmol/L)	-0.15 (*NS*, 8, 0.00)	-010 (*0.053*, 8, 0.00)	-0.55 (*0.030*, 5, 78.02)	-0.11 (*0.000*, 4, 0.00)	-0.04 (*NS*, 13, 0.00)	-0.02 (*NS*, 12, 0.00)
HDL-C (mmol/L)	-0.03 (*NS*, 8, 0.00)	+0.01 (*NS*, 8, 0.00)	+0.35 (*0.030*, 6, 40.88)	+0.04 (*NS*, 5, 0.00)	+0.01 (*NS*, 9, 21.33)	+0.04 (*NS*, 8, 0.00)
TAGs (mmol/L)	-0.06 (*NS*, 9, 0.00)	-0.05 (*NS*, 9, 0.00)	-0.60 (*NS*, 5, 86.36)	-0.11 (*0.000*, 4, 0.00)	-0.18 (*0.088*, 12, 22.56)	-0.08 (*NS*, 11, 0.00)
SBP (mm Hg)	-0.04 (*NS*, 8, 50.01)	-1.42 (*NS*, 5, 18.50)	-0.40 (*0.004*, 5, 0.00)	-1.15 (*0.003*, 4, 0.00)	-0.02 (*NS*, 8, 64.11)	-1.14 (*NS*, 6, 69.17)
DBP (mm Hg)	-0.24 (*0.096*, 7, 20.00)	-2.69 (*NS*, 6, 37.31)	-0.06 (*NS*, 5, 50.24)	-0.16 (*NS*, 4, 16.80)	-0.11 (*NS*, 8, 48.53)	-0.83 (*NS*, 8, 50.67)
FMD (%)	-	-	+0.55 (*NS*, 1, 0.00)	+1.10 (*NS*, 1, 0.00)	+0.66 (*0.037*, 2, 0.00)	+0.25 (*NS*, 2, 42.43)
Glucose (mmol/L)	-0.62 (*0.023*, 5, 75.59)	-0.23 (*0.016*, 5, 45.05)	-0.06 (*NS*, 3, 79.56)	-0.20 (*NS*, 3, 71.74)	+0.05 (*NS*, 8, 0.00)	+0.05 (*NS*, 8, 0.00)
Insulin (mIU/L)	-0.08 (*NS*, 6, 22.74)	+0.18 (*NS*, 5, 40.11)	-0.06 (*NS*, 3, 79.56)	+0.65 (*NS*, 3, 71.74)	-0.39 (*NS*, 3, 58.93)	-0.76 (*NS*, 2, 0.00)
Hb1Ac	-0.15 (*NS*, 1, 0.00)	-0.07 (*NS*, 1, 0.00)	-0.07 (*NS*, 1, 0.00)	-0.15 (*NS*, 1, 0.00)	+0.25 (*NS*, 4, 39.09)	+0.11 (*NS*, 4, 29.40)
HOMA-IR	+0.05 (*NS*, 2, 77.37)	+0.33 (*NS*, 2, 81.41)	-0.60 (*NS*, 2, 75.06)	-1.05 (*NS*, 2, 0.00)	+0.06 (*NS*, 3, 0.00)	+0.13 (*NS*, 3, 0.00)

Supplementary Table S9b. Stratification by the health status of the study participants: influence on the effects estimations (SDM, DM) of the impact of anthocyanins (ANCs: berries, red grapes, red wine) containing foods and products on a range of cardiovascular risk associated biomarkers.

**Health status**	**ANCs** Healthy		At risk		Disease	
	SDM (S, n, I^2)	DM (S, n, I^2)	SDM (S, n, I^2)	DM (S, n, I^2)	SDM (S, n, I^2)	DM (S, n, I^2)
BMI (Kg/m²)*	+0.01 (*NS*, 14, 0.00)	-0.01 (*NS*, 14, 0.00)	+0.04 (*NS*, 6, 0.00)	+0.04 (*NS*, 6, 0.00)	-0.06 (*NS*, 14, 0.00)	-0.10 (*NS*, 13, 0.00)
WC (cm)	+0.02 (*NS*, 5, 0.00)	+0.19 (*NS*, 5, 0.00)	-0.24 (*0.017*, 7, 0.00)	-1.72 (*0.064*, 7, 23.11)	+0.04 (*NS*, 3, 0.00)	+0.37 (*NS*, 3, 0.00)
T-C (mmol/L)	-0.12 (*NS*, 32, 24.30)	-0.15 (*0.003*, 32, 3.20)	-0.15 (*NS*, 13, 0.00)	-0.07 (*NS*, 11, 0.00)	-0.24 (*0.042*, 36, 77.92)	-0.16 (*NS*, 34, 88.49)
LDL-C (mmol/L)	-0.03 (*NS*, 27, 0.00)	-0.05 (*NS*, 27, 0.00)	+0.16 (*NS*, 14, 69.31)	+0.07 (*NS*, 13, 80.29)	-0.19 (*NS*, 29, 79.49)	-0.13 (*NS*, 27, 92.83)
HDL-C (mmol/L)	+0.08 (*NS*, 30, 0.00)	+0.03 (*NS*, 30, 0.00)	+0.05 (*NS*, 14, 43.11)	-0.04 (*0.068*, 11, 0.00)	+0.16 (*NS*, 32, 75.61)	+0.05 (*NS*, 30, 83.15)
TAGs (mmol/L)	+0.02 (*NS*, 25, 30.26)	+0.03 (*NS*, 25, 32.59)	-0.09 (*NS*, 12, 0.00)	+0.01 (*NS*, 10, 80.39)	+0.04 (*NS*, 34, 70.28)	+0.06 (*NS*, 29, 86.85)
SBP (mm Hg)	-0.26 (*0.006*, 28, 45.87)	-2.37 (*0.004*, *26*, 0.00)	-0.23 (*0.062*, 14, 31.85)	-1.24 (*NS*, 12, 0.00)	-0.19 (*0.001*, 30, 0.00)	-2.55 (*0.001*, 28, 0.00)
DBP (mm Hg)	-0.24 (*0.013*, 28, 42.12)	-1.73 (*0.004*, 25, 9.27)	-0.26 (*0.011*, 13, 9.34)	-1.36 (*0.021*, 12, 0.00)	-0.13 (*0.032*, 35, 11.77)	-1.21 (*0.011*, 32, 18.27)
FMD (%)	+0.18 (*NS*, 11, 64.83)	+0.92 (*0.049*, 10, 56.59)	+0.29 (*NS*, 3, 25.15)	+0.60 (*NS*, 3, 6.75)	+0.12 (*NS*, 4, 89.23)	+0.11 (*NS*, 4, 89.88)
Glucose (mmol/L)	-0.12 (*NS*, 17, 43.73)	-0.03 (*NS*, 15, 48.73)	+0.05 (*NS*, 8, 26.42)	+0.03 (*NS*, 6, 0.00)	-0.03 (*NS*, 25, 48.76)	+0.03 (*NS*, 23, 46.92)
Insulin (mIU/L)	-0.51 (*NS*, 4, 86.07)	-1.35 (*NS*, 4, 67.43)	-0.05 (*NS*, 6, 74.89)	+1.83 (*NS*, 4, 61.67)	+0.21 (*NS*, 11, 66.70)	-0.03 (*NS*, 5, 21.48)
Hb1Ac	+0.50 (*0.000*, 1, 0.00)	+5.00 (*0.000*, 1, 0.00)	+0.04 (*NS*, 3, 0.00)	+1.00 (*NS*, 2, 0.00)	-0.21 (*NS*, 10, 83.61)	-0.02 (*NS*, 10, 79.07)
HOMA-IR	-0.30 (*NS*, 1, 0.00)	-0.12 (*NS*, 1, 0.00)	-0.28 (*NS*, 2, 65.92)	+0.76 (*NS*, 1, 0.00)	+0.19 (*NS*, 8, 71.26)	-0.16 (*NS*, 6, 61.05)

BMI: Body Mass Index; WC: Waist Circumference; T-C: Total Cholesterol; LDL-C: Low density Lipoprotein Cholesterol; HDL-C: High Density Lipoprotein Cholesterol; TAGs: Triacylglycerols; SBP: Systolic Blood Pressure; DBP: Diastolic Blood Pressure; FMD: Flow Mediated Dilation; Hb1Ac: Glycated Haemoglobin; HOMA-IR: Homeostatic Model Assessment of Insulin Resistance; SDM: standardized difference in means; DM: Difference in means; n: total number of studies included in the analysis; I^2: heterogeneity index; Q-value: between-categories Q statistic. *: Units refer to the size effect estimation (DM); S: Significance of the analysis; *NS*: not significant, *P*-values < 0.05 were significant and indicated. *P*-values: 0.05 ≤ *P*-value < 0.1 were considered marginally significant and are also indicated.
