# Supplementary material for: Meta-Analysis of the Effects of Foods and Derived Products Containing Ellagitannins and Anthocyanins on Cardiometabolic Biomarkers: Analysis of Factors Influencing Variability of the Individual Responses
Source: Int J Mol Sci. 2018 Feb 28;19(3):694. doi: 10.3390/ijms19030694 (PMC5877555; doi:10.3390/ijms19030694)
Supplement: Supplementary file 1 [file ijms-19-00694-s001.zip › Supplementary Tables S10a-S10c.pdf]

**Supplementary Table S10a.** Stratification by the country where the study was carried out (Asian countries *vs.* all other countries): influence on the effects estimations (SDM, DM) of the impact of ellagitannins (ETs: pomegranate, nuts) and of anthocyanins (ANCs: berries, red grapes, red wine) containing foods and products on a range of cardiovascular risk associated biomarkers.

| | **ETs** | | | | **ANCs** | | | |
|---|---|---|---|---|---|---|---|---|
| **Country** | East Asian countries | | All other countries | | East Asian countries | | All other countries | |
| | SDM (S, n, $I^2$) | DM (S, n, $I^2$) | SDM (S, n, $I^2$) | DM (S, n, $I^2$) | SDM (S, n, $I^2$) | DM (S, n, $I^2$) | SDM (S, n, $I^2$) | DM (S, n, $I^2$) |
| BMI (Kg/m²)* | -0.08 (*NS*, 4, 15.87) | -0.02 (*NS*, 4, 18.12) | -0.07 (*NS*, 13, 0.00) | +0.06 (*NS*, 10, 0.00) | -0.05 (*NS*, 5, 0.00) | -0.05 (*NS*, 5, 0.00) | -0.01 (*NS*, 29, 0.00) | -0.01 (*NS*, 28, 0.00) |
| WC (cm) | -0.48 (*0.066*, 1, 0.00) | -0.62 (*0.063*, 1, 0.00) | -0.73 (*0.055*, 6, 85.58) | -2.38 (*0.000*, 5, 3.76) | -0.10 (*NS*, 4, 0.00) | -0.78 (*NS*, 4, 0.00) | -0.14 (*NS*, 12, 0.00) | -0.73 (*NS*, 12, 0.00) |
| T-C (mmol/L) | -0.20 (*NS*, 4, 0.00) | -0.19 (*0.050*, 4, 0.00) | -0.17 (*0.029*, 24, 24.88) | -0.08 (*0.000*, 22, 0.00) | -0.45 (*NS*, 6, 0.00) | -0.05 (*NS*, 6, 0.00) | -0.18 (*0.015*, 75, 62.83) | -0.11 (*NS*, 71, 78.11) |
| LDL-C (mmol/L) | +0.07 (*NS*, 4, 0.00) | -0.02 (*NS*, 4, 0.00) | -0.23 (*0.021*, 22, 48.52) | -0.11 (*0.000*, 20, 0.00) | -0.45 (*0.003*, 6, 65.23) | -0.30 (*0.000*, 6, 33.84) | -0.01 (*NS*, 64, 66.77) | 0.00 (*NS*, 61, 84.76) |
| HDL-C (mmol/L) | -0.04 (*NS*, 2, 59.46) | -0.01 (*NS*, 2, 60.50) | +0.13 (*NS*, 21, 21.73) | +0.03 (*0.057*, 19, 0.00) | +0.57 (*0.000*, 6, 0.00) | +0.15 (*0.000*, 6, 0.00) | +0.07 (*NS*, 70, 54.03) | +0.01 (*NS*, 65, 66.67) |
| TAGs (mmol/L) | +0.12 (*NS*, 4, 29.59) | +0.15 (*NS*, 4, 10.79) | -0.32 (*0.006*, 22, 58.96) | -0.11 (*0.000*, 20, 0.00) | -0.11 (*NS*, 6, 0.00) | -0.02 (*NS*, 3, 0.00) | +0.03 (*NS*, 65, 57.75) | +0.02 (*NS*, 61, 76.68) |
| SBP (mm Hg) | -0.04 (*NS*, 1, 0.00) | -0.43 (*NS*, 1, 0.00) | -0.12 (*NS*, 20, 53.34) | -2.12 (*NS*, 14, 42.33) | -0.22 (*0.004*, 10, 0.00) | -3.08 (*0.008*, 9, 0.00) | -0.23 (*0.000*, 64, 19.15) | -2.02 (*0.000*, 59, 0.00) |
| DBP (mm Hg) | - | - | -0.14 (*NS*, 20, 38.05) | -1.28 (*NS*, 16, 46.21) | -0.08 (*NS*, 9, 0.00) | -0.66 (*NS*, 9, 0.00) | -0.22 (*0.000*, 69, 26.88) | -1.66 (*0.000*, 62, 9.18) |
| FMD (%) | - | - | +0.62 (*0.014*, 3, 0.00) | +0.39 (*NS*, 3, 40.84) | +1.17 (*0.000*, 2, 35.93) | +2.19 (*0.000*, 2, 64.06) | -0.27 (*NS*, 3, 78.22) | -0.86 (*NS*, 3, 80.08) |
| Glucose (mmol/L) | +0.02 (*NS*, 2, 0.00) | +0.03 (*NS*, 2, 0.00) | -0.29 (*0.041*, 14, 53.82) | -0.15 (*0.007*, 13, 16.55) | +0.03 (*NS*, 4, 0.00) | +0.09 (*NS*, 4, 0.00) | -0.07 (*NS*, 47, 46.35) | -0.01 (*NS*, 41, 44.10) |
| Insulin (mIU/L) | -0.21 (*NS*, 2, 0.00) | -1.38 (*NS*, 2, 0.00) | -0.11 (*NS*, 10, 57.61) | +0.01 (*NS*, 8, 57.13) | +0.15 (*NS*, 3, 81.07) | -4.55 (*0.042*, 1, 0.00) | -0.02 (*NS*, 19, 72.16) | -0.23 (*NS*, 13, 52.90) |
| Hb1Ac | +0.35 (*NS*, 1, 0.00) | +0.08 (*NS*, 1, 0.00) | +0.03 (*NS*, 5, 25.26) | +0.06 (*NS*, 5, 26.70) | -0.02 (*NS*, 1, 0.00) | -0.20 (*NS*, 1, 0.00) | +0.15 (*NS*, 13, 89.44) | +0.04 (*NS*, 12, 95.29) |
| HOMA-IR | +0.25 (*NS*, 2, 46.82) | +1.00 (*0.054*, 2, 0.00) | -0.31 (*NS*, 5, 58.00) | -0.29 (*NS*, 5, 50.15) | -0.09 (*NS*, 3, 3.24) | -0.46 (*NS*, 1, 0.00) | -0.27 (*NS*, 8, 72.63) | -0.12 (*NS*, 7, 57.80) |

**Supplementary Table S10b.** Stratification by the country where the study was carried out (North America *vs.* all European countries): influence on the effects estimations (SDM, DM) of the impact of ellagitannins (ETs: pomegranate, nuts) and of anthocyanins (ANCs: berries, red grapes, red wine) containing foods and products on a range of cardiovascular risk associated biomarkers.

| **Country** | **ETs** | | | | **ANCs** | | | |
|---|---|---|---|---|---|---|---|---|
| | North America | | All European countries | | North America | | All European countries | |
| | SDM (S, n, $I^2$) | DM (S, n, $I^2$) | SDM (S, n, $I^2$) | DM (S, n, $I^2$) | SDM (S, n, $I^2$) | DM (S, n, $I^2$) | SDM (S, n, $I^2$) | DM (S, n, $I^2$) |
| BMI (Kg/m$^2$)* | +0.04 (*NS*, 5, 14.24) | +0.27 (*NS*, 5, 0.00) | -0.19 (*NS*, 4, 0.00) | -0.09 (*NS*, 1, 0.00) | +0.01 (*NS*, 11, 0.00) | +0.03 (*NS*, 11, 0.00) | -0.01 (*NS*, 16, 0.00) | -0.01 (*NS*, 16, 0.00) |
| WC (cm) | -0.59 (*NS*, 5, 85.60) | -1.98 (*0.002*, 4, 20.56) | - | - | -0.23 (*NS*, 7, 26.64) | -1.56 (*NS*, 7, 38.25) | +0.01 (*NS*, 4, 0.00) | +0.14 (*NS*, 4, 0.00) |
| T-C (mmol/L) | -0.23 (*NS*, 11, 52.24) | -0.08 (*0.000*, 10, 0.00) | -0.23 (*NS*, 6, 0.00) | -0.14 (*0.079*, 6, 0.00) | -0.20 (*0.098*, 24, 57.46) | -0.13 (*NS*, 23, 77.81) | -0.18 (*0.017*, 33, 23.61) | -0.17 (*0.000*, 31, 4.92) |
| LDL-C (mmol/L) | -0.30 (*NS*, 10, 70.09) | -0.11 (*0.000*, 9, 0.00) | -0.23 (*NS*, 4, 0.00) | -0.12 (*0.069*, 4, 0.00) | +0.10 (*NS*, 21, 61.22) | +0.04 (*NS*, 20, 74.28) | -0.13 (*NS*, 28, 25.07) | -0.07 (*NS*, 27, 26.98) |
| HDL-C (mmol/L) | +0.08 (*NS*, 10, 43.99) | -0.01 (*NS*, 9, 0.00) | +0.08 (*NS*, 4, 0.00) | +0.02 (*NS*, 4, 0.00) | -0.05 (*NS*, 23, 12.64) | -0.03 (*0.036*, 21, 0.00) | +0.06 (*NS*, 29, 38.94) | +0.01 (*NS*, 27, 23.14) |
| TAGs (mmol/L) | -0.39 (*0.070*, 11, 74.64) | -0.11 (*0.000*, 10, 0.00) | -0.83 (*NS*, 4, 0.00) | -0.06 (*NS*, 4, 0.00) | -0.02 (*NS*, 23, 56.34) | -0.03 (*NS*, 22, 18.69) | -0.07 (*NS*, 22, 41.08) | 0.00 (*NS*, 20, 45.60) |
| SBP (mm Hg) | 0.00 (*NS*, 7, 62.81) | +0.56 (*NS*, 6, 41.52) | -0.06 (*NS*, 7, 58.91) | -2.40 (*NS*, 3, 58.60) | -0.36 (*0.002*, 21, 54.97) | -2.97 (*0.001*, 19, 14.43) | -0.19 (*0.007*, 29, 0.00) | -1.57 (*0.083*, 27, 0.00) |
| DBP (mm Hg) | -0.09 (*NS*, 7, 63.71) | -1.00 (*NS*, 6, 63.86) | -0.33 (*0.097*, 6, 26.57) | -5.21 (*0.048*, 5, 11.51) | -0.36 (*0.005*, 21, 64.62) | -2.23 (*0.001*, 20, 55.38) | -0.18 (*0.004*, 36, 0.00) | -1.33 (*0.014*, 31, 0.00) |
| FMD (%) | +0.56 (*0.058*, 2, 0.00) | +1.04 (*0.053*, 2, 0.00) | - | - | -0.13 (*NS*, 1, 0.00) | -0.65 (*NS*, 1, 0.00) | -0.06 (*NS*, 9, 75.81) | -0.06 (*NS*, 8, 75.81) |
| Glucose (mmol/L) | -0.15 (*NS*, 8, 33.13) | -0.13 (*NS*, 7, 40.93) | -0.66 (*NS*, 3, 83.00) | -0.11 (*0.000*, 3, 0.00) | -0.12 (*NS*, 15, 48.47) | -0.02 (*NS*, 13, 22.74) | -0.12 (*NS*, 16, 48.47) | -0.11 (*NS*, 12, 46.88) |
| Insulin (mIU/L) | -0.38 (*0.065*, 6, 47.63) | -1.28 (*NS*, 4, 34.76) | +0.50 (*NS*, 2, 71.35) | 3.04 (*0.027*, 2, 0.00) | +0.21 (*0.086*, 10, 8.58) | +0.59 (*NS*, 7, 0.00) | -0.75 (*0.050*, 5, 84.85) | -0.87 (*NS*, 4, 81.40) |
| Hb1Ac | -0.11 (*NS*, 4, 0.00) | -0.04 (*NS*, 4, 0.00) | - | - | -0.63 (*NS*, 4, 91.41) | -0.09 (*NS*, 4, 80.35) | +1.48 (*0.099*, 4, 93.76) | +0.50 (*0.000*, 3, 0.00) |
| HOMA-IR | +0.20 (*NS*, 2, 0.00) | +0.37 (*NS*, 2, 0.00) | -0.47 (*NS*, 1, 0.00) | -0.37 (*NS*, 1, 0.00) | -0.33 (*NS*, 5, 82.17) | -0.16 (*NS*, 5, 67.86) | -0.76 (*0.056*, 1, 0.00) | - |

**Supplementary Table S10c.** Stratification by the country where the study was carried out (Mediterranean countries *vs.* Non-Mediterranean countries): influence on the effects estimations (SDM, DM) of the impact of ellagitannins (ETs: pomegranate, nuts) and of anthocyanins (ANCs: berries, red grapes, red wine) containing foods and products on a range of cardiovascular risk associated biomarkers.

| **Country** | **ETs** | | | | **ANCs** | | | |
|---|---|---|---|---|---|---|---|---|
| | Mediterranean countries | | Non-Mediterranean countries | | Mediterranean countries | | Non-Mediterranean countries | |
| | SDM<br>(S, n, $I^2$) | DM<br>(S, n, $I^2$) | SDM<br>(S, n, $I^2$) | DM<br>(S, n, $I^2$) | SDM<br>(S, n, $I^2$) | DM<br>(S, n, $I^2$) | SDM<br>(S, n, $I^2$) | DM<br>(S, n, $I^2$) |
| BMI<br>(Kg/m²)* | -0.26<br>(*NS*, 1, 0.00) | - | -0.16<br>(*NS*, 3, 0.00) | -0.09<br>(*NS*, 1, 0.00) | 0.00<br>(*NS*, 12, 0.00) | -0.01<br>(*NS*, 12, 0.00) | -0.02<br>(*NS*, 4, 0.00) | -0.11<br>(*NS*, 4, 0.00) |
| WC<br>(cm) | - | - | - | - | +0.01<br>(*NS*, 4, 0.00) | +0.14<br>(*NS*, 4, 0.00) | - | - |
| T-C<br>(mmol/L) | -0.07<br>(*NS*, 2, 0.00) | -0.07<br>(*NS*, 2, 0.00) | -0.29<br>(*0.085*, 4, 0.00) | -0.15<br>(*0.079*, 4, 0.00) | -0.24<br>(*NS*, 15, 51.26) | -0.20<br>(*0.030*, 15, 22.26) | -0.13<br>(*NS*, 18, 0.00) | -0.10<br>(*0.088*, 16, 0.00) |
| LDL-C<br>(mmol/L) | +0.11<br>(*NS*, 1, 0.00) | +0.09<br>(*NS*, 1, 0.00) | -0.33<br>(*NS*, 3, 0.00) | -0.13<br>(*0.054*, 3, 0.00) | -0.35<br>(*0.002*, 14, 13.18) | -0.19<br>(*0.003*, 14, 16.55) | +0.09<br>(*NS*, 14, 0.00) | +0.07<br>(*NS*, 13, 0.00) |
| HDL-C<br>(mmol/L) | -0.14<br>(*NS*, 1, 0.00) | -0.06<br>(*NS*, 1, 0.00) | +0.14<br>(*NS*, 3, 0.00) | +0.02<br>(*NS*, 3, 0.00) | -0.01<br>(*NS*, 14, 0.00) | +0.01<br>(*NS*, 14, 0.00) | +0.14<br>(*NS*, 15, 66.09) | +0.01<br>(*NS*, 13, 60.13) |
| TAGs<br>(mmol/L) | +0.04<br>(*NS*, 1, 0.00) | +0.03<br>(*NS*, 1, 0.00) | -0.12<br>(*NS*, 3, 0.00) | -0.07<br>(*NS*, 3, 0.00) | -0.13<br>(*NS*, 14, 46.53) | -0.08<br>(*NS*, 14, 56.28) | +0.02<br>(*NS*, 8, 33.33) | +0.10<br>(*NS*, 6, 0.00) |
| SBP<br>(mm Hg) | +0.04<br>(*NS*, 5, 68.16) | -1.89<br>(*NS*, 2, 77.04) | -0.31<br>(*NS*, 2, 0.00) | -5.30<br>(*NS*, 1, 0.00) | -0.20<br>(*0.072*, 14, 0.00) | -2.34<br>(*NS*, 13, 0.00) | -0.19<br>(*0.025*, 18, 0.00) | -1.48<br>(*NS*, 17, 0.00) |
| DBP<br>(mm Hg) | -0.18<br>(*NS*, 2, 0.00) | -2.13<br>(*NS*, 2, 0.00) | -0.44<br>(*NS*, 4, 54.18) | -7.93<br>(*0.084*, 3, 58.05) | -0.19<br>(*0.054*, 13, 0.00) | -1.86<br>(*0.019*, 12, 0.00) | -0.17<br>(*0.028*, 23, 0.00) | -0.87<br>(*NS*, 19, 0.00) |
| FMD<br>(%) | - | - | - | - | -0.67<br>(*NS*, 5, 79.16) | -0.54<br>(*NS*, 4, 71.16) | +0.54<br>(*0.006*, 13, 54.55) | +1.21<br>(*0.002*, 13, 61.87) |
| Glucose<br>(mmol/L) | +0.11<br>(*NS*, 2, 0.00) | +0.14<br>(*NS*, 2, 0.00) | -1.03<br>(*0.097*, 2, 81.81) | -0.11<br>(*0.000*, 2, 0.00) | -0.44<br>(*NS*, 7, 76.46) | -0.71<br>(*0.092*, 3, 85.38) | +0.02<br>(*NS*, 9, 0.00) | -0.01<br>(*NS*, 9, 0.00) |
| Insulin<br>(mIU/L) | -0.04<br>(*NS*, 1, 0.00) | +0.90<br>(*NS*, 1, 0.00) | +0.50<br>(*NS*, 2, 71.35) | +3.04<br>(*0.027*, 2, 0.00) | -2.30<br>(*0.000*, 1, 0.00) | -3.80<br>(*0.000*, 1, 0.00) | -0.40<br>(*NS*, 4, 69.96) | -0.20<br>(*NS*, 3, 33.41) |
| Hb1Ac | - | - | - | - | +2.06<br>(*NS*, 3, 94.64) | +0.50<br>(*0.000*, 3, 0.00) | -0.13<br>(*NS*, 1, 0.00) | - |
| HOMA-IR | - | - | -0.47<br>(*NS*, 1, 0.00) | -0.37<br>(*NS*, 1, 0.00) | - | - | -0.76<br>(*0.056*, 1, 0.00) | - |

BMI: Body Mass Index; WC: Waist Circumference; T-C: Total Cholesterol; LDL-C: Low density Lipoprotein Cholesterol; HDL-C: High Density Lipoprotein Cholesterol; TAGs: Triacylglycerols; SBP: Systolic Blood Pressure; DBP: Diastolic Blood Pressure; FMD: Flow Mediated Dilation; Hb1Ac: Glycated Haemoglobin; HOMA-IR: Homeostatic Model Assessment of Insulin Resistance; SDM: standardized difference in means; DM: Difference in means; n: total number of studies included in the analysis; $I^2$: heterogeneity index; Q-value: between-categories Q statistic. *: Units refer to the size effect estimation (DM); S: Significance of the analysis; *NS*: not significant, *P*-values < 0.05 were significant and indicated. *P*-values: 0.05 ≤ *P*-value < 0.1 were considered marginally significant and are also indicated.
